# Trends in Food Sources and Diet Quality Among US Children and Adults, 2003-2018

**DOI:** 10.1001/jamanetworkopen.2021.5262

**Published:** 2021-04-12

**Authors:** Junxiu Liu, Renata Micha, Yan Li, Dariush Mozaffarian

**Affiliations:** 1 Department of Population Health Science and Policy, Icahn School of Medicine at Mount Sinai, New York, New York; 2 Friedman School of Nutrition Science and Policy, Tufts University, Boston, Massachusetts; 3 Department of Obstetrics, Gynecology, and Reproductive Science, Icahn School of Medicine at Mount Sinai, New York, New York

## Abstract

**Importance:**

Time trends and population disparities in nutritional quality of foods from major US sources, including grocery stores, restaurants, schools, worksites, and other sources, are not well established.

**Objective:**

To investigate patterns and trends in diet quality by food sources among US children and adults overall and in sociodemographic subgroups.

**Design, Setting, and Participants:**

This serial, cross-sectional survey study included respondents from 8 National Health and Nutrition Examination Survey cycles (2003-2018) with valid dietary recalls. Data were analyzed from April 16, 2020, to July 20, 2020.

**Exposures:**

Survey cycle, food source, and key sociodemographic subgroups.

**Main Outcomes and Measures:**

Mean diet quality of foods (meals, snacks, and beverages) consumed per person, characterized by the American Heart Association diet score (range, 0-80, with higher scores indicating healthier diets), the Healthy Eating Index 2015 (range, 0-100, with higher scores indicating healthier diets), and their components. For the American Heart Association diet score, poor diet was defined as less than 40.0% adherence (score, <32.0), intermediate diet as 40.0% to 79.9% adherence (score, 32.0-63.9), and ideal as 80.0% or greater adherence (score, ≥64.0).

**Results:**

The study included 20 905 children 5 to 19 years of age (mean [SD] age, 12.1 [5.24] years; 51.0% male) and 39 757 adults 20 years or older (mean [SD] age, 47.3 [15.1] years; 51.9% female). Diet quality of foods consumed from grocery stores increased modestly in children (53.2% to 45.1% with poor diet quality; *P* = .006 for trend) and adults (40.1% to 32.9% with poor diet quality; *P* = .001 for trend), with smaller changes for restaurants among children (84.8% to 79.6% with poor diet quality; *P* = .003 for trend). Changes for restaurants among adults were not statistically significant (65.4% to 65.2% with poor diet quality; *P* = .07 with poor diet quality); the same was true of worksites (adults: 55.6% to 50.7% with poor diet quality; *P* = .25 for trend). Food quality from other sources worsened (children: 40.0% to 51.7% with poor diet quality; adults: 33.8% to 43.8% with poor diet quality; *P* < .001 for trend each). The largest improvement in diet quality was in schools, with the percentage with poor diet quality decreasing from 55.6% to 24.4% (*P* < .001 for trend), mostly after 2010, and with equitable improvements across population subgroups. Findings were similar for Healthy Eating Index 2015. Significant disparities in diet quality trends were seen by sex, race/ethnicity, educational level, and household income for food consumed from grocery stores. For example, the proportion of foods consumed from grocery stores that were of poor diet quality decreased among high-income adults (from 36.9% to 26.5%; *P* = .001 for trend) but not among low-income adults (from 45.8% to 41.3%; *P* = .09 for trend).

**Conclusions and Relevance:**

By 2017-2018, foods consumed at schools improved significantly and provided the best mean diet quality of major US food sources, without population disparities. Additional improvements are needed from all major US food sources, with particular attention on equity.

## Key Points

**Question:**

What are the trends in nutritional quality of foods consumed from major US sources?

**Findings:**

In this survey study of 20 905 children and 39 757 adults from 2003-2004 to 2017-2018, modest improvements were found in diet quality for foods from grocery stores and small improvements for foods from restaurants, each with disparities. Diet quality for foods from schools improved significantly, especially after 2010, and equitably across subgroups; by 2017-2018, food consumed at schools had the highest quality, followed by food from grocery stores, other sources, worksites, and restaurants.

**Meaning:**

By 2017-2018, foods consumed at schools provided the best mean quality of major sources, without disparities, although further improvements are needed in all sources, especially restaurants, with a focus on reducing disparities.

## Teaser

This survey study investigates patterns and trends in diet quality by food sources among US children and adults overall and in sociodemographic subgroups.

## Introduction

Suboptimal dietary habits are a major contributor to death and disability.^1,2,3^ Nearly half of US cardiometabolic deaths and many cases of cancer are estimated to be attributable to poor diet.^2^ Many studies^4,5,6,7^ have reported that measures of overall diet quality have modestly improved in recent years in US children and adults. However, 32% of adults and 49% of children continue to have overall poor diet quality.^4,7^

Of importance, foods and beverages are obtained from diverse sources, including grocery stores, restaurants, worksites, schools, and other sources (eg, entertainment venues, food trucks), yet patterns and trends in diet quality of foods consumed from these different sources are not well established. In addition, overall dietary disparities persist or have worsened by sociodemographic status,^4,5,6,7^ but potential differences in these disparities by food source are unclear. Children typically receive many calories at school and early childcare through the National School Lunch Program, School Breakfast Program, and Child and Adult Care Food Program,^8^ for which several policies have aimed to improve nutrition,^9^ yet diet quality of foods consumed in restaurants has generally not improved,^10^ and grocery stores remain the principal source of US calories.^11^ However, no prior studies, to our knowledge, have separately evaluated trends in nutritional quality of foods consumed from each of these different sources. Recent disruptions in supply chains and food security from COVID-19 further amplify relevance of understanding how nutritional quality may vary from different food sources and for different population subgroups. We investigated patterns and trends in diet quality of foods consumed from different major sources among US children (5-19 years of age) and adults (≥20 years of age) between 2003-2004 and 2017-2018, both overall and by population subgroups.

## Methods

### Study Population and Dietary Assessment

This investigation assessed 8 cross-sectional cycles of the nationally representative National Health and Nutrition Examination Survey (NHANES) 2003-2004 to 2017-2018 (eAppendix 1 in the Supplement).^12,13,14^ Data were analyzed from April 16, 2020, to July 20, 2020. Dietary assessments included up to two 24-hour diet recalls for which respondents reported all meals, snacks, and beverages (hereafter referred to as food) consumed during the previous 24 hours (midnight to midnight). Starting in 2003-2004, each participant reported the source of each item (where purchased or otherwise obtained^15^) grouped for this analysis as (1) grocery stores (grocery or supermarket); (2) restaurants (fast food/pizza or full service); (3) schools for children (kindergarten through 12th grade cafeteria or childcare center) or worksites for adults (cafeteria, vending machine, common coffee pot, or snack tray); and (4) other sources (eg, from someone else or gift, entertainment facility, food truck, or sports or recreation facility). In secondary analyses, we also separately assessed full-service vs fast-food/quick-serve restaurants and schools restricted to kindergarten through 12th grade. All study participants provided written informed consent. The data are publicly available and deidentified; therefore, per the Common Rule, this study was exempt from intitutional review board approval. This study followed the American Association for Public Opinion Research (AAPOR) reporting guideline.^13^

### Dietary Quality

For all food consumed by each person from each source, we calculated 2 established diet quality scores: the American Heart Association (AHA) diet score and Healthy Eating Index (HEI) 2015^16,17^ (eAppendix 1 in the Supplement). The AHA score was selected because it has been validated against risk of major disease outcomes in multiple populations and is a largely food-based score that is readily translated to the public and policymakers.^16^ We used the expanded continuous score with 8 components (total fruits and vegetables; whole grains; fish and shellfish; nuts, seeds, and legumes; sugar-sweetened beverages [SSBs]; processed meat; sodium; and saturated fat), each scored from 0 (worse) to 10 (better) and summed to range from 0 to 80 (with higher scores indicating healthier diets) (eTable 1 in the Supplement). On the basis of the AHA 2020 Strategic Goals, poor diet quality was defined as less than 40.0% adherence (<32.0 points), intermediate diet quality as 40.0% to 79.9% adherence (32.0-63.9 points), and ideal as 80.0% or greater adherence (≥64.0 points).^4,18^

The HEI-2015 measures adherence to the 2015-2020 Dietary Guidelines based on 9 adequacy components (total fruit, whole fruit, total vegetables, greens or beans, whole grains, dairy, total protein foods, seafood or plant protein, and fatty acids) and 4 moderation components (refined grains, sodium, added sugars, and saturated fat). Each component is scored from 0 to 5 or 10, summed to range from 0 to 100 (with higher scores indicating healthier diets) (eTable 2 in the Supplement). Poor, intermediate, and ideal cutoff thresholds for HEI-2015 are not defined.

Because optimal intake levels for each component in these scores are based on a 2000-kcal/d diet, we scaled food intakes from each source to 2000 kcal/d for scoring purposes; the AHA and HEI-2015 dietary targets for a 2000-kcal/d diet were equivalently scaled to the calories consumed by each individual from each food source.

### Population Subgroups

We examined subpopulations susceptible to diet-related health disparities by age (5-11, 12-19, 20-49, and ≥50 years), sex, race/ethnicity (non-Hispanic White, non-Hispanic Black, Hispanic, or other than non-Hispanic White, non-Hispanic Black, and Hispanic, including multiracial), educational level (less than high school graduate, high school graduate or General Educational Development, some college, or college graduate or above), and household income (low income, defined as families with income <1.30 of the ratio of family income to the federal poverty level [PIR]; middle income, PIR = 1.30-3.49; and high income, PIR ≥ 3.50) (eAppendix 2 in the Supplement).

### Statistical Analysis

Analysis incorporated NHANES sampling weights for diet, which consider sampling, stratification, and clustering to provide nationally representative estimates.^14^ The analyses estimate the mean per person quality of food as actually consumed (not as offered or potentially available) from each source on any given day in the US. Survey-weighted estimates for each food source, by cycle, included (1) mean proportions of calories consumed; (2) mean AHA and HEI-2015 total and component scores; and (3) proportions of individuals meeting poor, intermediate, or ideal AHA diet quality targets. Twenty-four-hour recalls provide unbiased estimates of stratum-specific means but can overestimate or underestimate proportions of specific individuals with habitual intake above or below a certain threshold; prior work^7^ has found such effects to be minor. A survey-weighted linear regression model was used to estimate trends, assessing the 2-year survey cycle as a continuous variable. Sensitivity analyses excluded fish and shellfish, not commonly consumed, from the AHA score. Potential differences in trends by subpopulations were tested using survey-weighted Wald *F* statistics to test for interaction between the 2-year survey cycles and subpopulation indicators. Absolute differences in means and proportions were calculated between 2003-2004 and 2017-2018. Analyses were performed using Stata statistical software, version 14.0 (StataCorp LLC), with a 2-sided *P* < .05 indicating statistical significance.

## Results

### Participant Characteristics

The investigation included 20 905 US children 5 to 19 years of age (mean [SD] age, 12.1 [5.24] years; 51.0% male) and 39 757 adults 20 years or older (mean [SD] age, 47.3 [15.1] years; 51.9% female) (Table 1). On any given day during this period, 99.4% of children and 99.5% of adults consumed foods from grocery stores, 61.7% of children and 61.9% of adults from restaurants, 42.4% of children from schools and 12.4% of adults from worksites, and 47.1% of children and 50.4% of adults from other sources.

**Table 1.** Sociodemographic Characteristics and Proportions of Calories of US Children and Adults Consuming Food From Grocery Stores, Restaurants, Schools, Worksites, and Other Sources, NHANES 2003-2018^a^

	Children (5-19 years of age)					Adults (≥20 years of age)				
	Overall (n = 20 905)	Grocery stores (n = 20 785)	Restaurants (n = 12 903)	Schools (n = 8860)	Others (n = 9848)	Overall (n = 39 757)	Grocery stores (n = 39 550)	Restaurants (n = 24 610)	Worksites (n = 4915)	Others (n = 20 036)
Population characteristics										
Age, mean (SD), y	12.1 (5.24)	12.1 (5.23)	12.5 (5.32)	10.9 (4.89)	11.9 (5.13)	47.3 (15.1)	47.4 (15.1)	45.3 (14.2)	42.6 (12.1)	48.1 (14.9)
Sex, No. (%)										
Male	10 556 (51.0)	10 486 (51.0)	6478 (50.1)	4493 (51.2)	4815 (48.9)	19 279 (48.1)	19 156 (48.0)	12 162 (49.8)	2607 (54.9)	9629 (48.2)
Female	10 349 (49.0)	10 299 (49.0)	6425 (49.9)	4367 (48.9)	5033 (51.1)	20 478 (51.9)	20 394 (51.9)	12 448 (50.2)	2308 (45.1)	10 407 (51.8)
Race/ethnicity, No. (%)										
Non-Hispanic										
White	5921 (56.5)	5891 (56.6)	3753 (58.1)	1960 (47.0)	3085 (59.3)	17 481 (67.7)	17 408 (67.8)	10 938 (68.4)	2011 (67.1)	9235 (70.1)
Black	5738 (14.3)	5704 (14.3)	3598 (14.2)	2670 (17.7)	2456 (12.4)	8511 (11.4)	8450 (11.3)	5459 (11.5)	1270 (12.9)	4000 (10.1)
Hispanic	7097 (20.9)	7061 (20.9)	4242 (19.6)	3430 (26.9)	3201 (19.7)	9895 (13.6)	9845 (13.6)	5888 (13.2)	1156 (12.8)	4787 (12.8)
Other^b^	2149 (8.22)	2129 (8.16)	1310 (8.11)	800 (8.37)	1106 (8.64)	3870 (7.33)	3847 (7.31)	2325 (6.93)	478 (7.19)	2014 (7.01)
Educational level, No. (%)^c^										
Less than high school	5676 (19.6)	5653 (19.6)	3196 (17.6)	2613 (23.7)	2450 (18.0)	9992 (16.1)	9956 (16.1)	5122 (13.5)	817 (10.5)	4448 (14.0)
High school or GED	10 750 (55.5)	10 679 (54.5)	6754 (55.7)	4605 (56.6)	5087 (54.5)	9264 (23.9)	9191 (23.8)	5725 (23.4)	1080 (21.7)	4434 (22.4)
Some college						11 616 (31.7)	11 551 (31.8)	7763 (32.9)	1650 (34.0)	5997 (32.0)
College graduate and above	3690 (24.9)	3672 (25.0)	2461 (26.7)	1342 (19.7)	1968 (27.5)	8844 (28.3)	8812 (28.3)	5985 (30.2)	1364 (33.7)	5134 (31.6)
Ratio of family income to poverty level										
<1.30	8457 (33.0)	8398 (33.0)	4698 (28.9)	3934 (39.5)	3712 (28.6)	11 329 (21.8)	11 269 (21.8)	6246 (19.3)	1040 (15.3)	5197 (19.4)
1.30-3.49	7022 (36.9)	6988 (37.0)	4491 (37.5)	3020 (37.6)	3415 (35.7)	13 970 (35.7)	13 892 (35.6)	8622 (34.6)	1704 (33.7)	7037 (34.8)
≥3.50	4008 (30.0)	3994 (30.1)	2834 (33.6)	1367 (22.9)	2721 (35.6)	11 241 (42.5)	11 191 (42.6)	7892 (46.0)	1869 (51.0)	1566 (45.8)
Proportions of calories, mean (95% CI), %										
Overall		65.7 (65.0-66.3)	18.5 (17.9-19.1)	8.50 (7.88-9.12)	7.33 (6.93-7.73)		68.9 (68.4-69.4)	21.4 (20.9-21.9)	1.55 (1.42-1.67)	8.13 (7.82-8.44)
By sex, mean (95% CI), %										
Male		66.3 (65.4-67.2)	18.0 (17.3-18.8)	8.57 (7.84-9.31)	7.10 (6.60-7.61)		67.2 (66.6-67.9)	23.0 (22.4-23.6)	1.66 (1.50-1.82)	8.15 (7.77-8.53)
Female		65.0 (64.2-65.8)	19.0 (18.3-19.7)	8.42 (7.78-9.06)	7.57 (7.09-8.05)		70.5 (69.9-71.1)	20.0 (19.4-20.5)	1.44 (1.29-1.59)	8.12 (7.77-8.46)
By race/ethnicity, mean (95% CI), %										
Non-Hispanic										
White		66.6 (65.5-67.6)	19.0 (18.1-19.8)	6.65 (5.84-7.45)	7.81 (7.20-8.42)		68.8 (68.1-69.5)	21.3 (20.6-22.0)	1.47 (1.32-1.62)	8.38 (7.97-8.78)
Black		63.2 (61.7-64.7)	18.7 (17.9-19.5)	11.9 (10.6-13.2)	6.21 (5.47-6.94)		67.2 (66.1-68.3)	23.2 (22.3-24.1)	2.14 (1.78-2.51)	7.47 (7.03-7.91)
Hispanic		64.6 (63.5-65.7)	17.5 (16.6-18.3)	11.3 (10.4-12.1)	6.70 (6.19-7.21)		69.8 (68.8-70.7)	21.1 (20.2-21.9)	1.46 (1.25-1.67)	7.71 (7.30-8.11)
Other^b^		66.4 (64.6-68.3)	17.7 (16.1-19.2)	8.28 (6.92-9.65)	7.62 (6.45-8.79)		70.6 (69.4-71.8)	20.2 (19.1-21.4)	1.50 (1.23-1.77)	7.68 (6.97-8.40)
By educational level, mean (95% CI), %										
Less than high school		66.0 (65.0-67.0)	16.1 (15.1-17.0)	11.0 (10.2-11.9)	6.91 (6.31-7.52)		74.1 (73.3-74.9)	17.3 (16.6-18.0)	1.08 (0.88-1.28)	7.52 (6.99-8.04)
High school or GED		64.6 (63.7-65.6)	19.2 (18.4-20.0)	8.89 (8.22-9.55)	7.28 (6.73-7.84)		68.9 (68.0-69.8)	21.5 (20.6-22.3)	1.37 (1.21-1.53)	8.24 (7.74-8.75)
Some college							67.4 (66.6-68.2)	22.7 (22.1-23.4)	1.76 (1.50-2.01)	8.11 (7.64-8.58)
College graduate and above		67.5 (66.1-68.8)	19.0 (17.7-20.3)	5.93 (5.07-6.79)	7.62 (6.94-8.30)		67.7 (66.8-68.9)	22.2 (21.4-23.0)	1.73 (1.53-1.92)	8.41 (7.93-8.89)
By ratio of family income to poverty level, mean (95% CI), %^d^										
<1.30		65.4 (64.1-66.7)	16.2 (15.2-17.2)	11.6 (10.7-12.4)	6.76 (6.05-7.48)		71.7 (70.4-73.0)	18.9 (18.0-19.7)	1.31 (0.96-1.66)	8.12 (7.65-8.59)
1.30-3.49		65.6 (64.5-66.6)	18.6 (17.8-19.5)	8.15 (7.40-8.90)	7.66 (6.99-8.34)		69.5 (68.7-70.3)	21.0 (20.3-21.8)	1.49 (1.34-1.65)	7.95 (7.53-8.36)
≥3.50		65.8 (64.4-67.2)	20.9 (19.8-22.1)	5.70 (4.77-6.64)	7.58 (6.87-8.30)		66.6 (65.8-67.4)	23.4 (22.6-24.2)	1.77 (1.58-1.96)	8.28 (7.83-8.72)

Abbreviations: GED, General Educational Development; NHANES, National Health and Nutrition Examination Surveys.

^a^ The top half of the table reports column percentages for sociodemographic compositions overall and by food sources (grocery stores, restaurants, worksites, and others). The bottom half of the table reports row percentages for calories from each of those food sources overall and by sociodemographic groups. Food sources were grouped as grocery stores (consumed food items obtained from grocery or supermarket), restaurants (restaurant fast food/pizza, restaurant with servers, or restaurant no additional information), schools (kindergarten through 12th grade school cafeteria or childcare center), worksites (cafeteria not in a kindergarten through 12th grade school, vending machine, common coffee pot, or snack tray) and other sources refer to food items obtained from elsewhere (sports, recreation, or entertainment facility, vending machine, community food program, from someone else or gift, and others). All analyses incorporate the NHANES complex sampling design and survey weights to provide nationally representative estimates.

^b^ Other includes race/ethnicity other than non-Hispanic White, non-Hispanic Black, and Hispanic, including multiracial.

^c^ For children, educational level refers to parental/household educational levels. Because of the existing categorization in NHANES 2017-2018 (less than high school, high school graduate or GED or some college, and college graduate and above), we combined the high school graduate or GED and some college together for previous NHANES cycles from 2003-2004 to 2015-2016.

^d^ Participants with missing data on educational level (n = 41), parental educational level (n = 789), and household income (n = 4635) were excluded in the subgroup analysis.

### Trends in Energy Intake by Food Source

From 2003-2004 to 2017-2018, calories from grocery stores decreased from 67.8% (95% CI, 66.0%-69.5%) to 64.6% (95% CI, 62.2%-66.9%) in children (*P* < .001 for trend). Calories from restaurants (19.3%; 95% CI, 17.9%-20.7% in 2003-2004 and 20.3%; 95% CI, 18.2%-22.4% in 2017-2018; *P* = .13 for trend) and schools (7.9%; 95% CI, 6.1%-9.8% in 2003-2004 and 6.9%; 95% CI, 4.9%-8.9%; *P* = .82 for trend) remained stable. Calories from other sources increased from 5.0% (95% CI, 4.5%-5.5%) to 8.2% (95% CI, 6.5%-10.0%; *P* < .001 for trend) (eFigure 1 and eTables 3 and 4 in the Supplement).

Among adults, calories from grocery stores decreased modestly from 68.8% (95% CI, 67.6%-70.1%) to 67.3% (95% CI, 65.6%-68.9%; *P* = .002 for trend). Calories from restaurants remained stable at 22.2% (95% CI, 65%.6%-68.9%) in 2003-2004 and 22.5% (95% CI, 20.8%-24.2%; *P* = .75 for trend). Calories from worksites were low and decreased from 2.1% (95% CI, 1.6%-2.6%) to 1.3% (95% CI, 1.1%-1.6%; *P* < .001 for trend). Calories from other sources increased from 6.9% (95% CI, 6.3%-7.4%) to 8.9% (95% CI, 8.0%-9.8%; *P* < .001 for trend) (eFigure 1 and eTables 3 and 4 in the Supplement).

### Patterns and Trends in Diet Quality by Food Sources Among US Children

During this period among children, the mean AHA score increased for food consumed from grocery stores from 31.2 (95% CI, 30.4-32.0) to 34.3 (95% CI, 32.9-35.7) (*P* < .001 for trend); for restaurants, the mean AHA score increased from 24.7 (95% CI, 24.1-25.4) to 26.1 (95% CI, 25.3-27.0) (*P* = .03 for trend); and for schools, the mean AHA score increased from 31.3 (95% CI, 30.2-32.5) to 39.5 (95% CI, 38.9-40.1) (*P* < .001 for trend) but decreased for other sources from 35.0 (95% CI, 34.3-35.8) to 33.1 (95% CI, 31.8-34.3) (*P* = .004 for trend) (Table 2). Secondary analysis findings were similar for HEI-2015 (eTable 5 in the Supplement) and for schools limited to kindergarten through 12th grade (eTable 6 in the Supplement). As 1 exception, diet quality of food consumed from grocery stores was stable (2017-2018 vs 2003-2004 difference, −0.20; 95% CI, −2.0 to 1.54), but slightly worse at restaurants (2017-2018 vs 2003-2004 difference, −1.40; 95% CI, −2.40 to −0.53). This difference was mainly from increased refined grains from these sources, captured in the HEI-2015 but not the AHA score.

**Table 2.** Trends in Diet Quality of Foods Consumed From Different Sources Among US Children 5 to 19 Years of Age, NHANES 2003-2018^a^

Diet components (scoring range)	Survey-weighted AHA score, mean (95% CI)								*P* value for trend	Difference (95% CI) for 2017-2018 vs 2003-2004
	2003-2004 (n = 3236)	2005-2006 (n = 3318)	2007-2008 (n = 2445)	2009-2010 (n = 2596)	2011-2012 (n = 2462)	2013-2014 (n = 2495)	2015-2016 (n = 2383)	2017-2018) (n = 1970)		
AHA diet score (0-80)										
Grocery stores	31.2 (30.4 to 32.0)	32.7 (31.2 to 34.2)	32.8 (31.5 to 34.1)	33.7 (33.2 to 34.2)	34.8 (33.6 to 36.1)	34.1 (33.0 to 35.2)	34.0 (32.9 to 35.1)	34.3 (32.9 to 35.7)	<.001	3.13 (1.54 to 4.72)
Restaurants	24.7 (24.1 to 25.4)	26.1 (25.5 to 26.6)	25.2 (24.5 to 25.9)	26.5 (25.5 to 27.4)	26.3 (25.7 to 27.0)	26.1 (25.4 to 26.8)	25.8 (25.0 to 26.5)	26.1 (25.3 to 27.0)	.03	1.40 (0.34 to 2.47)
Schools	31.3 (30.2 to 32.5)	30.6 (29.3 to 31.9)	30.4 (29.4 to 31.4)	31.8 (30.5 to 33.1)	33.6 (32.2 to 35.0)	37.0 (36.2 to 37.9)	38.0 (37.1 to 38.9)	39.5 (38.9 to 40.1)	<.001	8.20 (6.92 to 9.48)
Other sources	35.0 (34.3 to 35.8)	33.6 (32.5 to 34.8)	33.3 (32.2 to 34.5)	33.3 (32.7 to 34.0)	32.7 (31.8 to 33.6)	32.3 (31.3 to 33.4)	32.9 (32.1 to 33.7)	33.1 (31.8 to 34.3)	.004	−1.90 (−3.40 to −0.47)
Fruits and vegetables (0-10)										
Grocery stores	3.95 (3.77 to 4.13)	4.15 (3.94 to 4.37)	4.05 (3.70 to 4.40)	4.26 (4.01 to 4.51)	4.35 (4.06 to 4.65)	4.31 (4.09 to 4.53)	4.06 (3.72 to 4.39)	4.08 (3.70 to 4.45)	.51	0.13 (−0.29 to 0.54)
Restaurants	3.41 (3.25 to 3.56)	3.21 (3.04 to 3.39)	2.99 (2.76 to 3.22)	3.02 (2.85 to 3.19)	2.97 (2.74 to 3.20)	2.65 (2.49 to 2.81)	2.72 (2.52 to 2.92)	2.52 (2.36 to 2.68)	<.001	−0.88 (−1.10 to −0.66)
Schools	4.63 (4.07 to 5.20)	4.19 (3.95 to 4.42)	4.12 (3.84 to 4.39)	4.19 (3.81 to 4.57)	4.70 (4.28 to 5.11)	5.0 (4.59 to 5.42)	5.19 (4.84 to 5.54)	4.63 (4.35 to 4.92)	.002	0.002 (−0.63 to 0.64)
Other sources	2.65 (2.26 to 3.03)	2.96 (2.46 to 3.46)	2.81 (2.39 to 3.23)	3.26 (3.01 to 3.52)	2.83 (2.46 to 3.20)	2.47 (2.10 to 2.83)	2.61 (2.17 to 3.05)	2.72 (2.17 to 3.26)	.34	0.07 (−0.60 to 0.73)
Whole grains (0-10)										
Grocery stores	1.97 (1.78 to 2.16)	2.23 (1.95 to 2.51)	2.45 (2.18 to 2.72)	2.63 (2.52 to 2.74)	3.30 (3.02 to 3.57)	3.03 (2.72 to 3.34)	3.20 (2.87 to 3.54)	3.09 (2.84 to 3.34)	<.001	1.12 (0.8 to 1.43)
Restaurants	0.15 (0.09 to 0.20)	0.17 (0.08 to 0.26)	0.12 (0.08 to 0.17)	0.19 (0.07 to 0.31)	0.31 (0.19 to 0.43)	0.36 (0.25 to 0.46)	0.28 (0.19 to 0.37)	0.36 (0.27 to 0.45)	<.001	0.21 (0.11 to 0.32)
Schools	0.54 (0.36 to 0.71)	0.72 (0.53 to 0.91)	0.81 (0.61 to 1.00)	0.88 (0.69 to 1.08)	1.60 (1.20 to 2.01)	3.96 (3.67 to 4.26)	4.50 (4.03 to 4.98)	4.43 (4.08 to 4.77)	<.001	3.89 (3.50 to 4.28)
Other sources	0.91 (0.78 to 1.05)	0.98 (0.72 to 1.23)	0.61 (0.44 to 0.78)	0.86 (0.62 to 1.09)	1.27 (0.99 to 1.54)	1.05 (0.84 to 1.27)	1.08 (0.87 to 1.30)	1.04 (0.85 to 1.24)	.01	0.13 (−0.10 to 0.36)
Fish and shellfish (0-10)										
Grocery stores	0.89 (0.74 to 1.04)	0.90 (0.58 to 1.22)	0.76 (0.55 to 0.97)	0.76 (0.57 to 0.95)	0.80 (0.60 to 0.99)	0.74 (0.59 to 0.90)	0.62 (0.43 to 0.82)	0.60 (0.47 to 0.73)	.007	−0.29 (−0.48 to −0.09)
Restaurants	0.42 (0.24 to 0.59)	0.41 (0.28 to 0.54)	0.36 (0.26 to 0.47)	0.36 (0.22 to 0.49)	0.58 (0.37 to 0.79)	0.46 (0.30 to 0.62)	0.38 (0.25 to 0.52)	0.43 (0.29 to 0.58)	.60	0.02 (−0.21 to 0.24)
Schools	0.13 (0.05 to 0.21)	0.16 (0.09 to 0.24)	0.09 (0.05 to 0.14)	0.18 (0.02 to 0.35)	0.07 (0.01 to 0.13)	0.15 (0.04 to 0.25)	0.17 (0.06 to 0.29)	0.04 (0.02 to 0.07)	.45	−0.08 (−0.17 to 0)
Other sources	0.17 (0.06 to 0.27)	0.54 (0.24 to 0.84)	0.28 (0.12 to 0.44)	0.23 (0.10 to 0.36)	0.20 (0.09 to 0.32)	0.23 (0.06 to 0.40)	0.16 (0.07 to 0.25)	0.16 (0.05 to 0.28)	.03	−0.002 (−0.15 to 0.15)
Nuts, seeds, and legumes (0-10)										
Grocery stores	3.10 (2.83 to 3.38)	3.35 (2.97 to 3.72)	3.07 (2.81 to 3.34)	3.48 (3.21 to 3.76)	3.54 (3.21 to 3.87)	3.01 (2.70 to 3.32)	3.16 (2.79 to 3.52)	3.32 (3.01 to 3.62)	.84	0.22 (−0.20 to 0.63)
Restaurants	0.48 (0.25 to 0.71)	0.62 (0.37 to 0.88)	0.45 (0.30 to 0.60)	0.62 (0.45 to 0.78)	0.60 (0.36 to 0.84)	0.75 (0.56 to 0.94)	0.62 (0.46 to 0.78)	0.61 (0.48 to 0.74)	.16	0.13 (−0.13 to 0.40)
Schools	0.97 (0.79 to 1.16)	0.95 (0.55 to 1.36)	0.69 (0.47 to 0.92)	0.69 (0.54 to 0.84)	1.03 (0.79 to 1.27)	0.83 (0.63 to 1.02)	0.85 (0.51 to 1.19)	0.96 (0.77 to 1.16)	.93	−0.009 (−0.28 to 0.26)
Other sources	1.49 (1.13 to 1.85)	1.66 (1.23 to 2.10)	1.59 (1.17 to 2.02)	1.33 (0.97 to 1.69)	1.53 (1.22 to 1.83)	1.34 (1.08 to 1.61)	1.36 (1.15 to 1.58)	1.50 (1.22 to 1.79)	.38	0.02 (−0.44 to 0.48)
Sugar-sweetened beverages (0-10)										
Grocery stores	4.16 (3.77 to 4.54)	4.98 (4.59 to 5.37)	5.24 (4.91 to 5.58)	5.61 (5.39 to 5.84)	5.52 (5.16 to 5.88)	6.36 (5.97 to 6.75)	6.80 (6.44 to 7.16)	6.62 (6.26 to 6.97)	<.001	2.46 (1.94 to 2.98)
Restaurants	5.47 (5.10 to 5.83)	6.26 (5.90 to 6.63)	6.05 (5.65 to 6.45)	6.60 (6.13 to 7.07)	6.51 (6.25 to 6.78)	6.88 (6.57 to 7.19)	6.41 (6.09 to 6.72)	7.01 (6.64 to 7.37)	<.001	1.54 (1.02 to 2.06)
Schools	8.63 (8.29 to 8.98)	8.78 (8.33 to 9.23)	9.06 (8.78 to 9.34)	9.57 (9.41 to 9.72)	9.41 (9.19 to 9.62)	9.26 (9.03 to 9.49)	9.59 (9.36 to 9.81)	9.64 (9.48 to 9.79)	<.001	1.00 (0.62 to 1.38)
Other sources	6.75 (6.50 to 7.00)	6.87 (6.41 to 7.33)	6.87 (6.42 to 7.33)	7.10 (6.87 to 7.33)	6.88 (6.42 to 7.33)	6.52 (6.18 to 6.87)	7.22 (6.77 to 7.68)	7.39 (6.90 to 7.89)	.06	0.64 (0.09 to 1.20)
Processed meat (0-10)										
Grocery stores	6.96 (6.69 to 7.23)	6.93 (6.59 to 7.27)	6.90 (6.60 to 7.20)	6.86 (6.63 to 7.08)	6.87 (6.62 to 7.12)	7.04 (6.61 to 7.46)	6.95 (6.73 to 7.18)	7.04 (6.73 to 7.35)	.58	0.08 (−0.33 to 0.49)
Restaurants	7.97 (7.78 to 8.17)	8.51 (8.37 to 8.64)	8.46 (8.17 to 8.74)	8.60 (8.32 to 8.88)	8.36 (8.08 to 8.65)	8.41 (8.05 to 8.76)	8.38 (8.01 to 8.76)	8.48 (8.21 to 8.75)	.13	0.51 (0.18 to 0.84)
Schools	8.03 (7.66 to 8.41)	7.87 (7.48 to 8.25)	7.97 (7.44 to 8.50)	8.16 (7.82 to 8.51)	7.85 (7.52 to 8.17)	8.20 (7.82 to 8.58)	7.83 (7.49 to 8.18)	8.68 (8.41 to 8.95)	.09	0.64 (0.18 to 1.11)
Other sources	9.32 (9.17 to 9.47)	8.87 (8.60 to 9.15)	9.13 (8.90 to 9.37)	8.98 (8.80 to 9.16)	8.54 (8.19 to 8.89)	8.77 (8.50 to 9.03)	8.98 (8.82 to 9.13)	8.94 (8.56 to 9.32)	.06	−0.38 (−0.79 to 0.03)
Sodium (0-10)										
Grocery stores	4.97 (4.75 to 5.20)	5.29 (5.09 to 5.49)	5.15 (4.98 to 5.32)	4.66 (4.52 to 4.81)	4.94 (4.67 to 5.20)	4.56 (4.37 to 4.76)	4.60 (4.41 to 4.78)	4.99 (4.77 to 5.21)	<.001	0.02 (−0.29 to 0.34)
Restaurants	3.51 (3.27 to 3.76)	3.70 (3.43 to 3.97)	3.56 (3.30 to 3.81)	3.07 (2.74 to 3.40)	3.36 (3.11 to 3.61)	3.08 (2.83 to 3.32)	3.21 (2.95 to 3.46)	3.25 (3.00 to 3.51)	.001	−0.26 (−0.61 to 0.09)
School	4.67 (4.40 to 4.94)	4.41 (4.07 to 4.75)	4.03 (3.83 to 4.24)	4.28 (3.94 to 4.62)	4.45 (4.13 to 4.78)	4.33 (4.10 to 4.55)	4.84 (4.55 to 5.13)	5.65 (5.50 to 5.79)	<.001	0.98 (0.67 to 1.29)
Other sources	7.20 (6.92 to 7.48)	5.98 (5.68 to 6.29)	6.01 (5.62 to 6.40)	5.76 (5.38 to 6.15)	6.05 (5.69 to 6.42)	5.97 (5.45 to 6.48)	5.80 (5.59 to 6.01)	6.08 (5.57 to 6.58)	.001	−1.10 (−1.70 to −0.55)
Saturated fat (0-10)										
Grocery stores	5.16 (4.91 to 5.41)	4.86 (4.72 to 5.00)	5.18 (4.94 to 5.42)	5.45 (5.26 to 5.64)	5.54 (5.28 to 5.81)	5.09 (4.79 to 5.39)	4.62 (4.35 to 4.89)	4.55 (4.27 to 4.83)	<.001	−0.61 (−0.99 to −0.23)
Restaurants	3.32 (3.07 to 3.56)	3.18 (2.93 to 3.43)	3.21 (2.89 to 3.53)	4.00 (3.57 to 4.44)	3.63 (3.31 to 3.95)	3.49 (3.23 to 3.75)	3.76 (3.50 to 4.02)	3.45 (3.17 to 3.73)	.02	0.13 (−0.24 to 0.50)
Schools	3.71 (3.25 to 4.17)	3.54 (3.19 to 3.89)	3.61 (3.31 to 3.91)	3.83 (3.39 to 4.27)	4.47 (3.98 to 4.97)	5.29 (5.04 to 5.54)	5.04 (4.78 to 5.31)	5.49 (5.15 to 5.83)	<.001	1.78 (1.20 to 2.35)
Other sources	6.52 (6.21 to 6.84)	5.75 (5.36 to 6.15)	6.04 (5.56 to 6.51)	5.82 (5.41 to 6.23)	5.38 (5.07 to 5.68)	6.00 (5.60 to 6.39)	5.65 (5.29 to 6.01)	5.23 (4.87 to 5.59)	<.001	−1.30 (−1.80 to −0.81)

Abbreviations: AHA, American Heart Association; NHANES, National Health and Nutrition Examination Surveys.

^a^ All meals, snacks, and beverages consumed from these sources. To account for varying serving sizes and amounts as well as facilitate interpretation of the overall nutritional quality of different sources of food compared with diet quality scores and national dietary recommendations, we adjusted all intakes to 2000 kcal/d. The AHA diet scores ranged from 0 (worse) to 10 (better) and were summed to range from 0 to 80 (with higher scores indicating healthier diets) (see eAppendix 1 in the Supplement for details of the AHA diet score). All analyses incorporate the NHANES complex sampling design and survey weights to provide nationally representative estimates. The test for *P* trend across cycles evaluates the monotonic trend across the whole period. Food sources were grouped as grocery stores (consumed food items obtained from grocery or supermarket), restaurants (restaurant fast food/pizza, restaurant with servers, or restaurant no additional information), schools (kindergarten through 12th grade school cafeteria or childcare center), worksites (cafeteria not in a kindergarten through 12th grade school, vending machine, common coffee pot, or snack tray), and other sources refer to food items obtained from elsewhere (sports, recreation, or entertainment facility, street vendor, vending truck, from someone else or gift, and others).

During this period, the estimated proportion of children consuming food with poor diet quality from schools decreased by more than half, from 55.6% (95% CI, 49.1%-62.0%) to 24.4% (95% CI, 21.2%-27.5%; *P* < .001 for trend). This proportion decreased modestly from 53.2% (95% CI, 49.3%-57.1%) to 45.1% (95% CI, 40.8%-49.4%; *P* = .006 for trend) for grocery stores and from 84.8% (95% CI, 81.8%-87.7%) to 79.6% (95% CI, 76.4%-82.3%; *P* = .003 for trend) for restaurants and increased from 40.0% (95% CI, 34.9%-45.1%) to 51.7% (95% CI, 50.1%-58.0%; *P* < .001 for trend) for other sources (eFigure 2 and eTable 7 in the Supplement). For fast-food or quick-serve vs full-service restaurants, the proportion of children consuming food with poor diet quality decreased from 86.5% (95% CI, 83.1%-90.0%) to 81.2% (95% CI, 79.1%-83.2%; *P* = .001 for trend) for fast-food or quick-serve restaurants and did not significantly change in in full-service restaurants (69.3% [95% CI, 62.9%-75.8%] in 2003-2004 and 68.2% [95% CI, 61.4%-75.0%] in 2017-2018; *P* = .48 for trend) (eTable 8 in the Supplement). The large improvements in diet quality of food from schools largely occurred after 2010, with unchanged diet quality in the preceding years.

In a sensitivity analysis that excluded fish and shellfish, proportions of children consuming food with intermediate diet quality were higher from all sources and lower for children consuming food with poor diet quality (eTable 9 in the Supplement). However, comparative differences between sources and trends over time remained similar. For example, proportions of children consuming food with poor diet quality decreased from 37.9% (95% CI, 32.9%-42.9%) to 13.3% (95% CI, 10.6%-15.9%; *P* < .001 for trend) for schools, from 42.4% (95% CI, 39.1%-45.8%) to 33.0% (95% CI, 28.9%-37.0%; *P* < .001 for trend) for grocery stores, and from 70.4% (95% CI, 66.1%-74.6%) to 62.7% (95% CI, 57.3%-68.0%; *P* = .04 for trend) for restaurants and increased from 16.8% (95% CI, 13.7%-19.9%) to 29.3% (95% CI, 24.0%-35.2%; *P* = .001 for trend) for food consumed from other sources.

Among food and nutrient components, improvements for food consumed from grocery stores were largely associated with fewer SSBs (score difference, 2.46; *P* < .001 for trend) and more whole grains (score difference, 1.12; *P* < .001 for trend) (Table 2). The large improvements at schools were associated with increased whole grains (score difference, 3.89; *P* < .001 for trend) and less saturated fat (score difference, 1.78; *P* < .001 for trend), SSBs (score difference, 1.0; *P* < .001 for trend), and sodium (score difference, 0.98; *P* < .001 for trend). The net small changes in restaurants were attributable to offsetting reductions in SSBs (score difference, 1.54; *P* < .001 for trend) vs reductions in fruits and vegetables (score difference, −0.88; *P* < .001 for trend) (Table 2). Worsening diet quality in food from other sources was mostly associated with increased sodium (score difference, −1.10; *P* = .001 for trend) and saturated fat (score difference, −1.30; *P* < .001 for trend). Changes in other HEI-2015 foods and nutrients are given in eTable 5 in the Supplement (eg, increased total fruits [2017-2018 vs 2003-2004 difference, 0.38; 95% CI, 0.03-0.72] as well as greens and beans [2017-2018 vs 2003-2004 difference, 0.15; 95% CI, −0.03 to 0.33] and decreased refined grains [2017-2018 vs 2003-2004 difference, 0.56; 95% CI, −0.16 to 1.29] and added sugar [2017-2018 vs 2003-2004 difference, 0.50; 95% CI, 0.19-0.81] at schools).

### Patterns and Trends in Diet Quality by Food Sources Among US Adults

From 2003-2004 to 2017-2018 among adults, the mean AHA score increased for grocery stores from 35.8 (95% CI, 34.6-36.9) to 38.3 (95% CI, 37.0-39.5; *P* < .001 for trend) and for restaurants from 28.5 (95% CI, 27.8-29.2) to 29.0 (95% CI, 28.2-29.7; *P* = .02 for trend), remained stable for worksites at 33.5 (95% CI, 32.3-34.7) in 2003-2004 and 33.4 (95% CI, 32.1-34.7) in 2017-2018 (*P* = .13 for trend), and decreased for other sources from 36.1 (95% CI, 35.3-37.0) to 34.7 (95% CI, 33.9-35.6; *P* = .02 for trend) (Table 3). Findings were generally similar for HEI-2015, with mean AHA scores increasing for grocery stores from 52.2 (95% CI, 51.2-53.2) to 52.8 (95% CI, 51.3-54.3; *P* = .15 for trend) and for worksites from 38.6 (95% CI, 37.4-39.8) to 39.7 (95% CI, 37.6-41.7); *P* = .02 for trend) and decreasing for restaurants from 40.3 (95% CI, 39.5-41.2) to 40.1 (95% CI, 38.2-40.2; *P* = .72 for trend) and for other sources from 42.3 (95% CI, 41.6-43.0) to 36.2 (95% CI, 35.2-37.3); *P* < .001 for trend) (eTable 10 in the Supplement). During this period, estimated proportions of adults consuming food with poor diet quality from grocery stores decreased from 40.1% (95% CI, 36.9%-43.2%) to 32.9% (95% CI, 29.0%-36.8%; *P* = .001 for trend), was stable from restaurants at 65.4% (95% CI, 61.9%-68.8%) in 2003-2004 and 65.2% (95% CI, 61.4%-68.7%; *P* = .07 for trend) in 2017-2018 and worksites at 55.6% (95% CI, 49.2%-61.9%) in 2003-2004 and 50.7% (95% CI, 43.8%-57.6%) in 2017-2018 (*P* = .25 for trend), and increased from other sources from 33.8% (95% CI, 31.0%-36.6%) to 43.8% (95% CI, 39.7%-48.0%; *P* < .001 for trend) (eFigure 2 and eTable 11 in the Supplement). Similar overall trends and differences were evident when fish and shellfish were excluded from the diet score, with the estimated proportions of adults consuming food with poor diet quality from grocery stores decreasing from 30.0% (95% CI, 27.0%-33.1%) to 23.8% (95% CI, 20.3%-27.3%; *P* = .002 for trend), remaining stable from restaurants at 51.6% (95% CI, 48.6%-54.7%) to 51.3% (95% CI, 47.2%-55.5%; *P* = .07 for trend), and increasing at worksites from 14.7% (95% CI, 9.9%-19.5%) to 17.9% (95% CI, 12.6%-23.2%; *P* = .78 for trend) and other sources from 18.5% (95% CI, 16.1%-20.8%) to 21.4% (95% CI, 18.4%-24.4%; *P* = .002 for trend) (eTable 12 in the Supplement). Evaluating fast-food or quick-serve vs full-service restaurants, the proportion of adults consuming food with poor diet quality decreased from 74.9% (95% CI, 72.2%-77.7%) to 72.9% (95% CI, 69.2%-76.7%) in the former (*P* = .001 for trend) and did not significantly change in the latter (49.2% [95% CI, 44.7%-53.8%] to 52.5% [95% CI, 47.1%-57.9%]; *P* = .96 for trend) (eTable 13 in the Supplement).

**Table 3.** Trends in Diet Quality of Foods From Different Sources Among US Adults (≥20 Years of Age), NHANES 2003-2018^a^

Diet components (scoring range)	Survey-weighted AHA mean score (95% CI)								*P* value for trend	Difference (95% CI) for 2017-2018 vs 2003-2004
	2003-2004 (n = 4448)	2005-2006 (n = 4520)	2007-2008 (n = 5420)	2009-2010 (n = 5762)	2011-2012 (n = 4801)	2013-2014 (n = 5047)	2015-2016 (n = 5017)	2017-2018 (n = 4742)		
AHA diet score (0-80)										
Grocery stores	35.8 (34.6 to 36.9)	37.6 (36.5 to 38.7)	37.7 (36.3 to 39.0)	38.4 (37.7 to 39.1)	39.5 (38.6 to 40.3)	39.1 (38.2 to 39.9)	38.5 (37.5 to 39.5)	38.3 (37.0 to 39.5)	<.001	2.51 (0.85 to 4.17)
Restaurants	28.5 (27.8 to 29.2)	28.4 (27.8 to 28.9)	28.1 (27.6 to 28.6)	28.6 (27.8 to 29.3)	29.1 (28.3 to 29.9)	29.5 (28.9 to 30.2)	29.0 (28.1 to 29.9)	29.0 (28.2 to 29.7)	.02	0.42 (−0.60 to 1.43)
Worksites	33.5 (32.3 to 34.7)	32.7 (31.9 to 33.6)	33.1 (32.3 to 33.9)	34.3 (33.5 to 35.1)	33.8 (32.2 to 35.3)	34.3 (32.8 to 35.9)	34.6 (33.4 to 35.9)	33.4 (32.1 to 34.7)	.13	−0.07 (−1.90 to 1.72)
Other sources	36.1 (35.3 to 37.0)	35.5 (34.7 to 36.3)	35.1 (34.3 to 35.9)	35.5 (34.7 to 36.3)	35.6 (35.0 to 36.1)	34.7 (34.2 to 35.3)	35.0 (34.2 to 35.7)	34.7 (33.9 to 35.6)	.02	−1.40 (−2.60 to −0.19)
Fruits and vegetables (0-10**)**										
Grocery stores	4.87 (4.57 to 5.16)	4.97 (4.73 to 5.21)	5.01 (4.73 to 5.29)	5.11 (4.99 to 5.23)	5.17 (4.94 to 5.40)	5.00 (4.77 to 5.23)	4.99 (4.72 to 5.26)	4.74 (4.50 to 4.97)	.58	−0.13 (−0.51 to 0.25)
Restaurants	4.30 (4.13 to 4.47)	4.22 (4.02 to 4.41)	3.93 (3.77 to 4.09)	3.97 (3.81 to 4.14)	3.83 (3.68 to 3.98)	3.67 (3.53 to 3.81)	3.50 (3.36 to 3.65)	3.47 (3.25 to 3.70)	<.001	−0.82 (−1.10 to −0.54)
Worksites	2.53 (2.14 to 2.92)	2.44 (1.95 to 2.93)	1.81 (1.44 to 2.19)	2.76 (2.37 to 3.14)	2.93 (2.16 to 3.70)	2.70 (2.20 to 3.20)	3.12 (2.54 to 3.70)	2.70 (2.23 to 3.17)	.03	0.17 (−0.45 to 0.78)
Other sources	3.74 (3.36 to 4.12)	4.08 (3.64 to 4.51)	3.98 (3.65 to 4.31)	4.02 (3.71 to 4.33)	3.62 (3.27 to 3.97)	3.11 (2.88 to 3.34)	3.34 (3.01 to 3.68)	3.24 (2.75 to 3.73)	<.001	−0.50 (−1.10 to 0.12)
Whole grains (0-10)										
Grocery stores	2.60 (2.40 to 2.79)	2.98 (2.80 to 3.15)	2.95 (2.72 to 3.18)	3.37 (3.22 to 3.52)	3.50 (3.34 to 3.67)	3.43 (3.28 to 3.58)	3.33 (3.13 to 3.53)	3.04 (2.78 to 3.30)	<.001	0.44 (0.11 to 0.77)
Restaurants	0.36 (0.28 to 0.44)	0.42 (0.34 to 0.51)	0.44 (0.36 to 0.52)	0.40 (0.32 to 0.48)	0.68 (0.58 to 0.78)	0.76 (0.64 to 0.88)	0.81 (0.60 to 1.02)	0.53 (0.38 to 0.67)	<.001	0.16 (0.002 to 0.33)
Worksites	0.84 (0.32 to 1.37)	0.60 (0.36 to 0.85)	0.64 (0.39 to 0.89)	0.99 (0.68 to 1.29)	1.09 (0.66 to 1.51)	1.10 (0.89 to 1.31)	0.88 (0.47 to 1.30)	0.63 (0.36 to 0.89)	.53	−0.22 (−0.80 to 0.37)
Other sources	0.67 (0.53 to 0.81)	0.77 (0.62 to 0.92)	0.78 (0.62 to 0.93)	0.76 (0.62 to 0.91)	0.95 (0.83 to 1.07)	0.82 (0.73 to 0.90)	0.98 (0.84 to 1.12)	0.81 (0.68 to 0.93)	.02	0.14 (−0.05 to 0.32)
Fish and shellfish (0-10)										
Grocery stores	1.43 (1.20 to 1.65)	1.48 (1.31 to 1.65)	1.45 (1.31 to 1.59)	1.58 (1.38 to 1.79)	1.60 (1.37 to 1.82)	1.50 (1.30 to 1.70)	1.35 (1.13 to 1.57)	1.37 (1.16 to 1.59)	.48	−0.05 (−0.36 to 0.26)
Restaurants	1.22 (1.01 to 1.44)	1.28 (1.11 to 1.44)	1.24 (1.04 to 1.44)	1.29 (1.12 to 1.47)	1.09 (0.92 to 1.25)	1.24 (1.07 to 1.42)	1.14 (0.99 to 1.29)	1.28 (1.10 to 1.47)	.76	0.06 (−0.22 to 0.34)
Worksites	0.26 (0.15 to 0.37)	0.19 (0.09 to 0.29)	0.30 (0.12 to 0.47)	0.46 (0.13 to 0.79)	0.47 (0.27 to 0.67)	0.43 (0.15 to 0.71)	0.48 (0.12 to 0.84)	0.46 (0.15 to 0.78)	.02	0.21 (−0.13 to 0.54)
Other sources	0.57 (0.36 to 0.78)	0.86 (0.64 to 1.09)	0.40 (0.30 to 0.50)	0.71 (0.52 to 0.90)	0.33 (0.23 to 0.43)	0.43 (0.29 to 0.58)	0.37 (0.29 to 0.45)	0.37 (0.24 to 0.50)	<.001	−0.20 (−0.45 to 0.05)
Nuts, seeds, and legumes (0-10)										
Grocery stores	3.93 (3.59 to 4.27)	4.12 (3.79 to 4.44)	4.04 (3.71 to 4.37)	4.23 (4.01 to 4.46)	4.43 (4.25 to 4.61)	4.42 (4.15 to 4.69)	4.55 (4.23 to 4.87)	4.52 (4.20 to 4.84)	<.001	0.59 (0.12 to 1.06)
Restaurants	1.01 (0.81 to 1.22)	1.05 (0.88 to 1.22)	1.07 (0.84 to 1.30)	1.0 (0.80 to 1.19)	1.32 (1.11 to 1.52)	1.28 (1.14 to 1.41)	1.46 (1.24 to 1.69)	1.29 (1.06 to 1.52)	<.001	0.28 (−0.03 to 0.59)
Worksites	1.30 (1.00 to 1.61)	1.42 (1.05 to 1.78)	1.21 (0.93 to 1.49)	1.42 (1.16 to 1.67)	1.67 (1.17 to 2.17)	1.21 (0.81 to 1.61)	1.25 (0.78 to 1.72)	1.60 (0.98 to 2.22)	.56	0.30 (−0.40 to 0.99)
Other sources	1.80 (1.61 to 1.98)	1.72 (1.46 to 1.99)	1.55 (1.35 to 1.75)	1.45 (1.20 to 1.70)	1.95 (1.70 to 2.21)	2.01 (1.78 to 2.23)	1.93 (1.74 to 2.12)	1.92 (1.69 to 2.15)	.007	0.12 (−0.17 to 0.42)
Sugar-sweetened beverages (0-10)										
Grocery stores	6.06 (5.70 to 6.42)	6.51 (6.21 to 6.81)	6.41 (6.07 to 6.75)	6.75 (6.59 to 6.91)	6.96 (6.65 to 7.26)	7.30 (7.00 to 7.59)	7.35 (7.10 to 7.60)	7.33 (6.93 to 7.73)	<.001	1.27 (0.73 to 1.81)
Restaurants	7.08 (6.82 to 7.35)	7.39 (7.16 to 7.62)	7.44 (7.18 to 7.70)	7.46 (7.25 to 7.66)	7.41 (7.18 to 7.64)	7.72 (7.41 to 8.02)	7.59 (7.24 to 7.94)	8.0 (7.62 to 8.38)	<.001	0.92 (0.46 to 1.38)
Worksites	6.71 (6.27 to 7.15)	6.84 (6.26 to 7.43)	6.94 (6.39 to 7.49)	7.19 (6.75 to 7.62)	6.38 (5.88 to 6.88)	6.78 (6.11 to 7.44)	7.87 (7.33 to 8.41)	7.17 (6.50 to 7.83)	.10	0.46 (−0.34 to 1.25)
Other sources	8.41 (8.17 to 8.65)	8.82 (8.57 to 9.06)	8.78 (8.64 to 8.92)	8.80 (8.65 to 8.95)	8.47 (8.33 to 8.61)	8.25 (8.07 to 8.43)	8.38 (8.13 to 8.63)	8.41 (8.18 to 8.65)	.006	0.005 (−0.33 to 0.34)
Processed meat (0-10)										
Grocery stores	6.88 (6.67 to 7.09)	6.94 (6.61 to 7.27)	7.03 (6.81 to 7.26)	6.93 (6.71 to 7.15)	7.13 (6.91 to 7.36)	7.17 (6.97 to 7.38)	7.06 (6.89 to 7.23)	7.31 (7.15 to 7.46)	.003	0.43 (0.17 to 0.68)
Restaurants	7.85 (7.61 to 8.08)	7.80 (7.60 to 8.01)	7.99 (7.84 to 8.14)	7.90 (7.65 to 8.16)	7.66 (7.31 to 8.01)	7.90 (7.72 to 8.09)	7.99 (7.79 to 8.18)	8.09 (7.90 to 8.27)	.10	0.24 (−0.06 to 0.54)
Worksites	9.13 (8.94 to 9.31)	9.10 (8.85 to 9.35)	9.48 (9.30 to 9.66)	9.33 (9.10 to 9.56)	9.02 (8.58 to 9.45)	9.24 (9.00 to 9.48)	9.07 (8.65 to 9.49)	9.04 (8.81 to 9.27)	.40	−0.09 (−0.38 to 0.20)
Other sources	8.96 (8.79 to 9.13)	8.78 (8.54 to 9.02)	8.70 (8.55 to 8.85)	9.02 (8.87 to 9.17)	9.01 (8.82 to 9.20)	8.93 (8.65 to 9.20)	9.06 (8.86 to 9.26)	8.99 (8.76 to 9.21)	.10	0.03 (−0.26 to 0.31)
Sodium (0-10)										
Grocery stores	4.48 (4.34 to 4.63)	5.15 (4.94 to 5.36)	5.09 (4.94 to 5.24)	4.55 (4.43 to 4.68)	4.66 (4.57 to 4.74)	4.65 (4.54 to 4.77)	4.65 (4.46 to 4.84)	4.98 (4.85 to 5.11)	.83	0.50 (0.30 to 0.69)
Restaurants	2.98 (2.81 to 3.15)	2.57 (2.45 to 2.68)	2.58 (2.44 to 2.71)	2.53 (2.39 to 2.67)	2.93 (2.76 to 3.09)	2.79 (2.68 to 2.91)	2.61 (2.49 to 2.73)	2.73 (2.55 to 2.90)	.62	−0.25 (−0.50 to −0.01)
Worksites	5.97 (5.56 to 6.38)	6.0 (5.50 to 6.49)	6.33 (5.94 to 6.73)	5.61 (5.16 to 6.07)	5.43 (4.78 to 6.08)	5.88 (5.43 to 6.33)	5.34 (4.74 to 5.94)	5.67 (5.08 to 6.25)	.04	−0.30 (−1.0 to 0.41)
Other sources	5.86 (5.45 to 6.26)	4.73 (4.49 to 4.96)	5.06 (4.85 to 5.26)	4.72 (4.48 to 4.96)	5.24 (4.97 to 5.51)	5.43 (5.16 to 5.69)	5.14 (4.85 to 5.42)	5.46 (5.16 to 5.75)	.44	−0.40 (−0.90 to 0.10)
Saturated fat (0-10)										
Grocery stores	5.51 (5.25 to 5.76)	5.45 (5.28 to 5.61)	5.69 (5.45 to 5.94)	5.87 (5.68 to 6.06)	6.01 (5.77 to 6.25)	5.58 (5.39 to 5.77)	5.19 (4.99 to 5.39)	4.97 (4.77 to 5.17)	<.001	−0.54 (−0.86 to −0.21)
Restaurants	3.74 (3.49 to 4.0)	3.64 (3.42 to 3.85)	3.41 (3.23 to 3.58)	4.00 (3.75 to 4.25)	4.16 (3.90 to 4.41)	4.16 (3.95 to 4.38)	3.88 (3.65 to 4.11)	3.57 (3.35 to 3.78)	.16	−0.17 (−0.51 to 0.16)
Worksites	6.72 (6.39 to 7.04)	6.12 (5.59 to 6.65)	6.38 (6.02 to 6.74)	6.56 (6.20 to 6.92)	6.78 (6.33 to 7.23)	7.01 (6.31 to 7.70)	6.63 (6.07 to 7.18)	6.13 (5.50 to 6.76)	.84	−0.59 (−1.30 to 0.12)
Other sources	6.11 (5.86 to 6.36)	5.70 (5.44 to 5.96)	5.86 (5.54 to 6.18)	6.03 (5.77 to 6.30)	6.01 (5.82 to 6.20)	5.77 (5.45 to 6.08)	5.76 (5.41 to 6.11)	5.55 (5.27 to 5.83)	.03	−0.56 (−0.93 to −0.18)

Abbreviations: AHA, American Heart Association; NHANES, National Health and Nutrition Examination Surveys.

^a^ All meals, snacks, and beverages consumed from these sources. To account for varying serving sizes and amounts as well as facilitate interpretation of the overall nutritional quality of different sources of food compared with diet quality scores and national dietary recommendations, we adjusted all intakes to 2000 kcal/d. The AHA diet scores ranged from 0 (worse) to 10 (better) and were summed to range from 0 to 80 (with higher scores indicating healthier diets) (see eAppendix 1 in the Supplement for details of the AHA diet score). All analyses incorporate the NHANES complex sampling design and survey weights to provide nationally representative estimates. The test for *P* trend across cycles evaluates the monotonic trend across the whole period. Food sources were grouped as grocery stores (consumed food items obtained from grocery or supermarket), restaurants (restaurant fast food/pizza, restaurant with servers, or restaurant no additional information), schools (kindergarten through 12th grade school cafeteria or childcare center), worksites (cafeteria not in a kindergarten through 12th grade school, vending machine, common coffee pot, or snack tray), and other sources refer to food items obtained from elsewhere (sports, recreation, or entertainment facility, street vendor, vending truck, from someone else or gift, and others).

Among food and nutrient components, improvements in diet quality at grocery stores were largely associated with decreased SSBs (score difference, 1.27; *P* < .001 for trend), processed meat (score difference, 0.43; *P* = .003 for trend), increased nuts, seeds, and legumes (score difference, 0.59; *P* < .001 for trend), and whole grains (score difference, 0.44; *P* < .001 for trend) (Table 3). Changes in other HEI-2015 foods and nutrients are given in eTable 10 in the Supplement.

### Trends by Population Subgroups

Trends in calorie sources across population subgroups are reported in eTables 14 to 21 in the Supplement. Among non-Hispanic White children, proportions of calories from grocery stores (67.0%; 95% CI, 64.5%-69.6%) and restaurants (20.7%; 95% CI, 18.7%-22.7%) remained stable but shifted away from grocery stores (from 68.6% [95% CI, 63.3%-74.0%] to 60.5% [95% CI, 57.9%-63.1%]) and toward restaurants (from 17.7% [95% CI, 16.0%-19.5%] to 22.0% [95% CI, 19.8%-24.1%]) among non-Hispanic Black children (21.6% [95% CI, 17.9%-25.4%]) and Hispanic children (69.0% [95% CI, 65.9%-72.1%] to 60.8% [95% CI, 56.8%-64.8%] from grocery stores and 16.8% [95% CI, 14.6%-19.0%] to 21.6% [95% CI, 17.9%-25.4%] from restaurants). By 2017-2018, proportions of calories from schools also differed by race/ethnicity, parental educational level, and household income (eg, 9.9% [95% CI, 7.9%-11.9%] of calories for non-Hispanic black children vs 4.7% [95% CI, 2.6%-6.8%] of calories for non-Hispanic white children).

**Figure 1.**
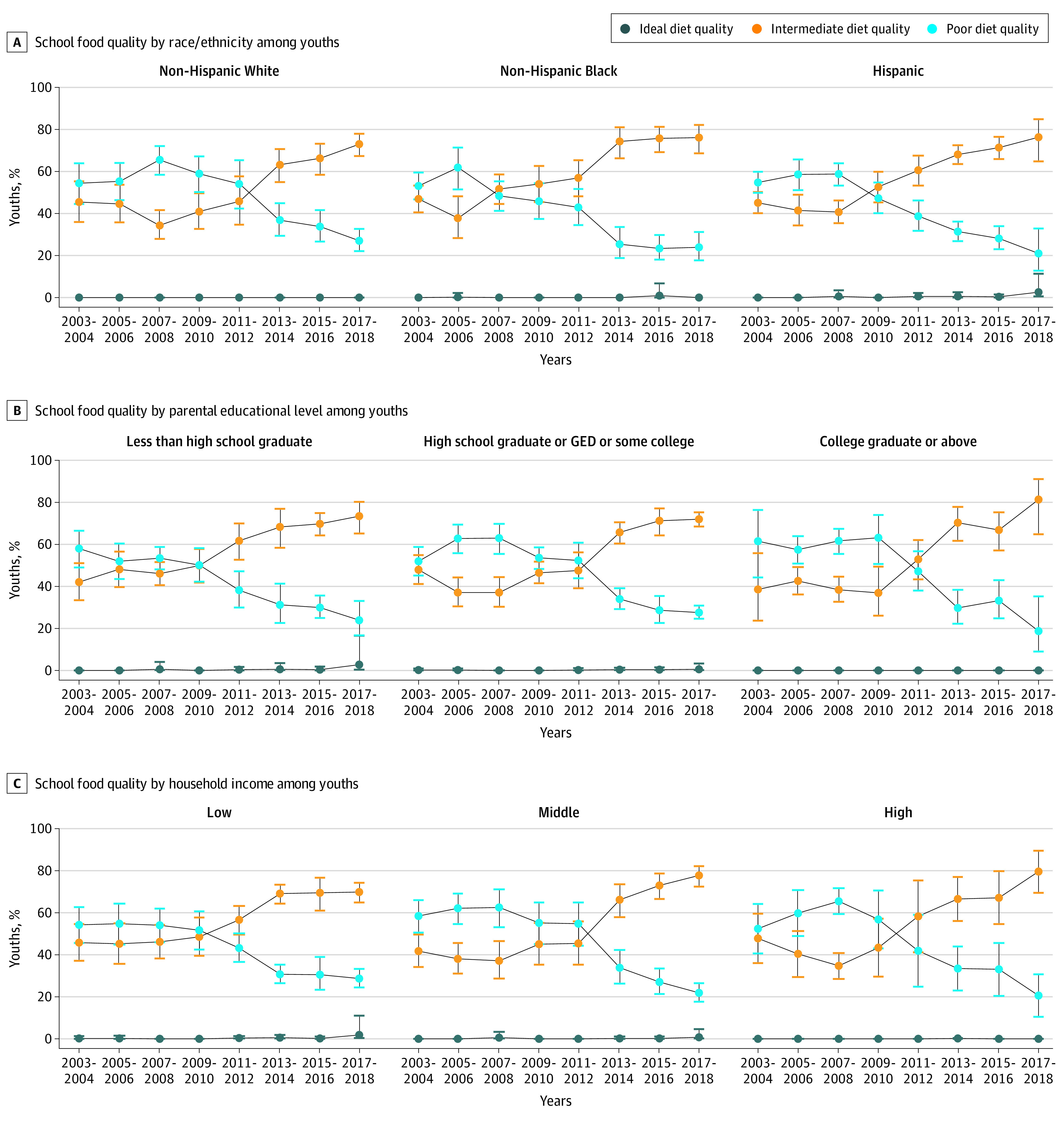
Trends in Estimated Proportions of US Children With Poor, Intermediate, or Ideal Diet Quality for Foods Consumed From Schools by Race/Ethnicity, Parental Educational Level, and Household Income, National Health and Nutrition Examination Surveys 2003-2018 The American Heart Association score is based on total fruits and vegetables; whole grains; fish and shellfish; nuts, seeds, and legumes; sugar-sweetened beverages; processed meat; sodium; and saturated fat. Data were weighted to be nationally representative. Data points indicate estimated percentages; error bars, 95% CIs. By race/ethnicity, *P* < .001 for trend and *P* = .12 for interaction for all; by parental educational level, *P* < .001 for trend and *P* = .39 for interaction for all; and by household income, *P* < .001 for trend and *P* = .18 for interaction for all (see eTable 12 in the Supplement for details). GED indicates General Educational Development.

Regarding nutritional quality, significant improvements occurred in some population subgroups but not others. Most equitable improvements occurred for food consumed from schools, which improved after 2010 in all subgroups (Figure 1 and eFigure 3 in the Supplement). For example, proportions consuming food with poor diet quality from schools decreased from 54.4% (95% CI, 44.5%-64.2%) to 27.0% (95% CI, 21.7%-32.3%) among non-Hispanic white children, from 54.9% (95% CI, 49.8%-59.9%) to 21.1% (95% CI, 11.1%-31.2%) among Hispanic children, and from 53.1% (95% CI, 46.7%-59.6%) to 23.9% (95% CI, 17.1%-30.7%) among non-Hispanic Black children. Before 2010, older children generally had worse diet quality of foods from school than younger kids, but this rapidly equalized (and improved) after 2010. These secondary analysis findings were similar for schools limited to kindergarten through 12th grade (eTable 8 in the Supplement).

**Figure 2.**
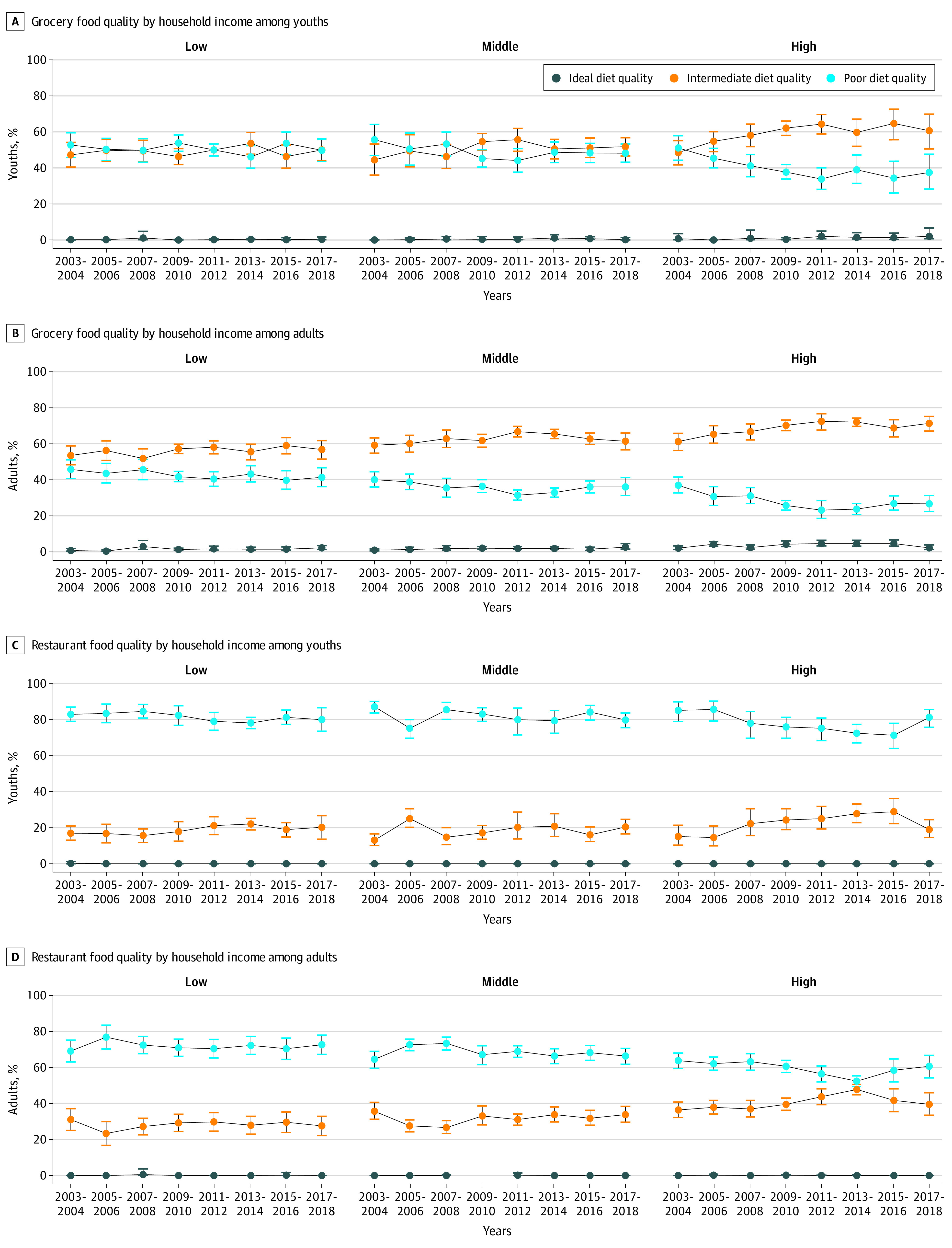
Trends in Estimated Proportions of US Children and Adults With Poor, Intermediate, or Ideal Diet Quality for Food Consumed From Grocery Stores and Restaurants by Household Income, National Health and Nutrition Examination Surveys 2003-2018 See Figure 1 for the American Heart Association score definition. Data points indicate estimated percentages; error bars, 95% CIs (see eTables 12 and 15 in the Supplement for details).

In contrast, increasing disparities were suggested by sex, race/ethnicity, educational level, and income in trends in diet quality of foods from grocery stores (Figure 2; eFigure 4, eTable 14, and eTables 16-18 in the Supplement). The proportion of children consuming food of poor diet quality from grocery stores was stable in low-income households (from 52.7% [95% CI, 45.8%-59.6%] to 49.7% [95% CI, 43.4%-55.9%]; *P* = .60 for trend) but decreased in high-income households (from 51.0% [95% CI, 44.2%-57.8%] to 37.4% [95% CI, 28.3%-47.5%]; *P* = .003 for trend).

Among adults, trends in diet quality of food from grocery stores also differed by age, race/ethnicity, and income (Figure 2; eTable 15 and eTables 19-21 in the Supplement). Proportions consuming food with poor diet quality from grocery stores improved among non-Hispanic White individuals (from 40.1% [95% CI, 36.5%-43.7%] to 33.9% [95% CI, 29.3%-38.5%]; *P* = .02 for trend), started lower and remained stable among Hispanic adults (from 32.7% [95% CI, 26.3%-39.2%] to 28.9% [95% CI, 23.8%-34.0%]; *P* = .21 for trend), and started higher and improved among non-Hispanic Black adults (from 50.6% [95% CI, 43.0%-58.2%] to 40.8% [95% CI, 33.8%-47.7%]; *P* = .01 for trend). By income, this proportion did not change significantly in low-income households (from 45.8% [95% CI, 40.6%-50.9%] to 41.3% [95% CI, 36.1%-46.5%]; *P* = .09 for trend) but decreased in high-income households (from 36.9% [95% CI, 32.7%-41.4%] to 26.5% [95% CI, 22.4%-31.1%]; *P* = .001 for trend).

Proportions of children and adults consuming restaurant foods of poor diet quality decreased only in high-income households (children: 85.0% [95% CI, 78.7%-89.7%] to 81.1% [95% CI, 75.7%-85.5%]; *P* = .004 for trend; adults: 63.7% [95% CI, 59.3%-67.9%] to 60.5% [95% CI, 54.1%-66.6%]; *P* = .03 for trend) (Figure 2).

## Discussion

On the basis of nationally representative data, this cross-sectional survey study found that patterns and trends in diet quality among US children and adults varied by food source, with additional differences among population subgroups. The largest improvements occurred in schools, overall, and in all subgroups. Nutritional quality of food from grocery stores modestly improved, with disparities by age, sex, race/ethnicity, and socioeconomic status. From each food source, children had worse overall diet quality than adults, except from schools that had the highest overall diet quality of any source. Food consumed from restaurants had the poorest overall diet quality, with little improvement. This study extends previous work on trends in overall diet quality^4,7^ and from restaurants among adults^10^ by examining trends and differences in proportions of calories and dietary quality of foods from major US sources in children and adults.

The novel findings of this study suggest that the retail grocery environment remains a top opportunity for improving diet quality, followed by restaurants, schools, and, increasingly, other settings, such as entertainment venues and food trucks. Food from grocery stores provided approximately two-thirds of calories (although it decreased in non-Hispanic Black and Hispanic children), calories from restaurants were stable at approximately 20%, and calories from several miscellaneous sources (eg, gifts from others, entertainment venues, and food trucks) increased to approximately 9%. Calories obtained from schools, including school lunch, breakfast, and snacks, were stable at approximately 9% but with variation across subgroups. (Food brought from home would be classified by its source, eg, grocery stores.) Because children are in school approximately 180 of 365 days per year and some younger (eg, 5 years of age) and older (eg, 19 years of age) youths in our sample may not be in school, approximately double these calories would typically be consumed from school foods on any given school day. With COVID-19 further shifting food purchases toward grocery sources, our results support testing and scaling new approaches to positively guide consumer choices in retail settings, including the new, powerful behavior-influencing opportunities (and dangers) inherent in online ordering.

Few calories (approximately 1% among all adults) came from worksites. Only approximately 60% of US adults are employed, many jobs have no worksite cafeteria (eg, small businesses, retail, food service, or construction), and even when cafeterias are available, employees often obtain food from restaurants or home (Blanck H, Warnock A, Wilde P, written communication, December 20, 2017). Consistent with our findings, a Centers for Disease Control and Prevention report^19^ using the US Department of Agriculture Food Acquisition and Purchasing Survey found employed US adults consumed an average of approximately 300 kcal per week from worksites in 2012-2013 (approximately 1.9% of calories based on a 2300-kcal/d diet). Our results suggest worksite nutrition promotion should focus not only on onsite procurement but also on influencing employee food choices more broadly, especially from grocery stores and restaurants (eg, through employee benefits for healthy food purchases, analogous to employer subsidies for gym memberships or exercise equipment).^20^

Another important finding was heterogeneity in levels, trends, and population disparities in diet quality according to food source. During this period among children, estimated proportions consuming food of poor diet quality from schools greatly improved (from 55.6% to 24.4%), with smaller improvements from grocery stores (53.2% to 45.1%) and restaurants (84.8% to 79.6%) and worsening from other sources (40.0% to 51.7%). Among adults, this proportion also modestly decreased for foods from grocery stores (40.1% to 32.9%) and worksites (55.6% to 50.7%), with no change from restaurants (65.4% to 65.2%) and worsening from other sources (33.8% to 43.8%). By 2017-2018, foods consumed from school by children provided the highest mean diet quality of major sources, and restaurants provided the lowest.

Across population subgroups, the consistency of improvements for food from schools was striking. Most improvement occurred after 2010, mainly attributable to increased whole grains and fruits, as well as greens and beans and decreased SSBs, refined grains, added sugar, and saturated fat. The timing and specific food changes coincide with passage of the 2010 Healthy, Hunger-Free Kids Act, which mandated new school and early childcare nutrition standards: more fruits, vegetables, and whole grains; less refined grains, sodium, and certain fats (total fat, saturated fat, and trans fat); and no SSBs.

These results, representing actual reported food consumed in a national sample, provide a crucial complement to a US Department of Agriculture analysis,^21^ which found major improvements in nutritional quality of foods offered in the National School Lunch Program and the School Breakfast Program after 2010.^21^ Of importance, these findings also document that improvements were equitable by age, sex, race/ethnicity, parental educational level, and household income, contrasting with persistent or increasing disparities in diet quality from other food sources. By 2017-2018, the mean proportion of children consuming foods with poor diet quality from schools decreased to 24.4% compared with 45.1% from grocery stores, 51.7% from other sources, and 79.6% from restaurants. Our findings suggest that, among the many national efforts aimed at improving diet quality (eg, education, dietary guidance, industry reformulation, consumer demand, and other food policies), the 2010 Healthy, Hunger-Free Kids Act produced large, specific, and equitable impacts.

This study found smaller improvements for food from grocery stores in children and adults, with significant disparities. Improvements in children were mainly attributable to increased whole grains and decreased SSBs and in improvements in adults to increased whole grains; nuts, seeds, and legumes; seafood; and plant protein and decreased SSBs, refined grains, and added sugars. Although the identified trends are promising, for 32.9% of adults and 45.1% of children (and in low-income households, 41.3% of adults and 49.7% of children), the overall food consumed from grocery stores remained of poor diet quality by 2017-2018.

Overall diet quality was lowest in restaurants. Although the US restaurant industry has made voluntary commitments toward nutrition, improvements appear small and insufficient.^10,22,23,24^ Before COVID-19, US residents were increasingly consuming meals away from home^25,26^; how trends may reset after COVID-19 remains uncertain. Amounts, venues, and diet quality of food from miscellaneous US sources, such as entertainment locations and food trucks, have not previously been reported. This study identified these trends as increasing sources of calories (by 2017-2018, nearly 10% of calories) and with worsening diet quality (the only major US food source with this trend). Our results highlight the importance of understanding determinants of diet quality of foods offered and selected by consumers from these diverse locations. Anecdotal increases in availability and popularity of food trucks, combined with low mean nutritional quality of other restaurant foods in the US, suggest this source warrants closer scrutiny.

The COVID-19 pandemic has created short-term disruptions in sources of foods for children and adults, with closures or reduced service of restaurants, schools, and childcare programs. Although data on changes in foods consumed are not yet available, sales data from grocery retailers suggest polarization of diets and potential increasing disparity in obesity risk.^27^ How trends in calories and diet quality from different food sources might evolve after COVID-19 and be positively stimulated by education, behavior change, or policy actions^28^ is ripe for further investigation.

### Limitations

Self-reported diet is subject to random and systematic error. However, interview-administered, 24-hour recalls reduce error, and random error does not bias stratum-specific means. In addition, the findings of this study for self-reported foods from schools are consistent with government reports of meal offerings. No single metric of diet quality is agreed on, and clinically relevant score differences are not established. However, this study used 2 different scores validated against disease end points. The findings represent mean nutritional quality of foods consumed on any given day overall and within each subgroup, not necessarily habitual intake of any individual, which varies from day to day. Dietary consumption represents summed effects of food availability and consumer choice. For instance, persistent poor nutritional quality of food consumed at restaurants could represent enduring consumer choices or few changes in menu offerings. Conversely, the large, equitable improvements in foods eaten from schools after 2010 demonstrate the power of food environments, rather than individual choices, for improving diet quality. The nationally representative NHANES sample does not contain details on different types of grocery stores, and heterogeneity could exist between types of grocery stores and urban vs rural or regional locations.

## Conclusions

By 2017-2018, diet quality of foods from schools increased significantly and equitably and provided the highest mean diet quality of any major food source. Modest improvements were identified in diet quality for foods consumed from grocery stores, small improvements for foods consumed from restaurants, and stable or worsening diet quality for foods consumed from other sources, all with persistent or increasing disparities.

## Notes

Supplement. **eAppendix 1.** National Health and Nutrition Examination Survey, Dietary Assessment, Dietary American Heart Association (AHA) Scores and Healthy Eating Index Scores **eAppendix 2.** Measurement of Education and Race/Ethnicity **eTable 1.** Dietary Components of the American Heart Association (AHA) 2020 Strategic Impact Goals and Scoring Standards **eTable 2.** Dietary Components of Healthy Eating Index (HEI)-2015 and Scoring Standards **eTable 3.** Trends in Estimated Percentage of Energy Intake from Grocery Stores, Restaurants, Schools, and Other Sources Among US Children Aged 5 to 19 Years and From Grocery Stores, Restaurants, Worksites and Other Sources Among Adults Aged 20 Years or Older, NHANES 2003 to 2018 **eTable 4.** Estimated Percentage of Energy Intake from Other Sources Among US Children Aged 5 to 19 Years and Adults Aged 20 Years or Older, NHANES 2017-2018 **eTable 5.** Trends in Quality of Dietary Components Consumed from Different Sources based on the Healthy Eating Index 2015 Among US Children Aged 5-19 Years Old, NHANES 2003-2018 **eTable 6.** Trends in diet quality of foods consumed from school (K-12 only) and restaurants (stratified by full service vs. fast food/quick-serve) among US children age 5-19 years, NHANES 2003-2018 **eTable 7.** Trends in Proportion of US children Aged 5-19 Years with Poor or Intermediate Diet based on American Heart Association Diet Score, by Different Food Sources and by Age, Sex, Race/Ethnicity, Education, and Income, NHANES 2003-2018 **eTable 8.** Trends in Proportion of US Children Aged 5-19 Years with Poor, Intermediate or Ideal Diet from School (K-12 only) and Restaurants (stratified by full service vs. fast food/quick-serve) based on American Heart Association Diet Score, NHANES 2003-2018 **eTable 9.** Sensitivity Analysisa on Trends in Percentage of Population with Poor or Intermediate Diet Based on American Heart Association Diet Score of 2020 Strategic Impact Goals for Meals Consumed from Different Sources by American Children Aged 5-19 Years, NHANES 2003-2018 **eTable 10.** Trends in Quality of Dietary Components Consumed from Different Sources based on the Healthy Eating Index 2015 Among US Adults Aged 20 Years and Older, NHANES 2003-2018 **eTable 11.** Trends in Proportion of US Adults Age 20+ with Poor or Intermediate Diet based on American Heart Association Diet Score, by Different Food Sources, NHANES 2003-2018 **eTable 12.** Sensitivity Analysisa on Trends in Proportion of US Adults Age 20+ Years with Poor, Intermediate or Ideal Diet based on American Heart Association Diet Score, by Different Food Sources, NHANES 2003-2018 **eTable 13.** Trends in Proportion of US Adults Aged 20+ Years with Poor, Intermediate or Ideal Diet from Restaurants based on American Heart Association Diet Score, NHANES 2003-2018 **eTable 14.** Trends in Estimated Percentage of Energy Intake from Grocery Stores, Restaurants, Schools and Other Sources Among American Children aged 5-19 years by Age, Sex, Race/Ethnicity, Parental Education, and Income, NHANES 2003-2018 **eTable 15.** Trends in Estimated Percentage of Energy Intake from Grocery Stores, Restaurants, Worksites and Other Sources Among Adults Aged 20 Years or Older by Age, Sex, Race/Ethnicity, Education and Income, NHANES 2003 to 2018 **eTable 16.** Trends in Proportions of US Children Aged 5-19 years with Poor or Intermediate Diet based on American Heart Association Diet Score, by Different Food Sources and by Age, Sex, Race/Ethnicity, Education and Income, NHANES 2003-2018 **eTable 17.** Trends in American Heart Association Diet Score for Meals Consumed from Different Sources by Age Group, Sex, Race/Ethnicity, Parental Education, and Income among American Children aged 5-19 years, NHANES 2003-2018 **eTable 18.** Trends in Healthy Eating Index-2015 for Meals Consumed from Different Sources by Age Group, Sex, Race/Ethnicity, Education, and Income among American Children aged 5-19 years, NHANES 2003-2018 **eTable 19.** Trends in Proportions of US Adults Age 20+ Years with Poor or Intermediate Diet based on American Heart Association Diet Score by Different Food Sources and by Age, Sex, Race/Ethnicity, Education, and Income, NHANES 2003-2018 **eTable 20.** Trends in American Heart Association Diet Score for Meals Consumed from Different Sources by Age, Sex, Race/Ethnicity, Education, and Income among American Adults aged 20+, NHANES 2003-2018 **eTable 21.** Trends in Healthy Eating Index-2015 for Meals Consumed from Different Sources by Age Group, Sex, Race/Ethnicity, Education, and Income among American Adults aged 20 years +, NHANES 2003-2018 **eFigure 1.** Trends in Estimated Percentage of Energy Intake from Grocery Stores, Restaurants, Schools or Worksites, and Other Sources Among US Children Aged 5-19 Years (A: top) and Adults Age 20+ Years (B: bottom), NHANES 2003-2004 to 2017-2018 **eFigure 2.** Trends in Estimated Proportions of Americans Consuming Foods from Grocery Stores (A), Restaurants (B), Schools or Worksites (C), and Other Sources (D) with Poor, Intermediate or Ideal Diet Quality Among Children Aged 5 to 19 Years (left) and Adults Aged 20 Years or Older (right), NHANES 2003-2004 to 2017-2018 **eFigure 3.** Trends in Estimated Proportions of Americans Consuming Foods from Schools with Poor, Intermediate or Ideal Diet Quality Among Children Aged 5 to 19 Years by Age (top) and Sex (bottom), NHANES 2003-2004 to 2017-2018 **eFigure 4.** Trends in Estimated Proportions of Americans Consuming Foods from Grocery with Poor, Intermediate or Ideal Diet Quality by Age for Children (top) and Adults (bottom), NHANES 2003-2004 to 2017-2018
