# Supplementary material for: Trends in Food Sources and Diet Quality Among US Children and Adults, 2003-2018
Source: JAMA Netw Open. 2021 Apr 12;4(4):e215262. doi: 10.1001/jamanetworkopen.2021.5262 (PMC8042524; doi:10.1001/jamanetworkopen.2021.5262)
Supplement: Supplement. — eAppendix 1. National Health and Nutrition Examination Survey, Dietary Assessment, Dietary American Heart Association (AHA) Scores and Healthy Eating Index Scores eAppendix 2. Measurement of Education and Race/Ethnicity eTable 1. Dietary Components of the American Heart Association (AHA) 2020 Strategic Impact Goals and Scoring Standards eTable 2. Dietary Components of Healthy Eating Index (HEI)-2015 and Scoring Standards eTable 3. Trends in Estimated Percentage of Energy Intake from Grocery Stores, Restaurants, Schools, and Other Sources Among US Children Aged 5 to 19 Years and From Grocery Stores, Restaurants, Worksites and Other Sources Among Adults Aged 20 Years or Older, NHANES 2003 to 2018 eTable 4. Estimated Percentage of Energy Intake from Other Sources Among US Children Aged 5 to 19 Years and Adults Aged 20 Years or Older, NHANES 2017-2018 eTable 5. Trends in Quality of Dietary Components Consumed from Different Sources based on the Healthy Eating Index 2015 Among US Children Aged 5-19 Years Old, NHANES 2003-2018 eTable 6. Trends in diet quality of foods consumed from school (K-12 only) and restaurants (stratified by full service vs. fast food/quick-serve) among US children age 5-19 years, NHANES 2003-2018 eTable 7. Trends in Proportion of US children Aged 5-19 Years with Poor or Intermediate Diet based on American Heart Association Diet Score, by Different Food Sources and by Age, Sex, Race/Ethnicity, Education, and Income, NHANES 2003-2018 eTable 8. Trends in Proportion of US Children Aged 5-19 Years with Poor, Intermediate or Ideal Diet from School (K-12 only) and Restaurants (stratified by full service vs. fast food/quick-serve) based on American Heart Association Diet Score, NHANES 2003-2018 eTable 9. Sensitivity Analysisa on Trends in Percentage of Population with Poor or Intermediate Diet Based on American Heart Association Diet Score of 2020 Strategic Impact Goals for Meals Consumed from Different Sources by American Children Aged 5-19 Years, NHANES [file jamanetwopen-e215262-s001.pdf]

Supplementary Online Content

Liu J, Micha R, Li Y, Mozaffarian D. Trends in food sources and diet quality among US children and adults, 2003-2018. *JAMA Netw Open*. 2021;4(4):e215262. doi:10.1001/jamanetworkopen.2021.5262

eAppendix 1. National Health and Nutrition Examination Survey, Dietary Assessment, Dietary American Heart Association (AHA) Scores and Healthy Eating Index Scores

eAppendix 2. Measurement of Education and Race/Ethnicity

eTable 1. Dietary Components of the American Heart Association (AHA) 2020 Strategic Impact Goals and Scoring Standards

eTable 2. Dietary Components of Healthy Eating Index (HEI)-2015 and Scoring Standards

eTable 3. Trends in Estimated Percentage of Energy Intake from Grocery Stores, Restaurants, Schools, and Other Sources Among US Children Aged 5 to 19 Years and From Grocery Stores, Restaurants, Worksites and Other Sources Among Adults Aged 20 Years or Older, NHANES 2003 to 2018

eTable 4. Estimated Percentage of Energy Intake from Other Sources Among US Children Aged 5 to 19 Years and Adults Aged 20 Years or Older, NHANES 2017-2018

eTable 5. Trends in Quality of Dietary Components Consumed from Different Sources based on the Healthy Eating Index 2015 Among US Children Aged 5-19 Years Old, NHANES 2003-2018

eTable 6. Trends in diet quality of foods consumed from school (K-12 only) and restaurants (stratified by full service vs. fast food/quick-serve) among US children age 5-19 years, NHANES 2003-2018

eTable 7. Trends in Proportion of US children Aged 5-19 Years with Poor or Intermediate Diet based on American Heart Association Diet Score, by Different Food Sources and by Age, Sex, Race/Ethnicity, Education, and Income, NHANES 2003-2018

eTable 8. Trends in Proportion of US Children Aged 5-19 Years with Poor, Intermediate or Ideal Diet from School (K-12 only) and Restaurants (stratified by full service vs. fast food/quick-serve) based on American Heart Association Diet Score, NHANES 2003-2018

eTable 9. Sensitivity Analysisa on Trends in Percentage of Population with Poor or Intermediate Diet Based on American Heart Association Diet Score of 2020 Strategic Impact Goals for Meals Consumed from Different Sources by American Children Aged 5-19 Years, NHANES 2003-2018

eTable 10. Trends in Quality of Dietary Components Consumed from Different Sources based on the Healthy Eating Index 2015 Among US Adults Aged 20 Years and Older, NHANES 2003-2018

© 2021 Liu J et al. *JAMA Network Open.*

eTable 11. Trends in Proportion of US Adults Age 20+ with Poor or Intermediate Diet based on American Heart Association Diet Score, by Different Food Sources, NHANES 2003-2018

eTable 12. Sensitivity Analysisa on Trends in Proportion of US Adults Age 20+ Years with Poor, Intermediate or Ideal Diet based on American Heart Association Diet Score, by Different Food Sources, NHANES 2003-2018

eTable 13. Trends in Proportion of US Adults Aged 20+ Years with Poor, Intermediate or Ideal Diet from Restaurants based on American Heart Association Diet Score, NHANES 2003-2018

eTable 14. Trends in Estimated Percentage of Energy Intake from Grocery Stores, Restaurants, Schools and Other Sources Among American Children aged 5-19 years by Age, Sex, Race/Ethnicity, Parental Education, and Income, NHANES 2003-2018

eTable 15. Trends in Estimated Percentage of Energy Intake from Grocery Stores, Restaurants, Worksites and Other Sources Among Adults Aged 20 Years or Older by Age, Sex, Race/Ethnicity, Education and Income, NHANES 2003 to 2018

eTable 16. Trends in Proportions of US Children Aged 5-19 years with Poor or Intermediate Diet based on American Heart Association Diet Score, by Different Food Sources and by Age, Sex, Race/Ethnicity, Education and Income, NHANES 2003-2018

eTable 17. Trends in American Heart Association Diet Score for Meals Consumed from Different Sources by Age Group, Sex, Race/Ethnicity, Parental Education, and Income among American Children aged 5-19 years, NHANES 2003-2018

eTable 18. Trends in Healthy Eating Index-2015 for Meals Consumed from Different Sources by Age Group, Sex, Race/Ethnicity, Education, and Income among American Children aged 5-19 years, NHANES 2003-2018

eTable 19. Trends in Proportions of US Adults Age 20+ Years with Poor or Intermediate Diet based on American Heart Association Diet Score by Different Food Sources and by Age, Sex, Race/Ethnicity, Education, and Income, NHANES 2003-2018

eTable 20. Trends in American Heart Association Diet Score for Meals Consumed from Different Sources by Age, Sex, Race/Ethnicity, Education, and Income among American Adults aged 20+, NHANES 2003-2018

eTable 21. Trends in Healthy Eating Index-2015 for Meals Consumed from Different Sources by Age Group, Sex, Race/Ethnicity, Education, and Income among American Adults aged 20 years +, NHANES 2003-2018

eFigure 1. Trends in Estimated Percentage of Energy Intake from Grocery Stores, Restaurants, Schools or Worksites, and Other Sources Among US Children Aged 5-19 Years (A: top) and Adults Age 20+ Years (B: bottom), NHANES 2003-2004 to 2017-2018

© 2021 Liu J et al. *JAMA Network Open.*

eFigure 2. Trends in Estimated Proportions of Americans Consuming Foods from Grocery Stores (A), Restaurants (B), Schools or Worksites (C), and Other Sources (D) with Poor, Intermediate or Ideal Diet Quality Among Children Aged 5 to 19 Years (left) and Adults Aged 20 Years or Older (right), NHANES 2003-2004 to 2017-2018

eFigure 3. Trends in Estimated Proportions of Americans Consuming Foods from Schools with Poor, Intermediate or Ideal Diet Quality Among Children Aged 5 to 19 Years by Age (top) and Sex (bottom), NHANES 2003-2004 to 2017-2018

eFigure 4. Trends in Estimated Proportions of Americans Consuming Foods from Grocery with Poor, Intermediate or Ideal Diet Quality by Age for Children (top) and Adults (bottom), NHANES 2003-2004 to 2017-2018

This supplementary material has been provided by the authors to give readers additional information about their work.

© 2021 Liu J et al. *JAMA Network Open.*

eAppendix 1. National Health and Nutrition Examination Survey, Dietary Assessment, Dietary American Heart Association (AHA) scores and Healthy Eating Index scores

NHANES uses a complex, multistage, probability sampling design to select a nationally representative population of civilian, non-institutionalized participants.[1,2] The National Center for Health Statistics ethics review board approved the study protocol, and all participants provided written informed consent. Because data are publicly available and de-identified, institutional review board approval was not required for the current investigation.

The US Department of Agriculture (USDA) Automated Multiple-Pass Method was used for collecting 24-hour dietary recalls using “What We Eat In America,” the dietary interview component of NHANES. The USDA Food Patterns Equivalents Database, which disaggregates mixed foods into their components parts, was used to assess changes in food groups. Nutrients were derived from cycle-specific versions of the USDA Food and Nutrient Database for Dietary Studies.

The American Heart Association (AHA) Diet Scores

To assess a summary diet score, we constructed a continuous diet score based on the AHA 2020 Strategic Impact Goal dietary targets, which have been significantly associated with cardiovascular and metabolic outcomes in multiple analyses.[4] The primary dietary targets are fruits/vegetables, whole grains, fish and shellfish, nuts/legumes/seeds, sugar-sweetened beverages, processed meat, sodium, and saturated fat (**eTable 1**). To best assess changes, we constructed a continuous score. Intake of each dietary item was scored from 0-10 (beneficial components) or 10-0 (harmful components) depending on whether consumption was encouraged or discouraged, respectively. For beneficial dietary components, individuals with zero intake received the lowest score (0). For harmful dietary components, the lowest score (0) was assigned to a higher level approximately equivalent to the 80th to 90th percentile of intake

© 2021 Liu J et al. *JAMA Network Open.*

among US adults and rounded to a practical value (eg, 4500mg/d of sodium, one 50-g servings/d of processed meat, two 8-oz servings/d of sugar-sweetened beverages, and 15% energy of saturated fat). Optimal intake (i.e., at or greater than the target AHA level for encouraged foods/nutrients; or at or less than the target AHA level for discouraged foods/nutrients) was assigned a score of 10, and intermediate intake was scored linearly between 0 and 10. The scoring ranges are provided in **eTable 1**. Details regarding the AHA score was published elsewhere.[5]

The Healthy Eating Index (HEI) 2015

The HEI is a measure of diet quality, independent of quantity, that can be used to assess compliance with the U.S. Dietary Guidelines for Americans (DGAs) and monitor changes in dietary patterns. The original HEI was released by the United States Department of Agriculture's (USDA) Center for Nutrition Policy and Promotion in 1995 and since then it has been significantly updated through a collaboration with the USDA and National Cancer Institute (NCI).[6] The HEI-2015 is the latest iteration of the index and was designed to align with key dietary recommendations from the *2015-2020 DAGs*. The HEI-2015 contains 13 components that sum to a total maximum score of 100 points (**eTable 2**). Each of the components is scored on a density basis out of 1,000 calories, with the exception of Fatty Acids, which is a ratio of unsaturated to saturated fatty acid. Currently, several methods associated with SAS macros that have been developed for use with the HEI are available to the public, and details can be found on the NCI website: https://epi.grants.cancer.gov/hei/hei-scores-for-describing-dietary-intake.html.

© 2021 Liu J et al. *JAMA Network Open.*

eAppendix 2. Measurement of Education and Race/Ethnicity

Educational attainment for children was classified according to the highest level of their parental education. Information on race/ethnicity was collected by trained NHANES interviewers according to the fixed categories provided by the National Center for Health Statistics using the Computer-Assisted Personal Interview system, with classification identified a household proxy for participants younger than 16 years and by the respondents directly for those aged 16 years and older.

© 2021 Liu J et al. *JAMA Network Open.*

eTable 1. Dietary Components of the American Heart Association (AHA) 2020 Strategic Impact Goals and Scoring Standards

Component	Points Range	Scoring Standard[a]	
		Max	Min
**AHA Score[b]**	0-80		
Fruits and vegetables[c]	0-10	≥4.5 cups equiv. per day	0
Whole grains	0-10	≥3 oz equiv. per day	0
Fish and shellfish	0-10	≥1 oz equiv. per day	0
Nuts, seeds and legumes[d]	0-10	≥4 servings per day	0
Sugar-sweetened beverages	10-0	≤ 5.14 fl oz per day	>16 fl per day
Processed meat	10-0	≤ 0.5 oz equiv. per day	>1.764 oz equiv. per day
Sodium	10-0	≤ 1500 mg per day	>4500 mg per day
Saturated fat	10-0	≤7% energy	>15% energy

[a] Intakes between the minimum and maximum standards are scored proportionately.
[b] All AHA dietary variables were energy-adjusted to 2000kcal/d prior to analysis.
[c] According to the AHA 2020 Goals, up to 3 cups/wk (0.42 cups/d) of starchy vegetables (e.g., potatoes, peas, corn) could be included; this maximum was incorporated into the analysis, with higher intake not contributing toward the score. 100% fruit juice could also be included; while its contribution was not capped in the original AHA 2020 Goals and thus not in our score, some organizations recommend no more than 1 serving/d of 100% fruit juice.
[d] A serving of nuts, seeds and legumes is 1-oz equivalent of nuts and seeds or ½ cup of legume.

© 2021 Liu J et al. *JAMA Network Open.*

eTable 2. Dietary Components of Healthy Eating Index (HEI)-2015 and Scoring Standards

Component	Points Range	Scoring Standard[a]	
		Max	Min
**HEI-2015 Adequacy**			
Total Fruits[b]	0-5	≥0.8 cup equiv. per 1,000 kcal	0
Whole Fruits[c]	0-5	≥0.4 cup equiv. per 1,000 kcal	0
Total Vegetables[d]	0-5	≥1.1 cup equiv. per 1,000 kcal	0
Greens and Beans[d]	0-5	≥0.2 cup equiv. per 1,000 kcal	0
Whole Grains	0-10	≥1.5 oz equiv. per 1,000 kcal	0
Dairy[e]	0-10	≥1.3 cup equiv. per 1,000 kcal	0
Total Protein Foods[f]	0-5	≥2.5 oz equiv. per 1,000 kcal	0
Seafood and Plant Proteins[e, g]	0-5	≥0.8 oz equiv. per 1,000 kcal	0
Fatty Acids[h]	0-10	(PUFAs + MUFAs)/SFAs ≥2.5	(PUFAs + MUFAs)/SFAs ≤1.2
**HEI-2015 Moderation**			
Refined Grains	0-10	≤1.8 oz equiv. per 1,000 kcal	≥4.3 oz equiv. per 1,000 kcal
Sodium	0-10	≤1.1 grams per 1,000 kcal	≥2.0 grams per 1,000 kcal
Added Sugars	0-10	≤6.5% of energy	≥26% of energy
Saturated Fats	0-10	≤8% of energy	≥16% of energy

[a] Intakes between the minimum and maximum standards are scored proportionately.
[b] Includes 100% fruit juice.
[c] Includes all forms except juice.
[d] Includes legumes (beans and peas)
[e] Includes all milk products, such as fluid milk, yogurt, and cheese, and fortified soy beverages.
[f] Includes legumes (beans and peas)
[g] Includes seafood, nuts, seeds, soy products (other than beverages), and legumes (beans and peas).
[h] Ratios of poly-and monosaturated fatty acids (PUFAs and MUFAs) to saturated fatty acids (SFAs).

© 2021 Liu J et al. *JAMA Network Open.*

eTable 3. Trends in Estimated Percentage of Energy Intake from Grocery Stores, Restaurants, Schools, and Other Sources Among US Children Aged 5 to 19 Years and From Grocery Stores, Restaurants, Worksites and Other Sources Among Adults Aged 20 Years or Older, NHANES 2003 to 2018.

Estimated percent of energy from each food source, %	Survey-weighted % (95% CI) [a]								*P* - trend	*2017-2018 versus 2003-2004, difference (95% CI)*
	**2003-2004**	**2005-2006**	**2007-2008**	**2009-2010**	**2011-2012**	**2013-2014**	**2015-2016**	**2017-2018**		
**Children**	**(n=3236)**	**(n=3318)**	**(n=2445)**	**(n=2596)**	**(n=2462)**	**(n=2495)**	**(n=2383)**	**(n=1970)**		
Grocery stores	67.8 (66.0-69.5)	67.6 (66.1-69.1)	66.3 (64.3-68.3)	68.5 (66.7-70.3)	63.1 (61.3-64.8)	64.5 (62.6-66.4)	63.1 (60.7-65.5)	64.6 (62.2-66.9)	<.001	-3.20 (-6.10, -0.27)
Restaurants	19.3 (17.9-20.7)	18.1 (17.0-19.2)	17.7 (16-19.4)	15.8 (14.7-17.0)	19.4 (17.1-21.7)	18.8 (17.9-19.7)	18.5 (17.0-20.1)	20.3 (18.2-22.4)	.13	1.02 (-1.50, 3.52)
Schools	7.93 (6.09-9.77)	7.53 (6.42-8.65)	9.01 (6.84-11.2)	8.90 (7.12-10.7)	9.14 (7.33-11.0)	9.16 (7.40-10.9)	9.43 (7.99-10.9)	6.88 (4.88-8.89)	.82	-1.10 (-3.80, 1.67)
Others	5.0 (4.54-5.46)	6.79 (6.09-7.49)	6.99 (5.92-8.07)	6.73 (6.17-7.29)	8.39 (6.96-9.81)	7.55 (6.61-8.50)	8.91 (7.65-10.2)	8.23 (6.51-9.96)	<.001	3.23 (1.45, 5.01)
**Adults**	**(n=4448)**	**(n=4520)**	**(n=5420)**	**(n=5762)**	**(n=4801)**	**(n=5047)**	**(n=5017)**	**(n=4,742)**		
Grocery stores	68.8 (67.6-70.1)	69.1 (67.7-70.4)	70.8 (69.1-72.6)	72.3 (71.3-73.3)	67.5 (65.6-69.4)	67.4 (66.5-68.3)	68.4 (66.9-69.9)	67.3 (65.6-68.9)	.002	-1.50 (-3.60, 0.52)
Restaurants	22.2 (21.1-23.2)	21.3 (19.7-22.8)	20.7 (19.1-22.4)	19.0 (18.2-19.7)	21.6 (20.4-22.8)	22.7 (21.3-24.0)	21.2 (19.7-22.8)	22.5 (20.8-24.2)	.75	-0.95 (-2.80, 0.93)
Worksites	2.13 (1.63-2.62)	1.98 (1.63-2.33)	1.35 (1.10-1.59)	1.54 (1.19-1.88)	1.94 (1.38-2.49)	1.19 (1.01-1.36)	1.05 (0.74-1.35)	1.33 (1.10-1.56)	<.001	-0.79 (-1.30, -0.25)
Others	6.87 (6.34-7.40)	7.70 (6.72-8.68)	7.10 (6.42-7.77)	7.20 (6.64-7.75)	8.94 (7.79-10.1)	8.77 (7.93-9.61)	9.29 (8.27-10.3)	8.88 (7.96-9.80)	<.001	2.01 (0.95, 3.08)

Abbreviations: NHANES, National Health and Nutrition Examination Survey.
Food sources were grouped as grocery stores (consumed food items obtained from “grocery or supermarket”), restaurants (“restaurant fast food/pizza, restaurant with waiter/waitress, restaurant no additional information”), schools (“K-12 school cafeteria, childcare center”), worksites (“cafeteria not in a K-12 school, vending machine, common coffee pot or snack tray) and other sources refer to food items obtained from elsewhere (“sport, recreation, or entertainment facility, street vendor, vending truck, from someone else/gift, and others).
[a] All estimates incorporate NHANES survey weights to be nationally representative.

© 2021 Liu J et al. *JAMA Network Open.*

eTable 4. Estimated Percentage of Energy Intake from Other Sources Among US Children Aged 5 to 19 Years and Adults Aged 20 Years or Older, NHANES 2017-2018.

Children		Adults	
	**Survey-weighted % (95% CI)** [a]		**Survey-weighted % (95% CI)** [a]
Bar/tavern/lounge	0.04 (0-0.08)	Bar/tavern/lounge	0.47 (0.30-0.64)
Community food program - other	0.07 (0-0.15)	Community food program - other	0.08 (0.04-0.12)
From someone else/gift	4.49 (3.45-5.53)	From someone else/gift	4.38 (3.95-4.80)
Mail order purchase	0.04 (0-0.07)	Mail order purchase	0.41 (0.24-0.58)
Grown or caught by you or someone you know[b]	0.31 (0.08-0.53)	Grown or caught by you or someone you know[b]	0.61 (0.21-1.01)
Sport, recreation, or entertainment facility	0.65 (0.40-0.89)	Sport, recreation, or entertainment facility	0.32 (0.21-0.43)
Street vendor, vending truck	0.33 (0.20-0.47)	Street vendor, vending truck	0.30 (0.16-0.45)
Child/Adult home care	0	Child/Adult care/home center	0.17 (0.01-0.34)
Soup kitchen/shelter/food pantry	0.01 (0-0.03)	Soup kitchen/shelter/food pantry	0.10 (0-0.23)
Cafeteria NOT in a K-12 school	0.60 (0-1.30)	Cafeteria in a K-12 school	1.03 (0.77-1.29)
Meals on Wheels	0.09 (0-0.27)	Meals on Wheels	0.06 (0-0.11)
Residential dining facility	0.08 (0-0.21)	Residential dining facility	0.07 (0-0.15)
Fundraiser sales	0.06 (0.03-0.10)	Fundraiser sales	0.06 (0.02-0.10)
Vending machine	0.09 (0.05-0.12)		
Common coffee pot or snack tray	0.03 (0-0.06)		
Others[c]	0	Others[c]	0.81 (0.42-1.20)

Abbreviations: NHANES, National Health and Nutrition Examination Survey.

[a] All estimates incorporate NHANES survey weights to be nationally representative.

[b] Refers to the combination of grown or caught by you or someone you know and fish caught by you or someone you know.

[c] Refers to other, specify and don’t know

© 2021 Liu J et al. *JAMA Network Open.*

eTable 5. Trends in Quality of Dietary Components Consumed from Different Sources based on the Healthy Eating Index 2015 Among US Children Aged 5-19 Years Old, NHANES 2003-2018.

HEI-2015	Survey-weighted AHA mean score (95% CI) [a]								*P*-trend	*2017-2018 versus 2003-2004, difference (95% CI)*
	2003-2004 (n=3,236)	2005-2006 (n=3,318)	2007-2008 (n=2,445)	2009-2010 (n=2,596)	2011-2012 (n=2,462)	2013-2014 (n=2,495)	2015-2016 (n=2,383)	2017-2018 (n=1,970)		
**Total Score**										
Grocery stores	47.7 (46.7-48.7)	47.1 (45.9-48.3)	47.3 (46.1-48.5)	48.3 (47.6-49.1)	50.5 (49.2-51.7)	48.3 (47.2-49.5)	47.6 (46.0-49.1)	47.5 (46.1-48.9)	.47	-0.20 (-2.0, 1.54)
Restaurants	37.1 (36.5-37.6)	36.5 (35.8-37.1)	35.6 (34.6-36.6)	37.7 (36.6-38.8)	37.5 (36.8-38.3)	35.7 (34.7-36.8)	35.5 (34.9-36.1)	35.6 (34.9-36.4)	.002	-1.40 (-2.40, -0.53)
Schools	44.1 (42.5-45.7)	42.0 (41.1-43.0)	42.3 (41.1-43.5)	42.7 (41.2-44.2)	46.2 (44.3-48.2)	51.3 (50.0-52.5)	52.8 (51.3-54.2)	51.6 (50.4-52.7)	<.001	7.50 (5.55, 9.46)
Other sources	38.9 (37.9-39.9)	37.9 (36.3-39.5)	37.4 (36.2-38.6)	37.3 (36.2-38.4)	34.2 (33.1-35.3)	34.8 (33.2-36.4)	34.9 (33-36.8)	34.7 (33.3-36.2)	<.001	-4.20 (-6.0, -2.5)
**Total vegetables**[b] (0-5)										
Grocery stores	2.11 (2.03-2.19)	1.92 (1.83-2.01)	1.94 (1.83-2.05)	2.07 (1.97-2.16)	2.04 (1.91-2.16)	2.11 (2.01-2.21)	2.11 (1.99-2.24)	1.89 (1.79-2.0)	.91	-0.22 (-0.35, -0.09)
Restaurants	3.05 (2.94-3.16)	2.80 (2.68-2.91)	2.58 (2.38-2.77)	2.59 (2.48-2.70)	2.45 (2.27-2.63)	2.31 (2.17-2.46)	2.25 (2.11-2.40)	2.16 (2.08-2.25)	<.001	-0.89 (-1.0, -0.74)
Schools	2.28 (2.10-2.46)	1.98 (1.80-2.17)	1.86 (1.71-2.02)	1.68 (1.52-1.85)	1.74 (1.60-1.88)	2.02 (1.80-2.23)	1.81 (1.65-1.97)	1.53 (1.29-1.77)	<.001	-0.75 (-1.0, -0.45)
Other sources	0.94 (0.80-1.08)	1.27 (1.10-1.45)	1.14 (0.97-1.31)	1.34 (1.11-1.58)	1.14 (0.99-1.29)	1.04 (0.87-1.21)	1.09 (0.88-1.29)	1.06 (0.78-1.34)	.58	0.11 (-0.20, 0.43)
**Greens and beans**[b]										
Grocery stores	0.80 (0.69-0.91)	0.87 (0.75-0.99)	0.79 (0.65-0.92)	0.93 (0.82-1.04)	1.03 (0.87-1.19)	1.08 (1.0-1.16)	0.96 (0.85-1.06)	0.95 (0.87-1.03)	<.001	0.15 (0.008, 0.29)
Restaurants	0.40 (0.27-0.54)	0.45 (0.32-0.58)	0.43 (0.32-0.55)	0.53 (0.43-0.64)	0.54 (0.39-0.69)	0.60 (0.46-0.73)	0.51 (0.41-0.61)	0.49 (0.37-0.60)	.10	0.08 (-0.10, 0.26)
Schools	0.30 (0.20-0.40)	0.28 (0.18-0.37)	0.28 (0.19-0.37)	0.34 (0.23-0.45)	0.53 (0.41-0.65)	0.57 (0.46-0.68)	0.62 (0.49-0.75)	0.45 (0.30-0.60)	<.001	0.15 (-0.03, 0.33)
Other sources	0.14 (0.10-0.18)	0.20 (0.13-0.27)	0.25 (0.14-0.35)	0.25 (0.14-0.35)	0.21 (0.16-0.27)	0.23 (0.14-0.32)	0.26 (0.18-0.34)	0.26 (0.14-0.38)	.06	0.12 (0, 0.25)
**Total fruits**[c]										
Grocery stores	2.49 (2.30-2.68)	2.66 (2.47-2.85)	2.47 (2.24-2.70)	2.57 (2.39-2.75)	2.64 (2.45-2.84)	2.49 (2.29-2.68)	2.28 (2.04-2.51)	2.37 (2.05-2.69)	.10	-0.12 (-0.49, 0.25)
Restaurants	0.36 (0.29-0.42)	0.31 (0.21-0.41)	0.31 (0.24-0.37)	0.36 (0.30-0.41)	0.46 (0.31-0.61)	0.30 (0.23-0.37)	0.40 (0.30-0.49)	0.38 (0.31-0.45)	.22	0.02 (-0.08, 0.12)
Schools	1.99 (1.68-2.3)	2.10 (1.87-2.32)	1.97 (1.75-2.20)	2.18 (1.90-2.46)	2.36 (2.14-2.59)	2.42 (2.18-2.67)	2.67 (2.46-2.89)	2.37 (2.22-2.52)	<.001	0.38 (0.03-0.72)

© 2021 Liu J et al. *JAMA Network Open.*

HEI-2015	Survey-weighted AHA mean score (95% CI) [a]								*P*-trend	*2017-2018 versus 2003-2004, difference (95% CI)*
	**2003-2004 (n=3,236)**	**2005-2006 (n=3,318)**	**2007-2008 (n=2,445)**	**2009-2010 (n=2,596)**	**2011-2012 (n=2,462)**	**2013-2014 (n=2,495)**	**2015-2016 (n=2,383)**	**2017-2018 (n=1,970)**		
Other sources	1.05 (0.85-1.24)	1.0 (0.77-1.24)	0.95 (0.76-1.14)	1.03 (0.89-1.18)	0.83 (0.62-1.04)	0.90 (0.71-1.1)	0.89 (0.67-1.12)	0.95 (0.67-1.23)	.32	-0.10 (-0.44, 0.25)
**Whole fruits[d]**										
Grocery stores	3.12 (2.90-3.34)	2.29 (2.13-2.46)	2.23 (2.02-2.44)	2.30 (2.11-2.49)	2.36 (2.15-2.57)	2.31 (2.09-2.54)	2.13 (1.86-2.39)	2.25 (1.95-2.55)	<.001	-0.87 (-1.20, -0.50)
Restaurants	0.53 (0.44-0.62)	0.21 (0.14-0.28)	0.25 (0.19-0.31)	0.25 (0.19-0.31)	0.33 (0.17-0.49)	0.31 (0.23-0.40)	0.33 (0.23-0.44)	0.27 (0.18-0.35)	.08	-0.26 (-0.39, -0.14)
Schools	2.36 (2.04-2.67)	1.58 (1.39-1.76)	1.52 (1.37-1.68)	1.74 (1.54-1.95)	1.94 (1.7-2.19)	2.10 (1.87-2.33)	2.11 (1.89-2.33)	1.86 (1.66-2.06)	.48	-0.50 (-0.87, -0.12)
Other sources	1.19 (0.99-1.38)	0.73 (0.59-0.86)	0.74 (0.53-0.95)	0.84 (0.68-1.0)	0.66 (0.52-0.80)	0.71 (0.54-0.89)	0.71 (0.51-0.90)	0.73 (0.49-0.97)	.01	-0.46 (-0.77, -0.15)
**Whole grains**										
Grocery stores	1.97 (1.78-2.16)	2.23 (1.95-2.51)	2.45 (2.18-2.72)	2.63 (2.52-2.74)	3.29 (3.01-3.57)	3.01 (2.70-3.32)	3.17 (2.83-3.51)	3.10 (2.86-3.35)	<.001	1.13 (0.82-1.44)
Restaurants	0.15 (0.09-0.20)	0.17 (0.08-0.26)	0.12 (0.08-0.17)	0.19 (0.07-0.31)	0.31 (0.19-0.43)	0.35 (0.25-0.45)	0.29 (0.20-0.38)	0.36 (0.27-0.45)	<.001	0.21 (0.11-0.32)
Schools	0.54 (0.36-0.71)	0.72 (0.53-0.91)	0.81 (0.61-1.0)	0.88 (0.69-1.08)	1.60 (1.20-2.01)	3.96 (3.67-4.25)	4.50 (3.98-5.03)	4.43 (4.08-4.77)	<.001	3.89 (3.50-4.28)
Other sources	0.91 (0.78-1.05)	0.98 (0.72-1.23)	0.61 (0.44-0.78)	0.86 (0.62-1.09)	1.03 (0.85-1.22)	1.01 (0.79-1.24)	1.04 (0.82-1.25)	1.03 (0.82-1.24)	.06	0.12 (-0.13, 0.37)
**Total dairy[e]**										
Grocery stores	6.49 (6.17-6.81)	6.66 (6.37-6.95)	6.30 (6.04-6.56)	6.77 (6.53-7.01)	6.39 (6.07-6.70)	6.48 (6.20-6.76)	6.01 (5.66-6.36)	5.70 (5.49-5.91)	<.001	-0.79 (-1.20, -0.41)
Restaurants	4.99 (4.67-5.31)	5.05 (4.74-5.35)	5.09 (4.64-5.53)	5.32 (4.93-5.71)	5.88 (5.53-6.23)	5.67 (5.22-6.12)	5.14 (4.84-5.44)	5.12 (4.68-5.56)	.13	0.13 (-0.42, 0.67)
Schools	7.58 (7.12-8.03)	7.69 (7.38-8)	7.95 (7.39-8.50)	8.49 (8.18-8.79)	8.13 (7.82-8.44)	7.83 (7.54-8.12)	7.85 (7.30-8.41)	7.31 (6.88-7.75)	.59	-0.26 (-0.90, 0.37)
Other sources	2.16 (1.87-2.45)	2.72 (2.42-3.02)	2.53 (2.18-2.89)	2.98 (2.57-3.38)	2.65 (2.23-3.08)	2.59 (2.11-3.06)	2.42 (2.02-2.82)	2.40 (2.01-2.78)	.89	0.23 (-0.25, 0.71)
**Total protein foods[b]**										
Grocery stores	3.39 (3.28-3.49)	3.36 (3.24-3.48)	3.50 (3.39-3.61)	3.56 (3.45-3.68)	3.51 (3.37-3.64)	3.42 (3.25-3.60)	3.41 (3.25-3.57)	3.46 (3.33-3.59)	.55	0.08 (-0.09, 0.24)
Restaurants	3.54 (3.40-3.68)	3.47 (3.31-3.62)	3.47 (3.21-3.72)	3.58 (3.44-3.71)	3.46 (3.25-3.67)	3.37 (3.20-3.53)	3.27 (3.08-3.46)	3.28 (3.10-3.46)	.005	-0.26 (-0.49, -0.03)
Schools	2.68 (2.44-2.92)	2.79 (2.62-2.96)	2.94 (2.81-3.06)	2.70 (2.54-2.86)	3.10 (2.93-3.28)	2.90 (2.74-3.06)	2.88 (2.67-3.08)	2.49 (2.28-2.69)	.79	-0.19 (-0.51, 0.12)
Other sources	1.41 (1.21-1.61)	1.91 (1.77-2.04)	1.81 (1.64-1.99)	1.75 (1.54-1.97)	1.66 (1.48-1.84)	1.63 (1.41-1.86)	1.65 (1.47-1.82)	1.77 (1.50-2.03)	.77	0.35 (0.02, 0.68)

© 2021 Liu J et al. *JAMA Network Open.*

HEI-2015	Survey-weighted AHA mean score (95% CI) [a]								*P*-trend	*2017-2018 versus 2003-2004, difference (95% CI)*
	2003-2004 (n=3,236)	2005-2006 (n=3,318)	2007-2008 (n=2,445)	2009-2010 (n=2,596)	2011-2012 (n=2,462)	2013-2014 (n=2,495)	2015-2016 (n=2,383)	2017-2018 (n=1,970)		
**Seafood and plant protein,[b,f]**										
Grocery stores	1.73 (1.59-1.86)	1.76 (1.52-2.01)	1.65 (1.52-1.78)	1.85 (1.74-1.96)	2.0 (1.86-2.15)	1.70 (1.56-1.85)	1.76 (1.57-1.94)	1.77 (1.66-1.89)	.56	0.05 (-0.13, 0.22)
Restaurants	0.55 (0.42-0.68)	0.61 (0.45-0.77)	0.52 (0.43-0.61)	0.57 (0.45-0.68)	0.78 (0.61-0.95)	0.72 (0.54-0.89)	0.59 (0.50-0.69)	0.57 (0.48-0.66)	.31	0.02 (-0.14, 0.17)
Schools	0.59 (0.48-0.70)	0.53 (0.39-0.67)	0.41 (0.32-0.50)	0.52 (0.35-0.70)	1.27 (1.03-1.51)	1.0 (0.80-1.21)	1.02 (0.81-1.24)	0.94 (0.81-1.07)	<.001	0.35 (0.18-0.51)
Other sources	0.75 (0.60-0.90)	0.97 (0.76-1.17)	0.92 (0.72-1.13)	0.72 (0.51-0.93)	0.68 (0.55-0.81)	0.74 (0.55-0.94)	0.71 (0.58-0.84)	0.79 (0.64-0.94)	.13	0.04 (-0.17, 0.25)
**(PUFAs + MUFAs) /SFAs[g]**										
Grocery stores	3.67 (3.47-3.88)	3.38 (3.15-3.61)	3.76 (3.56-3.96)	3.92 (3.65-4.18)	4.54 (4.28-4.80)	3.80 (3.52-4.07)	3.93 (3.71-4.14)	3.97 (3.75-4.19)	<.001	0.30 (-0.004, 0.6)
Restaurants	4.83 (4.60-5.07)	4.58 (4.28-4.88)	4.37 (3.95-4.79)	5.06 (4.63-5.48)	4.48 (4.17-4.79)	4.13 (3.71-4.56)	4.53 (4.25-4.80)	4.57 (4.29-4.86)	.09	-0.26 (-0.63, 0.11)
Schools	3.43 (2.99-3.86)	3.37 (3.06-3.67)	3.25 (2.85-3.65)	2.83 (2.54-3.12)	3.27 (2.79-3.75)	4.11 (3.74-4.47)	3.71 (3.28-4.14)	3.93 (3.51-4.35)	.002	0.50 (-0.10, 1.11)
Other sources	5.35 (4.94-5.75)	4.74 (4.25-5.22)	4.83 (4.43-5.24)	4.55 (4.16-4.93)	4.18 (3.86-4.5)	4.34 (3.87-4.8)	4.19 (3.71-4.67)	4.08 (3.8-4.35)	<.001	-1.30 (-1.80, -0.78)
**Sodium**										
Grocery stores	6.02 (5.74-6.30)	5.80 (5.53-6.06)	5.67 (5.46-5.87)	5.05 (4.87-5.23)	5.46 (5.09-5.83)	4.96 (4.72-5.19)	4.98 (4.73-5.23)	5.47 (5.18-5.76)	<.001	-0.55 (-0.96, -0.15)
Restaurants	3.57 (3.24-3.89)	3.76 (3.42-4.09)	3.56 (3.26-3.87)	3.10 (2.72-3.47)	3.44 (3.10-3.78)	3.06 (2.76-3.36)	3.14 (2.83-3.46)	3.25 (2.99-3.51)	.002	-0.31 (-0.74, 0.11)
Schools	4.99 (4.64-5.33)	4.57 (4.13-5)	4.19 (3.92-4.45)	4.49 (4.07-4.91)	4.67 (4.30-5.04)	4.52 (4.25-4.8)	5.21 (4.86-5.55)	6.15 (6.01-6.29)	<.001	1.16 (0.79, 1.54)
Other sources	7.53 (7.20-7.87)	6.22 (5.92-6.52)	6.27 (5.89-6.65)	5.98 (5.57-6.39)	5.77 (5.32-6.22)	5.70 (5.22-6.18)	5.68 (5.42-5.93)	6.07 (5.52-6.62)	<.001	-1.50 (-2.10, -0.82)
**Refined grains**										
Grocery stores	5.51 (5.22-5.80)	5.57 (5.39-5.76)	5.51 (5.27-5.75)	5.03 (4.85-5.21)	5.47 (5.25-5.70)	5.27 (5.10-5.43)	5.20 (4.95-5.44)	5.20 (4.92-5.47)	.01	-0.31 (-0.71, 0.09)
Restaurants	4.19 (3.92-4.45)	4.39 (4.03-4.75)	4.25 (3.89-4.61)	4.50 (4.12-4.87)	4.09 (3.70-4.48)	3.68 (3.37-3.99)	3.72 (3.43-4.0)	4.0 (3.66-4.34)	.002	-0.19 (-0.62, 0.24)
Schools	5.63 (5.03-6.23)	5.08 (4.64-5.52)	5.24 (4.96-5.52)	4.79 (4.34-5.24)	4.66 (4.36-4.96)	5.98 (5.64-6.32)	6.57 (6.17-6.97)	6.19 (5.79-6.6)	<.001	0.56 (-0.16, 1.29)
Other sources	6.86 (6.50-7.23)	6.56 (6.10-7.03)	6.73 (6.41-7.06)	6.55 (6.21-6.90)	6.09 (5.48-6.71)	6.04 (5.67-6.41)	6.21 (5.72-6.7)	5.85 (5.39-6.31)	<.001	-1.0 (-1.60, -0.43)

© 2021 Liu J et al. *JAMA Network Open.*

HEI-2015	Survey-weighted AHA mean score (95% CI) [a]								*P*-trend	*2017-2018 versus 2003-2004, difference (95% CI)*
	2003-2004 (n=3,236)	2005-2006 (n=3,318)	2007-2008 (n=2,445)	2009-2010 (n=2,596)	2011-2012 (n=2,462)	2013-2014 (n=2,495)	2015-2016 (n=2,383)	2017-2018 (n=1,970)		
**Saturated fat**										
Grocery stores	6.09 (5.84-6.34)	5.79 (5.64-5.94)	6.08 (5.84-6.32)	6.34 (6.15-6.53)	6.40 (6.14-6.67)	5.93 (5.63-6.24)	5.49 (5.22-5.76)	5.42 (5.13-5.71)	<.001	-0.67 (-1.1, -0.28)
Restaurants	4.21 (3.94-4.48)	3.96 (3.68-4.24)	3.94 (3.61-4.27)	4.71 (4.25-5.18)	4.38 (4.09-4.67)	4.14 (3.83-4.44)	4.44 (4.22-4.67)	4.10 (3.82-4.38)	.34	-0.11 (-0.50, 0.28)
Schools	4.46 (3.98-4.94)	4.35 (3.96-4.73)	4.42 (4.08-4.75)	4.65 (4.18-5.11)	5.25 (4.72-5.79)	6.06 (5.78-6.35)	5.82 (5.56-6.08)	6.18 (5.84-6.51)	<.001	1.72 (1.13-2.31)
Other sources	6.94 (6.63-7.25)	6.26 (5.87-6.64)	6.47 (6.01-6.94)	6.28 (5.89-6.68)	5.46 (5.20-5.73)	6.03 (5.63-6.44)	5.58 (5.08-6.08)	5.24 (4.95-5.52)	<.001	-1.70 (-2.10, -1.30)
**Added sugars**										
Grocery stores	4.32 (3.98-4.65)	4.80 (4.54-5.06)	4.94 (4.74-5.15)	5.32 (5.13-5.52)	5.37 (5.14-5.59)	5.79 (5.51-6.07)	6.14 (5.85-6.43)	5.95 (5.66-6.24)	<.001	1.63 (1.19, 2.08)
Restaurants	6.70 (6.35-7.05)	6.71 (6.24-7.18)	6.72 (6.43-7.01)	6.97 (6.56-7.38)	6.93 (6.65-7.21)	7.09 (6.84-7.33)	6.92 (6.63-7.21)	7.07 (6.77-7.37)	.04	0.37 (-0.09, 0.83)
Schools	7.26 (7.03-7.49)	7.03 (6.62-7.44)	7.45 (7.19-7.71)	7.44 (7.04-7.84)	7.69 (7.27-8.12)	7.80 (7.54-8.07)	7.98 (7.61-8.36)	7.76 (7.55-7.97)	<.001	0.50 (0.19-0.81)
Other sources	3.71 (3.29-4.12)	4.35 (4-4.7)	4.12 (3.67-4.57)	4.17 (3.84-4.5)	3.80 (3.46-4.15)	3.79 (3.33-4.26)	4.46 (4.09-4.83)	4.51 (4.1-4.93)	.06	0.81 (0.22-1.39)

Abbreviations: NHANES, National Health and Nutrition Examination Survey; HEI, Healthy Eating Index.
Food sources were grouped as grocery stores (consumed food items obtained from “grocery or supermarket”), restaurants (“restaurant fast food/pizza, restaurant with waiter/waitress, restaurant no additional information”), schools (“K-12 school cafeteria, childcare center”), worksites (“cafeteria not in a K-12 school, vending machine, common coffee pot or snack tray) and other sources refer to food items obtained from elsewhere (“sport, recreation, or entertainment facility, street vendor, vending truck, from someone else/gift, and others).

[a] Percentages and CIs were estimated using survey weights to be nationally representative.
[b] Includes legumes (beans and peas).
[c] Includes 100% fruit juice.
[d] Includes all forms except juice.
[e] Includes all milk products, such as fluid milk, yogurt, and cheese, and fortified soy beverages.
[f] Includes seafood, nuts, seeds, soy products (other than beverages), and legumes (beans and peas).
[g] Ratio of poly- and monounsaturated fatty acids (PUFAs and MUFAs) to saturated.

© 2021 Liu J et al. *JAMA Network Open.*

eTable 6. Trends in diet quality of foods consumed from school (K-12 only) and restaurants (stratified by full service vs. fast food/quick-serve) among US children age 5-19 years, NHANES 2003-2018. [a]

Diet Components (scoring range) (intake target)	Survey-weighted AHA mean score (95%CI)								P-trend	*2017-2018 versus 2003-2004, difference (95%CI)*
	2003-2004	2005-2006	2007-2008	2009-2010	2011-2012	2013-2014	2015-2016	2017-2018		
**AHA Diet Score (0-80)**										
School	31.1 (30.0-32.2)	30.2 (29.0-31.3)	30.2 (29.3-31.2)	31.8 (30.5-33.2)	33.4 (32.0-34.7)	37.0 (36.2-37.9)	38.3 (37.4-39.1)	39.5 (38.9-40.1)	<0.001	8.44 (7.17-9.70)
Full-service	27.6 (26.3-28.9)	28.1 (27.0-29.2)	27.9 (27.0-28.9)	28.1 (27.0-29.1)	29.6 (28.1-31.1)	28.6 (27.7-29.5)	27.4 (26.2-28.5)	28.1 (26.2-30)	0.69	0.51 (-1.80, 2.82)
Fast-food	24.6 (24.0-25.2)	25.5 (25.0-26.0)	24.7 (24.0-25.3)	26.3 (25.3-27.2)	25.6 (24.7-26.5)	25.6 (24.8-26.4)	25.6 (24.7-26.5)	26.1 (25.5-26.6)	0.003	1.47 (0.65, 2.29)
**Score Components**										
**Fruits and vegetables (0-10)**										
School	4.67 (4.11-5.23)	4.15 (3.87-4.42)	4.10 (3.82-4.38)	4.20 (3.84-4.55)	4.66 (4.24-5.08)	5.04 (4.61-5.48)	5.27 (4.91-5.63)	4.66 (4.37-4.95)	<0.001	-0.01 (-0.64, 0.62)
Full-service	3.92 (3.69-4.14)	3.42 (3.24-3.60)	3.33 (2.83-3.83)	3.18 (2.72-3.64)	3.30 (2.82-3.78)	3.19 (2.71-3.67)	2.90 (2.52-3.27)	2.92 (2.46-3.38)	<.001	-1.0 (-1.50, -0.48)
Fast-food	3.27 (3.11-3.43)	3.13 (2.92-3.35)	2.76 (2.57-2.94)	2.87 (2.66-3.09)	2.70 (2.48-2.92)	2.43 (2.23-2.62)	2.56 (2.41-2.71)	2.44 (2.33-2.56)	<.001	-0.82 (-1.0, -0.62)
**Whole grains (0-10)**										
School	0.48 (0.32-0.65)	0.63 (0.44-0.81)	0.78 (0.59-0.98)	0.89 (0.69-1.09)	1.59 (1.19-1.99)	3.99 (3.70-4.29)	4.55 (4.07-5.02)	4.46 (4.14-4.78)	<0.001	3.97 (3.61-4.34)
Full-service	0.17 (0.06-0.29)	0.34 (0.14-0.53)	0.19 (0.07-0.31)	0.33 (0.09-0.58)	0.33 (0.15-0.52)	0.29 (0.19-0.38)	0.57 (0.35-0.79)	0.64 (0.26-1.01)	0.005	0.47 (0.07, 0.86)
Fast-food	0.14 (0.06-0.22)	0.10 (0.04-0.16)	0.10 (0.04-0.16)	0.11 (0.003-0.21)	0.25 (0.15-0.36)	0.36 (0.23-0.49)	0.18 (0.10-0.25)	0.24 (0.18-0.29)	<0.001	0.09 (-0.005, 0.19)
**Fish and shellfish (0-10)**										
School	0.13 (0.05-0.22)	0.15 (0.07-0.22)	0.09 (0.04-0.13)	0.19 (0.02-0.35)	0.07 (0.01-0.13)	0.15 (0.04-0.26)	0.17 (0.06-0.29)	0.04 (0.02-0.07)	0.52	-0.09 (-0.18, 0)
Full-service	0.74 (0.30-1.18)	0.74 (0.44-1.04)	0.67 (0.40-0.94)	0.62 (0.31-0.92)	1.07 (0.58-1.56)	1.02 (0.66-1.38)	0.73 (0.47-1.0)	0.84 (0.51-1.16)	0.35	0.10 (-0.46, 0.65)
Fast-food	0.21 (0.11-0.31)	0.19 (0.12-0.27)	0.16 (0.05-0.26)	0.17 (0.07-0.27)	0.17 (0.11-0.24)	0.17 (0.08-0.27)	0.14 (0.03-0.26)	0.19 (0.08-0.29)	0.60	-0.02 (-0.17, 0.12)
**Nuts, seeds and legumes (0-10)**										
School	0.95 (0.76-1.15)	0.94 (0.52-1.37)	0.67 (0.46-0.89)	0.70 (0.54-0.86)	0.94 (0.75-1.13)	0.82 (0.63-1.02)	0.84 (0.52-1.17)	0.93 (0.74-1.12)	0.95	-0.02 (-0.29, 0.25)
Full-service	0.78 (0.46-1.09)	1.08 (0.66-1.50)	0.67 (0.36-0.98)	1.04 (0.72-1.35)	0.57 (0.34-0.79)	0.99 (0.59-1.40)	0.90 (0.54-1.26)	0.85 (0.49-1.21)	0.97	0.07 (-0.41, 0.55)
Fast-food	0.32 (0.11-0.54)	0.38 (0.21-0.54)	0.33 (0.18-0.47)	0.44 (0.23-0.65)	0.52 (0.20-0.84)	0.63 (0.39-0.87)	0.47 (0.32-0.62)	0.45 (0.28-0.61)	0.06	0.12 (-0.15, 0.40)
**Sugar-sweetened beverages (0-10)**										
School	8.67 (8.33-9.01)	8.75 (8.29-9.22)	9.08 (8.80-9.35)	9.57 (9.43-9.72)	9.47 (9.25-9.70)	9.27 (9.04-9.50)	9.71 (9.60-9.83)	9.66 (9.50-9.81)	<0.001	0.98 (0.61, 1.36)
Full-service	5.88	6.04	6.21	6.67	6.52	6.43	6.38	6.83	0.02	0.94

© 2021 Liu J et al. *JAMA Network Open.*

Diet Components (scoring range) (intake target)	Survey-weighted AHA mean score (95%CI)								*P-trend*	*2017-2018 versus 2003-2004, difference (95%CI)*
	2003-2004	2005-2006	2007-2008	2009-2010	2011-2012	2013-2014	2015-2016	2017-2018		
	(5.33-6.44)	(5.41-6.66)	(5.62-6.80)	(6.04-7.29)	(5.96-7.09)	(6.03-6.83)	(5.74-7.02)	(6.28-7.37)		(0.16, 1.72)
Fast-food	5.65 (5.17-6.13)	6.49 (6.09-6.89)	6.34 (5.78-6.90)	6.89 (6.46-7.31)	6.89 (6.57-7.21)	7.26 (6.87-7.65)	6.75 (6.42-7.09)	7.38 (7.01-7.74)	<0.001	1.72 (1.12, 2.32)
**Processed meat (0-10)**										
School	8.01 (7.63-8.39)	7.88 (7.50-8.26)	7.95 (7.43-8.48)	8.15 (7.81-8.50)	7.84 (7.51-8.18)	8.20 (7.8-8.59)	7.90 (7.52-8.28)	8.67 (8.41-8.94)	0.06	0.66 (0.20, 1.13)
Full-service	8.93 (8.60-9.25)	8.84 (8.54-9.15)	9.17 (8.80-9.55)	8.66 (8.29-9.04)	9.22 (9.01-9.44)	9.03 (8.65-9.42)	8.79 (8.40-9.17)	9.21 (8.98-9.43)	0.37	0.28 (-0.12, 0.67)
Fast-food	7.87 (7.64-8.09)	8.47 (8.30-8.65)	8.29 (7.97-8.61)	8.56 (8.25-8.88)	8.21 (7.81-8.61)	8.28 (7.97-8.58)	8.28 (7.91-8.66)	8.37 (8.06-8.68)	0.21	0.50 (0.12, 0.89)
**Sodium (0-10)**										
School	4.59 (4.34-4.83)	4.27 (3.96-4.58)	4.0 (3.80-4.19)	4.27 (3.90-4.63)	4.43 (4.11-4.76)	4.29 (4.08-4.5)	4.86 (4.54-5.18)	5.59 (5.46-5.73)	<0.001	1.01 (0.73, 1.29)
Full-service	3.02 (2.68-3.36)	3.16 (2.92-3.40)	3.10 (2.66-3.54)	3.17 (2.86-3.49)	3.61 (3.15-4.07)	2.83 (2.34-3.32)	2.85 (2.49-3.21)	2.35 (1.91-2.78)	0.01	-0.68 (-1.20, -0.12)
Fast-food	3.89 (3.62-4.15)	3.88 (3.56-4.19)	3.76 (3.44-4.07)	3.18 (2.75-3.60)	3.51 (3.12-3.89)	3.27 (3.02-3.53)	3.51 (3.26-3.76)	3.70 (3.47-3.94)	0.02	-0.18 (-0.54, 0.17)
**Saturated fat (0-10)**										
School	3.55 (3.15-3.96)	3.42 (3.12-3.73)	3.57 (3.26-3.89)	3.84 (3.39-4.30)	4.37 (3.91-4.84)	5.27 (5.0-5.53)	4.95 (4.68-5.21)	5.48 (5.15-5.81)	<0.001	1.92 (1.40-2.45)
Full-service	4.18 (3.77-4.58)	4.46 (3.95-4.98)	4.57 (4.08-5.07)	4.39 (3.88-4.90)	4.99 (4.14-5.84)	4.84 (4.38-5.30)	4.24 (3.67-4.80)	4.50 (3.96-5.04)	0.45	0.33 (-0.35, 1.0)
Fast-food	3.25 (2.96-3.54)	2.86 (2.62-3.11)	2.94 (2.63-3.24)	4.03 (3.54-4.52)	3.34 (2.95-3.74)	3.24 (2.89-3.60)	3.68 (3.33-4.03)	3.30 (3.0-3.61)	0.03	0.05 (-0.37, 0.48)

Abbreviations: NHANES, National Health and Nutrition Examination Survey; AHA, American Heart Association.
Schools refer to food/beverages consumed from “K-12 school cafeteria”; full-service restaurants refer to food/beverages consumed from “restaurant with waiter/waitress”; and fast-food restaurants refer to food/beverages consumed from “restaurant fast food/pizza”.
[a] All meals, snacks, and beverages consumed from these sources. To account for varying serving sizes and amounts as well as facilitate interpretation of the overall nutritional quality of different sources of food as compared to diet quality scores and national dietary recommendations, all intakes were adjusted to 2000 kcal/d. See eText 1 for details of the AHA diet score.
All analyses incorporate the NHANES complex sampling design and survey weights to provide nationally representative estimates.
The test for P-trend across cycles evaluates the monotonic trend across the whole period.

© 2021 Liu J et al. *JAMA Network Open.*

eTable 7. Trends in Proportion of US children Aged 5-19 Years with Poor or Intermediate Diet based on American Heart Association Diet Score, by Different Food Sources and by Age, Sex, Race/Ethnicity, Education, and Income, NHANES 2003-2018.

Diet Quality by AHA Scores	AHA Score, Survey-weighted % (95% CI) [a]								*P* - trend
	2003-2004	2005-2006	2007-2008	2009-2010	2011-2012	2013-2014	2015-2016	2017-2018	
**Poor diet quality[b]**									
Grocery stores	53.2 (49.3-57.1)	48.8 (43.0-54.6)	47.6 (42.8-52.3)	44.9 (42.4-47.5)	43.3 (39.2-47.6)	45.1 (41.4-48.8)	45.4 (40.9-49.9)	45.1 (40.8-49.4)	.006
Restaurants	84.8 (81.8-87.7)	80.6 (77.4-83.8)	82.2 (78.8-85.6)	79.5 (75.4-83.5)	77.8 (74.2-81.4)	76.9 (74.2-79.6)	78.7 (75.9-81.5)	79.6 (76.4-82.3)	.003
Schools	55.6 (49.1-62.0)	59.2 (52.6-65.7)	59.8 (55.4-64.2)	54.9 (48.5-61.3)	47.2 (39.6-54.8)	32.1 (28.2-36.1)	30.0 (26.0-33.9)	24.4 (21.2-27.5)	<.001
Others	40.0 (34.9-45.1)	45.7 (41.0-50.5)	49.0 (43.2-54.7)	48.4 (45.9-50.9)	48.4 (45.6-51.2)	50.8 (44.9-56.7)	52.3 (47.4-57.2)	51.7 (47.3-56.1)	<.001
**Intermediate[b]**									
Grocery stores	46.6 (42.8-50.4)	51.1 (45.3-56.9)	51.7 (46.9-56.5)	54.8 (52.3-57.2)	56.0 (52.0-59.9)	54.1 (50.5-57.6)	54.0 (49.5-58.4)	54.1 (50.1-58.0)	.01
Restaurants	15.2 (12.2-18.1)	19.4 (16.2-22.6)	17.8 (14.4-21.2)	20.5 (16.5-24.6)	22.2 (18.6-25.8)	23.1 (20.4-25.8)	21.3 (18.5-24.1)	20.4 (17.2-23.6)	.003
Schools	44.3 (37.9-50.8)	40.8 (34.1-47.4)	40.1 (35.7-44.5)	45.1 (38.7-51.5)	52.6 (45.1-60.2)	67.6 (63.6-71.5)	69.8 (65.9-73.8)	74.8 (71.6-77.9)	<.001
Others	59.7 (54.5-64.9)	54.1 (49.3-59.0)	51.0 (45.3-56.8)	51.6 (49.1-54.1)	51.6 (48.8-54.4)	49.2 (43.3-55.1)	47.6 (42.7-52.5)	48.2 (43.8-52.6)	<.001
**Ideal[b]**									
Grocery stores	0.20 (0.05-0.91)	0.09 (0.02-0.41)	0.73 (0.26-1.97)	0.29 (0.12-0.69)	0.66 (0.30-1.43)	0.86 (0.45-1.64)	0.64 (0.29-1.37)	0.85 (0.06, 1.64)	.02
Restaurants	0	0	0	0	0	0	0	0	
Schools	0.07 (0-0.21)	0.07 (0-0.2)	0.13 (0-0.38)	0	0.17 (0-0.40)	0.29 (0-0.62)	0.23 (0-0.53)	0.86 (0-2.13)	.05
Others	0.27 (0-0.79)	0.12 (0-0.36)	0	0	0	0	0.08 (0-0.24)	0.09 (0, 0.21)	.44

Abbreviations: NHANES, National Health and Nutrition Examination Survey.

Food sources were grouped as grocery stores (consumed food items obtained from "grocery or supermarket"), restaurants ("restaurant fast food/pizza, restaurant with waiter/waitress, restaurant no additional information"), schools ("K-12 school cafeteria, childcare center"), worksites ("cafeteria not in a K-12 school, vending machine, common coffee pot or snack tray) and other sources refer to food items obtained from elsewhere ("sport, recreation, or entertainment facility, street vendor, vending truck, from someone else/gift, and others).

[a] Percentages and CIs were estimated using survey weights to be nationally representative.

[b] Poor die quality was defined as being less than 40% adherent (<32 points). Intermediate was defined as adherence of 40%-79.9% (32-63.9 points). Ideal was defined as 80% adherence or greater (≥64 points).

© 2021 Liu J et al. *JAMA Network Open.*

eTable 8. Trends in Proportion of US Children Aged 5-19 Years with Poor, Intermediate or Ideal Diet from School (K-12 only) and Restaurants (stratified by full service vs. fast food/quick-serve) based on American Heart Association Diet Score, NHANES 2003-2018. [a]

Diet Quality by AHA Scores	Survey-weighted AHA mean score (95%CI)								*P*-trend
	2003-2004	2005-2006	2007-2008	2009-2010	2011-2012	2013-2014	2015-2016	2017-2018	
**Full-Service Restaurant**									
Poor[b]	69.3 (62.9-75.8)	71.1 (64.6-77.6)	70.4 (62.2-78.5)	70.8 (65.9-75.7)	65.7 (57.7-73.7)	64.9 (60.0-69.8)	70.8 (63.9-77.6)	68.2 (61.4-75.0)	0.48
Intermediate [b]	30.5 (24.0-36.9)	28.9 (22.4-35.4)	29.6 (21.5-37.8)	29.2 (24.3-34.1)	34.3 (26.3-42.3)	35.1 (30.2-40.0)	29.3 (22.4-36.1)	31.8 (25.0-38.6)	0.46
Ideal [b]	0.18 (0-0.54)	0	0	0	0	0	0	0	
**Fast-Food Restaurant**									
Poor[a]	86.5 (83.1-90.0)	82.8 (79.7-86.0)	86.4 (84.4-88.5)	80.1 (75.4-84.9)	80.3 (76.0-84.7)	80.4 (77.4-83.4)	80.8 (77.6-84.0)	81.2 (79.1-83.2)	0.001
Intermediate [b]	13.5 (10.0-16.9)	17.2 (14.0-20.3)	13.6 (11.5-15.6)	19.9 (15.1-24.7)	19.7 (15.3-24.0)	19.6 (16.6-22.6)	19.2 (16.0-22.4)	18.8 (16.8-20.9)	0.001
Ideal [b]	0	0	0	0	0	0	0	0	
**School**									
Poor[a]	56.5 (50.4-62.6)	60.8 (54.6-67.1)	60.8 (56.6-65.1)	55.1 (48.8-61.3)	47.9 (40.5-55.3)	32.2 (28.3-36.1)	29.0 (25.2-32.9)	24.6 (21.1-28.0)	<0.001
Intermediate [b]	43.4 (37.3-49.6)	39.1 (32.8-45.4)	39.0 (34.8-43.2)	44.9 (38.7-51.2)	51.9 (44.5-59.3)	67.4 (63.5-71.4)	70.7 (66.9-74.6)	74.6 (71.1-78.0)	<0.001
Ideal [b]	0.08 (0-0.22)	0.07 (0-0.21)	0.13 (0-0.39)	0	0.17 (0-0.41)	0.34 (0-0.70)	0.24 (0-0.56)	0.87 (0-2.17)	0.05

Abbreviations: NHANES, National Health and Nutrition Examination Survey.
full-service restaurants refer to food/beverages consumed from “restaurant with waiter/waitress”; and fast-food restaurants refer to food/beverages consumed from “restaurant fast food/pizza”.

[a] Percentages and CIs were estimated using survey weights to be nationally representative.

[b] Poor die quality was defined as being less than 40% adherent (<32 points). Intermediate was defined as adherence of 40%-79.9% (32-63.9 points). Ideal was defined as 80% adherence or greater (≥64 points).

© 2021 Liu J et al. *JAMA Network Open.*

eTable 9. Sensitivity Analysis[a] on Trends in Percentage of Population with Poor or Intermediate Diet Based on American Heart Association Diet Score of 2020 Strategic Impact Goals for Meals Consumed from Different Sources by American Children Aged 5-19 Years, NHANES 2003-2018.

Diet Quality by AHA Scores	Survey-weighted % (95% CI)[b]								*P* - trend
	2003-2004	2005-2006	2007-2008	2009-2010	2011-2012	2013-2014	2015-2016	2017-2018	
**Poor diet quality[c] (<32)**									
Grocery stores	42.4 (39.1-45.8)	38.2 (33.1-43.6)	37.2 (33.1-41.5)	33.4 (31.9-34.9)	32.0 (28.9-35.3)	32.3 (28.4-36.6)	34.2 (30.6-37.9)	33.0 (28.9-37.0)	<.001
Restaurants	70.4 (66.1-74.6)	62.6 (59.9-65.2)	66.3 (62.4-70.2)	58.9 (53.5-64.2)	62.1 (57.2-67.1)	63.1 (61.2-64.9)	63.2 (59.9-66.5)	62.7 (57.3-68.0)	.04
Schools	37.9 (32.9-42.9)	38.8 (33.0-44.6)	39.2 (35.3-43.2)	33.8 (27.8-39.7)	28.8 (21.9-35.8)	18.5 (14.7-22.3)	19.5 (16.2-22.7)	13.3 (10.6-15.9)	<.001
Other sources	16.8 (13.7-19.9)	26.3 (23.4-29.2)	23.8 (20.7-26.9)	27.0 (24.5-29.6)	28.7 (25.4-32.1)	27.3 (21.8-32.7)	25.8 (22.0-29.6)	29.3 (24.0-35.2)	.001
**Intermediate[c] (32-63.9)**									
Grocery stores	57.0 (53.5-60.3)	60.1 (55.1-64.9)	61.0 (57.0-64.9)	64.5 (62.9-66.2)	64.6 (62.1-67.1)	65.1 (61.4-68.6)	63.1 (59.8-66.3)	57.0 (53.6-60.4)	.01
Restaurants	29.5 (25.3-33.8)	37.3 (34.7-40)	33.6 (29.7-37.6)	41.1 (35.7-46.5)	37.8 (32.9-42.8)	36.9 (35.1-38.8)	36.6 (33.3-39.9)	36.9 (31.5-42.3)	.05
Schools	61.7 (56.6-66.7)	60.5 (54.6-66.5)	60.3 (56.3-64.4)	65.7 (59.9-71.5)	69.5 (62.5-76.5)	79.6 (75.7-83.6)	78.4 (75-81.8)	81.3 (78.0-84.7)	<.001
Other sources	82.3 (79.3-85.3)	72.3 (69.1-75.5)	75.7 (72.7-78.6)	72.2 (69.2-75.3)	69.9 (66.6-73.2)	72.4 (67.2-77.7)	73.5 (69.7-77.3)	69.5 (63.5-74.9)	.001
**Ideal[c] (≥64)**									
Grocery stores	0.63 (0.26-1.51)	1.72 (1.03-2.86)	1.79 (1.16-2.75)	2.08 (1.44-3.0)	3.36 (2.16-5.17)	2.59 (1.91-3.50)	2.75 (2.01-3.76)	3.33 (2.22-4.43)	<.001
Restaurants	0.10 (0.03-0.41)	0.10 (0.02-0.67)	0.04 (0.0-0.31)	0.02 (0.0, 0.12)	0.03 (0.0, 0.12)	0	0.22 (0.06-0.83)	0.45 (0.14-0.77)	.07
Schools	0.45 (0.06-0.83)	0.67 (0.19-1.15)	0.43 (0-0.87)	0.49 (0.06-0.93)	1.65 (0.26-3.04)	1.93 (0.84-3.02)	2.15 (1.35-2.94)	5.40 (3.64-7.16)	<.001
Other sources	0.89 (0-1.96)	1.42 (0-2.83)	0.54 (0.05-1.03)	0.76 (0-1.62)	1.34 (0.43-2.25)	0.31 (0-0.63)	0.68 (0.11-1.24)	1.17 (0.59-2.33)	.79

Abbreviations: NHANES, National Health and Nutrition Examination Survey.

Food sources were grouped as grocery stores (consumed food items obtained from “grocery or supermarket”), restaurants (“restaurant fast food/pizza, restaurant with waiter/waitress, restaurant no additional information”), schools (“K-12 school cafeteria, childcare center”), worksites (“cafeteria not in a K-12 school, vending machine, common coffee pot or snack tray) and other sources refer to food items obtained from elsewhere (“sport, recreation, or entertainment facility, street vendor, vending truck, from someone else/gift, and others).

[a] Sensitivity analysis refers to diet quality based on the AHA score excluding fish/shellfish with a total score multiplied by 80/70.

[b] Percentages and CIs were estimated using survey weights to be nationally representative.

[c] Poor die quality was defined as being less than 40% adherent (<32 points). Intermediate was defined as adherence of 40%-79.9% (32-63.9 points). Ideal was defined as 80% adherence or greater (≥64 points).

© 2021 Liu J et al. *JAMA Network Open.*

eTable 10. Trends in Quality of Dietary Components Consumed from Different Sources based on the Healthy Eating Index 2015 Among US Adults Aged 20 Years and Older, NHANES 2003-2018.

HEI-2015	Survey-weighted AHA mean score (95% CI) [a]								*P*-trend	*2015-2016 versus 2003-2004, difference (95% CI)*
	**2003-2004 (n=4,448)**	**2005-2006 (n=4,520)**	**2007-2008 (n=5,420)**	**2009-2010 (n=5,762)**	**2011-2012 (n=4,801)**	**2013-2014 (n=5,047)**	**2015-2016 (n=5,017)**	**2017-2018 (n=4,742)**		
**HEI-2015 Total Score**										
Grocery	52.2 (51.2-53.2)	52.8 (51.9-53.8)	53.4 (51.8-54.9)	54.5 (53.9-55.2)	55.1 (54.3-55.9)	54.5 (53.6-55.5)	53.9 (52.6-55.1)	52.8 (51.3-54.3)	.15	0.56 (-1.20, 2.33)
Restaurants	40.3 (39.5-41.2)	39.8 (39.2-40.4)	39.3 (38.7-39.8)	39.9 (39.1-40.6)	41.2 (40.4-41.9)	40.7 (40.2-41.3)	40.1 (39.2-40.9)	39.2 (38.2-40.2)	.72	-1.20 (-2.50, 0.12)
Worksites	38.6 (37.4-39.8)	37.9 (36.6-39.2)	37.2 (35.8-38.6)	39.9 (38.6-41.2)	40.5 (38.6-42.4)	39.7 (38.2-41.1)	39.8 (38.3-41.2)	39.7 (37.6-41.7)	.02	1.06 (-1.30, 3.45)
Other sources	42.3 (41.6-43.0)	41.2 (40.3-42)	41.0 (40.1-41.8)	41.0 (40.3-41.7)	38.1 (37.3-38.9)	37.1 (36.2-38)	37.3 (36.2-38.4)	36.2 (35.2-37.3)	<.001	-6.0 (-7.3, -4.8)
**Total vegetables[b]** (0-5)										
Grocery	2.77 (2.70-2.83)	2.73 (2.61-2.85)	2.82 (2.72-2.93)	2.82 (2.76-2.89)	2.86 (2.79-2.93)	2.73 (2.63-2.82)	2.77 (2.67-2.87)	2.74 (2.62-2.86)	.68	-0.03 (-0.17, 0.11)
Restaurants	3.45 (3.34-3.55)	3.37 (3.25-3.49)	3.22 (3.12-3.32)	3.16 (3.09-3.23)	3.10 (2.99-3.22)	3.03 (2.92-3.13)	3.01 (2.93-3.09)	2.92 (2.76-3.07)	<.001	-0.53 (-0.72, -0.34)
Worksites	1.40 (1.14-1.67)	1.30 (1.03-1.57)	1.05 (0.85-1.25)	1.47 (1.27-1.68)	1.64 (1.26-2.01)	1.42 (1.22-1.63)	1.53 (1.20-1.86)	1.67 (1.40-1.94)	.03	0.27 (-0.11, 0.64)
Other sources	1.89 (1.71-2.06)	2.08 (1.89-2.27)	1.89 (1.70-2.09)	1.93 (1.79-2.08)	1.75 (1.58-1.93)	1.51 (1.35-1.68)	1.65 (1.45-1.86)	1.55 (1.27-1.84)	<.001	-0.34 (-0.67, -0.004)
**Greens and beans[b]**										
Grocery	1.38 (1.28-1.47)	1.36 (1.22-1.49)	1.45 (1.30-1.61)	1.57 (1.49-1.65)	1.59 (1.50-1.68)	1.65 (1.54-1.76)	1.65 (1.51-1.79)	1.58 (1.44-1.72)	<.001	0.21 (0.04, 0.38)
Restaurants	1.01 (0.84-1.18)	0.98 (0.87-1.10)	0.97 (0.85-1.10)	0.95 (0.81-1.09)	1.19 (1.05-1.32)	1.12 (1.0-1.23)	1.21 (1.06-1.37)	1.09 (0.99-1.19)	.01	0.08 (-0.12, 0.27)
Worksites	0.37 (0.19-0.56)	0.47 (0.33-0.61)	0.40 (0.24-0.56)	0.55 (0.33-0.78)	0.60 (0.41-0.79)	0.46 (0.26-0.67)	0.40 (0.14-0.65)	0.69 (0.48-0.90)	.08	0.32 (0.04, 0.59)
Other sources	0.48 (0.42-0.55)	0.78 (0.68-0.89)	0.54 (0.45-0.63)	0.56 (0.46-0.65)	0.60 (0.51-0.69)	0.46 (0.38-0.55)	0.56 (0.45-0.67)	0.53 (0.45-0.60)	.07	0.04 (-0.06, 0.14)
**Total fruits[c]**										
Grocery	2.54 (2.33-2.75)	2.58 (2.46-2.71)	2.54 (2.36-2.72)	2.67 (2.60-2.75)	2.61 (2.51-2.72)	2.50 (2.38-2.62)	2.40 (2.24-2.57)	2.27 (2.10-2.43)	.007	-0.28 (-0.54, -0.008)
Restaurants	0.42	0.41	0.36	0.37	0.43	0.38	0.36	0.29	.007	-0.13

© 2021 Liu J et al. *JAMA Network Open.*

	(0.36-0.48)	(0.34-0.47)	(0.31-0.41)	(0.30-0.44)	(0.39-0.47)	(0.36-0.40)	(0.31-0.40)	(0.23-0.36)		(-0.21, -0.04)
Worksites	0.68 (0.52-0.85)	0.55 (0.36-0.74)	0.40 (0.27-0.53)	0.66 (0.54-0.77)	0.80 (0.47-1.14)	0.50 (0.29-0.70)	0.74 (0.52-0.96)	0.48 (0.32-0.64)	.91	-0.20 (-0.43, 0.03)
Other sources	1.04 (0.94-1.14)	1.08 (0.94-1.21)	1.04 (0.83-1.25)	1.12 (1.02-1.22)	1.0 (0.87-1.12)	0.92 (0.82-1.02)	0.84 (0.71-0.97)	0.87 (0.75-0.99)	<.001	-0.17 (-0.32, -0.01)
**Whole fruits[d]**										
Grocery	3.08 (2.87-3.3)	2.44 (2.32-2.57)	2.51 (2.33-2.69)	2.62 (2.54-2.7)	2.58 (2.45-2.72)	2.56 (2.42-2.69)	2.48 (2.30-2.66)	2.38 (2.17-2.59)	<.001	-0.70 (-1.0, -0.40)
Restaurants	0.61 (0.53-0.69)	0.32 (0.26-0.38)	0.33 (0.28-0.39)	0.33 (0.26-0.40)	0.34 (0.28-0.40)	0.36 (0.32-0.39)	0.32 (0.27-0.37)	0.28 (0.21-0.35)	<.001	-0.33 (-0.43, -0.22)
Worksites	0.83 (0.63-1.03)	0.35 (0.21-0.49)	0.36 (0.23-0.49)	0.57 (0.45-0.69)	0.57 (0.33-0.82)	0.49 (0.29-0.69)	0.54 (0.35-0.72)	0.36 (0.22-0.49)	.05	-0.48 (-0.71, -0.24)
Other sources	1.24 (1.14-1.34)	0.96 (0.84-1.08)	0.97 (0.75-1.19)	0.97 (0.84-1.09)	0.88 (0.76-1.01)	0.84 (0.74-0.94)	0.82 (0.69-0.95)	0.84 (0.74-0.95)	<.001	-0.39 (-0.54, -0.24)
**Whole grains**										
Grocery	2.60 (2.40-2.79)	2.98 (2.80-3.15)	2.95 (2.72-3.18)	3.37 (3.22-3.52)	3.48 (3.32-3.65)	3.41 (3.26-3.56)	3.25 (3.07-3.43)	3.02 (2.76-3.28)	<.001	0.43 (0.10-0.75)
Restaurants	0.36 (0.28-0.44)	0.42 (0.34-0.51)	0.44 (0.36-0.52)	0.40 (0.32-0.48)	0.68 (0.58-0.78)	0.76 (0.64-0.89)	0.80 (0.59-1.01)	0.53 (0.38-0.67)	<.001	0.16 (0.002, 0.33)
Worksites	0.85 (0.33-1.38)	0.61 (0.36-0.87)	0.65 (0.41-0.90)	1.0 (0.68-1.31)	1.09 (0.67-1.51)	1.11 (0.89-1.32)	0.94 (0.51-1.36)	0.63 (0.36-0.89)	.51	-0.23 (-0.81, 0.36)
Other sources	0.66 (0.53-0.80)	0.76 (0.61-0.91)	0.78 (0.62-0.93)	0.75 (0.60-0.91)	0.92 (0.82-1.03)	0.79 (0.69-0.89)	0.93 (0.77-1.09)	0.78 (0.65-0.91)	.06	0.11 (-0.07, 0.30)
**Total dairy[e]**										
Grocery	5.17 (4.96-5.38)	5.49 (5.32-5.67)	5.34 (5.08-5.6)	5.73 (5.63-5.83)	5.23 (4.99-5.47)	5.24 (5.07-5.42)	5.09 (4.83-5.35)	4.73 (4.55-4.9)	<.001	-0.44 (-0.72, -0.17)
Restaurants	3.97 (3.75-4.19)	4.09 (3.88-4.31)	4.19 (4.0-4.39)	4.43 (4.21-4.64)	4.66 (4.45-4.87)	4.63 (4.47-4.78)	4.30 (4.07-4.52)	4.30 (4.07-4.52)	.001	0.33 (0.01-0.64)
Worksites	1.88 (1.66-2.11)	2.42 (2.0-2.84)	2.14 (1.90-2.38)	2.67 (2.25-3.09)	2.36 (1.82-2.9)	2.18 (1.74-2.63)	1.87 (1.29-2.45)	2.12 (1.54-2.70)	.97	0.24 (-0.39, 0.87)
Other sources	1.79 (1.60-1.99)	2.28 (2.12-2.45)	2.20 (2.01-2.38)	2.31 (2.03-2.59)	2.02 (1.80-2.25)	2.31 (2.14-2.49)	2.11 (1.83-2.39)	1.93 (1.68-2.18)	.86	0.14 (-0.18, 0.45)
**Total protein foods[b]**										
Grocery	3.84 (3.75-3.92)	3.88 (3.79-3.98)	3.94 (3.87-4.02)	3.99 (3.91-4.06)	3.93 (3.85-4.01)	4.01 (3.98-4.05)	4.06 (3.97-4.15)	4.02 (3.94-4.10)	<.001	0.18 (0.06-0.30)
Restaurants	4.04 (3.90-4.18)	4.14 (4.02-4.27)	4.20 (4.10-4.29)	4.10 (3.96-4.25)	4.04 (3.91-4.17)	4.0 (3.91-4.08)	3.98 (3.87-4.1)	3.95 (3.81-4.09)	.02	-0.09 (-0.29, 0.11)
Worksites	1.69 (1.50-1.89)	1.78 (1.48-2.08)	1.34 (1.12-1.55)	1.82 (1.58-2.07)	2.12 (1.70-2.53)	1.69 (1.50-1.88)	1.70 (1.40-2.0)	2.21 (1.90-2.51)	.01	0.52 (0.16-0.88)
Other sources	2.34	2.46	2.28	2.32	2.15	2.13	2.01	2.05	<.001	-0.30

© 2021 Liu J et al. *JAMA Network Open.*

	(2.14-2.55)	(2.27-2.64)	(2.15-2.40)	(2.11-2.54)	(1.99-2.30)	(1.94-2.32)	(1.87-2.15)	(1.92-2.17)		(-0.53, -0.06)
**Seafood and plant protein[b,f]**										
Grocery	2.31 (2.16-2.45)	2.33 (2.18-2.48)	2.39 (2.24-2.53)	2.52 (2.43-2.62)	2.54 (2.44-2.63)	2.57 (2.46-2.68)	2.59 (2.45-2.73)	2.56 (2.4-2.72)	<.001	0.26 (0.04, 0.47)
Restaurants	1.28 (1.14-1.43)	1.27 (1.12-1.42)	1.26 (1.10-1.42)	1.26 (1.10-1.43)	1.34 (1.21-1.46)	1.39 (1.29-1.49)	1.42 (1.28-1.56)	1.37 (1.21-1.52)	.07	0.08 (-0.13, 0.29)
Worksites	0.77 (0.65-0.89)	0.79 (0.58-1.0)	0.75 (0.57-0.93)	1.01 (0.85-1.18)	1.20 (0.91-1.49)	0.81 (0.58-1.04)	0.87 (0.58-1.15)	1.06 (0.72-1.41)	.04	0.30 (-0.07, 0.66)
Other sources	1.16 (1.03-1.29)	1.24 (1.14-1.34)	1.0 (0.89-1.11)	1.10 (0.95-1.25)	1.07 (0.98-1.16)	1.15 (1.02-1.27)	1.06 (0.97-1.15)	1.03 (0.90-1.16)	.08	-0.13 (-0.32, 0.05)
**(PUFAs + MUFAs) /SFAs[g]**										
Grocery	4.53 (4.42-4.65)	4.28 (4.09-4.46)	4.64 (4.4-4.88)	4.67 (4.52-4.82)	5.21 (5.02-5.39)	4.95 (4.8-5.1)	4.68 (4.52-4.85)	4.79 (4.57-5.01)	<.001	0.25 (0.004, 0.50)
Restaurants	5.51 (5.36-5.67)	5.45 (5.23-5.67)	5.25 (5.07-5.42)	5.42 (5.18-5.67)	5.37 (5.18-5.57)	5.23 (5.05-5.4)	5.23 (5.04-5.43)	5.17 (4.96-5.38)	.005	-0.34 (-0.60, -0.08)
Worksites	5.01 (4.43-5.59)	4.58 (4.27-4.90)	4.80 (4.29-5.32)	4.88 (4.37-5.39)	5.20 (4.75-5.64)	4.93 (4.44-5.41)	4.85 (4.33-5.37)	5.05 (4.56-5.55)	.49	0.04 (-0.72, 0.81)
Other sources	5.87 (5.60-6.14)	5.32 (5.07-5.56)	5.47 (5.15-5.80)	5.43 (5.25-5.60)	4.80 (4.49-5.11)	4.42 (4.18-4.65)	4.59 (4.27-4.9)	4.40 (4.23-4.57)	<.001	-1.50 (-1.80, -1.10)
**Moderation components**										
**Sodium**										
Grocery	5.54 (5.36-5.72)	5.61 (5.36-5.86)	5.54 (5.38-5.7)	4.87 (4.72-5.03)	5.05 (4.92-5.17)	5.04 (4.90-5.18)	5.08 (4.86-5.3)	5.48 (5.32-5.63)	<.001	-0.06 (-0.30, 0.17)
Restaurants	2.98 (2.77-3.19)	2.44 (2.30-2.57)	2.40 (2.25-2.56)	2.44 (2.28-2.59)	2.89 (2.70-3.08)	2.71 (2.58-2.85)	2.48 (2.33-2.63)	2.58 (2.37-2.79)	.31	-0.40 (-0.70, -0.10)
Worksites	6.05 (5.62-6.47)	6.14 (5.59-6.7)	6.50 (6.06-6.94)	5.77 (5.29-6.25)	5.46 (4.75-6.17)	5.97 (5.40-6.54)	5.44 (4.77-6.11)	5.77 (5.07-6.47)	.06	-0.28 (-1.1, 0.54)
Other sources	6.36 (6.02-6.69)	4.78 (4.51-5.06)	5.20 (5.0-5.40)	4.74 (4.45-5.02)	4.72 (4.41-5.02)	4.84 (4.57-5.12)	4.46 (4.12-4.80)	4.73 (4.37-5.10)	<.001	-1.60 (-2.10, -1.10)
**Refined grains**										
Grocery	6.29 (6.12-6.47)	6.74 (6.56-6.91)	6.62 (6.44-6.8)	6.60 (6.44-6.76)	6.66 (6.47-6.86)	6.86 (6.71-7.01)	6.97 (6.78-7.16)	6.78 (6.64-6.92)	<.001	0.49 (0.27, 0.71)
Restaurants	4.49 (4.18-4.80)	4.51 (4.31-4.72)	4.50 (4.32-4.69)	4.48 (4.28-4.67)	4.76 (4.51-5.02)	4.53 (4.37-4.69)	4.62 (4.35-4.89)	4.42 (4.09-4.75)	.90	-0.07 (-0.53, 0.38)
Worksites	7.25 (6.79-7.71)	7.55 (7.15-7.94)	7.68 (7.40-7.96)	7.44 (6.90-7.98)	7.26 (6.74-7.79)	7.62 (7.27-7.96)	7.84 (7.29-8.39)	7.52 (6.98-8.06)	.37	0.27 (-0.44, 0.98)
Other sources	6.90	7.05	7.05	6.93	6.55	6.58	6.82	6.49	<.001	-0.40

© 2021 Liu J et al. *JAMA Network Open.*

	(6.65-7.14)	(6.77-7.34)	(6.82-7.28)	(6.67-7.18)	(6.38-6.73)	(6.35-6.82)	(6.58-7.06)	(6.24-6.75)		(-0.76, -0.05)
**Saturated fat**										
Grocery	6.33 (6.09-6.57)	6.23 (6.07-6.39)	6.54 (6.32-6.75)	6.70 (6.50-6.90)	6.83 (6.60-7.07)	6.42 (6.24-6.59)	6.07 (5.87-6.27)	5.81 (5.61-6.01)	<.001	-0.52 (-0.84, -0.21)
Restaurants	4.51 (4.25-4.77)	4.40 (4.18-4.62)	4.15 (3.97-4.32)	4.73 (4.47-4.99)	4.88 (4.63-5.13)	4.86 (4.65-5.06)	4.57 (4.32-4.81)	4.25 (4.01-4.49)	.54	-0.26 (-0.62, 0.10)
Worksites	7.05 (6.72-7.38)	6.53 (6.02-7.05)	6.73 (6.39-7.08)	6.93 (6.60-7.27)	7.20 (6.79-7.61)	7.33 (6.66-8.0)	6.96 (6.44-7.48)	6.51 (5.93-7.08)	.85	-0.54 (-1.20, 0.12)
Other sources	6.60 (6.34-6.86)	6.13 (5.86-6.40)	6.28 (5.99-6.57)	6.43 (6.21-6.64)	5.89 (5.65-6.13)	5.46 (5.12-5.79)	5.42 (5.08-5.76)	5.18 (4.91-5.44)	<.001	-1.40 (-1.80, -1.0)
**Added sugars**										
Grocery	5.84 (5.59-6.09)	6.16 (5.92-6.4)	6.10 (5.76-6.45)	6.39 (6.24-6.54)	6.54 (6.33-6.74)	6.58 (6.38-6.78)	6.80 (6.58-7.02)	6.63 (6.37-6.88)	<.001	0.79 (0.43, 1.14)
Restaurants	7.71 (7.52-7.91)	7.98 (7.80-8.16)	7.97 (7.76-8.18)	7.82 (7.65-7.99)	7.49 (7.31-7.67)	7.73 (7.56-7.9)	7.76 (7.60-7.93)	8.04 (7.85-8.23)	.76	0.33 (0.05, 0.60)
Worksites	4.77 (4.29-5.25)	4.80 (4.24-5.35)	4.40 (3.92-4.87)	5.11 (4.69-5.54)	5.02 (4.51-5.53)	5.14 (4.64-5.65)	6.10 (5.37-6.84)	5.60 (5.1-6.11)	<.001	0.83 (0.13, 1.53)
Other sources	5.96 (5.71-6.21)	6.24 (5.94-6.55)	6.29 (5.97-6.62)	6.40 (6.09-6.70)	5.75 (5.44-6.06)	5.65 (5.33-5.97)	6.01 (5.77-6.26)	5.85 (5.49-6.22)	.03	-0.11 (-0.55, 0.34)

Abbreviations: NHANES, National Health and Nutrition Examination Survey; HEI, Healthy Eating Index.

Food sources were grouped as grocery stores (consumed food items obtained from “grocery or supermarket”), restaurants (“restaurant fast food/pizza, restaurant with waiter/waitress, restaurant no additional information”), schools (“K-12 school cafeteria, childcare center”), worksites (“cafeteria not in a K-12 school, vending machine, common coffee pot or snack tray) and other sources refer to food items obtained from elsewhere (“sport, recreation, or entertainment facility, street vendor, vending truck, from someone else/gift, and others).

[a] Percentages and CIs were estimated using survey weights to be nationally representative.

[b] Includes legumes (beans and peas).

[c] Includes 100% fruit juice.

[d] Includes all forms except juice.

[e] Includes all milk products, such as fluid milk, yogurt, and cheese, and fortified soy beverages.

[f] Includes seafood, nuts, seeds, soy products (other than beverages), and legumes (beans and peas).

[g] Ratio of poly- and monounsaturated fatty acids (PUFAs and MUFAs) to saturated.

© 2021 Liu J et al. *JAMA Network Open.*

eTable 11. Trends in Proportion of US Adults Age 20+ with Poor or Intermediate Diet based on American Heart Association Diet Score, by Different Food Sources, NHANES 2003-2018.

Diet Quality by AHA Scores	Survey-weighted % (95% CI) [a]								*P* - trend
	2003-2004	2005-2006	2007-2008	2009-2010	2011-2012	2013-2014	2015-2016	2017-2018	
**Poor diet quality[b] (<32)**									
Grocery stores	40.1 (36.9-43.2)	35.9 (31.8-39.9)	35.1 (30.7-39.5)	32.7 (30.9-34.5)	30.3 (28.0-32.5)	31.4 (29.2-33.6)	32.6 (29.3-35.9)	32.9 (29.0-36.8)	.001
Restaurants	65.4 (61.9-68.8)	67.6 (65.1-70.0)	68.1 (65.5-70.7)	64.7 (61.3-68.2)	63.3 (60.3-66.3)	61.3 (59.2-63.4)	63.7 (59.4-68)	65.2 (61.4-68.7)	.07
Worksites	55.6 (49.2-61.9)	56.7 (51.1-62.4)	56.9 (51.1-62.7)	52.7 (48.8-56.7)	57.4 (48.5-66.2)	57.8 (51.8-63.9)	50.4 (42.2-58.7)	50.7 (43.8-57.6)	.25
Others	33.8 (31.0-36.6)	39.8 (36.4-43.1)	40.0 (36.7-43.3)	38.3 (35.3-41.3)	38.7 (36.1-41.3)	42.7 (40.4-45.1)	41.9 (38.6-45.1)	43.8 (39.7-48.0)	<.001
**Intermediate[b] (32-63.9)**									
Grocery	58.6 (55.5-61.8)	61.8 (57.9-65.7)	62.7 (58.5-66.9)	64.6 (62.9-66.3)	66.8 (64.9-68.7)	65.7 (63.7-67.8)	64.6 (61.4-67.8)	64.8 (61.2-68.5)	.01
Restaurants	34.6 (31.2-38.1)	32.4 (29.9-34.8)	31.8 (29.2-34.4)	35.2 (31.8-38.7)	36.6 (33.6-39.6)	38.7 (36.6-40.8)	36.3 (31.9-40.6)	34.8 (31.3-38.6)	.07
Worksites	44.3 (38.0-50.6)	43.3 (37.6-48.9)	42.7 (37.2-48.3)	47.3 (43.3-51.2)	42.6 (33.8-51.5)	41.7 (35.7-47.8)	49.6 (41.3-57.8)	49.3 (42.4-56.2)	.24
Others	66.0 (63.2-68.9)	60.0 (56.7-63.4)	59.8 (56.5-63.1)	61.6 (58.6-64.6)	61.1 (58.5-63.7)	57.1 (54.7-59.5)	57.9 (54.6-61.2)	55.7 (51.6-59.8)	<.001
**Ideal[b] (≥64)**									
Grocery	1.29 (0.88-1.7)	2.35 (1.68-3.03)	2.21 (1.35-3.07)	2.68 (1.89-3.46)	2.90 (2.07-3.74)	2.88 (2.09-3.66)	2.81 (1.99-3.63)	2.25 (1.53-2.98)	<.001
Restaurants	0	0	0	0	0	0	0	0	
Worksites	0	0	0	0	0	0	0	0	
Others	0.18 (0.02-1.27)	0.21 (0.06	0.21 (0.04-1.16)	0.07 (0.02-0.25)	0.26 (0.04-1.56)	0.15 (0.05-0.50)	0.19 (0.07-0.50)	0.46 (0.17-1.21)	.37

Abbreviations: NHANES, National Health and Nutrition Examination Survey.
Food sources were grouped as grocery stores (consumed food items obtained from "grocery or supermarket"), restaurants ("restaurant fast food/pizza, restaurant with waiter/waitress, restaurant no additional information"), schools ("K-12 school cafeteria, childcare center"), worksites ("cafeteria not in a K-12 school, vending machine, common coffee pot or snack tray) and other sources refer to food items obtained from elsewhere ("sport, recreation, or entertainment facility, street vendor, vending truck, from someone else/gift, and others).
[a] Percentages and CIs were estimated using survey weights to be nationally representative.
[b] Poor die quality was defined as being less than 40% adherent (<32 points). Intermediate was defined as adherence of 40%-79.9% (32-63.9 points). Ideal was defined as 80% adherence or greater (≥64 points).

© 2021 Liu J et al. *JAMA Network Open.*

eTable 12. Sensitivity Analysis[a] on Trends in Proportion of US Adults Age 20+ Years with Poor, Intermediate or Ideal Diet based on American Heart Association Diet Score, by Different Food Sources, NHANES 2003-2018.

Diet Quality by AHA Scores	Survey-weighted % (95% CI) [b]								*P* - trend
	2003-2004	2005-2006	2007-2008	2009-2010	2011-2012	2013-2014	2015-2016	2017-2018	
**Poor diet quality[c] (<32)**									
Grocery stores	30.0 (27.0-33.1)	28.7 (25.7-31.7)	27.8 (24.0-31.6)	25.0 (23.5-26.6)	21.3 (19.5-23.1)	22.4 (20.9-23.9)	24.9 (22.0-27.7)	23.8 (20.3-27.3)	.002
Restaurants	51.6 (48.6-54.7)	53.5 (50.9-56.2)	56.1 (54.1-58.2)	51.8 (48.6-55.0)	50.0 (46.5-53.4)	48.6 (45.9-51.2)	50.6 (46.6-54.6)	51.3 (47.2-55.5)	.07
Worksites	14.7 (9.90-19.5)	18.8 (15.0-22.7)	16.2 (13.2-19.3)	12.9 (10.3-15.4)	18.3 (11.2-25.3)	16.9 (12.5-21.2)	14.5 (9.40-19.6)	17.9 (12.6, 23.2)	.78
Others	18.5 (16.1-20.8)	22.5 (20.3-24.6)	23.9 (21.0-26.8)	20.6 (17.8-23.3)	19.6 (16.9-22.3)	22.2 (19.4-25.0)	20.2 (17.4-22.9)	21.4 (18.4-24.4)	.002
**Intermediate[c] (32-63.9)**									
Grocery stores	65.9 (63.2-68.6)	66.5 (63.7-69.3)	67.6 (64.5-70.7)	68.0 (66.5-69.5)	71.0 (69.6-72.4)	70.1 (68.6-71.7)	68.4 (65.9-70.9)	70.0 (67.2-72.9)	.004
Restaurants	48.3 (45.3-51.4)	46.3 (43.6-49.0)	43.8 (41.8-45.8)	48.2 (44.9-51.4)	49.8 (46.3-53.2)	51.2 (48.7-53.7)	49.0 (44.9-53.0)	48.2 (44.3-52.1)	.11
Worksites	85.2 (80.4-89.9)	80.7 (76.7-84.7)	83.2 (80.1-86.3)	85.4 (82.6-88.3)	80.9 (73.5-88.3)	80.7 (76.5-84.9)	84.4 (79.0-89.8)	81.9 (76.5-87.3)	.56
Others	80.3 (78.4-82.1)	76.9 (74.7-79.1)	74.7(72-77.5)	78.5 (75.5-81.5)	79.5 (76.9-82.1)	76.6 (73.6-79.5)	78.4 (75.5-81.3)	77.7 (74.5-80.9)	.002
**Ideal[c](≥64)**									
Grocery stores	4.12 (3.26-4.98)	4.82 (3.89-5.75)	4.60 (3.16-6.03)	6.99 (6.06-7.92)	7.73 (6.60-8.86)	7.49 (6.19-8.78)	6.75 (5.72-7.77)	6.16 (4.58-7.75)	.02
Restaurants	0.01 (0-0.04)	0.17 (0.03-0.32)	0.04 (0-0.08)	0.06 (0-0.14)	0.25 (0.03-0.48)	0.26 (0-0.54)	0.43 (0-0.88)	0.49 (0-1.27)	.02
Worksites	0.13 (0-0.37)	0.50 (0-1.08)	0.57 (0-1.26)	1.70 (0.08-3.32)	0.84 (0-1.95)	2.44 (0.43-4.44)	1.12 (0-2.53)	0.20 (0-0.50)	.20
Others	1.25 (0.33-2.17)	0.65 (0.29-1.01)	1.36 (0.46-2.27)	0.95 (0.45-1.44)	0.92 (0.43-1.40)	1.20 (0.77-1.63)	1.43 (0.75-2.11)	0.94 (0.22-1.65)	.98

Abbreviations: NHANES, National Health and Nutrition Examination Survey.

Food sources were grouped as grocery stores (consumed food items obtained from “grocery or supermarket”), restaurants (“restaurant fast food/pizza, restaurant with waiter/waitress, restaurant no additional information”), schools (“K-12 school cafeteria, childcare center”), worksites (“cafeteria not in a K-12 school, vending machine, common coffee pot or snack tray) and other sources refer to food items obtained from elsewhere (“sport, recreation, or entertainment facility, street vendor, vending truck, from someone else/gift, and others).

[a] Sensitivity analysis refers to diet quality based on the AHA score excluding fish/shellfish with a total score multiplied by 80/70.

[b] Percentages and CIs were estimated using survey weights to be nationally representative.

[c] Poor die quality was defined as being less than 40% adherent (<32 points). Intermediate was defined as adherence of 40%-79.9% (32-63.9 points). Ideal was defined as 80% adherence or greater (≥64 points).

© 2021 Liu J et al. *JAMA Network Open.*

eTable 13. Trends in Proportion of US Adults Aged 20+ Years with Poor, Intermediate or Ideal Diet from Restaurants based on American Heart Association Diet Score, NHANES 2003-2018. [a]

Diet Quality by AHA Scores	Survey-weighted AHA mean score (95%CI)								*P*-trend
	2003-2004	2005-2006	2007-2008	2009-2010	2011-2012	2013-2014	2015-2016	2017-2018	
**Full-Service Restaurant**									
Poor[b]	49.2 (44.7-53.8)	50.1 (45.5-54.7)	53.9 (50.4-57.4)	50.0 (46.1-54.0)	45.5 (41.4-49.5)	43.6 (38.4-48.8)	52.0 (47.3-56.7)	52.5 (47.1-57.9)	0.96
Intermediate[b]	50.6 (46.1-55.2)	49.9 (45.3-54.5)	46.0 (42.4-49.5)	50.0 (46.0-53.9)	54.2 (50.4-58.0)	56.3 (51.1-61.5)	47.9 (43.2-52.7)	47.3 (41.8-52.8)	0.98
Ideal[b]	0.13 (0-0.39)	0	0.18 (0-0.53)	0	0.32 (0-0.75)	0.07 (0-0.17)	0.07 (0-0.22)	0.17 (0-0.52)	0.70
**Fast-Food Restaurant**									
Poor[a]	74.9 (72.2-77.7)	79.3 (76.2-82.4)	76.4 (74.0-78.8)	75.6 (71.8-79.3)	72.8 (69.9-75.7)	71.2 (68.7-73.6)	70.1 (66.0-74.3)	72.9 (69.2-76.7)	0.001
Intermediate[b]	25.1 (22.3-27.8)	20.6 (17.5-23.7)	23.6 (21.2-26.0)	24.4 (20.7-28.2)	27.2 (24.3-30.1)	28.9 (26.4-31.3)	29.9 (25.7-34.0)	27.1 (23.3-30.8)	0.001
Ideal[b]	0	0.17 (0.06-0.28)	0	0	0	0	0	0	

Abbreviations: NHANES, National Health and Nutrition Examination Survey.
full-service restaurants refer to food/beverages consumed from “restaurant with waiter/waitress”; and fast-food restaurants refer to food/beverages consumed from “restaurant fast food/pizza”.

[a] Percentages and CIs were estimated using survey weights to be nationally representative.

[b] Poor die quality was defined as being less than 40% adherent (<32 points). Intermediate was defined as adherence of 40%-79.9% (32-63.9 points). Ideal was defined as 80% adherence or greater (≥64 points).

© 2021 Liu J et al. *JAMA Network Open.*

eTable 14. Trends in Estimated Percentage of Energy Intake from Grocery Stores, Restaurants, Schools and Other Sources Among American Children aged 5-19 years by Age, Sex, Race/Ethnicity, Parental Education, and Income, NHANES 2003-2018.

	Survey-weighted % (95% CI) [a]								P for trend	2017-2018 versus 2003-2004, difference (95% CI)
	2003-2004	2005-2006	2007-2008	2009-2010	2011-2012	2013-2014	2015-2016	2017-2018		
**Grocery stores**										
**Age group, y**										
5-11	69.6 (66.8-72.5)	68.7 (66.4-71)	68 (65.7-70.3)	68.9 (66.3-71.4)	66.8 (64.4-69.3)	66.4 (63.9-68.9)	65.9 (62.5-69.3)	68.8 (65.9-71.7)	.15	-0.82 (-4.90, 3.24)
12-19	66.2 (64.5-67.8)	66.6 (64.5-68.8)	65.0 (62.7-67.2)	68.2 (66.5-70)	59.9 (56.6-63.3)	62.9 (60.9-64.9)	60.8 (58.5-63)	61.0 (57.5-64.4)	<.001	-5.20 (-9.0, -1.40)
P for interaction				.35						
**Sex**										
Female	67.3 (65.2-69.5)	67.6 (65.2-70)	66.2 (63.7-68.8)	67.7 (65.9-69.4)	61.1 (58.7-63.4)	63.9 (61.2-66.6)	62.4 (60.7-64.1)	63.8 (61.2-66.3)	<.001	-3.60 (-6.9, -0.24)
Male	68.1 (66-70.3)	67.6 (65.8-69.3)	66.4 (64.3-68.5)	69.4 (67.2-71.6)	65.0 (62.7-67.2)	65.0 (63.0-67.0)	63.9 (59.9-67.8)	65.3 (61.7-68.9)	.01	-2.80 (-7.0, 1.38)
P for interaction				.75						
**Race/ethnicity[b]**										
Non-Hispanic white	67.0 (64.5-69.6)	69.1 (67.4-70.8)	66.4 (63-69.8)	70.4 (67.4-73.4)	62.2 (59.2-65.2)	65.7 (63.1-68.3)	63.8 (60.2-67.4)	67.1 (62.9-71.2)	.08	0.02 (-4.80, 4.89)
Non-Hispanic black	68.6 (63.3-74)	63.4 (59.2-67.5)	63.7 (59.2-68.3)	63.2 (61.1-65.4)	61.0 (55.3-66.6)	61.8 (58.4-65.2)	62.7 (60.2-65.3)	60.5 (57.9-63.1)	.01	-8.10 (-14.0, -2.20)
Hispanics	69 (65.9-72.1)	67 (63.2-70.9)	67.2 (64-70.4)	67.1 (64.5-69.6)	65.6 (62.8-68.3)	62.2 (59.4-65)	61.3 (58.5-64)	60.8 (56.8-64.8)	<.001	-8.20 (-13.0, -3.10)
P for interaction				.33						
**Education**										
<High school graduate	68.9 (65.1-72.6)	69.1 (65.9-72.3)	67.4 (65.5-69.4)	65.7 (64.2-67.3)	65.8 (63.6-68.1)	63.6 (61.1-66.1)	63.1 (59.8-66.3)	64.3 (60.4-68.1)	.001	-4.59 (-9.96, 0.78)
High school graduate or GED or some college	67.0 (64.7-69.2)	66.8 (65.0-68.7)	64.6 (61.3-67.9)	68.3 (66.2-70.4)	60.4 (57.6-63.2)	64.3 (62.0-66.5)	62.0 (59.3-64.8)	62.9 (59.2-66.6)	.001	-4.06 (-8.340.22)
College graduate or above	67.5 (63.9-71.1)	67.6 (64.8-70.7)	69.6 (66.6-72.6)	70.3 (66.7-74.0)	66.1 (62.5-69.8)	65.1 (60.5-69.7)	65.0 (60.4-69.6)	68.6 (64.9-72.2)	.27	1.06 (-4.08, 6.21)
P for interaction				.45						
**Ratio of family income to poverty level[c]**										
<1.30 [d]	68.5 (64.7-72.3)	67.6 (64.3-71)	67.1 (64.2-70.0)	67.9 (65.6-70.2)	62.8 (58.9-66.7)	64.0 (61.1-66.9)	63.4 (60.8-66)	63.0 (57.7-68.2)	.005	-5.53 (-12.0, 0.95)

© 2021 Liu J et al. *JAMA Network Open.*

	Survey-weighted % (95% CI) [a]								P for trend	2017-2018 versus 2003-2004, difference (95% CI)
	2003-2004	2005-2006	2007-2008	2009-2010	2011-2012	2013-2014	2015-2016	2017-2018		
1.30-3.49	67.3 (63.7-70.9)	68.7 (66.5-71.0)	66.2 (62.6-69.8)	68.1 (66.5-69.6)	62.4 (59.8-64.9)	64.1 (60.9-67.3)	63.6 (60.8-66.3)	64.2 (61.1-67.3)	.004	-3.14 (-7.89, 1.63)
≥3.50 [e]	67.0 (62.9-71.2)	65.9 (63.6-68.3)	65.6 (62.5-68.8)	68.7 (64.2-73.1)	63.1 (57.8-68.4)	65.0 (60.9-69.1)	62.2 (56.9-67.4)	68.3 (64.6-72.0)	.51	1.24 (-4.31, 6.80)
P for interaction				.42						
				**Restaurants**						
**Age group, y**										
5-11	15.2 (13.3-17.2)	13.0 (11.5-14.4)	13.1 (10.7-15.5)	12.3 (11.1-13.5)	13.3 (11.3-15.2)	14.4 (12.7-16)	14.7 (12.7-16.6)	15.1 (13.1-17.2)	.33	-0.11 (-2.90, 2.69)
12-19	22.8 (21.1-24.5)	22.3 (21-23.6)	21.4 (19.3-23.5)	18.8 (17.2-20.5)	24.5 (21-28.1)	22.5 (21.2-23.9)	21.8 (19.5-24.2)	24.7 (22.1-27.4)	.14	1.95 (-1.20, 5.06)
P for interaction				<.001						
**Sex**										
Female	19.0 (17.5-20.4)	17.8 (16.1-19.4)	18.4 (16.2-20.7)	16.3 (14.7-17.9)	20.5 (18.0-23.1)	19.5 (18-21)	19.2 (17.4-21)	21.5 (18.9-24.2)	.02	2.56 (-0.48, 5.59)
Male	19.6 (17.8-21.5)	18.4 (16.6-20.2)	16.9 (15.1-18.8)	15.4 (14.3-16.5)	18.3 (15.3-21.3)	18.2 (16.2-20.2)	17.9 (16.2-19.6)	19.2 (16.5-21.8)	.95	-0.46 (-3.70, 2.77)
P for interaction				0.46						
**Race/ethnicity[b]**										
Non-Hispanic white	20.7 (18.7-22.7)	18.6 (17.3-19.9)	18.5 (15.9-21.1)	15.5 (13.7-17.3)	21.3 (17.4-25.3)	19.3 (17.3-21.3)	18.3 (16.1-20.5)	19.5 (16.8-22.2)	.84	-1.20 (-4.5, 2.13)
Non-Hispanic black	17.7 (16-19.5)	17.1 (14.4-19.9)	18.4 (15.5-21.2)	18.1 (16.1-20.1)	17.7 (14.8-20.6)	20.1 (17.5-22.7)	19.1 (17.2-20.9)	22.0 (19.8-24.1)	.004	4.23 (1.47, 7.0)
Hispanics	16.8 (14.6-19)	15.1 (13.1-17.1)	15.3 (14-16.7)	15.9 (13.7-18)	16.5 (15.5-17.4)	17.8 (15-20.6)	18.8 (16.3-21.3)	21.6 (17.9-25.4)	<.001	4.83 (0.47, 9.19)
P for interaction				.001						
**Education**										
<High school graduate	17.5 (15.3-19.6)	14.0 (12.3-15.7)	15.4 (12.9-17.8)	15.1 (13.6-16.6)	16.2 (13.4-19.0)	15.7 (13.2-18.1)	17.0 (13.9-20.1)	17.6 (13.7-21.4)	.38	0.009 (-4.30, 4.48)
High school graduate or GED or some college	19.9 (18.3-21.4)	18.3 (16.9-19.8)	19.8 (17.1-22.5)	16.6 (14.8-18.3)	21.1 (17.2-24.9)	19.1 (17.4-20.8)	18.3 (16.5-20.1)	20.6 (18.1-23.1)	.60	0.72 (-2.22, 3.66)
College graduate or above	19.6 (15.2-24.0)	21.2 (18.4-24.1)	16.0 (13.7-18.3)	15.5 (11.6-19.4)	19.2 (15.5-22.8)	20.8 (17.4-24.3)	19.5 (16.2-22.7)	20.5 (16.8-24.1)	.36	0.86 (-4.85, 6.57)
P for interaction				.01						

© 2021 Liu J et al. *JAMA Network Open.*

	Survey-weighted % (95% CI) [a]									
	2003-2004	2005-2006	2007-2008	2009-2010	2011-2012	2013-2014	2015-2016	2017-2018	P for trend	2017-2018 versus 2003-2004, difference (95% CI)
**Ratio of family income to poverty level[c]**										
<1.30 [d]	17.1 (14.7-19.4)	14.5 (11.8-17.1)	16.0 (13.7-18.4)	14.1 (12.2-15.9)	16.8 (12.8-20.8)	15.0 (13.2-16.8)	16.4 (14.1-18.7)	19.7 (16.4-23)	.16	2.65 (-1.30, 6.64)
1.30-3.49	20.3 (18.2-22.4)	16.8 (15.0-18.5)	18.8 (16.7-20.9)	16.2 (15-17.4)	20.4 (16.6-24.2)	20.4 (17.4-23.4)	17.0 (15.1-18.8)	19.5 (17.3-21.7)	.80	-0.79 (-3.80, 2.26)
≥3.50 [e]	20.9 (16.1-25.8)	22.4 (19.8-24.9)	18.6 (16.6-20.6)	17.9 (14.7-21.0)	22.1 (17.7-26.5)	21.6 (19.0-24.3)	23.0 (19.8-26.2)	21.2 (17.7-24.8)	.47	0.27 (-5.78, 6.31)
P for interaction				.01						
					**School**					
**Age group, y**										
5-11	9.97 (7.78-12.2)	9.98 (8.03-11.9)	11.6 (8.77-14.5)	11.7 (9.56-13.9)	12.3 (10.1-14.6)	12.4 (9.65-15.1)	11.9 (9.57-14.3)	8.66 (5.92-11.4)	.84	-1.30 (-4.80, 2.21)
12-19	6.19 (4.48-7.90)	5.51 (4.38-6.65)	6.87 (5.01-8.73)	6.53(4.74-8.32)	6.49 (4.83-8.14)	6.46 (5.02-7.9)	7.26 (5.8-8.73)	5.37 (3.80-6.94)	.87	-0.82 (-3.10, 1.50)
P for interaction				<0.001						
**Sex**										
Female	8.20 (6.36-10)	7.62 (6.71-8.53)	8.56 (6.26-10.9)	8.62 (6.78-10.5)	9.59 (7.59-11.6)	8.80 (7.2-10.4)	9.42 (7.98-10.9)	6.57 (4.54-8.59)	.93	-1.60 (-4.4, 1.1)
Male	7.68 (5.60-9.76)	7.45 (5.89-9.02)	9.47 (7.11-11.8)	9.19 (7.21-11.2)	8.72 (6.56-10.9)	9.50 (7.21-11.8)	9.43 (7.42-11.4)	7.19 (5.02-9.36)	.64	-0.49 (-3.50, 2.51)
P for interaction				0.68						
**Race/ethnicity[b]**										
Non-Hispanic white	6.97 (4.98-8.95)	4.96 (3.6-6.33)	7.32 (4-10.6)	6.5 (4.05-8.94)	7.78 (5.37-10.2)	7.10 (4.77-9.43)	7.93 (6.56-9.3)	4.70 (2.63-6.77)	.98	-2.30 (-5.1, 0.60)
Non-Hispanic black	8.96 (4.97-13)	14.3 (11.2-17.3)	11.9 (8.58-15.3)	14.4 (11.2-17.6)	12.6 (7.26-17.9)	11.7 (9.62-13.8)	11.4 (8.24-14.6)	9.86 (7.87-11.9)	.72	0.90 (-3.6, 5.36)
Hispanics	9.86 (7.94-11.8)	11.9 (9.57-14.2)	11.8 (9.12-14.5)	11.7 (9.68-13.7)	11.0 (8.46-13.5)	12.7 (10.2-15.2)	11.3 (9.60-13)	9.84 (7.24-12.5)	.78	-0.01 (-3.2, 3.22)
P for interaction				<0.001						
**Parental Education**										
<High school graduate	9.81 (6.74-12.9)	11.1 (8.42-13.8)	10.7 (8.07-13.3)	13.2 (11.1-15.3)	10.4 (8.87-11.9)	12.0 (9.26-14.8)	10.5 (8.34-12.6)	10.7 (7.34-14.1)	.83	.91 (-3.65, 5.46)
High school graduate or GED or some college	7.86 (5.51-8.65)	7.56 (6.47-8.65)	9.07 (7.01-11.1)	8.90 (6.97-10.8)	10.1 (7.87-12.3)	9.84 (8.55-11.1)	11.0 (9.67-12.3)	7.21 (4.93-9.49)	.21	-0.65 (-3.92, 2.62)

© 2021 Liu J et al. *JAMA Network Open.*

	Survey-weighted % (95% CI) [a]								P for trend	2017-2018 versus 2003-2004, difference (95% CI)
	2003-2004	2005-2006	2007-2008	2009-2010	2011-2012	2013-2014	2015-2016	2017-2018		
College graduate or above	7.15 (4.69-9.61)	4.43 (2.29-6.57)	7.92 (4.31-11.5)	6.27 (3.82-8.71)	5.92 (3.20-8.64)	5.68 (3.24-8.11)	6.30 (4.42-8.18)	4.23 (2.81-5.66)	.19	-2.91 (-5.76, -0.08)
P for interaction				.02						
**Ratio of family income to poverty level**[c]										
<1.30 [d]	10.4 (7.43-13.4)	11.6 (9.82-13.4)	10.9 (8.95-12.9)	12.5 (10.6-14.5)	12.6 (9.61-15.5)	13.2 (11.0-15.3)	12.6 (10.9-14.2)	8.60 (6.05-11.1)	.98	-1.80 (-5.70, 2.11)
1.30-3.49	7.49 (4.83-10.2)	7.03 (5.46-8.59)	7.59 (5.72-9.46)	8.73 (6.07-11.4)	7.98 (6.10-9.86)	8.79 (6.89-10.7)	9.87 (7.81-11.9)	7.66 (5.38-9.94)	.19	0.17 (-3.30, 3.67)
≥3.50 [e]	6.22 (4.85-7.58)	5.27 (3.22-7.32)	8.55 (4.21-12.9)	5.30 (2.0-8.60)	6.53 (3.75-9.31)	4.80 (2.78-6.82)	5.67 (3.74-7.60)	3.01 (1.554.48)	.02	-1.20 (-3.50, 1.09)
P for interaction				<.001						
				**Others**						
**Age group, y**										
5-11	5.17 (4.42-5.93)	8.32 (7.38-9.27)	7.27 (6.04-8.5)	7.11 (6.08-8.13)	7.59 (6.33-8.84)	6.83 (5.74-7.93)	7.48 (6.56-8.40)	7.41 (5.54-9.28)	.20	2.24 (0.22, 4.25)
12-19	4.86 (4.34-5.38)	5.52 (4.61-6.44)	6.77 (5.34-8.21)	6.41 (5.52-7.31)	9.05 (7.14-11.0)	8.16 (6.72-9.59)	10.1 (8.31-12.0)	8.93 (6.59-11.3)	<.001	4.08 (1.68, 6.47)
P for interaction				<0.001						
**Sex**										
Female	5.48 (4.73-6.23)	7.01 (6.42-7.6)	6.78 (5.67-7.88)	7.42 (6.52-8.32)	8.82 (6.84-10.8)	7.82 (6.71-8.93)	9.03 (7.59-10.5)	8.14 (6.18-10.1)	<.001	2.66 (0.56, 4.76)
Male	4.55 (3.79-5.31)	6.58 (5.55-7.6)	7.22 (5.82-8.61)	6.03 (4.93-7.13)	7.97 (6.34-9.6)	7.30 (5.97-8.64)	8.80 (6.99-10.6)	8.32 (6.59-10.0)	<.001	3.77 (1.88, 5.65)
P for interaction				0.11						
**Race/ethnicity**[b]										
Non-Hispanic white	5.28 (4.57-5.99)	7.37 (6.09-8.65)	7.77 (6.48-9.06)	7.53 (6.54-8.53)	8.70 (6.71-10.7)	7.88 (6.28-9.48)	9.94 (7.68-12.2)	8.71 (6.15-11.3)	.001	3.43 (0.77, 6.09)
Non-Hispanic black	4.67 (3.3-6.04)	5.23 (4.59-5.87)	5.99 (4.9-7.08)	4.33 (3.41-5.24)	8.75 (4.7-12.8)	6.38 (5.26-7.49)	6.74 (5.16-8.33)	7.66 (6.38-8.93)	<.001	2.98 (1.12, 4.85)
Hispanics	4.39 (3.39-5.39)	5.97 (4.39-7.56)	5.62 (4.28-6.97)	5.39 (4.45-6.33)	6.99 (6.07-7.92)	7.30 (5.88-8.72)	8.64 (6.9-10.4)	7.73 (6.26-9.20)	<.001	3.34 (1.56, 5.12)
P for interaction				0.06						
**Education**										

© 2021 Liu J et al. *JAMA Network Open.*

	Survey-weighted % (95% CI) [a]								P for trend	2017-2018 versus 2003-2004, difference (95% CI)
	2003-2004	2005-2006	2007-2008	2009-2010	2011-2012	2013-2014	2015-2016	2017-2018		
<High school graduate	3.86 (3.23-4.49)	5.74 (4.43-7.05)	6.47 (4.64-8.30)	5.96 (4.85-7.06)	7.62 (6.06-9.18)	8.71 (5.99-11.4)	9.49 (7.40-11.6)	7.46 (5.88-9.03)	<.001	3.86 (3.23, 4.49)
High school graduate or GED or some college	5.30 (4.73-5.88)	7.27 (6.56-7.97)	6.55 (5.29-7.81)	6.27 (5.20-7.33)	8.46 (6.63-10.3)	6.82 (5.72-7.93)	8.65 (6.65-10.6)	9.30 (6.59-12.0)	.001	4.0 (1.23, 6.76)
College graduate or above	5.75 (4.18-7.33)	6.59 (5.10-8.08)	6.50 (5.17-7.83)	7.88 (6.07-9.69)	8.80 (6.22-11.4)	8.40 (7.46-9.34)	9.24 (6.94-11.5)	6.75 (4.90-8.60)	.04	0.99 (-1.43, 3.42)
P for interaction				.02						
**Ratio of family income to poverty level[c]**										
<1.30 [d]	4.05 (3.41-4.69)	6.31 (4.82-7.79)	5.94 (4.83-7.04)	5.50 (4.49-6.51)	7.84 (5.99-9.7)	7.85 (6.27-9.43)	7.65 (5.98-9.32)	8.74 (4.65-12.8)	.002	4.68 (0.54, 8.82)
1.30-3.49	4.93 (3.91-5.95)	7.46 (6.36-8.56)	7.40 (5.94-8.87)	7.04 (5.90-8.17)	9.26 (5.82-12.7)	6.75 (5.36-8.13)	9.61 (7.37-11.8)	8.68 (7.18-10.2)	<.001	3.76 (1.94, 5.57)
≥3.50 [e]	5.79 (4.77-6.81)	6.44 (5.0-7.88)	7.24 (4.89-9.59)	8.15 (6.46-9.84)	8.24 (6.16-10.3)	8.58 (6.53-10.6)	9.16 (6.58-11.7)	7.47 (5.30-9.66)	.02	1.37 (-0.80, 3.54)
P for interaction				.08						

Abbreviations: NHANES, National Health and Nutrition Examination Survey.
Food sources were grouped as grocery stores (consumed food items obtained from “grocery or supermarket”), restaurants (“restaurant fast food/pizza, restaurant with waiter/waitress, restaurant no additional information”), schools (“K-12 school cafeteria, childcare center”), worksites (“cafeteria not in a K-12 school, vending machine, common coffee pot or snack tray) and other sources refer to food items obtained from elsewhere (“sport, recreation, or entertainment facility, street vendor, vending truck, from someone else/gift, and others).
[a] All percentages were survey-weighted; %, proportion.
[b] Trends in diet for the other race or mixed race group are not presented in the race/ethnicity-stratified results due to their small sample sizes.
[c] Represents the ratio of family income to the federal poverty threshold, adjusting for household size. For reference, the federal threshold in 2016 for a family of 4 was $24,300/y. A family of 4 earning $33,534/y would have a ratio of 1.38.
[d] Indicates a lower level of income.
[e] Indicates a higher level of income.

© 2021 Liu J et al. *JAMA Network Open.*

eTable 15. Trends in Estimated Percentage of Energy Intake from Grocery Stores, Restaurants, Worksites and Other Sources Among Adults Aged 20 Years or Older by Age, Sex, Race/Ethnicity, Education and Income, NHANES 2003 to 2018.

	Survey-weighted % (95% CI) [a]								P for trend	2017-2018 versus 2003-2004, difference (95% CI)
	2003-2004	2005-2006	2007-2008	2009-2010	2011-2012	2013-2014	2015-2016	2017-2018		
**Grocery stores**										
**Age group, y**										
20-49	65.3 (64.0-66.5)	66.4 (64.8-68.1)	67.9 (66.3-69.5)	69.4 (68.0-70.8)	62.6 (60.3-64.9)	63.5 (61.8-65.2)	64.0 (62.2-65.8)	63.6 (61.8-65.5)	<.001	-1.70 (-3.90, 0.56)
≥50	73.8 (72.3-75.3)	72.7 (70.8-74.6)	74.8 (72.3-77.3)	76.1 (74.9-77.3)	73.3 (71.5-75.2)	71.9 (70.7-73.1)	73.3 (71.6-75)	71.2 (69.1-73.4)	.02	-2.50 (-5.10, 0.03)
P for interaction				<.001						
**Sex**										
Female	69.5 (68.0-70.9)	71.0 (69.7-72.2)	72.5 (70.5-74.5)	73.7 (72.4-75)	69.1 (67.1-71.1)	68.9 (67.8-70)	70.2 (68.4-72)	69.2 (67.5-70.9)	.04	-0.28 (-2.60, 1.99)
Male	68.1 (66.5-69.8)	67.0 (65.2-68.9)	69.0 (67-70.9)	70.8 (69.3-72.3)	65.8 (63.6-68.1)	65.7 (64.6-66.8)	66.5 (64.9-68.2)	65.2 (63.3-67.1)	.001	-2.90 (-5.40, -0.41)
P for interaction				.002						
**Race/ethnicity[b]**										
Non-Hispanic white	68.4 (66.9-69.9)	68.4 (66.7-70.1)	70.5 (68.3-72.8)	71.7 (70.4-73)	67.6 (65.2-70.1)	67.5 (66.4-68.5)	69.1 (67.0-71.2)	67.4 (64.9-70)	.21	-1.0 (-4.0, 1.96)
Non-Hispanic black	70.6 (66.9-74.4)	68.7 (66.5-70.9)	69.9 (67.2-72.6)	74.1 (71.5-76.6)	64.4 (60.5-68.2)	64.1 (62.3-66.0)	63.6 (60.6-66.5)	63.3 (60.7-65.9)	<.001	-7.30 (-12.0, -2.8)
Hispanics	70.7 (68.4-73.1)	71.8 (68.6-75.0)	71.6 (69.2-73.9)	73.7 (72.0-75.4)	68.8 (64.5-73.1)	68.8 (66.3-71.4)	67.2 (65.3-69.2)	67.4 (65.4-69.5)	<.001	-3.30 (-6.40, -0.19)
P for interaction				.02						
**Education**										
<High school graduate	74.9 (73.6-76.2)	75.1 (72.9-77.3)	75.2 (73.1-77.2)	77.3 (74.9-79.7)	73.2 (71.6-74.7)	71.5 (69.6-73.4)	71.5 (67.9-75)	72.6 (69.8-75.4)	.002	-2.30 (-5.40, 0.77)
High school graduate or GED	68.1 (66-70.2)	69.7 (67.4-72)	71.5 (69.3-73.6)	71.0 (68.9-73.1)	68.8 (66.1-71.5)	67.0 (65.4-68.6)	68.8 (66.4-71.2)	67.0 (63.6-70.4)	.11	-1.10 (-5.1, 2.86)
Some college or above	67.8 (65.6-69.9)	68.2 (65.9-70.4)	68.6 (66.5-70.7)	70.6 (69.4-71.8)	65.9 (62.3-69.5)	66.0 (64.5-67.5)	67.5 (65.4-69.6)	65.2 (62.5-67.8)	.02	-2.60 (-6.0, 0.80)
College graduate or above	66.4 (63.6-69.2)	65.5 (63.6-67.4)	69.2 (65.9-72.5)	72.0 (69.5-74.6)	65.4 (63.3-67.5)	67.1 (65.4-68.8)	67.9 (65.4-70.4)	67.9 (65.1-70.6)	.68	1.48 (-2.40, 5.37)
P for interaction				<.001						
**Ratio of family income to poverty level[c]**										

© 2021 Liu J et al. *JAMA Network Open.*

	Survey-weighted % (95% CI)[a]								P for trend	2017-2018 versus 2003-2004, difference (95% CI)
	2003-2004	2005-2006	2007-2008	2009-2010	2011-2012	2013-2014	2015-2016	2017-2018		
<1.30[d]	73.4 (71.5-75.3)	75.9 (74-77.9)	74.8 (72.5-77.1)	73.5 (71.8-75.2)	69.4 (62.9-75.9)	69.5 (67.1-71.9)	70.1 (67.5-72.8)	69.1 (65.2-73)	<.001	-4.30 (-8.60, 0.10)
1.30-3.49	68.7 (65.9-71.5)	70.8 (68.5-73.1)	71.7 (68.8-74.6)	73.7 (71.8-75.5)	67.8 (65.4-70.2)	67.9 (65.7-70)	68.3 (66.5-70)	67.6 (65.6-69.6)	.02	-1.10 (-4.50, 2.33)
≥3.50[e]	66.9 (65-68.7)	65.1 (63.2-66.9)	68.0 (65.6-70.4)	70.0 (67.8-71.5)	65.2 (62.7-67.7)	65.3 (63.8-66.9)	66.9 (64.3-69.6)	65.8 (63.1-68.4)	.50	-0.46 (-3.30, 2.40)
P for interaction				<.001						
**Restaurants**										
**Age group, y**										
20-49	25.8 (24.4-27.1)	24.1 (22.3-25.9)	23.6 (21.9-25.3)	22.1 (21.2-23)	25.5 (24.3-26.8)	26.3 (24.3-28.3)	25.4 (23.7-27.2)	26.4 (24.4-28.4)	.05	0.64 (-1.80, 3.05)
≥50	17.2 (16.1-18.2)	17.4 (15.9-18.8)	16.8 (14.9-18.8)	14.9 (13.8-15.9)	16.9 (15.6-18.3)	18.4 (17.2-19.7)	16.6 (14.9-18.3)	18.3 (16.5-20.1)	.24	1.11 (-0.99, 3.22)
P for interaction				<.001						
**Sex**										
Female	21.1 (19.9-22.3)	19.5 (18-21.1)	18.9 (17.1-20.8)	17.3 (16.2-18.4)	20.2 (19.0-21.4)	21.8 (20.2-23.4)	19.8 (18.2-21.4)	21.0 (19.0-23.0)	.23	-0.07 (-2.40, 2.28)
Male	23.4 (21.9-24.8)	23.1 (21.2-25)	22.7 (20.7-24.8)	20.7 (19.5-22.0)	23.1 (21.4-24.8)	23.6 (22.2-25.1)	22.8 (20.9-24.7)	24.1 (22.4-25.8)	.40	0.75 (-1.50, 3.03)
P for interaction				<.001						
**Race/ethnicity[b]**										
Non-Hispanic white	22.2 (20.8-23.5)	21.2 (19.2-23.2)	20.7 (18.5-22.8)	19.2 (18.2-20.3)	21.8 (20-23.6)	22.7 (20.9-24.5)	20.5 (18.6-22.4)	22.2 (19.7-24.8)	.67	0.07 (-2.80, 2.97)
Non-Hispanic black	22.0 (19.2-24.9)	23.2 (21.3-25.2)	22.5 (20.6-24.3)	18.6 (16.2-21)	23.1 (21.4-24.8)	24.2 (21.7-26.8)	25.3 (22.6-28)	26.0 (23.6-28.5)	.003	4.01 (0.26, 7.76)
Hispanics	21.9 (19.9-23.8)	20.0 (17.3-22.8)	20.3 (18-22.6)	18.3 (16.2-20.3)	20.7 (17.6-23.7)	21.5 (19.1-23.9)	22.6 (20.4-24.7)	22.7 (20.8-24.6)	.04	0.82 (-1.90, 3.54)
P for interaction				.61						
**Education**										
<High school graduate	17.5 (15.8-19.1)	17.2 (14.8-19.7)	16.6 (15-18.3)	15.5 (13.7-17.3)	17.5 (15.8-19.2)	18.5 (16.8-20.3)	17.7 (14.5-20.8)	18.9 (16.5-21.3)	.21	1.42 (-1.5, 4.3)
High school graduate or GED	23.4 (21-25.8)	20.9 (18.7-23.1)	21 (18.5-23.4)	20.3 (18.7-21.9)	21.0 (18.8-23.2)	21.9 (20-23.9)	20.4 (18.1-22.7)	22.4 (19.2-25.5)	.81	-1.0 (-5, 2.88)
Some college or above	22.6 (20.9-24.3)	21.7 (19.5-23.9)	22.3 (20.4-24.2)	20.7 (19.7-21.6)	23.0 (20.9-25.1)	24.5 (22.8-26.2)	22.5 (20.9-24)	24.4 (21.8-27.0)	.06	1.81 (-1.3, 4.88)

© 2021 Liu J et al. *JAMA Network Open.*

	Survey-weighted % (95% CI)[a]								P for trend	2017-2018 versus 2003-2004, difference (95% CI)
	2003-2004	2005-2006	2007-2008	2009-2010	2011-2012	2013-2014	2015-2016	2017-2018		
College graduate or above	23.9 (21.9-26)	23.7 (21.5-26)	22.0 (19.3-24.6)	18.2 (16.1-20.2)	22.7 (20.8-24.6)	23.3 (21.1-25.5)	22.1 (19.3-24.8)	22.0 (20.0-23.9)	.49	-1.90 (-4.7, 0.88)
P for interaction				<.001						
**Ratio of family income to poverty level**[c]										
<1.30[d]	19.2 (17.6-20.8)	15.6 (14.2-17.0)	17.2 (15.5-18.8)	18.3 (16.6-20.0)	20.1 (16.5-23.7)	20.2 (17.8-22.5)	19.2 (17-21.4)	19.8 (16.7-23)	.04	0.65 (-2.9, 4.21)
1.30-3.49	22.3 (19.6-25.0)	20.0 (17.9-22.0)	20.0 (17.5-22.6)	18.2 (16.7-19.8)	21.5 (18.9-24.1)	22.4 (20.2-24.6)	21.6 (20.2-22.9)	22.2 (20.6-23.8)	.20	-0.08 (-3.2, 3.06)
≥3.50[e]	24.0 (22.0-26.0)	24.5 (22.1-26.8)	23.2 (20.6-25.7)	20.7 (19.2-22.1)	23.3 (21.2-25.5)	24.8 (22.9-26.6)	22.6 (20.1-25.2)	24.0 (21.0-26.9)	.94	-0.004 (-3.56, 3.55)
P for interaction				<.001						
**Worksites**										
**Age group, y**										
20-49	2.70 (2.12-3.29)	2.23 (1.80-2.66)	1.48 (1.22-1.75)	1.72 (1.31-2.13)	2.62 (1.75-3.49)	1.50 (1.27-1.72)	1.26 (0.88-1.63)	1.65 (1.30-2.01)	<.001	-1.10 (-1.70, -0.36)
≥50	1.32 (0.82-1.81)	1.63 (1.26-1.99)	1.16 (0.81-1.52)	1.30 (0.92-1.68)	1.13 (0.78-1.47)	0.82 (0.58-1.07)	0.81 (0.47-1.15)	0.99 (0.57-1.40)	.006	-0.33 (-0.97, 0.32)
P for interaction				<.001						
**Sex**										
Female	2.19 (1.66-2.72)	1.76 (1.39-2.13)	1.23 (0.95-1.5)	1.56 (0.99-2.12)	1.70 (1.11-2.28)	0.97 (0.82-1.12)	1.0 (0.64-1.35)	1.24 (0.89-1.59)	<.001	-0.95 (-1.60, -0.31)
Male	2.06 (1.44-2.67)	2.21 (1.78-2.64)	1.49 (1.17-1.81)	1.52 (1.09-1.95)	2.19 (1.53-2.85)	1.41 (1.05-1.77)	1.1 (0.75-1.45)	1.44 (1.07-1.81)	.002	-0.62 (-1.30, 0.10)
P for interaction				.48						
**Race/ethnicity**[b]										
Non-Hispanic white	2.15 (1.44-2.86)	1.97 (1.56-2.37)	1.28 (0.96-1.59)	1.63 (1.18-2.09)	1.59 (1.16-2.01)	1.0 (0.78-1.22)	0.90 (0.56-1.25)	1.24 (0.96-1.52)	<.001	-0.91 (-1.70, -0.14)
Non-Hispanic black	2.24 (1.54-2.94)	2.43 (2.01-2.85)	1.96 (1.42-2.5)	1.58 (1.17-1.98)	3.71 (1.50-5.92)	2.16 (1.53-2.79)	1.56 (0.99-2.12)	1.55 (1.20-1.90)	.07	-0.69 (-1.50, 0.10)
Hispanics	1.53 (0.86-2.2)	1.94 (1.17-2.7)	1.36 (1.01-1.71)	1.23 (0.95-1.51)	2.04 (1.11-2.97)	1.21 (0.89-1.53)	1.02 (0.62-1.42)	1.53 (0.86-2.19)	.34	-0.002 (-0.94, 0.94)
P for interaction				.10						
**Education**										

© 2021 Liu J et al. *JAMA Network Open.*

	Survey-weighted % (95% CI) [a]								P for trend	2017-2018 versus 2003-2004, difference (95% CI)
	2003-2004	2005-2006	2007-2008	2009-2010	2011-2012	2013-2014	2015-2016	2017-2018		
<High school graduate	1.19 (0.69-1.69)	1.41 (0.64-2.18)	1.02 (0.73-1.32)	1.33 (0.53-2.14)	1.06 (0.46-1.67)	0.97 (0.59-1.35)	0.67 (0.35-0.99)	0.86 (0.47-1.24)	.05	-0.33 (-0.97, 0.30)
High school graduate or GED	1.43 (0.96-1.89)	2.08 (1.49-2.67)	1.28 (0.83-1.72)	1.32 (0.86-1.79)	1.56 (1.05-2.07)	1.16 (0.80-1.52)	0.76 (0.28-1.24)	1.37 (1.04-1.70)	.03	-0.06 (-0.63, 0.51)
Some college or above	2.96 (1.81-4.11)	2.0 (1.51-2.50)	1.47 (1.10-1.83)	1.50 (1.10-1.91)	2.43 (1.02-3.85)	1.22 (1.03-1.41)	1.10 (0.67-1.54)	1.50 (1.23-1.76)	.001	-1.46 (-2.65, -0.28)
College graduate or above	2.50 (1.75-3.24)	2.22 (1.58-2.85)	1.55 (1.04-2.06)	1.90 (1.56-2.23)	2.11 (1.44-2.79)	1.28 (0.97-1.59)	1.33 (0.82-1.84)	1.29 (0.69-1.88)	.001	-1.21 (-2.17, -0.26)
P for interaction				<.001						
**Ratio of family income to poverty level**[c]										
<1.30 [d]	1.34 (0.82-1.87)	1.15 (0.74-1.55)	0.77 (0.51-1.03)	1.23 (0.88-1.59)	2.57 (0.43-4.70)	1.04 (0.76-1.32)	0.90 (0.42-1.37)	1.25 (0.66-1.85)	.98	-0.09 (-0.88, 0.71)
1.30-3.49	2.03 (1.40-2.66)	2.06 (1.41-2.71)	1.01 (0.79-1.24)	1.41 (1.02-1.80)	1.72 (1.51-1.93)	1.08 (0.88-1.28)	0.90 (0.58-1.21)	1.72 (1.20-2.23)	.03	-0.31 (-1.10, 0.50)
≥3.50 [e]	2.51 (1.83-3.20)	2.27 (1.96-2.58)	1.95 (1.52-2.38)	1.82 (1.09-2.55)	1.27 (0.95-1.59)	1.29 (0.77-1.80)	1.29 (0.77-1.80)	1.21 (0.73-1.70)	<.001	-1.30 (-2.14, -0.46)
P for interaction				<.001						
				**Others**						
**Age group, y**										
20-49	6.25 (5.70-6.81)	7.27 (6.58-7.96)	7.03 (6.09-7.98)	6.80 (5.93-7.66)	9.23 (8.24-10.2)	8.69 (7.65-9.74)	9.33 (8.18-10.5)	8.32 (7.50-9.13)	<.001	2.06 (1.08, 3.05)
≥50	7.72 (6.54-8.9)	8.30 (6.75-9.85)	7.19 (6.42-7.95)	7.72 (6.73-8.72)	8.60 (6.95-10.2)	8.85 (7.97-9.74)	9.26 (7.85-10.7)	9.48 (8.27-10.7)	.005	1.76 (0.07, 3.45)
P for interaction				.06						
**Sex**										
Female	7.26 (6.77-7.74)	7.73 (6.62-8.85)	7.35 (6.43-8.27)	7.47 (6.80-8.14)	8.99 (7.89-10.1)	8.34 (7.47-9.21)	9.03 (7.98-10.1)	8.56 (7.33-9.79)	.003	1.30 (-0.02, 2.62)
Male	6.44 (5.61-7.28)	7.67 (6.59-8.74)	6.81 (5.92-7.70)	6.90 (6.30-7.51)	8.89 (7.54-10.2)	9.22 (8.14-10.3)	9.58 (8.31-10.9)	9.22 (8.29-10.2)	<.001	2.78 (1.53, 4.03)
P for interaction				.01						
**Race/ethnicity**[b]										
Non-Hispanic white	7.25 (6.49-8.01)	8.41 (7.13-9.7)	7.52 (6.62-8.42)	7.44 (6.85-8.02)	8.99 (7.62-10.4)	8.86 (7.78-9.95)	9.46 (8.07-10.8)	9.08 (7.84-10.3)	.002	1.83 (0.37, 3.29)
Non-Hispanic black	5.11 (3.95-6.27)	5.62 (4.69-6.55)	5.67 (4.76-6.57)	5.76 (4.67-6.86)	8.82 (7.6-10)	9.46 (8.11-10.8)	9.57 (8.27-10.9)	9.11 (7.94-10.3)	<.001	4.0 (2.36, 5.65)

© 2021 Liu J et al. *JAMA Network Open.*

	Survey-weighted % (95% CI)[a]								P for trend	2017-2018 versus 2003-2004, difference (95% CI)
	2003-2004	2005-2006	2007-2008	2009-2010	2011-2012	2013-2014	2015-2016	2017-2018		
Hispanics	5.88 (5.31-6.44)	6.20 (5.18-7.21)	6.78 (5.92-7.64)	6.80 (5.71-7.89)	8.47 (6.85-10.1)	8.48 (7.72-9.24)	9.20 (8.26-10.1)	8.37 (7.34-9.41)	<.001	2.50 (1.32, 3.68)
P for interaction				<.001						
**Education**										
<High school graduate	6.42 (5.28-7.56)	6.24 (4.82-7.66)	7.19 (5.33-9.06)	5.87 (4.7-7.04)	8.25 (6.92-9.59)	9.01 (8.03-9.99)	10.2 (8.33-12.1)	7.66 (6.24-9.09)	<.001	1.24 (-0.58, 3.07)
High school graduate or GED	7.08 (6.01-8.15)	7.28 (5.98-8.58)	6.27 (5.45-7.08)	7.40 (6.36-8.44)	8.67 (6.52-10.8)	9.91 (8.12-11.7)	10.1 (8.4-11.8)	9.30 (8.16-10.4)	<.001	2.22 (0.66, 3.78)
Some college or above	6.68 (6.01-7.36)	8.14 (6.86-9.43)	7.62 (6.69-8.55)	7.23 (6.38-8.09)	8.69 (6.69-10.7)	8.26 (7.1-9.42)	8.98 (7.53-10.4)	8.94 (7.61-10.3)	.003	2.26 (0.76, 3.75)
College graduate or above	7.19 (6.11-8.27)	8.55 (7.27-9.82)	7.27 (6.11-8.42)	7.91 (6.67-9.14)	9.74 (8.51-11)	8.34 (7.50-9.19)	8.72 (7.23-10.2)	8.85 (7.11-10.6)	.08	1.67 (-0.38, 3.71)
P for interaction				.047						
**Ratio of family income to poverty level**[c]										
<1.30[d]	6.09 (5.30-6.88)	7.29 (6.12-8.47)	7.29 (5.61-8.98)	6.97 (6.17-7.77)	7.89 (6.52-9.27)	9.32 (7.91-10.7)	9.74 (8.68-10.8)	9.78 (8.40-11.2)	<.001	3.70 (2.10, 5.29)
1.30-3.49	7.0 (6.01-7.99)	7.16 (5.67-8.65)	7.20 (5.66-8.73)	6.68 (6.0-7.37)	8.99 (7.55-10.4)	8.62 (7.5-9.74)	9.28 (8.29-10.3)	8.50 (7.58-9.41)	<.001	1.50 (0.15, 2.84)
≥3.50[e]	6.66 (5.86-7.46)	8.20 (7.4-9.12)	6.93 (6.29-7.56)	7.73 (6.66-8.80)	9.67 (8.23-11.1)	8.63 (7.40-9.86)	9.13 (7.36-10.9)	9.05 (7.73-10.4)	.001	2.39 (0.84, 3.93)
P for interaction				.10						

Abbreviations: NHANES, National Health and Nutrition Examination Survey.

Food sources were grouped as grocery stores (consumed food items obtained from “grocery or supermarket”), restaurants (“restaurant fast food/pizza, restaurant with waiter/waitress, restaurant no additional information”), schools (“K-12 school cafeteria, childcare center”), worksites (“cafeteria not in a K-12 school, vending machine, common coffee pot or snack tray) and other sources refer to food items obtained from elsewhere (“sport, recreation, or entertainment facility, street vendor, vending truck, from someone else/gift, and others).

[a] All percentages were survey-weighted; %, proportion.

[b] Trends in diet for the other race or mixed race group are not presented in the race/ethnicity-stratified results due to their small sample sizes.

[c] Represents the ratio of family income to the federal poverty threshold, adjusting for household size. For reference, the federal threshold in 2016 for a family of 4 was $24,300/y. A family of 4 earning $33,534/y would have a ratio of 1.38.

[d] Indicates a lower level of income.

© 2021 Liu J et al. *JAMA Network Open.*

[e] Indicates a higher level of income.

© 2021 Liu J et al. *JAMA Network Open.*

eTable 16. Trends in Proportions of US Children Aged 5-19 years with Poor or Intermediate Diet based on American Heart Association Diet Score, by Different Food Sources and by Age, Sex, Race/Ethnicity, Education and Income, NHANES 2003-2018

Grocery	AHA Diet Score, Weighted Mean (95% CI)[a]								P for trend	P for interaction
	2003-2004	2005-2006	2007-2008	2009-2010	2011-2012	2013-2014	2015-2016	2017-2018		
**Poor Diet**										
					**Grocery stores**					
**Age group, y**										
5-11	49.8 (44.5-55.0)	40.8 (32.1-49.5)	44.2 (39.7-48.7)	40.6 (37.2-44.1)	41.4 (36.3-46.6)	39.9 (34.0-45.9)	43.8 (39.6-48.1)	41.7 (36.247.2)	.17	.16
12-19	56.2 (52.5-59.8)	55.4 (44.8-59.8)	50.3 (45.9-55.8)	48.6 (44.3-52.9)	44.9 (40.1-49.7)	49.4 (46.1-52.6)	46.7 (41.1-52.3)	48.0 (42.4-53.7)	.002	
**Sex**										
Female	52.2 (47.9-56.5)	49.3 (42.1-56.6)	42.6 (37.6-47.8)	43.0 (39.3-46.7)	40.6 (34.6-46.7)	41.1 (37.2-44.9)	41.1 (37.0-45.2)	41.0 (34.8-47.5)	.001	.22
Male	54.1 (48.6-59.6)	48.3 (42.7-54.0)	52.6 (46.1-59.0)	46.9 (43.9-49.9)	45.9 (41.2-50.6)	48.7 (43.3-54.1)	49.4 (43.6-55.2)	49.1 (45.0-53.2)	.11	
**Race/ethnicity[b]**										
Non-Hispanic white	54.0 (48-60)	48.5 (41.2-55.8)	47.8 (40.8-54.9)	45.1 (40.4-49.9)	41.1 (34.9-47.3)	48.2 (42.2-54.2)	44.2 (38.1-50.2)	46.1 (39.3-53.0)	.05	.86
Non-Hispanic black	56.8 (53.3-60.3)	60.0 (54.6-65.3)	56.4 (52.3-60.4)	50.3 (45.6-55)	54.1 (49.5-58.8)	51.1 (47.7-54.6)	52.9 (46.0-59.8)	55.5 (48.7-62.1)	.09	
Hispanics	47.2 (40.1-54.3)	41.7 (37.6-46.0)	43.4 (36.9-50.1)	43.7 (37.3-50.1)	41.8 (35.6-48.0)	39.3 (35.3-43.4)	44.0 (39.0-49.1)	42.3 (36.4-48.4)	.42	
**Parental Education**										
<High school graduate	51.9 (44.5-59.3)	52.2 (47-57.4)	51.2 (44.3-58)	44.0 (37.9-50.1)	49.5 (41.5-57.6)	49.1 (43.2-55)	45.7 (38.1-53.3)	42.8 (35.6-50.1)	.04	.88
High school graduate or GED or some college	55.9 (49.6-62.2)	49.8 (44.1-55.4)	51.5 (47-56.1)	49.6 (45.8-53.3)	46.2 (42.2-50.2)	45.5 (41.2-49.8)	48.8 (43.5-54.1)	51.3 (45.9-56.7)	.10	
College graduate or above	45.1 (35.8-54.5)	41.2 (27.8-54.6)	35.8 (28.7-42.9)	36.9 (32-41.9)	32.1 (24.8-39.5)	40.6 (33.1-48)	36.9 (28.7-45.1)	35.2 (26.6-43.7)	.26	
**Ratio of family income to poverty level[c]**										
<1.30 [d]	52.7 (45.8-59.6)	50.2 (44.1-56.4)	49.6 (43.1-56.2)	53.7 (49.2-58.2)	49.9 (46.6-53.3)	46.0 (39.8-52.2)	53.6 (47.3-60)	49.7 (43.4-55.9)	.60	.08
1.30-3.49	55.5 (46.8-64.3)	50.4 (41.5-59.3)	53.2 (46.5-59.9)	45.2 (40.3-50)	44.0 (37.5-50.5)	48.6 (42.9-54.3)	48.3 (42.8-53.7)	48.1 (43-53.2)	.11	
≥3.50	51.0 (44.2-57.8)	45.4 (40.0-50.9)	41.1 (35.0-47.4)	37.7 (33.7-41.9)	33.8 (28.0-40.0)	39.0 (31.3-47.2)	34.2 (26.0-43.6)	37.4 (28.3-47.5)	.003	

© 2021 Liu J et al. *JAMA Network Open.*

Grocery	AHA Diet Score, Weighted Mean (95% CI)[a]								P for trend	P for interaction
	2003-2004	2005-2006	2007-2008	2009-2010	2011-2012	2013-2014	2015-2016	2017-2018		
**Intermediate Diet**										
**Age group, y**										
5-11	50.2 (45.0-55.4)	59.1 (50.4-67.9)	54.5 (49.8-59.2)	58.9 (55.8-61.9)	58.2 (53.1-63.3)	58.7 (52.9-64.6)	55.6 (51.4-59.8)	58.1 (52.6-63.6)	.19	.23
12-19	43.5 (40.2-46.8)	44.4 (39.3-48.3)	49.4 (42.9-54.0)	51.3 (47.1-55.6)	54.2 (49.7-58.7)	50.2 (47.1-53.2)	52.6 (47.1-58.1)	50.6 (45.3-55.8)	.001	
**Sex**										
Female	47.4 (43.5-51.2)	50.5 (43.2-57.8)	56.0 (50.9-61.0)	56.9 (53.2-60.6)	58.4 (52.5-64.4)	58.0 (54.4-61.6)	58.1 (53.9-62.3)	57.5 (51.4-63.3)	.001	.03
Male	45.9 (40.4-51.4)	51.7 (46.0-57.3)	47.3 (40.9-53.8)	52.6 (49.6-55.7)	53.7 (49.0-58.4)	50.5 (45.0-56.0)	50.1 (44.5-55.7)	50.7 (46.7-54.7)	.16	
**Race/ethnicity[b]**										
Non-Hispanic white	45.8 (39.9-51.6)	51.4 (44.1-58.7)	51.3 (44.2-58.4)	54.5 (49.8-59.2)	57.9 (52.0-63.8)	50.6 (45.2-56.1)	55.1 (49.2-61.0)	53.1 (46.4-59.7)	.08	.90
Non-Hispanic black	43.2 (39.7-46.7)	39.9 (34.7-45.3)	43.5 (39.4-47.6)	49.5 (44.8-54.2)	45.9 (41.2-50.5)	48.6 (45.1-52.1)	47.1 (40.2-54.0)	43.7 (37.3-50.3)	.12	
Hispanics	52.4 (45.3-59.6)	58.2 (54.0-62.3)	56.3 (49.7-62.7)	56.0 (49.7-62.3)	57.8 (51.5-64.1)	60.5 (56.5-64.4)	55.2 (50.0-60.4)	57.3 (51.3-63.0)	.48	
**Parental Education**										
<High school graduate	48.1 (40.7-55.4)	47.8 (42.6-53.0)	48.5 (41.8-55.2)	55.7 (49.7-61.8)	50.5 (42.4-58.5)	50.7 (45.0-56.4)	54.0 (46.4-61.6)	56.5 (48.9-64.1)	.06	.92
High school graduate or GED or some college	44.1 (37.8-50.4)	50.1 (44.5-55.7)	48.4 (43.9-53.0)	50.3 (46.6-54.0)	53.7 (49.7-57.6)	53.6 (49.2-57.9)	50.8 (45.5-56.1)	48.7 (43.3-54)	.13	
College graduate or above	53.8 (44.2-63.3)	58.7 (45.3-72.1)	61.3 (53.6-69.0)	62.5 (57.2-67.7)	66.3 (59.3-73.3)	58.3 (51.3-65.2)	61.7 (54.2-69.1)	62.2 (53.3-71.1)	.38	
**Ratio of family income to poverty level[c]**										
<1.30 [d]	47.2 (40.3-54.2)	49.7 (43.6-55.8)	49.3 (43.4-55.3)	46.2 (41.8-50.7)	49.9 (46.6-53.3)	53.6 (47.5-59.8)	46.2 (39.7-52.7)	49.9 (43.8-56.1)	.61	.18
1.30-3.49	44.4 (35.7-53.2)	49.4 (40.4-58.4)	46.3 (39.5-53.1)	54.4 (49.7-59.1)	55.6 (49.3-61.9)	50.4 (45-55.8)	51.1 (45.7-56.5)	51.7 (46.6-56.8)	.13	
≥3.50	48.4 (43.2-53.6)	54.6 (49.1-60.0)	58.1 (51.7-64.2)	62.0 (58.0-65.8)	64.3 (58.6-70.0)	59.7 (52.0-66.6)	64.5 (55.6-72.5)	60.6 (50.6-69.8)	.007	
					**Restaurants**					
**Age group, y**										
5-11	86.4 (82.6-90.2)	76.8 (71.7-82.0)	79.9 (75.9-84)	79.0 (74.7-83.2)	76.4 (72.3-80.5)	78.4 (74.2-82.7)	80.9 (76.0-85.8)	80.7 (75.7-85.7)	.37	.09

© 2021 Liu J et al. *JAMA Network Open.*

Grocery	AHA Diet Score, Weighted Mean (95% CI)[a]								P for trend	P for interaction
	2003-2004	2005-2006	2007-2008	2009-2010	2011-2012	2013-2014	2015-2016	2017-2018		
12-19	83.5 (80.2-86.9)	83.3 (80.3-86.4)	83.8 (78.7-88.9)	79.8 (75.1-84.5)	78.7 (72.6-84.9)	75.7 (72.2-79.3)	76.8 (73.5-80.2)	78.8 (74.3-83.3)	<.001	
**Sex**										
Female	82.1 (78.0-86.1)	76.1 (68.8-83.4)	78.2 (73.9-82.5)	78.7 (72.8-84.6)	77.4 (72.2-82.6)	75.0 (70.7-79.2)	78.0 (74.6-81.4)	77.4 (72.5-82.2)	.30	.13
Male	87.1 (82.9-91.3)	84.7 (80.0-89.3)	86.6 (82.8-90.5)	80.3 (75.4-85.2)	78.3 (73.9-82.6)	78.7 (73.2-84.3)	79.3 (75.6-83.0)	81.9 (78.1-85.8)	.002	
**Race/ethnicity[b]**										
Non-Hispanic white	87.4 (84.4-90.4)	82.4 (77.3-87.5)	82.2 (77.8-86.7)	81.2 (76.3-86)	80.7 (75.5-85.9)	77.1 (72.5-81.8)	78.9 (73.1-84.8)	79.5 (75.1-83.9)	.002	.03
Non-Hispanic black	80.6 (76.3-84.9)	77.8 (72.1-83.6)	82.1 (77.9-86.3)	75.2 (63.8-86.6)	78.2 (73.8-82.6)	78.9 (73.4-84.3)	82.0 (79-85)	80.6 (77.2-84.1)	.65	
Hispanics	79.1 (74.4-83.8)	75.4 (69.1-81.6)	85.1 (81.7-88.5)	79.7 (75.1-84.2)	74.3 (67.2-81.3)	73.5 (68.7-78.3)	78.1 (76.2-80)	79.1 (72.4-85.7)	.46	
**Parental Education**										
<High school graduate	83.5 (79.2-87.9)	80.1 (74.6-85.6)	87.5 (84.8-90.1)	80.6 (73.7-87.4)	80.9 (73.7-88.2)	78.9 (72.5-85.3)	78.3 (72.7-83.9)	74.0 (67.6-80.4)	.006	
High school graduate or GED or some college	86.2 (84-88.4)	81.2 (76.4-85.9)	82.0 (77-87)	81.2 (75.7-86.8)	76.3 (70.3-82.4)	76.1 (71.3-80.9)	82.0 (77.1-86.9)	81.2 (77.3-85.1)	.03	.55
College graduate or above	83.3 (73.9-92.7)	80.6 (73.1-88.1)	78.4 (72-84.8)	77.6 (71.5-83.7)	78.4 (73.2-83.7)	76.5 (71.1-82)	74.8 (68.7-80.9)	79.4 (73.3-85.6)	.27	
**Ratio of family income to poverty level[c]**										
<1.30 [d]	82.9 (78.9-86.8)	83.3 (78.2-88.4)	84.5 (80.7-88.3)	82.2 (76.7-87.6)	79.0 (74-84)	78.0 (74.9-81.2)	81.2 (77.2-85.1)	79.9 (73.4-86.4)	.10	.19
1.30-3.49	87.1 (83.8-90.3)	75.1 (69.9-80.3)	85.3 (80.6-90)	83.0 (79.3-86.7)	79.8 (72.4-87.3)	79.3 (73-85.6)	84.1 (80.1-88.1)	79.7 (75.7-83.8)	.38	
≥3.50	85.0 (78.7-89.7)	85.5 (79.1-90.2)	77.9 (69.5-84.5)	75.8 (69.6-81.1)	75.1 (68.3-80.8)	72.4 (66.9-77.3)	71.3 (63.8-77.8)	81.1 (75.7-85.5)	.004	
**Intermediate Diet**										
**Age group, y**										
5-11	13.6 (9.81-17.4)	23.2 (18.0-28.3)	20.1 (16.0-24.1)	21.0 (16.8-25.3)	23.6 (19.5-27.7)	21.6 (17.3-25.8)	19.1 (14.2-24.0)	19.3 (14.3-24.3)	.37	.09
12-19	16.4 (13.1-19.6)	16.7 (13.6-19.7)	16.2 (11.1-21.3)	20.2 (15.5-24.9)	21.3 (15.1-27.4)	24.3 (20.7-27.8)	23.2 (19.8-26.5)	21.2 (16.7-25.7)	<.001	
**Sex**										

© 2021 Liu J et al. *JAMA Network Open.*

Grocery	AHA Diet Score, Weighted Mean (95% CI)[a]								P for trend	P for interaction
	2003-2004	2005-2006	2007-2008	2009-2010	2011-2012	2013-2014	2015-2016	2017-2018		
Female	17.8 (13.9-21.7)	23.9 (16.6-31.2)	21.8 (17.5-26.1)	21.3 (15.4-27.2)	22.6 (17.4-27.8)	25.0 (20.8-29.3)	22.0 (18.6-25.4)	22.6 (17.8-27.5)	.28	.14
Male	12.9 (8.65-17.1)	15.3 (10.7-20.0)	13.4 (9.54-17.2)	19.7 (14.8-24.6)	21.7 (17.4-26.1)	21.3 (15.7-26.8)	20.7 (17.0-24.4)	18.1 (14.2-21.9)	.002	
**Race/ethnicity[b]**										
Non-Hispanic white	12.5 (9.54-15.5)	17.6 (12.5-22.7)	17.8 (13.3-22.2)	18.8 (14-23.7)	19.3 (14.1-24.5)	22.9 (18.2-27.5)	21.1 (15.2-26.9)	20.5 (16.1-24.9)	.002	.03
Non-Hispanic black	19.4 (15.1-23.7)	22.2 (16.4-27.9)	17.9 (13.7-22.1)	24.8 (13.4-36.2)	21.8 (17.4-26.2)	21.1 (15.7-26.6)	18.0 (15-21)	19.4 (15.9-22.8)	.65	
Hispanics	20.9 (16.2-25.6)	24.6 (18.4-30.9)	14.9 (11.5-18.3)	20.3 (15.8-24.9)	25.7 (18.7-32.8)	26.5 (21.7-31.3)	21.9 (20-23.8)	20.9 (14.3-27.6)	.46	
**Parental Education**										
<High school graduate	16.5 (12.1-20.8)	19.9 (14.4-25.4)	12.5 (9.91-15.2)	19.4 (12.6-26.3)	19.1 (11.8-26.3)	21.1 (14.7-27.5)	21.7 (16.1-27.3)	26 (19.6-32.4)	.006	
High school graduate or GED or some college	13.7 (11.4-16)	18.8 (14.1-23.6)	18 (13-23)	18.8 (13.2-24.3)	23.7 (17.6-29.7)	23.9 (19.1-28.7)	18 (13.1-22.9)	18.8 (14.9-22.7)	.03	.56
College graduate or above	16.7 (7.32-26.1)	19.4 (11.9-26.9)	21.6 (15.2-28)	22.4 (16.3-28.5)	21.6 (16.3-26.8)	23.5 (18-28.9)	25.2 (19.1-31.3)	20.6 (14.4-26.7)	.27	
**Ratio of family income to poverty level[c]**										
<1.30 [d]	16.9 (13-20.8)	16.7 (11.6-21.8)	15.5 (11.7-19.3)	17.8 (12.4-23.3)	21.0 (16.0-26.0)	22.0 (18.8-25.1)	18.8 (14.9-22.8)	20.1 (13.6-26.6)	.09	.19
1.30-3.49	12.9 (9.67-16.2)	24.9 (19.7-30.1)	14.7 (9.98-19.4)	17.0 (13.3-20.7)	20.2 (12.7-27.6)	20.7 (14.4-27)	15.9 (11.9-19.9)	20.3 (16.2-24.3)	.39	
≥3.50	15.0 (10.3-21.3)	14.5 (9.81-20.9)	22.1 (15.5-30.5)	24.2 (18.9-30.4)	24.9 (19.2-31.7)	27.6 (22.7-33.1)	28.7 (22.2-36.2)	18.9 (14.5-24.3)	.004	
**School**										
**Age group, y**										
5-11	50.5 (43.4-57.5)	54.7 (47.1-62.3)	53.4 (47.6-59.1)	47.9 (41.2-54.6)	43.6 (35.2-51.9)	30.2 (26.4-34.0)	32.0 (28.1-35.9)	21.8 (16.8-26.9)	<.001	.003
12-19	62.6 (53.8-71.3)	66.0 (60.0-72.0)	68.0 (62.5-73.5)	64.2 (54.7-73.7)	52.6 (43.4-61.8)	34.9 (28.5-41.2)	27.4 (21.8-33.0)	27.5 (23.4-31.7)	<.001	
**Sex**										
Female	51.0 (43.8-58.1)	54.7 (49-60.5)	58.6 (52.7-64.6)	54.6 (48.2-61)	45.0 (33.7-56.3)	31.3 (27.1-35.5)	29.5 (24.3-34.6)	22.5 (17.5-27.4)	<.001	.13
Male	60.3 (52.8-67.7)	63.8 (54.7-72.8)	60.9 (55.2-66.5)	55.2 (45.7-64.6)	49.6 (44.5-54.6)	32.9 (27.2-38.5)	30.4 (23.8-37)	25.9 (20.2-31.6)	<.001	

© 2021 Liu J et al. *JAMA Network Open.*

Grocery	AHA Diet Score, Weighted Mean (95% CI)[a]								P for trend	P for interaction
	2003-2004	2005-2006	2007-2008	2009-2010	2011-2012	2013-2014	2015-2016	2017-2018		
**Race/ethnicity[b]**										
Non-Hispanic white	54.4 (44.5-64.2)	55.4 (46.4-64.4)	65.6 (58.7-72.5)	59.0 (50.4-67.6)	54.1 (42.4-65.8)	36.8 (29.0-44.7)	33.8 (26.3-41.2)	27.0 (21.7-32.3)	<.001	.12
Non-Hispanic black	53.1 (46.7-59.6)	62.0 (51.9-72)	48.3 (41.3-55.4)	45.9 (37.3-54.5)	42.9 (34.1-51.6)	25.5 (18.0-33.0)	23.4 (17.5-29.3)	23.9 (17.1-30.7)	<.001	
Hispanics	54.9 (49.8-59.9)	58.6 (51.3-66)	58.8 (53.5-64.1)	47.4 (40.1-54.7)	38.8 (31.5-46.1)	31.4 (26.7-36)	28.2 (22.8-33.6)	21.1 (11.1-31.2)	<.001	
**Parental Education**										
<High school graduate	58.0 (49.2-66.9)	51.9 (43.4-60.5)	53.4 (48.0-58.8)	50.2 (42.1-58.2)	38.1 (29.4-46.8)	31.1 (21.7-40.6)	30.0 (24.6-35.3)	23.8 (15.6-32)	<.001	.39
High school graduate or GED or some college	52.0 (45.1-58.8)	62.8 (55.9-69.7)	62.9 (55.7-70)	53.5 (48.4-58.7)	52.3 (43.7-60.8)	34.0 (29-38.9)	28.5 (22.1-34.9)	27.6 (24.4-30.8)	<.001	
College graduate or above	61.5 (44.8-78.1)	57.4 (50.9-64)	61.6 (55.6-67.6)	63.1 (51.2-75)	47.2 (37.7-56.7)	29.6 (21.5-37.8)	33.2 (24.1-42.4)	18.7 (5.72-31.8)	<.001	
**Ratio of family income to poverty level[c]**										
<1.30 [d]	54.2 (45.6-62.8)	54.7 (44.8-64.5)	54.0 (45.9-62.1)	51.5 (42.3-60.7)	43.1 (36.3-50.0)	30.6 (26.2-35)	30.5 (22.7-38.3)	28.6 (24.2-32.9)	<.001	.18
1.30-3.49	58.4 (50.7-66.1)	62.0 (54.8-69.2)	62.4 (53.4-71.5)	55.0 (44.9-65.1)	54.7 (44.2-65.2)	33.8 (25.8-41.8)	27.0 (20.9-33.1)	21.7 (17.3-26.1)	<.001	
≥3.50	52.3 (40.6-64.0)	59.7 (48.7-70.6)	65.4 (59.2-71.6)	56.7 (42.9-70.5)	41.8 (24.7-58.9)	33.4 (22.9-43.9)	33.0 (20.4-45.5)	20.6 (10.5-30.7)	<.001	
**Intermediate Diet**										
**Age group, y**										
5-11	49.5 (42.5-56.6)	45.2 (37.5-52.8)	46.4 (40.7-52.1)	52.1 (45.4-58.8)	56.3 (48-64.6)	69.7 (65.8-73.6)	67.6 (63.8-71.4)	77.1 (72.2-82.0)	<.001	.002
12-19	37.2 (28.4-46.1)	34.0 (28-40)	32.0 (26.5-37.5)	35.8 (26.3-45.3)	47.2 (37.9-56.5)	64.7 (58-71.3)	72.6 (67-78.2)	71.9 (67.9-75.8)	<.001	
**Sex**										
Female	49.0 (41.9-56.2)	45.1 (39.4-50.9)	41.1 (35.2-47)	45.4 (39.0-51.8)	54.9 (43.7-66.2)	68.2 (63.9-72.5)	70.5 (65.4-75.7)	76.2 (69.9-82.5)	<.001	.13
Male	39.6 (32,0-47.1)	36.2 (27.2-45.3)	39.1 (33.5-44.8)	44.8 (35.4-54.3)	50.1 (45.1-55.1)	67.0 (61.4-72.6)	69.2 (62.5-75.8)	73.6 (68-79.3)	<.001	
**Race/ethnicity[b]**										
Non-Hispanic white	45.5 (35.6-55.3)	44.6 (35.6-53.6)	34.4 (27.5-41.3)	41.0 (32.4-49.6)	45.9 (34.2-57.6)	63.2 (55.3-71)	66.2 (58.8-73.7)	73.0 (67.7-78.3)	<.001	.13
Non-Hispanic black	46.9	37.7	51.7	54.1	57.0	74.4	75.7	76.1	<.001	

© 2021 Liu J et al. *JAMA Network Open.*

Grocery	AHA Diet Score, Weighted Mean (95% CI)[a]								P for trend	P for interaction
	2003-2004	2005-2006	2007-2008	2009-2010	2011-2012	2013-2014	2015-2016	2017-2018		
	(40.4-53.3)	(27.7-47.8)	(44.6-58.7)	(45.5-62.7)	(48.4-65.6)	(66.9-81.9)	(69.6-81.8)	(69.3-82.9)		
Hispanics	45.1 (40.1-50.2)	41.4 (34.0-48.7)	40.7 (35.3-46.1)	52.6 (45.3-59.9)	60.7 (53.5-67.8)	68.1 (63.6-72.6)	71.5 (66.1-76.8)	76.3 (66.2-86.3)	<.001	
**Education**										
<High school graduate	42.0 (33.1-50.8)	48.1 (39.5-56.6)	46.0 (40.5-51.5)	49.8 (41.8-57.9)	61.7 (53-70.3)	68.4 (59-77.7)	69.8 (64.4-75.1)	73.4 (65.8-81)	<.001	.35
High school graduate or GED or some college	47.9 (41-54.8)	37.1 (30.1-44)	37.1 (30-44.3)	46.5 (41.3-51.6)	47.6 (38.9-56.2)	65.7 (60.6-70.8)	71.2 (64.7-77.6)	72.0 (68.6-75.4)	<.001	
College graduate or above	38.5 (21.9-55.2)	42.6 (36-49.1)	38.4 (32.4-44.4)	36.9 (25-48.8)	52.8 (43.3-62.3)	70.4 (62.2-78.5)	66.8 (57.6-75.9)	81.3 (68.2-94.3)	<.001	
**Ratio of family income to poverty level[c]**										
<1.30 [d]	45.6 (36.9-54.4)	45.1 (35.4-54.9)	46.0 (37.9-54.1)	48.5 (39.3-57.7)	56.5 (49.7-63.4)	68.9 (64.4-73.4)	69.3 (61.5-77.2)	69.7 (65.0-74.4)	<.001	.15
1.30-3.49	41.6 (33.9-49.3)	38.0 (30.8-45.2)	37.1 (28.1-46.1)	45.0 (34.9-55.1)	45.3 (34.8-55.8)	66.1 (58.2-73.9)	72.9 (66.9-78.9)	77.6 (72.8-82.5)	<.001	
≥3.50	47.7 (36.0-59.4)	40.3 (29.4-51.3)	34.6 (28.4-40.8)	43.3 (29.5-57.1)	58.2 (41.1-75.3)	66.5 (56.0-77.0)	67.1 (54.5-79.6)	79.4 (69.3-89.5)	<.001	
Others										
**Age group, y**										
5-11	36.1 (25.7-46.5)	44.8 (37.9-51.8)	49.1 (40.8-57.4)	45.4 (42.5-48.4)	45.6 (37.9-53.3)	48.4 (41.2-55.7)	47.4 (40.7-54)	46.1 (38.1-54.2)	.18	.22
12-19	43.8 (40.8-46.9)	46.0 (40.5-51.5)	47.5 (40.0-54.9)	51.7 (45.9-57.4)	50.7 (45.0-56.4)	53.0 (46.4-59.5)	56.7 (51.2-62.2)	56.8 (50.8-62.8)	<.001	
**Sex**										
Female	39.2 (33.5-44.9)	47.6 (42.9-52.3)	45.8 (37.5-54.2)	44.9 (40.7-49.1)	45.2 (40.1-50.4)	49.8 (42.9-56.6)	51.3 (44.6-58)	48.7 (44.8-52.6)	.01	.33
Male	41.0 (34.0-47.9)	43.3 (36.0-50.5)	50.8 (45.7-55.8)	52.5 (49.4-55.6)	51.6 (46-57.2)	51.8 (45.4-58.3)	53.3 (48.3-58.3)	55.0 (47.3-62.6)	.001	
**Race/ethnicity[b]**										
Non-Hispanic white	40.7 (33.5-47.8)	46.0 (39.4-52.7)	48.2 (40.4-56.0)	50.3 (46.5-54.1)	49.2 (43.2-55.1)	56.4 (48.0-64.8)	55.7 (48.0-63.4)	54.3 (47.1-61.5)	.001	.04
Non-Hispanic black	39.8 (33.2-46.5)	45.2 (39.4-51)	47.0 (40.6-53.3)	39.3 (29.2-49.4)	50.1 (38.7-61.5)	48.3 (40.6-55.9)	50.2 (41.4-59.0)	55.7 (48.7-62.6)	.002	
Hispanics	42.0 (35.2-48.7)	46.3 (36.5-56.1)	47.1 (41.4-52.9)	49.7 (42.3-57.1)	50.1 (43.4-56.9)	45.0 (39.4-50.6)	47.7 (41.2-54.3)	43.9 (38.1-49.7)	.98	
**Parental Education**										

© 2021 Liu J et al. *JAMA Network Open.*

Grocery	AHA Diet Score, Weighted Mean (95% CI)[a]								P for trend	P for interaction
	2003-2004	2005-2006	2007-2008	2009-2010	2011-2012	2013-2014	2015-2016	2017-2018		
<High school graduate	39.4 (34.2-44.5)	52.0 (40.0-63.9)	48.1 (40.5-55.7)	48.7 (43.7-53.6)	48.6 (39.6-57.5)	40.9 (29.5-52.2)	44.7 (36.3-53)	41.4 (29.8-52.9)	.48	.12
High school graduate or GED or some college	40.2 (33.3-47.2)	44.0 (37.0-51.0)	48.8 (40.2-57.5)	46.4 (38.8-54.1)	51.0 (46.0-56.0)	50.5 (43.6-57.5)	54.7 (50.3-59.0)	55.1 (51.9-58.3)	<.001	
College graduate or above	38.1 (26.7-49.4)	44.6 (33.8-55.4)	47.7 (34.2-61.3)	51.0 (42.3-59.8)	44.6 (37.2-52.0)	55.3 (45.5-65.1)	53.0 (42.6-63.5)	48.3 (39.2-57.4)	.12	
**Ratio of family income to poverty level[c]**										
<1.30 [d]	39.1 (33.4-44.9)	42.1 (35-49.2)	42.6 (33.2-52.1)	51.1 (42.5-59.7)	48.2 (40.5-55.9)	46.4 (36.2-56.5)	48.7 (40.6-56.8)	46.3 (38.1-54.4)	.08	.36
1.30-3.49	32.7 (24.6-40.8)	49.6 (42.6-56.6)	51.2 (43.8-58.6)	47.2 (40.3-54)	47.0 (39.0-55.1)	51.6 (42.8-60.5)	51.9 (44.6-59.3)	56.4 (50.2-62.5)	.001	
≥3.50	48.4 (38.0-58.9)	43.3 (36.4-50.3)	48.3 (39.9-56.6)	47.9 (39.8-55.9)	50.8 (39.6-62.1)	55.8 (43.8-67.9)	55.0 (44.1-65.9)	52.6 (42.4-62.8)	.09	
**Intermediate Diet**										
**Age group, y**										
5-11	63.9 (53.5-74.3)	54.9 (47.8-62.1)	50.9 (42.6-59.2)	54.6 (51.6-57.5)	54.4 (46.7-62.1)	51.6 (44.3-58.8)	52.6 (46-59.3)	53.8 (45.7-61.8)	.18	.24
12-19	55.6 (52.2-59)	54.0 (48.5-59.5)	52.5 (45.1-60)	48.3 (42.6-54.1)	49.3 (43.6-55)	47.0 (40.5-53.6)	43.2 (37.7-48.6)	43.1 (37.1-49.1)	<.001	
**Sex**										
Female	60.3 (54.4-66.2)	52.2 (47.3-57.1)	54.2 (45.8-62.5)	55.1 (50.9-59.3)	54.8 (49.6-59.9)	50.2 (43.4-57.1)	48.5 (41.9-55.2)	51.2 (47.3-55)	.01	.31
Male	59.0 (52.1-66.0)	56.7 (49.5-64.0)	49.2 (44.2-54.3)	47.5 (44.4-50.6)	48.4 (42.8-54.0)	48.2 (41.7-54.6)	46.7 (41.7-51.7)	45.0 (37.4-52.7)	.001	
**Race/ethnicity[b]**										
Non-Hispanic white	58.9 (51.6-66.3)	54.0 (47.3-60.6)	51.8 (44.0-59.6)	49.7 (45.9-53.5)	50.8 (44.9-56.8)	43.6 (35.2-52)	44.2 (36.4-51.9)	45.7 (38.5-52.9)	.001	.04
Non-Hispanic black	60.2 (53.5-66.8)	54.8 (49.0-60.6)	53.0 (46.7-59.4)	60.7 (50.6-70.8)	49.9 (38.5-61.3)	51.7 (44.1-59.4)	49.8 (41.0-58.6)	44.3 (37.4-51.3)	.002	
Hispanics	58.0 (51.3-64.8)	52.8 (42.8-62.8)	52.9 (47.1-58.6)	50.3 (42.9-57.7)	49.9 (43.1-56.6)	55.0 (49.4-60.6)	52.3 (45.7-58.8)	55.9 (50.2-61.6)	.98	
**Parental Education**										
<High school graduate	60.6 (55.5-65.8)	48.0 (36.1-60.0)	51.9 (44.3-59.5)	51.3 (46.4-56.3)	51.4 (42.5-60.4)	59.1 (47.8-70.5)	55.3 (47.0-63.7)	58.3 (46.9-69.7)	.49	.12
High school graduate or GED or some college	59.3 (52.3-66.3)	55.8 (48.9-62.7)	51.2 (42.5-59.8)	53.6 (45.9-61.2)	49.0 (44.0-53.9)	49.5 (42.5-56.4)	45.3 (41.0-49.7)	44.9 (41.7-48.0)	<.001	

© 2021 Liu J et al. *JAMA Network Open.*

Grocery	AHA Diet Score, Weighted Mean (95% CI)[a]								P for trend	P for interaction
	2003-2004	2005-2006	2007-2008	2009-2010	2011-2012	2013-2014	2015-2016	2017-2018		
College graduate or above	61.9 (50.6-73.3)	55.4 (44.6-66.2)	52.3 (38.7-65.8)	49.0 (40.2-57.7)	55.4 (48-62.8)	44.7 (34.9-54.5)	46.7 (36.3-57.1)	51.7 (42.6-60.8)	.12	
**Ratio of family income to poverty level**[c]										
<1.30 [d]	60.9 (55.1-66.6)	57.4 (50.1-64.7)	57.4 (47.9-66.8)	48.9 (40.3-57.5)	51.7 (44.0-59.5)	53.6 (43.5-63.8)	51.3 (43.2-59.4)	53.6 (45.5-61.6)	.09	
1.30-3.49	66.5 (58.1-75.0)	50.4 (43.4-57.4)	48.8 (41.4-56.2)	52.8 (46.0-59.7)	53.0 (44.9-61.0)	48.4 (39.5-57.2)	48.1 (40.7-55.4)	43.5 (37.4-49.6)	.001	.39
≥3.50	52.0 (41.3-62.7)	57.1 (50.0-64.3)	50.2 (42.2-58.1)	52.2 (44.5-59.9)	49.8 (40.9-58.8)	45.6 (33.9-57.2)	44.2 (35.4-53.1)	48.6 (41.2-56)	.08	

Abbreviations: NHANES, National Health and Nutrition Examination Survey.
Food sources were grouped as grocery stores (consumed food items obtained from “grocery or supermarket”), restaurants (“restaurant fast food/pizza, restaurant with waiter/waitress, restaurant no additional information”), schools (“K-12 school cafeteria, childcare center”), worksites (“cafeteria not in a K-12 school, vending machine, common coffee pot or snack tray) and other sources refer to food items obtained from elsewhere (“sport, recreation, or entertainment facility, street vendor, vending truck, from someone else/gift, and others).

[a] All percentages were survey-weighted; %, proportion.

[b] Trends in diet for the other race or mixed race group are not presented in the race/ethnicity-stratified results due to their small sample sizes. [c]Represents the ratio of family income to the federal poverty threshold, adjusting for household size. For reference, the federal threshold in 2016 for a family of 4 was $24,300/y. A family of 4 earning $33,534/y would have a ratio of 1.38.

[d] Indicates a lower level of income.

[e] Indicates a higher level of income.

© 2021 Liu J et al. *JAMA Network Open.*

eTable 17. Trends in American Heart Association Diet Score for Meals Consumed from Different Sources by Age Group, Sex, Race/Ethnicity, Parental Education, and Income among American Children aged 5-19 years, NHANES 2003-2018

	AHA Diet Score, Weighted Mean (95% CI)[a]								P for trend	2017-2018 versus 2003-2004, difference (95% CI)
	2003-2004	2005-2006	2007-2008	2009-2010	2011-2012	2013-2014	2015-2016	2017-2018		
				Grocery stores						
**Age group, y**										
5-11	32.3 (31.1-33.5)	34.3 (32.1-36.5)	33.4 (32.1-34.7)	35.1 (34.1-36.1)	35.7 (34.1-37.3)	35.7 (34.3-37.1)	34.7 (33.6-35.8)	35.2 (33.7-36.8)	.003	2.90 (0.97, 4.83)
12-19	30.2 (29.3-31.0)	31.4 (30.0-32.7)	32.3 (31.0-33.7)	32.6 (31.8-33.3)	34.1 (32.7-35.6)	32.8 (31.9-33.8)	33.4 (32.1-34.8)	33.5 (31.8-35.2)	<.001	3.33 (1.39, 5.27)
P for interaction				.45						
**Sex**										
Female	31.7 (30.7-32.8)	33.2 (31.6-34.9)	34.2 (32.3-36.0)	34.4 (33.7-35.1)	35.5 (33.9-37.2)	35.0 (34.1-36.0)	35.0 (33.9-36)	35.7 (33.8-37.5)	.002	3.93 (1.80, 6.07)
Male	30.6 (29.8-31.5)	32.2 (30.6-33.8)	31.4 (30.0-32.9)	33.0 (32.3-33.7)	34.2 (32.9-35.4)	33.3 (31.8-34.8)	33.1 (31.7-34.5)	32.9 (31.7-34.1)	<.001	2.33 (0.85, 3.80)
P for interaction				.17						
**Race/ethnicity[b]**										
Non-Hispanic white	31.0 (29.7-32.4)	32.9 (30.9-35)	32.9 (31.1-34.8)	33.8 (32.7-34.8)	35.7 (33.9-37.4)	33.8 (32.1-35.5)	34.5 (33.0-36.0)	33.8 (31.3-36.3)	.008	2.77 (-0.03, 5.58)
Non-Hispanic black	29.9 (29.1-30.7)	29.8 (28.9-30.7)	30.5 (29.2-31.7)	32.0 (31.2-32.8)	31.5 (30.2-32.7)	32.1 (31.0-33.2)	31.3 (29.6-33)	31.2 (29.5-33)	.01	1.35 (-0.54, 3.24)
Hispanics	32.0 (30.5-33.6)	34.0 (32.8-35.1)	33.3 (31.8-34.8)	34.1 (32.1-36)	34.9 (33.4-36.5)	35.0 (34.0-36.0)	34.1 (32.6-35.6)	35.3 (33.9-36.8)	.006	3.28 (1.14, 5.43)
P for interaction				.69						
**Parental Education**										
<High school graduate	31.0 (29.1-32.9)	31.8 (30.2-33.5)	31.3 (29.5-33.1)	33.3 (32.0-34.7)	32.3 (30.5-34.1)	32.9 (31.3-34.5)	33.1 (30.9-35.2)	34.3 (32.6-36)	.009	3.31 (0.76, 5.86)
High school graduate or GED or some college	30.1 (29.1-31.2)	32.5 (31.1-33.9)	31.6 (30.7-32.5)	32.7 (31.7-33.7)	33.7 (32.5-34.8)	33.6 (32.2-34.9)	32.9 (31.8-34)	32.6 (30.8-34.4)	.004	2.46 (0.39, 4.53)
College graduate or above	34.5 (32.0-37.0)	34.4 (31.0-37.7)	36.9 (35-38.9)	35.9 (34.8-37.0)	39.3 (37.1-41.6)	36.3 (34.3-38.4)	37.1 (35.4-38.8)	37.2 (35.2-39.3)	.04	2.76 (-0.49, 6.01)
P for interaction				.91						
**Ratio of family income to poverty level[c]**										
<1.30 [d]	31.4 (29.7-33.1)	32.2 (30.5-33.8)	32.2 (30.3-34.1)	31.5 (30.4-32.6)	32.6 (31.9-33.4)	32.8 (30.8-34.7)	30.9 (29.4-32.5)	33.1 (31.1-35.2)	.40	1.76 (-0.89, 4.42)

© 2021 Liu J et al. *JAMA Network Open.*

	AHA Diet Score, Weighted Mean (95% CI)[a]								P for trend	2017-2018 versus 2003-2004, difference (95% CI)
	2003-2004	2005-2006	2007-2008	2009-2010	2011-2012	2013-2014	2015-2016	2017-2018		
1.30-3.49	30.3 (28.6-32.0)	32.6 (30.3-34.9)	31.0 (29.9-32.2)	33.7 (32.8-34.6)	34.1 (32.1-36.0)	34.0 (32.6-35.3)	33.7 (32.3-35.1)	33.4 (32.1-34.6)	.001	3.09 (0.95, 5.22)
≥3.50 [e]	32.0 (30.5-33.6)	33.3 (31.6-34.9)	34.8 (32.6-36.9)	35.4 (34.6-36.2)	38.9 (36.8-40.9)	36.1 (34.1-38.0)	37.3 (35.4-39.2)	36.5 (34.1-39.0)	<.001	4. 51 (1.64, 7.38)
P for interaction				.009						
					**Restaurants**					
**Age group, y**										
5-11	24.8 (24.0-25.6)	27.2 (26.3-28.2)	26.1 (25.3-27.0)	26.3 (25.3-27.4)	26.4 (25.3-27.5)	26.0 (25.2-26.8)	25.9 (24.5-27.4)	26.3 (25.4-27.3)	.53	1.53 (0.33, 2.73)
12-19	24.7 (23.8-25.5)	25.2 (24.6-25.8)	24.5 (23.4-25.6)	26.5 (25.4-27.6)	26.3 (25.4-27.3)	26.1 (25.3-26.9)	25.6 (24.8-26.4)	26.0 (24.8-27.1)	.01	1.31 (-0.09, 2.70)
P for interaction				.15						
**Sex**										
Female	25.2 (24.4-26.0)	27.3 (26.0-28.6)	26.0 (25.1-27.0)	26.7 (25.6-27.8)	26.8 (26.0-27.6)	26.7 (25.7-27.7)	25.8 (24.7-26.9)	27.1 (25.7-28.4)	.26	1.89 (0.29, 3.48)
Male	24.3 (23.5-25.1)	25.0 (24.4-25.6)	24.3 (23.6-25.0)	26.2 (25.1-27.3)	25.8 (24.9-26.7)	25.5 (24.1-26.8)	25.7 (24.8-26.6)	25.1 (24.1-26.1)	.04	0.81 (-0.51, 2.12)
P for interaction				.69						
**Race/ethnicity[b]**										
Non-Hispanic white	24.3 (23.4-25.1)	25.7 (24.8-26.6)	25.2 (24.3-26.0)	26.5 (25.3-27.8)	25.8 (24.7-26.9)	25.8 (24.5-27)	25.4 (23.9-26.9)	26.6 (25.2-27.9)	.03	2.30 (0.71, 3.88)
Non-Hispanic black	25.4 (24.4-26.4)	26.7 (25.8-27.5)	25.2 (24.3-26.2)	26.6 (24.8-28.4)	26.4 (25.3-27.6)	26.0 (24.7-27.2)	25.3 (24.2-26.4)	25.0 (24.0-26.1)	.30	-0.39 (-1.80, 1.05)
Hispanics	25.8 (24.7-27)	26.7 (25.5-27.8)	25.2 (24.3-26)	25.6 (24.7-26.6)	27.1 (25.7-28.5)	26.7 (25.9-27.5)	26.0 (25.2-26.7)	26.1 (24.6-27.6)	.59	0.26 (-1.60, 2.12)
P for interaction				.09						
**Parental Education**										
<High school graduate	25.4 (24.4-26.3)	25.7 (24.3-27)	24.1 (23.1-25)	25.8 (24.5-27)	25.7 (24.5-26.9)	25.4 (24.1-26.8)	25.8 (24.7-27)	26.9 (25.2-28.6)	.08	1.52 (-0.40, 3.43)
High school graduate or GED or some college	24.5 (23.9-25.1)	25.8 (25-26.5)	25.2 (24.2-26.3)	26.2 (25-27.3)	26.5 (25.4-27.5)	25.9 (24.8-27)	25.0 (23.9-26.1)	25.6 (24.5-26.7)	.25	1.09 (-0.14, 2.33)
College graduate or above	24.7 (23-26.4)	26.8 (25.7-27.9)	25.9 (24.6-27.1)	26.9 (25.2-28.7)	26.5 (24.9-28.1)	26.8 (25.7-27.9)	26.9 (25.4-28.4)	26.8 (25.4-28.2)	.13	2.05 (-0.18, 4.28)
P for interaction				.59						
**Ratio of family income to poverty level[c]**										

© 2021 Liu J et al. *JAMA Network Open.*

	AHA Diet Score, Weighted Mean (95% CI)[a]								P for trend	2017-2018 versus 2003-2004, difference (95% CI)
	2003-2004	2005-2006	2007-2008	2009-2010	2011-2012	2013-2014	2015-2016	2017-2018		
<1.30 [d]	25.7 (24.8-26.7)	26.1 (25.1-27.1)	24.6 (23.8-25.4)	25.5 (24.5-26.6)	25.9 (24.9-26.9)	26.1 (25.4-26.9)	25.2 (23.9-26.6)	25.1 (23.7-26.5)	.67	-0.63 (-2.30, 1.05)
1.30-3.49	23.8 (22.9-24.8)	26.4 (25.5-27.3)	24.9 (23.8-26.1)	25.9 (24.8-27)	25.2 (24.0-26.4)	25.1 (23.5-26.6)	25.0 (23.7-26.3)	26.2 (25.0-27.3)	.22	2.35 (0.85, 3.85)
≥3.50 [e]	24.8 (23.8-25.9)	25.5 (24.4-26.7)	26.1 (24.9-27.3)	27.0 (25.5-28.5)	27.8 (26.6-29.0)	27.4 (26.7-28.2)	27.0 (25.4-28.5)	26.9 (25.7-28.0)	.001	2.05 (0.51, 3.58)
P for interaction				.02						
				**Schools**						
**Age group, y**										
5-11	32.5 (31.3-33.7)	31.5 (30.0-32.9)	31.6 (30.2-33.0)	33.4 (32.3-34.4)	34.3 (32.8-35.8)	37.8 (37.0-38.7)	37.5 (36.5-38.6)	40.3 (39.1-41.6)	<.001	7.82 (6.09, 9.55)
12-19	29.7 (28.2-31.2)	29.3 (28.2-30.4)	28.8 (27.9-29.8)	29.6 (27.6-31.7)	32.6 (30.5-34.7)	35.9 (34.7-37.1)	38.6 (37.2-40)	38.5 (37.5-39.5)	<.001	8.83 (7.03, 10.6)
P for interaction				.003						
**Sex**										
Female	32.1 (30.5-33.7)	31.4 (30.1-32.8)	31.0 (29.6-32.3)	32.0 (30.5-33.5)	34.1 (32.2-36.1)	37.6 (36.2-38.9)	37.7 (36.6-38.8)	40.3 (39.0-41.6)	<.001	8.19 (6.12, 10.3)
Male	30.5 (29.3-31.8)	29.8 (28.1-31.4)	29.8 (28.7-31.0)	31.6 (30.0-33.1)	33.0 (31.6-34.4)	36.5 (35.5-37.6)	38.3 (37.0-39.6)	38.9 (37.9-39.9)	<.001	8.37 (6.78, 9.97)
P for interaction				.06						
**Race/ethnicity[b]**										
Non-Hispanic white	31.6 (29.8-33.3)	31.2 (29.7-32.6)	29.1 (27.3-30.9)	31.3 (29.2-33.4)	32.2 (30.1-34.4)	35.9 (34.3-37.6)	37.0 (35.4-38.5)	38.1 (36.7-39.5)	<.001	6.55 (4.31, 8.80)
Non-Hispanic black	31.5 (30.2-32.9)	30.2 (27.9-32.6)	32.3 (30.6-33.9)	33.0 (31.3-34.7)	33.4 (32.0-34.9)	37.9 (36.4-39.4)	38.4 (36.6-40.3)	39.1 (37.5-40.7)	<.001	7.54 (5.42, 9.65)
Hispanics	31.4 (30.1-32.7)	30.9 (29.4-32.5)	31.1 (29.8-32.3)	32.7 (31.3-34.2)	35.8 (34.3-37.3)	37.7 (36.8-38.6)	39.5 (38.4-40.5)	41.4 (39.3-43.5)	<.001	10.0 (7.55, 12.4)
P for interaction				.01						
**Parental Education**										
<High school graduate	30.4 (28.8-31.9)	31.7 (30.2-33.3)	32.0 (31.1-33.0)	32.7 (30.9-34.5)	35.4 (33.8-36.9)	37.9 (35.8-39.9)	38.3 (37.1-39.4)	40.4 (38-42.7)	<.001	10.0 (7.21, 12.8)
High school graduate or GED or some college	31.8 (30.7-33.0)	30.0 (28.6-31.4)	29.4 (27.9-30.8)	32.2 (31.0-33.4)	32.6 (31.0-34.3)	36.3 (35.4-37.3)	38.0 (36.8-39.1)	39.2 (38.6-39.8)	<.001	7.40 (6.11, 8.69)
College graduate or above	31.2 (27.8-34.7)	30.4 (28.3-32.5)	30.6 (29.1-32.2)	29.9 (27.9-31.9)	33.5 (31.3-35.7)	37.7 (35.7-39.6)	37.8 (35.4-40.1)	39.2 (37.2-41.1)	<.001	7.94 (4.0, 11.9)
P for interaction				.71						

© 2021 Liu J et al. *JAMA Network Open.*

	AHA Diet Score, Weighted Mean (95% CI)[a]								P for trend	2017-2018 versus 2003-2004, difference (95% CI)
	2003-2004	2005-2006	2007-2008	2009-2010	2011-2012	2013-2014	2015-2016	2017-2018		
**Ratio of family income to poverty level[c]**										
<1.30 [d]	32.1 (30.5-33.7)	31.1 (29.1-33.2)	31.3 (29.7-32.8)	32.2 (30.3-34.2)	34.5 (33.0-36.0)	37.5 (36.4-38.6)	37.8 (36.3-39.3)	39.7 (38.1-41.3)	<.001	7.58 (5.31, 9.85)
1.30-3.49	30.7 (29.7-31.8)	30.3 (28.6-31.9)	29.7 (28.1-31.3)	31.8 (29.7-34.0)	32.3 (30.0-34.7)	36.3 (34.8-37.7)	38.6 (37.7-39.5)	39.9 (38.8-40.9)	<.001	9.12 (7.60, 10.6)
≥3.50 [e]	31.1 (28.6-33.6)	30.5 (28.3-32.8)	29.7 (28.1-31.3)	31.2 (28.9-33.5)	33.9 (31.9-36)	36.9 (33.6-40.3)	37.5 (34.8-40.1)	38.9 (37.0-40.9)	<.001	7.81 (4.63, 11.0)
P for interaction				.40						
				**Others**						
**Age group, y**										
5-11	35.4 (33.9-37.0)	34.2 (32.5-35.9)	33.2 (31.5-35.0)	34.0 (33.3-34.6)	33.7 (31.3-36.1)	32.6 (31.4-33.7)	33.8 (32.8-34.9)	34.1 (32.1-36.1)	.29	-1.30 (-3.90, 1.21)
12-19	34.6 (33.9-35.3)	33.0 (32.1-33.9)	33.5 (31.9-35.0)	32.7 (31.2-34.1)	31.8 (30.5-33.1)	32.1 (31.0-33.3)	32.0 (30.8-33.2)	32.1 (30.9-33.3)	<.001	-2.50 (-3.90, -1.10)
P for interaction				.24						
**Sex**										
Female	35.6 (34.4-36.7)	33.3 (31.9-34.7)	34.0 (32.5-35.5)	34.5 (33.5-35.4)	33.4 (31.9-34.9)	32.7 (31.5-33.8)	33.4 (32.3-34.5)	33.9 (32.4-35.4)	.08	-1.70 (-3.60, 0.20)
Male	34.4 (33.3-35.5)	33.9 (32.1-35.8)	32.7 (31.7-33.8)	32.1 (31.5-32.6)	31.9 (30.5-33.4)	32.0 (30.8-33.2)	32.3 (31.4-33.2)	32.2 (30.6-33.7)	.007	-2.20 (-4.10, -0.28)
P for interaction				.43						
**Race/ethnicity[b]**										
Non-Hispanic white	34.9 (33.9-35.9)	33.3 (31.6-35.1)	33.0 (31.2-34.7)	33.0 (32.1-34.0)	31.9 (30.3-33.5)	31.0 (29.6-32.3)	32.1 (31.0-33.2)	32.5 (30.2-34.8)	.008	-2.40 (-5.0, -0.12)
Non-Hispanic black	34.5 (33.2-35.9)	34.0 (32.5-35.4)	34.1 (32.6-35.6)	33.8 (31.4-36.2)	32.6 (30.6-34.6)	33.6 (32.3-35.0)	34.4 (33.0-35.7)	32.2 (30.6-33.7)	.09	-2.4 (-4.50, -0.31)
Hispanics	34.8 (33.5-36.1)	34.1 (32.2-36.1)	34.1 (32.7-35.4)	32.9 (31.6-34.3)	34.0 (32.7-35.3)	33.7 (33.1-34.4)	33.2 (31.4-35.0)	35.1 (33.4-36.8)	.99	0.31 (-1.80, 2.43)
P for interaction				.20						
**Parental Education**										
<High school graduate	35.1 (33.7-36.6)	32.8 (31.5-34.2)	33.4 (31.7-35.1)	33.5 (32.8-34.2)	32.2 (29.2-35.1)	33.6 (31.4-35.7)	33.7 (31.7-35.7)	36.2 (33.2-39.2)	.49	1.10 (-2.20, 4.43)
High school graduate or GED or some college	35.0 (33.8-36.1)	34.0 (32.5-35.4)	33.2 (31.5-34.9)	33.7 (32.4-35)	32.2 (30.9-33.6)	32.9 (31.9-33.8)	32.6 (31.6-33.6)	31.6 (30.6-32.6)	<.001	-3.30 (-4.90, -1.80)

© 2021 Liu J et al. *JAMA Network Open.*

	AHA Diet Score, Weighted Mean (95% CI)[a]								P for trend	2017-2018 versus 2003-2004, difference (95% CI)
	2003-2004	2005-2006	2007-2008	2009-2010	2011-2012	2013-2014	2015-2016	2017-2018		
College graduate or above	35.3 (34.0-36.6)	33.4 (30.7-36.0)	34.0 (31.4-36.5)	32.7 (31.0-34.4)	33.8 (31.2-36.3)	31.2 (29.3-33.1)	32.9 (31.2-34.7)	34.6 (32.1-37.0)	.53	-0.75 (-3.50, 1.99)
P for interaction				.02						
**Ratio of family income to poverty level**[c]										
<1.30 [d]	34.7 (33.5-35.8)	34.2 (32.9-35.4)	35.3 (32.8-37.7)	33.1 (31.7-34.5)	33.2 (31.7-34.7)	33.3 (31.9-34.8)	33.8 (32.2-35.3)	33.6 (31.8-35.4)	.15	-1.10 (-3.20, 1.07)
1.30-3.49	36.0 (34.7-37.4)	32.9 (31.2-34.6)	31.9 (30.2-33.7)	33.4 (31.9-34.8)	32.2 (30.0-34.4)	32.5 (31.2-33.9)	32.6 (31.4-33.8)	31.9 (30.4-33.4)	.006	-4.10 (-6.10, -2.10)
≥3.50 [e]	34.3 (32.4-36.1)	34.2 (31.8-36.6)	33.0 (31.7-34.3)	33.4 (31.2-35.5)	32.4 (29.4-35.4)	31.1 (28.9-33.3)	32.5 (30.6-34.4)	33.8 (31.4-36.2)	.22	-0.55 (-3.60, 2.49)
P for interaction				.55						

Abbreviations: NHANES, National Health and Nutrition Examination Survey.
Food sources were grouped as grocery stores (consumed food items obtained from “grocery or supermarket”), restaurants (“restaurant fast food/pizza,

[a] All percentages were survey-weighted; %, proportion.

[b] Trends in diet for the other race or mixed race group are not presented in the race/ethnicity-stratified results due to their small sample sizes. [c]Represents the ratio of family income to the federal poverty threshold, adjusting for household size. For reference, the federal threshold in 2016 for a family of 4 was $24,300/y. A family of 4 earning $33,534/y would have a ratio of 1.38.

[d] Indicates a lower level of income.

[e] Indicates a higher level of income.

© 2021 Liu J et al. *JAMA Network Open.*

eTable 18. Trends in Healthy Eating Index-2015 for Meals Consumed from Different Sources by Age Group, Sex, Race/Ethnicity, Education, and Income among American Children aged 5-19 years, NHANES 2003-2018

	HEI-2015, Weighted Mean (95% CI)[a]								P for trend	2017-2018 versus 2003-2004, difference (95% CI)
	2003-2004	2005-2006	2007-2008	2009-2010	2011-2012	2013-2014	2015-2016	2017-2018		
**Grocery stores**										
**Age group, y**										
5-11	48.9 (47.1-50.7)	49.1 (47.4-50.7)	48.5 (47.3-49.7)	49.9 (48.8-50.9)	51.9 (50.3-53.6)	50.4 (48.3-52.4)	48.6 (47.1-50.2)	49.0 (47.5-50.5)	.58	0.10 (-2.20, 2.44)
12-19	46.7 (45.8-47.6)	45.5 (44.4-46.5)	46.3 (44.9-47.6)	47.1 (46.2-47.9)	49.3 (47.6-51)	46.7 (45.6-47.7)	46.6 (44.8-48.5)	46.2 (44.5-47.9)	.52	-0.46 (-2.40, 1.50)
P for interaction				.99						
**Sex**										
Female	48.1 (46.9-49.3)	47.3 (45.9-48.7)	48.0 (45.9-50.1)	48.4 (47.6-49.3)	50.8 (49.1-52.4)	48.8 (47.6-50.1)	48.4 (47.2-49.6)	48.7 (47.2-50.2)	.11	0.58 (-1.30, 2.49)
Male	47.3 (46.2-48.4)	46.9 (45.6-48.2)	46.6 (45.4-47.7)	48.2 (47.3-49.2)	50.2 (48.9-51.5)	47.9 (46.4-49.4)	46.7 (44.7-48.7)	46.3 (44.7-48)	.86	-0.98 (-2.90, 0.99)
P for interaction				.06						
**Race/ethnicity[b]**										
Non-Hispanic white	47.0 (45.5-48.6)	47.0 (45.0-49.0)	46.9 (45.0-48.7)	48.2 (47.1-49.4)	51.2 (49.6-52.9)	47.9 (45.9-49.8)	47.6 (45.4-49.8)	47.1 (44.4-49.9)	.40	0.11 (-3.0, 3.24)
Non-Hispanic black	46.7 (45.5-47.9)	45.1 (44.3-45.9)	45.7 (44.2-47.1)	46.3 (45.1-47.6)	46.5 (45.3-47.8)	46.4 (45.4-47.3)	45.6 (44.1-47.2)	44.6 (42.2-47.0)	.39	-2.10 (-4.80, 0.57)
Hispanics	50.4 (48.5-52.3)	49.3 (48.2-50.4)	49.3 (47.9-50.7)	49.9 (48.5-51.3)	51 (49.6-52.5)	50.0 (48.8-51.2)	48.2 (46.5-49.8)	49.1 (47.9-50.2)	.17	-1.40 (-3.60, 0.85)
P for interaction				.34						
**Parental Education**										
<High school graduate	47.8 (45.9-49.6)	47.4 (45.5-49.3)	47.1 (45.6-48.7)	47.8 (47.0-48.7)	49.4 (47.1-51.7)	47.0 (45.1-48.9)	46.7 (44.1-49.3)	46.4 (44.9-47.9)	.41	-1.40 (-3.70, 1.01)
High school graduate or GED or some college	47.2 (46.0-48.4)	46.7 (45.6-47.8)	45.5 (44.3-46.8)	47.4 (46.3-48.6)	48.4 (47.3-49.6)	47.2 (45.8-48.5)	46.2 (44.6-47.8)	45.7 (43.8-47.5)	.46	-1.50 (-3.70, 0.69)
College graduate or above	49.5 (46.6-52.4)	48.3 (45.1-51.5)	51.7 (49.8-53.6)	50.2 (48.4-51.9)	55.4 (53.3-57.4)	51.9 (49.3-54.4)	50.7(48.6-52.8)	51.4 (49.1-53.6)	.10	1.92 (-1.70, 5.58)
P for interaction				.13						

© 2021 Liu J et al. *JAMA Network Open.*

	HEI-2015, Weighted Mean (95% CI)[a]								P for trend	2017-2018 versus 2003-2004, difference (95% CI)
	2003-2004	2005-2006	2007-2008	2009-2010	2011-2012	2013-2014	2015-2016	2017-2018		
**Ratio of family income to poverty level[c]**										
<1.30 [d]	48.3 (46.4-50.1)	47.5 (45.6-49.5)	47.0 (45.0-49.0)	46.6 (45.6-47.7)	47.8 (47.0-48.7)	46.8 (45.0-48.7)	44.8 (42.8-46.9)	45.7 (43.9-47.4)	.02	-2.60 (-5.10, 0.02)
1.30-3.49	46.4 (44.9-47.8)	46.8 (45.0-48.5)	45.4 (44.4-46.4)	47.7 (46.2-49.1)	50.9 (48.8-53.1)	47.8 (46.1-49.5)	47.4 (45.8-48.9)	46.9 (45.3-48.4)	.11	0.52 (-1.60, 2.64)
≥3.50 [e]	48.8 (47.1-50.6)	47.0 (45.7-48.2)	49.0 (47.1-50.8)	50.1 (49.3-50.9)	53.6 (51.4-55.8)	50.9 (49.0-52.8)	50.1 (47.7-52.5)	50.5 (48.2-52.9)	.006	1.70 (-1.24, 4.65)
P for interaction				<.001						
				**Restaurants**						
**Age group, y**										
5-11	37.3 (36.2-38.4)	36.6 (35.0-38.2)	36.3 (34.8-37.8)	37.8 (36.4-39.2)	37.0 (35.9-38.1)	34.9 (33.5-36.4)	35.2 (34.1-36.4)	35.5 (34.1-36.9)	.005	-1.80 (-3.60, -0.07)
12-19	36.9 (36.0-37.7)	36.4 (35.7-37.1)	35.1 (34.2-36.0)	37.7 (36.5-38.9)	37.9 (36.8-38.9)	36.3 (35.1-37.6)	35.7 (34.9-36.6)	35.7 (34.4-37)	.27	-1.20 (-2.70, 0.37)
P for interaction				.17						
**Sex**										
Female	37.7 (36.7-38.7)	37.5 (36.0-39.0)	36.6 (35.2-38.1)	37.7 (36.4-38.9)	37.9 (36.4-39.5)	36.0 (34.8-37.2)	35.5 (34.8-36.1)	36.2 (35.0-37.4)	.004	-1.50 (-3.0, 0.01)
Male	36.5 (35.5-37.5)	35.5 (34.7-36.4)	34.5 (33.6-35.4)	37.8 (36.3-39.3)	37.1 (36.0-38.1)	35.4 (33.6-37.3)	35.6 (34.2-36.9)	35.0 (33.5-36.5)	.29	-1.50 (-3.20, 0.25)
Interaction				.29						
**Race/ethnicity[b]**										
Non-Hispanic white	36.6 (35.9-37.4)	35.6 (34.8-36.4)	35.2 (33.9-36.6)	37.7 (36.2-39.2)	37.0 (35.7-38.3)	35.1 (33.6-36.5)	35.3 (34.3-36.4)	35.8 (34.4-37.2)	.28	-0.87 (-2.50, 0.75)
Non-Hispanic black	38.2 (37.2-39.2)	38.8 (37.7-40)	36.6 (35.1-38.0)	38.1 (36.2-40.0)	38.5 (37.1-40.0)	36.7 (35.6-37.8)	35.5 (34.4-36.7)	35.3 (33.8-36.9)	<.001	-2.90 (-4.70, -1.0)
Hispanics	37.5 (36.7-38.3)	38.1 (36.9-39.4)	35.9 (34.8-37.0)	37.0 (36.0-38.1)	38.0 (36.9-39.0)	36.7 (35.4-38.1)	35.5 (33.9-37.1)	36.4 (34.4-38.4)	.09	-1.10 (-3.20, 1.02)
P for interaction				.10						
**Parental Education**										
<High school graduate	36.5 (35.7-37.3)	36.6 (35.2-38.0)	34.6 (33.1-36.1)	36.8 (35.8-37.8)	37.3 (35.8-38.8)	34.9 (32.9-36.9)	34.7 (33.5-36.0)	37.1 (34.9-39.4)	.68	0.62 (-1.80, 3.0)

© 2021 Liu J et al. *JAMA Network Open.*

	HEI-2015, Weighted Mean (95% CI)[a]								P for trend	2017-2018 versus 2003-2004, difference (95% CI)
	2003-2004	2005-2006	2007-2008	2009-2010	2011-2012	2013-2014	2015-2016	2017-2018		
High school graduate or GED or some college	37.0 (36.6-37.5)	36.4 (35.6-37.2)	35.6 (34.3-36.9)	37.4 (35.7-39.0)	37.7 (36.6-38.7)	35.8 (34.5-37.2)	35.8 (34.9-36.7)	35.9 (34.5-37.3)	.14	-1.20 (-2.60, 0.32)
College graduate or above	37.0 (35.3-38.6)	36.4 (35.3-37.6)	36.8 (34.8-38.7)	38.4 (36.4-40.4)	37.2 (35.5-38.9)	36.1 (34.4-37.8)	35.0 (33.7-36.2)	34.5 (33.0-36.0)	.003	-2.50 (-4.60, -0.28)
P for interaction				.21						
**Ratio of family income to poverty level[c]**										
<1.30 [d]	38.2 (37.1-39.3)	36.8 (35.9-37.7)	35.2 (34.0-36.5)	36.8 (36.0-37.7)	37.4 (36.0-38.8)	35.5 (33.9-37.1)	34.9 (33.5-36.3)	35.6 (33.6-37.7)	.01	-2.60 (-4.90, -0.23)
1.30-3.49	36.7 (35.6-37.8)	36.6 (35.5-37.7)	35.1 (33.4-36.8)	37.4 (35.5-39.4)	37.3 (36-38.6)	35.2 (33.7-36.6)	35.0 (34.2-35.9)	36.0 (33.8-38.3)	.21	-0.65 (-3.20, 1.85)
≥3.50 [e]	36.4 (35.3-37.5)	36.0 (34.9-37.1)	36.6 (34.7-38.5)	38.5 (36.7-40.3)	37.5 (35.2-39.8)	36.7 (35.3-38.1)	36.3 (34.9-37.7)	34.8 (33.5-36.1)	.30	-1.56 (-3.23, 0.12)
P for interaction				.47						
					**Schools**					
**Age group, y**										
5-11	45.5 (43.6-47.4)	43.1 (42.0-44.3)	44.3 (42.9-45.7)	45.0 (43.8-46.2)	47.9 (45.8-50.1)	52.6 (51.7-53.5)	52.7 (51.2-54.1)	52.8 (50.9-54.6)	<.001	7.23 (4.59, 9.88)
12-19	42.1 (40.6-43.6)	40.3 (39.2-41.5)	39.7 (37.9-41.6)	39.7 (37.4-42.0)	43.7 (41.2-46.3)	49.5 (47.0-51.9)	52.9 (50.8-55)	50.1 (48.3-52.0)	<.001	8.01 (5.61, 10.4)
P for interaction				.07						
**Sex**										
Female	44.5 (42.5-46.4)	41.9 (40.6-43.2)	41.9 (40.1-43.6)	43.3 (41.3-45.4)	46.7 (43.8-49.6)	51.3 (49.3-53.3)	52.0 (50.2-53.9)	51.6 (49.9-53.3)	<.001	7.12 (4.54, 9.70)
Male	43.7 (41.9-45.4)	42.2 (41.2-43.3)	42.7 (41.1-44.2)	42.1 (40.5-43.8)	45.7 (44.2-47.3)	51.3 (50.0-52.6)	53.4 (51.4-55.4)	51.6 (50.1-53)	<.001	7.89 (5.64, 10.1)
P for interaction				.41						
**Race/ethnicity[b]**										
Non-Hispanic white	43.8 (41.1-46.4)	41.8 (40.7-42.9)	41.9 (39.5-44.3)	41.8 (39.4-44.3)	43.9 (41.2-46.5)	49.8 (46.7-52.8)	51.1 (49.1-53.1)	48.8 (46.3-51.3)	<.001	5.04 (1.38, 8.69)
Non-Hispanic black	44.8 (43.4-46.1)	44.0 (42.2-45.7)	44.0 (42.4-45.6)	44.5 (43.1-45.9)	47.5 (45.4-49.7)	52.4 (51.2-53.7)	53.6 (51.3-55.9)	52.8 (51.6-54.1)	<.001	8.08 (6.29, 9.88)
Hispanics	44.9	42.1	41.9	44.6	48.9	52.7	54.5	54.1	<.001	9.24

© 2021 Liu J et al. *JAMA Network Open.*

	HEI-2015, Weighted Mean (95% CI)[a]								P for trend	2017-2018 versus 2003-2004, difference (95% CI)
	2003-2004	2005-2006	2007-2008	2009-2010	2011-2012	2013-2014	2015-2016	2017-2018		
	(43.3-46.5)	(39.6-44.5)	(40.7-43.0)	(42.5-46.6)	(47.1-50.8)	(51.3-54.1)	(53.3-55.8)	(51.7-56.5)		(6.34, 12.1)
P for interaction				.01						
**Parental Education**										
<High school graduate	43.0 (40.6-45.3)	43.2 (41.3-45.2)	43.6 (42.3-44.9)	43.9 (41.5-46.2)	46.4 (43.7-49.2)	52.6 (50.3-55.0)	53.4 (51.8-55.0)	53.8 (51.3-56.2)	<.001	10.8 (7.44, 14.2)
High school graduate or GED	44.7 (43.2-46.1)	42.0 (41.0-43.0)	41.4 (39.8-43.1)	43.7 (42.4-45.0)	46.0 (43.6-48.4)	50.2 (49.0-51.4)	52.9 (51.2-54.5)	52.2 (51.0-53.4)	<.001	7.51 (5.61, 9.40)
Some college or above	44.3 (38.7-49.8)	40.1 (38.1-42.1)	42.5 (40.4-44.6)	39.2 (37.4-41.0)	46.4 (43.3-49.6)	52.7 (49.0-56.4)	51.7 (48.7-54.8)	48.2 (45.0-51.4)	<.001	3.94 (-2.50, 10.3)
P for interaction				.41						
**Ratio of family income to poverty level**[c]										
<1.30 [d]	45.5 (43.5-47.5)	44.4 (42.9-46.0)	43.2 (41.9-44.5)	44.6 (42.6-46.6)	48.2 (45.9-50.5)	52.3 (51.1-53.4)	53.4 (51.8-55.0)	53.1 (50.9-55.3)	<.001	7.57 (4.61, 10.5)
1.30-3.49	42.8 (41.0-44.5)	41.2 (39.8-42.6)	41.4 (39.8-43)	42.0 (40.1-44.0)	43.8 (41.0-46.6)	50.5 (48.0-53.0)	53.0 (51.1-54.9)	51.6 (50.3-53.0)	<.001	8.86 (6.64, 11.1)
≥3.50 [e]	44.0 (40.2-47.8)	40.3 (38.3-42.3)	41.7 (38.7-44.8)	40.1 (37.0-43.3)	46.4 (44.0-48.7)	50.3 (44.3-56.3)	51.7 (48.4-54.9)	48.6 (45.9-53.3)	<.001	4.60 (-0.62, 9.82)
P for interaction				.29						
				**Others**						
**Age group, y**										
5-11	39.1 (37.2-41.0)	37.9 (35.4-40.4)	36.8 (35.4-38.2)	37.6 (36.4-38.7)	35.2 (32.8-37.5)	35.2 (33.8-36.5)	35.8 (33.5-38.1)	34.7 (32.8-36.6)	<.001	-4.40 (-7.10, -1.70)
12-19	38.8 (37.6-40.0)	37.9 (36.8-39.0)	38.0 (36.4-39.5)	37.0 (34.9-39.2)	33.3 (31.2-35.5)	34.4 (32.0-36.8)	34.0 (32.0-36.1)	34.7 (32.6-36.8)	<.001	-4.0 (-6.40, -1.60)
P for interaction				.41						
**Sex**										
Female	39.5 (38.2-40.8)	37.6 (35.7-39.4)	37.6 (36.4-38.8)	38.4 (36.6-40.2)	34.6 (33-36.1)	35.6 (34.1-37.2)	35.2 (32.7-37.7)	34.9 (33.2-36.6)	<.001	-4.60 (-6.80, -2.50)
Male	38.3 (37.1-39.4)	38.2 (36.3-40.1)	37.2 (35.8-38.6)	36.1 (35.3-36.9)	33.8 (32.6-34.9)	33.9 (32.0-35.8)	34.5 (32.7-36.3)	34.5 (32.5-36.6)	<.001	-3.70 (-6.10, -1.40)
P for interaction				.82						
**Race/ethnicity**[b]										

© 2021 Liu J et al. *JAMA Network Open.*

	HEI-2015, Weighted Mean (95% CI)[a]								P for trend	2017-2018 versus 2003-2004, difference (95% CI)
	2003-2004	2005-2006	2007-2008	2009-2010	2011-2012	2013-2014	2015-2016	2017-2018		
Non-Hispanic white	38.7 (37.2-40.2)	38.1 (35.4-40.8)	37.3 (35.5-39.2)	36.8 (35.4-38.3)	34.0 (32.1-35.8)	34.5 (32.3-36.7)	34.5 (31.8-37.3)	34.9 (32.6-37.1)	<.001	-3.80 (-6.50, -1.20)
Non-Hispanic black	39.4 (38.4-40.3)	38.1 (36.8-39.4)	38.1 (36.4-39.8)	38.0 (36.4-39.6)	32.2 (29.4-35.1)	34.4 (31.7-37.0)	33.4 (31.7-35.2)	33.6 (31.7-35.5)	<.001	-5.80 (-7.90, -3.70)
Hispanics	38.2 (36.1-40.3)	37.2 (35.0-39.3)	37.2 (35.8-38.6)	37.8 (36.3-39.4)	34.6 (31.9-37.4)	34.8 (32.0-37.6)	35.9 (33.8-38.0)	35.3 (32.9-37.7)	.02	-2.90 (-6.10, 0.25)
P for interaction				.03						
**Parental Education**										
<High school graduate	39.5 (37.1-41.9)	36.9 (34.2-39.5)	39.0 (36.2-41.8)	37.9 (36.7-39.1)	33.6 (30.5-36.7)	36.7 (33.4-40.0)	37.2 (34.6-39.8)	35.8 (31.7-39.9)	0.1	-3.70 (-8.40, 1.08)
High school graduate or GED	38.9 (37.4-40.3)	38.5 (36.5-40.6)	37.4 (35.4-39.4)	37.2 (35.6-38.7)	34.1 (32.4-35.8)	34.1 (32.0-36.2)	34.1 (31.8-36.5)	34.3 (32.1-36.5)	<.001	-4.60 (-7.20, -1.90)
Some college or above	39.0 (37.0-41.0)	37.0 (34.9-39.1)	36.8 (33.8-39.9)	37.5 (35.8-39.2)	34.3 (32.2-36.4)	35.2 (32.4-38.0)	34.8 (32.2-37.3)	34.6 (32.4-36.8)	.003	-4.30 (-7.30, -1.40)
P for interaction				.34						
**Ratio of family income to poverty level[c]**										
<1.30 [d]	38.5 (36.8-40.2)	39.1 (37.5-40.6)	38.5 (36.3-40.7)	37.1 (35.5-38.7)	34.8 (33.2-36.3)	35.0 (32.7-37.3)	35.9 (33.1-38.8)	35.3 (31.8-38.8)	.004	-3.30 (-7.10, 0.64)
1.30-3.49	39.9 (38.0-41.9)	37.4 (36-38.8)	37.1 (35.0-39.2)	37.7 (35.9-39.5)	33.2 (31.7-34.7)	34.3 (31.5-37.0)	34.7 (32.7-36.8)	33.5 (31.6-35.4)	<.001	-6.50 (-9.20, -3.70)
≥3.50 [e]	38.7 (36.8-40.6)	37.8 (33.7-42.0)	36.7 (35.2-38.1)	37.0 (35.6-38.3)	34.5 (31.9-37.0)	35.6 (32.9-37.4)	34.7 (32.4-37.0)	35.0 (32.7-37.3)	.007	-3.68 (-6.62, -0.74)
P for interaction				.49						

Abbreviations: NHANES, National Health and Nutrition Examination Survey.

Food sources were grouped as grocery stores (consumed food items obtained from “grocery or supermarket”), restaurants (“restaurant fast food/pizza, restaurant with waiter/waitress, restaurant no additional information”), schools (“K-12 school cafeteria, childcare center”), worksites (“cafeteria not in a K-12 school, vending machine, common coffee pot or snack tray) and other sources refer to food items obtained from elsewhere (“sport, recreation, or entertainment facility, street vendor, vending truck, from someone else/gift, and others).

[a] All percentages were survey-weighted; %, proportion.

[b] Trends in diet for the other race or mixed race group are not presented in the race/ethnicity-stratified results due to their small sample sizes.

[c] Represents the ratio of family income to the federal poverty threshold, adjusting for household size. For reference, the federal threshold in 2016 for a family of 4 was $24,300/y. A family of 4 earning $33,534/y would have a ratio of 1.38.

[d] Indicates a lower level of income.

© 2021 Liu J et al. *JAMA Network Open.*

[e] Indicates a higher level of income.

eTable 19. Trends in Proportions of US Adults Age 20+ Years with Poor or Intermediate Diet based on American Heart Association Diet Score by Different Food Sources and by Age, Sex, Race/Ethnicity, Education, and Income, NHANES 2003-2018.

Grocery	AHA Diet Score, Weighted % (95% CI)[a]								*P*-trend	P for interaction
	2003-2004	2005-2006	2007-2008	2009-2010	2011-2012	2013-2014	2015-2016	2017-2018		
**Poor Diet**										
					Grocery stores					
**Age group, y**										
20-49	46.4 (42.1-50.6)	41 (35.9-46.1)	40.8 (36.2-45.4)	37.3 (34.3-40.3)	36.1 (32.5-39.6)	35.6 (33.3-37.9)	36.1 (31.5-40.6)	35.5 (31.5-39.5)	<.001	<.001
≥50	31.3 (28.5-34.1)	28.8 (25.1-32.4)	27.3 (22.1-32.5)	26.7 (24.4-29)	23.4 (20.3-26.5)	26.5 (23.3-29.7)	28.8 (25.9-31.7)	30.1 (25.5-34.6)	.76	
**Sex**										
Female	36.0 (32.0-40.1)	31.1 (27.2-34.9)	31.5 (26.5-36.5)	28.8 (26.8-30.9)	26.8 (24.3-29.3)	26.6 (23.3-29.9)	28.5 (24.8-32.1)	28.9 (24.5-33.2)	.006	.74
Male	44.4 (40.8-48.1)	41.0 (36.2-45.8)	39.2 (34.1-44.3)	36.9 (33.8-40.0)	34.0 (30.9-37.0)	36.5 (34.6-38.4)	37.1 (32.8-41.4)	37.3 (32.7-41.8)	.006	
**Race/ethnicity[b]**										
Non-Hispanic white	40.1 (36.5-43.7)	34.8 (30.3-39.3)	34.9 (28.8-41)	32.6 (30.7-34.4)	29.1 (26.4-31.7)	31.1 (28.5-33.7)	33.2 (28.9-37.5)	33.9 (29.3-38.5)	.02	.47
Non-Hispanic black	50.6 (43-58.2)	47.5 (39.5-55.5)	44.2 (39.8-48.6)	43.3 (37-49.6)	39.6 (32.9-46.2)	40.5 (35.8-45.1)	40.6 (35.6-45.6)	40.8 (33.8-47.7)	.01	
Hispanics	32.7 (26.3-39.2)	34.2 (29-39.5)	34.6 (31.2-37.9)	30 (24.5-35.6)	34.7 (29.9-39.5)	31.5 (25.8-37.2)	32.7 (29.1-36.2)	28.9 (23.8-34)	.21	
**Education**										
<High school graduate	42.8 (37.5-48.0)	42.6 (38.1-47)	44.8 (40.6-49.0)	39.1 (35.5-42.7)	40.9 (38.4-43.5)	38.2 (34.5-41.8)	40.6 (35.8-45.3)	37.7 (31.3-44.2)	.06	.54
High school graduate or GED	48.3 (45.6-50.9)	43.0 (38.3-47.8)	40.1 (33.8-46.4)	43.5 (39.6-47.4)	35.6 (31.4-39.7)	38.9 (35.3-42.4)	38.5 (34.8-42.2)	41.8 (37.2-46.4)	.01	
Some college or above	41.9 (38.0-45.8)	36.3 (30.4-42.1)	34.8 (30.5-39.2)	34.4 (31.3-37.6)	32.7 (28.7-36.6)	34.4 (30.4-38.5)	33.8 (28.3-39.3)	38.4 (33.6-43.2)	.23	
College graduate or above	26.2 (22.6-29.9)	24.0 (19.1-28.9)	22.3 (17.4-27.3)	17.7 (14.5-21.0)	18.9 (15.7-22.1)	19.2 (15.2-23.2)	24.0 (20.5-27.6)	17.4 (14.2-20.7)	.01	
**Ratio of family income to poverty level[c]**										
<1.30 [d]	45.8 (40.6-50.9)	43.5 (38.0-49.0)	45.5 (39.8-51.1)	41.7 (38.9-44.5)	40.4 (36.3-44.4)	43.1 (38.7-47.6)	39.7 (34.5-44.8)	41.3 (36.1-46.5)	.09	.09

© 2021 Liu J et al. *JAMA Network Open.*

Grocery	AHA Diet Score, Weighted % (95% CI)[a]								*P*-trend	P for interaction
	2003-2004	2005-2006	2007-2008	2009-2010	2011-2012	2013-2014	2015-2016	2017-2018		
1.30-3.49	40.1 (35.8-44.4)	38.7 (34.3-43)	35.4 (30.2-40.7)	36.3 (32.7-39.9)	31.4 (28.6-34.2)	32.8 (30.2-35.4)	35.9 (32.6-39.3)	36.0 (31.1-41.0)	.07	
≥3.50 [e]	36.9 (32.7-41.4)	30.7 (25.7-36.2)	31.0 (26.8-35.6)	25.7 (23.1-28.5)	23.1 (18.6-28.4)	23.6 (20.7-26.8)	26.8 (23.0-31.0)	26.5 (22.4-31.1)	.001	
**Intermediate Diet**										
**Age group, y**										
20-49	52.9 (48.6-57.1)	57.4 (52.8-62.1)	57.3 (52.7-62)	60.7 (58-63.4)	61.8 (58.5-65.1)	62.3 (59.8-64.7)	61.8 (57.3-66.4)	63.1 (59.3-66.8)	<.001	<.001
≥50	66.7 (64.1-69.3)	67.8 (64.2-71.5)	70 (65.1-74.9)	69.8 (67.5-72)	72.8 (70.3-75.4)	69.8 (66.7-72.8)	67.6 (64.7-70.5)	66.7 (62.4-71.1)	.89	
**Sex**										
Female	62.5 (58.6-66.5)	65.8 (62.3-69.3)	66.3 (61.5-71.0)	68.5 (66.6-70.4)	69.7 (67.4-72.0)	70.0 (66.8-73.3)	68.2 (64.6-71.8)	68.2 (64.0-72.4)	.02	.98
Male	54.4 (50.6-58.2)	57.4 (52.8-62.1)	58.7 (53.4-63.9)	60.4 (57.6-63.3)	63.8 (61.1-66.4)	61.1 (59.1-63.2)	60.7 (56.4-64.9)	61.2 (56.9-65.6)	.01	
**Race/ethnicity[b]**										
Non-Hispanic white	58.5 (54.9-62.0)	62.5 (58.4-66.7)	63.0 (57.3-68.8)	64.3 (62.3-66.4)	67.9 (65.8-69.9)	66.1 (63.6-68.5)	63.8 (59.4-68.2)	64.0 (59.4-68.5)	.04	.21
Non-Hispanic black	48.5 (40.7-56.2)	50.6 (43.3-57.9)	53.9 (49.4-58.4)	54.5 (49.0-60.0)	58.3 (52.5-64.2)	57.2 (52.6-61.8)	57.0 (52.4-61.7)	57.5 (51.1-63.9)	.01	
Hispanics	66.6 (60.5-72.7)	65.3 (60.1-70.4)	64.0 (60.4-67.6)	68.6 (62.9-74.3)	63.7 (58.6-68.8)	66.0 (60.5-71.5)	65.5 (62.0-69.0)	67.8 (63.1-72.5)	.67	
**Education**										
<High school graduate	56.9 (51.6-62.1)	56.8 (52.2-61.3)	54.1 (50.2-57.9)	60.3 (56.6-63.9)	57.2 (54.6-59.9)	60.7 (57.3-64.0)	56.8 (52.2-61.5)	59.8 (53.9-65.8)	.24	
High school graduate or GED	50.7 (48.3-53.2)	56.0 (51.3-60.7)	57.8 (51.4-64.1)	53.6 (49.8-57.3)	62.4 (58.0-66.8)	59.3 (55.7-63.0)	59.8 (56.0-63.5)	56.9 (52.2-61.7)	.01	.76
Some college or above	57.2 (53.0-61.4)	61.5 (55.9-67.1)	62.7 (58.5-66.9)	64.2 (60.9-67.5)	65.1 (61.2-68.9)	63.8 (59.9-67.8)	65 (59.7-70.3)	60 (55.1-64.9)	.22	
College graduate or above	70.9 (66.6-75.2)	71.0 (66.7-75.4)	74.8 (69.3-80.3)	76.9 (73.5-80.4)	76.4 (74.5-78.4)	75 (71.5-78.5)	70.7 (66.5-74.8)	78.8 (76.0-81.7)	.06	
**Ratio of family income to poverty level[c]**										
<1.30 [d]	53.5 (48.2-58.8)	56.1 (50.6-61.6)	51.7 (46.2-57.1)	57.1 (54.5-59.7)	57.9 (54.3-61.6)	55.4 (51-59.7)	58.9 (54.3-63.4)	56.6 (51.5-61.8)	.16	.12
1.30-3.49	59.0 (54.8-63.2)	60.0 (55.4-64.7)	62.8 (57.9-67.6)	61.7 (58.1-65.3)	66.7 (63.7-69.7)	65.4 (62.8-67.9)	62.7 (59.4-65.9)	61.4 (56.7-66.1)	.14	

© 2021 Liu J et al. *JAMA Network Open.*

Grocery	AHA Diet Score, Weighted % (95% CI)[a]								*P*-trend	P for interaction
	2003-2004	2005-2006	2007-2008	2009-2010	2011-2012	2013-2014	2015-2016	2017-2018		
≥3.50 [e]	61.1 (56.2-65.8)	65.2 (60.1-69.9)	66.6 (62.0-70.8)	70.1 (67.1-73.0)	72.3 (67.6-76.5)	71.9 (69.5-74.2)	68.6 (63.7-73.2)	71.2 (67.0-75.1)	.001	
Restaurants										
**Age group, y**										
20-49	69.3 (65.4-73.1)	70.7 (67.3-74.1)	72.1 (68.4-75.7)	68.8 (64.4-73.2)	66.2 (62.5-69.8)	67.5 (64.6-70.3)	66.3 (62.6-70)	67.6 (62.9-72.2)	.06	.20
≥50	58.4 (54.2-62.5)	62.2 (58.5-66.0)	61.5 (58.5-64.4)	58.0 (53.8-62.3)	59.2 (55.4-63.1)	52.6 (47.5-57.6)	60.1 (53.2-66.9)	62.2 (56.8-67.6)	.88	
**Sex**										
Female	63.0 (57.9-68.1)	64.2 (60.2-68.2)	66.8 (63.7-70.0)	62.4 (59.1-65.8)	60.0 (55.7-64.4)	58.6 (56.4-60.9)	62.0 (57.1-66.9)	61.7 (57.3-66.0)	.12	.80
Male	67.8 (63.5-72.1)	70.9 (68.1-73.7)	69.4 (65.3-73.5)	67.0 (62.0-72.0)	66.5 (63.7-69.4)	64.1 (61.0-67.2)	65.5 (60.0-70.9)	68.9 (64.9-72.8)	.21	
**Race/ethnicity[b]**										
Non-Hispanic white	65.7 (61.5-69.9)	67.5 (64.5-70.4)	68.0 (64.7-71.4)	63.3 (58.9-67.8)	63.7 (59.7-67.8)	59.8 (56.8-62.7)	62.9 (56.5-69.3)	66.3 (61.1-71.6)	.17	.63
Non-Hispanic black	70.9 (64.9-76.8)	74.8 (68.8-80.8)	73.8 (71.1-76.6)	71.7 (67.9-75.5)	69.8 (62.8-76.7)	67.3 (62.6-72)	69.0 (64.5-73.5)	68.6 (64.6-72.6)	.04	
Hispanics	62.5 (54.4-70.5)	68.1 (61.5-74.8)	64.7 (60.1-69.3)	65.6 (61.7-69.5)	59.0 (55.6-62.5)	66.7 (63.0-70.5)	65.8 (61.9-69.6)	61.1 (55.3-66.9)	.59	
**Education**										
<High school graduate	66.3 (61.8-70.9)	75.1 (71.0-79.3)	72.2 (67.8-76.6)	65.7 (59.8-71.7)	65.9 (60.6-71.1)	69.6 (65.5-73.7)	69.1 (64.9-73.4)	68.1 (61.5-74.6)	.42	.09
High school graduate or GED	72.8 (68.0-77.5)	71.5 (67.2-75.8)	72.7 (69.3-76.0)	71.2 (65.6-76.9)	76.0 (71.6-80.5)	71.1 (64.5-77.7)	76.2 (71.2-81.2)	74.7 (70.4-78.9)	.26	
Some college or above	64.0 (59.4-68.6)	68.8 (64.5-73.1)	69.4 (65.5-73.3)	68.2 (63.8-72.5)	63.9 (58.5-69.4)	63.1 (60-66.3)	65.5 (59.5-71.4)	66.2 (61.3-71.1)	.46	
College graduate or above	58.6 (50.2-67)	59.1 (56.1-62.0)	59.7 (54.5-64.9)	54.8 (49.9-59.6)	54.0 (48.1-59.9)	49.6 (46.1-53)	53 (46.6-59.4)	55.5 (50.3-60.7)	.05	
**Ratio of family income to poverty level[c]**										
<1.30 [d]	69.0 (63.0-75.1)	76.7 (70.1-83.4)	72.4 (67.6-77.1)	70.8 (66.0-75.6)	70.3 (65.2-75.4)	72.1 (67.2-77.1)	70.3 (64.4-76.1)	72.5 (67.2-77.9)	.96	.33
1.30-3.49	64.3 (59.6-69.0)	72.6 (69.3-75.8)	73.3 (69.6-76.9)	66.9 (61.7-72.1)	68.9 (65.6-72.1)	66.3 (62.1-70.5)	68.2 (64-72.3)	66.2 (61.9-70.6)	.35	
≥3.50 [e]	63.7 (59.3-67.9)	62.1 (58.3-65.7)	63.1 (58.3-67.6)	60.5 (57.1-63.8)	56.4 (51.9-60.7)	52.3 (49.4-55.2)	58.4 (52.0-64.6)	60.5 (54.1-66.6)	.03	

© 2021 Liu J et al. *JAMA Network Open.*

Grocery	AHA Diet Score, Weighted % (95% CI)[a]								*P*-trend	P for interaction
	2003-2004	2005-2006	2007-2008	2009-2010	2011-2012	2013-2014	2015-2016	2017-2018		
**Intermediate Diet**										
**Age group, y**										
20-49	30.7 (26.9-34.6)	29.2 (25.8-32.6)	27.8 (24.1-31.4)	31.1 (26.7-35.5)	33.8 (30.2-37.5)	32.5 (29.6-35.4)	33.6 (29.9-37.3)	32.4 (27.8-37.1)	.05	.19
≥50	41.6 (37.5-45.8)	37.8 (34.0-41.5)	38.5 (35.5-41.4)	42.0 (37.7-46.2)	40.6 (36.7-44.4)	47.4 (42.4-52.4)	39.9 (33.1-46.8)	37.8 (32.4-43.2)	.88	
**Sex**										
Female	37.0 (31.9-42.1)	35.7 (31.7-39.8)	33.0 (29.8-36.1)	37.6 (34.2-40.9)	40.0 (35.6-44.3)	41.3 (39.1-43.6)	38.0 (33.1-42.9)	38.3 (34.0-42.7)	.11	.77
Male	32.2 (27.9-36.5)	29.1 (26.3-31.9)	30.6 (26.5-34.7)	33.0 (28.0-38.0)	33.4 (30.6-36.1)	35.9 (32.8-39.0)	34.4 (28.9-39.9)	31.1 (27.2-35.1)	.21	
**Race/ethnicity[b]**										
Non-Hispanic white	34.3 (30.1-38.5)	32.5 (29.5-35.4)	31.8 (28.5-35.2)	36.7 (32.2-41.1)	36.2 (32.2-40.2)	40.2 (37.3-43.2)	37.0 (30.6-43.5)	33.7 (28.4-38.9)	.16	.62
Non-Hispanic black	29.1 (23.2-35.1)	25.2 (19.2-31.2)	26.1 (23.4-28.7)	28.0 (23.8-32.2)	30.2 (23.3-37.2)	32.5 (27.7-37.3)	31.0 (26.5-35.5)	31.4 (27.4-35.4)	.04	
Hispanics	37.5 (29.5-45.6)	31.9 (25.2-38.5)	35.3 (30.7-39.9)	34.4 (30.5-38.3)	40.9 (37.4-44.4)	33.2 (29.3-37.0)	34.2 (30.4-38.1)	38.9 (33.1-44.7)	.60	
**Education**										
<High school graduate	33.7 (29.1-38.2)	24.9 (20.7-29)	27.8 (23.4-32.2)	34.3 (28.3-40.2)	34.1 (28.9-39.4)	30.3 (26.2-34.4)	30.9 (26.6-35.1)	31.9 (25.4-38.5)	.43	.09
High school graduate or GED	27.2 (22.5-32)	28.5 (24.2-32.8)	27.3 (24-30.7)	28.8 (23.1-34.4)	24.0 (19.5-28.4)	28.9 (22.3-35.5)	23.8 (18.8-28.8)	25.3 (21.1-29.6)	.26	
Some college or above	36.0 (31.4-40.6)	31.2 (26.9-35.5)	30.3 (26.4-34.2)	31.7 (27.3-36.1)	36.1 (30.6-41.5)	36.8 (33.6-39.9)	34.4 (28.5-40.4)	33.8 (28.9-38.7)	.46	
College graduate or above	41.4 (33.0-49.8)	40.8 (37.8-43.7)	40.2 (35.1-45.4)	45.2 (40.4-50.1)	45.8 (39.9-51.6)	50.4 (47.0-53.9)	47.0 (40.6-53.4)	44.5 (39.3-49.7)	.04	
**Ratio of family income to poverty level[c]**										
<1.30 [d]	31.0 (24.9-37.0)	23.3 (16.6-29.9)	27.1 (22.5-31.7)	29.2 (24.4-34.0)	29.7 (24.6-34.8)	27.9 (22.9-32.8)	29.5 (23.8-35.2)	27.5 (22.1-32.8)	.94	
1.30-3.49	35.7 (31.0-40.4)	27.4 (24.2-30.7)	26.7 (23.1-30.3)	33.1 (27.9-38.3)	31.0 (27.8-34.1)	33.7 (29.5-37.9)	31.8 (27.7-36)	33.8 (29.4-38.1)	.35	.33
≥3.50 [e]	36.3 (32.1-40.7)	37.8 (34.2-41.7)	36.9 (32.4-41.7)	39.4 (36.1-42.9)	43.6 (39.3-48.0)	47.6 (44.8-48.0)	41.6 (35.4-48.0)	39.5 (33.4-46.0)	.03	
Worksites										

© 2021 Liu J et al. *JAMA Network Open.*

Grocery	AHA Diet Score, Weighted % (95% CI)[a]								*P*-trend	P for interaction
	2003-2004	2005-2006	2007-2008	2009-2010	2011-2012	2013-2014	2015-2016	2017-2018		
**Age group, y**										
20-49	58.5 (50.8-66.1)	59.4 (53.5-65.2)	62.6 (56.1-69.0)	57.4 (53.1-61.6)	58.7 (49.2-68.2)	55.6 (49.3-61.9)	48.4 (39.7-57.2)	51.5 (43.1-60.0)	.04	.08
≥50	47.5 (36.3-58.6)	52.1 (43.6-60.7)	46.3 (38.0-54.6)	44.0 (35.7-52.3)	54.6 (40.5-68.8)	61.7 (46.8-76.6)	54.5 (41.6-67.5)	49.0 (31-67.1)	.38	
**Sex**										
Female	51.6 (42.6-60.7)	56.1 (46.8-65.4)	50.9 (45.7-56.1)	54.0 (45.5-62.5)	52.4 (42.5-62.2)	52.0 (45.0-59.0)	47.1 (32.8-61.3)	46.1 (39.5-52.8)	.23	.63
Male	58.9 (51.8-66.0)	57.2 (51.3-63.1)	62.2 (52.4-72.0)	51.5 (43.3-59.7)	61.5 (51.3-71.6)	62.0 (54.2-69.8)	53.1 (42.3-63.8)	54.5 (44.0-65.0)	.53	
**Race/ethnicity[b]**										
Non-Hispanic white	52.6 (45.4-59.7)	55.8 (48.9-62.8)	56.5 (49.6-63.3)	50.0 (45.4-54.5)	59.8 (46.1-73.4)	59.2 (50.2-68.1)	54.0 (44.3-63.7)	52.8 (42.6-63.0)	.73	.01
Non-Hispanic black	61.1 (50.2-72.1)	48.9 (41.2-56.5)	53.3 (41.6-65.1)	56.7 (43.4-69.9)	58.8 (49.0-68.6)	50.9 (42.6-59.1)	42.5 (26.0-59.0)	41.9 (33.7-50.0)	.04	
Hispanics	72.7 (64.2-81.1)	65.0 (53.9-76.1)	62.5 (53.7-71.4)	59.5 (49.9-69.2)	52.1 (39.7-64.5)	56.7 (44.8-68.6)	55.8 (43.6-68.1)	50.1 (40.4-59.9)	.001	
**Education**										
<High school graduate	71.1 (57.9-84.3)	54.1 (39.3-68.9)	54.7 (44.5-64.9)	61.7 (46.9-76.6)	63.4 (50.8-75.9)	51.0 (42.1-59.9)	53.0 (40.5-65.6)	43.9 (17.1-70.7)	.10	.21
High school graduate or GED	61.5 (53.2-69.7)	68.1 (59.8-76.5)	61.0 (53.4-68.6)	50.7 (37.7-63.7)	69.5 (61.5-77.6)	65.9 (57.9-74)	51.9 (34.9-69)	54.9 (44.7-65)	.17	
Some college or above	54.3 (46.9-61.7)	52.1 (41.8-62.5)	55.2 (46.2-64.1)	52.1 (47.2-57)	56.8 (45.8-67.7)	60.2 (51.8-68.7)	57.5 (47.0-68.1)	53.5 (41.9-65.1)	.50	
College graduate or above	47.1 (32.7-61.5)	55.3 (42.8-67.9)	56.5 (43.4-69.6)	51.5 (45.0-58.0)	50.0 (32.5-67.5)	52.8 (42.6-62.9)	43.2 (33.2-53.2)	46.2 (33.1-59.3)	.42	
**Ratio of family income to poverty level[c]**										
<1.30 [d]	60.3 (46.5-74.2)	62.9 (53.4-72.4)	63.8 (53.6-74.1)	53.0 (43.0-62.9)	74.2 (67.3-81.1)	64.7 (53.7-75.7)	48.9 (34.1-63.6)	41.8 (27.4-56.3)	.14	.65
1.30-3.49	59.2 (52.6-65.9)	57.0 (49.0-65.0)	59.8 (53.3-66.2)	55.7 (45.5-66)	54.4 (48-60.7)	60.1 (50.6-69.5)	48.1 (36.3-59.8)	54.2 (44.3-64.1)	.21	
≥3.50 [e]	52.3 (41.7-62.3)	55.5 (47.2-63.5)	54.5 (46.4-62.4)	49.4 (45.1-53.7)	52.6 (36.0-68.7)	55.2 (44.9-65.1)	51.0 (39.8-62.2)	48.5 (35.4-61.9)	.59	
**Intermediate Diet**										
**Age group, y**										

© 2021 Liu J et al. *JAMA Network Open.*

Grocery	AHA Diet Score, Weighted % (95% CI)[a]								P-trend	P for interaction
	2003-2004	2005-2006	2007-2008	2009-2010	2011-2012	2013-2014	2015-2016	2017-2018		
20-49	41.4 (33.9-48.9)	40.6 (34.8-46.5)	36.9 (30.5-43.3)	42.6 (38.4-46.9)	41.3 (31.8-50.8)	43.7 (37.0-50.3)	51.6 (42.8-60.3)	48.5 (40-56.9)	.04	.68
≥50	52.5 (41.4-63.7)	47.9 (39.3-56.4)	53.7 (45.4-62)	56.0 (47.7-64.3)	45.4 (31.2-59.5)	38.3 (23.4-53.2)	45.5 (32.5-58.4)	51.0 (32.9-69)	.38	
**Sex**										
Female	48.4 (39.3-57.4)	43.9 (34.6-53.2)	48.3 (43.1-53.5)	46.0 (37.5-54.5)	47.6 (37.8-57.5)	47.3 (41.0-53.7)	52.9 (38.7-67.2)	53.9 (47.2-60.5)	.23	.65
Male	40.8 (33.8-47.9)	42.8 (36.9-48.7)	37.8 (28-47.6)	48.5 (40.3-56.7)	38.5 (28.4-48.7)	37.7 (29.5-45.9)	46.9 (36.2-57.7)	45.5 (35.0-56.0)	.52	
**Race/ethnicity[b]**										
Non-Hispanic white	47.3 (40.2-54.4)	44.2 (37.2-51.1)	43.0 (36.5-49.6)	50.0 (45.5-54.6)	40.2 (26.6-53.9)	40.4 (31.6-49.2)	46.0 (36.3-55.7)	47.2 (37.0-57.4)	.74	.01
Non-Hispanic black	38.9 (27.9-49.8)	51.1 (43.5-58.8)	46.7 (34.9-58.4)	43.3 (30.1-56.6)	41.2 (31.4-51)	47.9 (38.8-57.1)	57.5 (41.0-74.0)	58.1 (50.0-66.3)	.04	
Hispanics	27.3 (18.9-35.8)	35.0 (23.9-46.1)	37.5(28.6-46.3)	40.5 (30.8-50.1)	47.9 (35.5-60.3)	43.3 (31.4-55.2)	44.2 (31.9-56.4)	49.9 (40.1-59.6)	.001	
**Education**										
<High school graduate	28.9 (15.7-42.1)	45.9 (31.1-60.7)	45.3 (35.1-55.5)	38.3 (23.4-53.1)	36.6 (24.1-49.2)	49.0 (40.1-57.9)	47.0 (34.4-59.5)	56.1 (29.3-82.9)	.10	.22
High school graduate or GED	38.5 (30.3-46.8)	31.9 (23.5-40.2)	39.0 (31.4-46.6)	49.3 (36.3-62.3)	30.5 (22.4-38.5)	34.1 (26.0-42.1)	48.1 (31-65.1)	45.1 (35.0-55.3)	.17	
Some college or above	45.4 (38.1-52.6)	47.9 (37.5-58.2)	44.8 (35.9-53.8)	47.9 (43.0-52.8)	43.2 (32.3-54.2)	39.8 (31.3-48.2)	42.5 (31.9-53)	46.5 (34.9-58.1)	.53	
College graduate or above	52.9 (38.5-67.3)	44.7 (32.1-57.2)	42.4 (29.8-55.0)	48.5 (42.0-55.0)	50.0 (32.5-67.5)	46.0 (36.1-55.9)	56.8 (46.8-66.8)	53.8 (40.7-66.9)	.43	
**Ratio of family income to poverty level[c]**										
<1.30 [d]	39.7 (25.8-53.5)	37.1 (27.6-46.6)	36.2 (25.9-46.4)	47.0 (37.1-57.0)	25.8 (18.9-32.7)	35.3 (24.3-46.3)	51.1 (36.4-65.9)	58.2 (43.7-72.6)	.14	.65
1.30-3.49	40.8 (34.1-47.4)	43.0 (35.0-51.0)	40.2 (33.8-46.7)	44.3 (34.0-54.5)	45.6 (39.3-52.0)	39.9 (30.5-49.4)	51.9 (40.2-63.7)	45.8 (35.9-55.7)	.21	
≥3.50 [e]	47.5 (37.6-57.6)	44.5 (36.5-52.8)	44.9 (37.6-52.4)	50.6 (46.3-54.9)	47.4 (31.3-64.0)	43.8 (34.0-54.1)	49.0 (37.8-60.2)	51.5 (38.1-64.6)	.59	
					Others					
**Age group, y**										
20-49	35.6 (32.0-39.2)	39.9 (36.0-43.8)	44.2 (40.1-48.3)	40.9 (36.4-45.4)	43.8 (40.4-47.1)	45.2 (40.8-49.5)	46.3 (43.2-49.5)	48.1 (42.8-53.4)	<.001	.18

© 2021 Liu J et al. *JAMA Network Open.*

Grocery	AHA Diet Score, Weighted % (95% CI)[a]								*P*-trend	P for interaction
	2003-2004	2005-2006	2007-2008	2009-2010	2011-2012	2013-2014	2015-2016	2017-2018		
≥50	31.5 (26.3-36.8)	38.3 (33.7-43.0)	34.0 (29.3-38.7)	35.4 (31.9-38.8)	32.6 (27.9-37.4)	40.0 (37.1-43.0)	37.1 (32.2-41.9)	39.6 (35.4-43.9)	.03	
**Sex**										
Female	33.9 (30.3-37.5)	37.2 (32.9-41.6)	36.7 (31.8-41.7)	36.4 (33.5-39.2)	35.8 (32.0-39.7)	39.8 (36.8-42.8)	38.3 (34.9-41.7)	40.8 (37.7-44.0)	.009	.24
Male	33.7 (29.6-37.8)	41.4 (38-44.8)	43.0 (39.1-46.9)	40.5 (36.1-45.0)	41.7 (37.4-46)	45.7 (41.7-49.7)	45.3 (41.0-49.6)	46.9 (38.5-55.3)	.002	
**Race/ethnicity[b]**										
Non-Hispanic white	32.2 (29.0-35.5)	39.0 (35.0-42.9)	39.4 (35.4-43.5)	37.6 (34.0-41.1)	37.5 (33.9-41.1)	44.4 (41-47.8)	42.2 (37.7-46.7)	46.0 (39.6-52.3)	<.001	.001
Non-Hispanic black	35.0 (27.4-42.6)	42.6 (37.2-48.1)	42.1 (37.3-47.0)	41.8 (35.4-48.3)	37.6 (33.7-41.4)	39.5 (33.8-45.1)	41.7 (37.5-46.0)	42.8 (37.4-48.3)	.34	
Hispanics	44.4 (36.7-52.2)	42.8 (36.4-49.2)	41.2 (36.0-46.3)	43.4 (38.7-48.1)	43.8 (35.6-52.1)	38.8 (33.9-43.6)	44.0 (41.8-46.2)	39.6 (33.5-45.6)	.36	
**Education**										
<High school graduate	36.8 (30.0-43.7)	39.1 (33.1-45.2)	41.0 (33.1-49.0)	38.9 (32.5-45.3)	38.4 (31.6-45.2)	43.8 (38.5-49.1)	42.5 (35.7-49.3)	42.6 (35.1-50.1)	.15	.04
High school graduate or GED	34.3 (27.5-41.1)	37.1 (28.7-45.6)	43.1 (38.4-47.8)	40.4 (34.6-46.3)	43.9 (35.6-52.3)	47.5 (43.1-51.9)	47.1 (40.9-53.2)	48.5 (43.2-53.8)	<.001	
Some college or above	34.2 (31.4-37.0)	41.7 (37.1-46.3)	34.5 (28.5-40.4)	40.6 (36.6-44.6)	40.6 (36.7-44.5)	43.0 (39.5-46.5)	44.4 (39.9-49.0)	47.6 (44.2-51)	<.001	
College graduate or above	31.0 (26.7-35.3)	38.0 (32.4-43.7)	41.2 (37.3-45.1)	34.1 (26.2-42.0)	34.6 (29.9-39.3)	38.9 (34.5-43.3)	36.1 (31.2-40.9)	36.3 (28.8-43.8)	.71	
**Ratio of family income to poverty level[c]**										
<1.30 [d]	37.8 (30.4-45.1)	40.4 (35.0-45.8)	42.3 (35.3-49.4)	39.3 (32.9-45.6)	41.8 (36.1-47.5)	41.9 (36.9-46.8)	45.7 (38.9-52.6)	46.1 (41.3-50.8)	.03	.41
1.30-3.49	35.6 (31.4-39.7)	39.3 (34.0-44.5)	35.4 (32.1-38.7)	36.5 (31.3-41.7)	39.2 (34.0-44.4)	48.2 (44.2-52.2)	43.2 (37.5-48.9)	44.1 (38.9-49.2)	<.001	
≥3.50 [e]	31.5 (27.5-35.8)	39.2 (36.4-42.0)	42.6 (37.8-47.6)	39.6 (35.6-43.9)	35.9 (31.5-40.5)	39.9 (35.4-44.5)	38.2 (34.6-42.0)	43.4 (35.4-51.6)	.10	
**Intermediate Diet**										
**Age group, y**										
20-49	64.4 (60.8-68.0)	60.1 (56.2-64)	55.8 (51.7-59.9)	59.0 (54.5-63.6)	56.2 (52.9-59.6)	54.7 (50.3-59)	53.5 (50.5-56.6)	51.7 (46.4-57.0)	<.001	.19
≥50	68.1 (62.3-73.8)	61.2 (56.4-66.0)	65.6 (60.8-70.3)	64.6 (61.2-67.9)	66.8 (61.9-71.7)	59.8 (57.0-62.7)	62.7 (57.7-67.7)	59.7 (55.3-64.0)	.04	

© 2021 Liu J et al. *JAMA Network Open.*

Grocery	AHA Diet Score, Weighted % (95% CI)[a]								*P*-trend	P for interaction
	2003-2004	2005-2006	2007-2008	2009-2010	2011-2012	2013-2014	2015-2016	2017-2018		
**Sex**										
Female	65.7 (62.2-69.3)	62.6 (58.2-67.0)	62.9 (57.9-67.9)	63.6 (60.8-66.4)	63.7 (59.6-67.8)	59.9 (56.9-62.9)	61.4 (58.0-64.9)	58.6 (55.4-61.9)	.008	.23
Male	66.3 (62.2-70.4)	58.4 (55.0-61.8)	57.0 (53.1-60.8)	59.4 (55.0-63.8)	58.3 (54.0-62.6)	54.3 (50.3-58.3)	54.5 (50.2-58.9)	52.7 (44.4-61.0)	.002	
**Race/ethnicity[b]**										
Non-Hispanic white	67.5 (64.3-70.8)	60.8 (56.8-64.8)	60.3 (56.2-64.4)	62.4 (58.8-65.9)	62.2 (58.5-65.8)	55.5 (52.0-59.0)	57.6 (53.0-62.2)	53.5 (47.2-59.7)	<.001	<.001
Non-Hispanic black	65.0 (57.4-72.6)	57.4 (51.9-62.8)	57.9 (53.0-62.7)	58.2 (51.7-64.6)	62.4 (58.6-66.2)	60.5 (54.9-66.2)	58.0 (53.8-62.2)	57.2 (51.7-62.6)	.33	
Hispanics	55.5 (47.8-63.3)	57.2 (50.8-63.6)	58.8 (53.7-64)	56.6 (51.9-61.3)	56.2 (47.9-64.4)	61.2 (56.4-66.1)	56.0 (53.8-58.2)	60.3 (54.2-66.4)	.38	
**Education**										
<High school graduate	63.2 (56.3-70.0)	60.9 (54.8-66.9)	59.0 (51.0-66.9)	61.1 (54.7-67.5)	61.6 (54.8-68.4)	56.2 (50.9-61.5)	57.5 (50.7-64.3)	57.3 (49.8-64.9)	.15	.05
High school graduate or GED	65.7 (58.9-72.5)	62.4 (54.0-70.7)	56.9 (52.2-61.6)	59.6 (53.7-65.4)	56.1 (47.7-64.4)	52.5 (48.1-56.9)	52.7 (46.5-58.9)	51.5 (46.2-56.8)	<.001	
Some college or above	65.8 (63.0-68.6)	58.2 (53.3-63)	65.5 (59.5-71.5)	59.4 (55.4-63.4)	59.4 (55.5-63.3)	57.0 (53.5-60.5)	55.5 (50.9-60.1)	52.3 (48.9-55.7)	<.001	
College graduate or above	68.4 (63.4-73.3)	61.8 (56.2-67.4)	58.1 (54.4-61.7)	65.7 (57.9-73.5)	64.7 (59.4-70.1)	60.6 (56.0-65.2)	63.6 (58.7-68.6)	62.4 (55.0-69.8)	.61	
**Ratio of family income to poverty level[c]**										
<1.30 [d]	62.2 (54.9-69.5)	59.6 (54.2-65)	57.5 (50.5-64.5)	60.7 (54.4-67.1)	58.2 (52.5-63.8)	58.1 (53.2-63.1)	54.1 (47.3-60.9)	53.6 (49.1-58.1)	.03	.34
1.30-3.49	64.4 (60.3-68.6)	60.6 (55.3-65.9)	64.6 (61.3-67.9)	63.4 (58.2-68.6)	60.8 (55.6-66)	51.5 (47.5-55.5)	56.5 (50.9-62.1)	55.3 (50.3-60.3)	<.001	
≥3.50 [e]	68.5 (64.7-72.3)	60.5 (57.7-63.2)	57.0 (51.9-61.9)	60.3 (56.1-64.4)	63.6 (58.6-68.2)	60.0 (55.1-64.5)	61.6 (57.7-65.4)	56.2 (48.0-64.1)	.09	

Abbreviations: NHANES, National Health and Nutrition Examination Survey.

Food sources were grouped as grocery stores (consumed food items obtained from "grocery or supermarket"), restaurants ("restaurant fast food/pizza, restaurant with waiter/waitress, restaurant no additional information"), schools ("K-12 school cafeteria, childcare center"), worksites ("cafeteria not in a K-12 school, vending machine, common coffee pot or snack tray) and other sources refer to food items obtained from elsewhere ("sport, recreation, or entertainment facility, street vendor, vending truck, from someone else/gift, and others).

[a] All percentages were survey-weighted; %, proportion.

© 2021 Liu J et al. *JAMA Network Open.*

[b] Trends in diet for the other race or mixed race group are not presented in the race/ethnicity-stratified results due to their small sample sizes. [c]Represents the ratio of family income to the federal poverty threshold, adjusting for household size. For reference, the federal threshold in 2016 for a family of 4 was $24,300/y. A family of 4 earning $33,534/y would have a ratio of 1.38.
[d] Indicates a lower level of income.
[e] Indicates a higher level of income.

© 2021 Liu J et al. *JAMA Network Open.*

eTable 20. Trends in American Heart Association Diet Score for Meals Consumed from Different Sources by Age, Sex, Race/Ethnicity, Education, and Income among American Adults aged 20+, NHANES 2003-2018

	AHA Diet Score, Weighted Mean (95% CI)[a]								P for trend	2017-2018 versus 2003-2004, difference (95% CI)
	2003-2004	2005-2006	2007-2008	2009-2010	2011-2012	2013-2014	2015-2016	2017-2018		
**Grocery stores**										
**Age group, y**										
20-49	33.8 (32.5-35.1)	35.6 (34.1-37.1)	35.7 (34.4-37.0)	36.6 (35.5-37.7)	37.4 (36.2-38.5)	37.4 (36.6-38.3)	37.2 (35.9-38.6)	37.2 (35.9-38.5)	<.001	3.35 (1.52, 5.18)
≥50	38.4 (37.3-39.6)	40.3 (39.4-41.3)	40.4 (38.6-42.2)	40.7 (39.9-41.6)	41.9 (40.8-43.1)	41.0 (39.7-42.2)	39.8 (38.9-40.7)	39.4 (38.1-40.7)	.59	0.98 (-0.74, 2.70)
P for interaction				<.001						
**Sex**										
Female	37.1 (35.8-38.4)	39.3 (38.1-40.4)	39.0 (37.6-40.5)	40.0 (39.5-40.6)	41.1 (39.9-42.3)	40.8 (39.8-41.9)	40.0 (39.0-41.0)	39.5 (38.2-40.7)	.002	2.43 (0.62, 4.23)
Male	34.3 (33.2-35.5)	35.8 (34.6-37.0)	36.2 (34.7-37.6)	36.6 (35.6-37.7)	37.7 (36.8-38.6)	37.2 (36.2-38.1)	36.8 (35.5-38.1)	36.9 (35.6-38.2)	.002	2.59 (0.86, 4.32)
P for interaction				.97						
**Race/ethnicity[b]**										
Non-Hispanic white	35.9 (34.5-37.3)	38.2 (37.0-39.4)	37.9 (35.9-39.9)	38.8 (38.0-39.7)	40.0 (38.8-41.2)	39.2 (38.1-40.4)	38.5 (37.4-39.6)	37.9 (36.5-39.3)	.03	2.0 (0.008, 4.0)
Non-Hispanic black	32.5 (30.9-34.2)	33.7 (31.4-36.1)	34.6 (33.5-35.8)	34.7 (32.9-36.4)	35.8 (33.8-37.8)	35.9 (34.6-37.2)	35.6 (34.1-37.2)	36.1 (34.2-38.0)	.001	3.59 (1.08, 6.11)
Hispanics	37.1 (35.2-38.9)	37.0 (35.7-38.2)	36.9 (35.9-37.9)	37.8 (36.5-39.1)	37.7(36.5-38.9)	38.1 (36.7-39.5)	37.5 (36.6-38.4)	39.1 (37.5-40.6)	.03	1.99 (-0.41, 4.39)
P for interaction				.31						
**Education**										
<High school graduate	34.2 (32.9-35.5)	34.8 (33.7-36.0)	34.3 (32.9-35.7)	35.7 (34.5-36.8)	35.6 (34.8-36.4)	36.0 (35.0-36.9)	35.5 (34.0-36.9)	36.5 (34.4-38.6)	.02	2.27 (-0.20, 4.73)
High school graduate or GED	33.0 (31.8-34.3)	35.4 (34.4-36.4)	36.0 (34.5-37.6)	35.0 (33.7-36.3)	36.8 (35.7-37.8)	36.1 (34.7-37.5)	35.4 (34.4-36.5)	35.5 (34.1-36.8)	.05	2.41 (0.57, 4.26)
Some college or above	35.6 (34.6-36.6)	37.4 (35.8-38.9)	37.3 (35.9-38.7)	37.6 (36.6-38.6)	38.8 (37.3-40.2)	37.8 (36.7-39.0)	37.6 (36.1-39.1)	36.6 (35.6-37.7)	.19	1.05 (-0.42, 2.51)
College graduate or above	40.3 (39.0-41.6)	41.8 (40.2-43.4)	42.6 (41.3-43.9)	44.0 (43.0-44.9)	43.9 (42.4-45.4)	44.1 (43.1-45.1)	42.6 (41.4-43.8)	43.2 (41.7-44.6)	.006	2.86 (0.92, 4.80)
P for interaction				.34						

© 2021 Liu J et al. *JAMA Network Open.*

**Ratio of family income to poverty level[c]**										
<1.30 [d]	33.4 (31.8-34.9)	34.8 (33.4-36.3)	34.8 (32.9-36.7)	35.0 (34.0-36.1)	35.7 (34.4-37.0)	35.2 (34.1-36.4)	35.5 (34.2-36.8)	35.4 (33.8-37.1)	.05	2.07 (-0.16, 4.31)
1.30-3.49	35.5 (34.0-37.1)	36.5 (35.7-37.3)	37.1 (35.6-38.6)	37.2 (36.1-38.3)	38.5 (37.6-39.5)	38.0 (37.2-38.9)	36.8 (35.7-37.9)	37.5 (35.8-39.2)	.05	1.95 (-0.35, 4.24)
≥3.50 [e]	37.3 (36.4-38.2)	39.5 (37.9-41.2)	39.3 (38.0-40.6)	41.0 (40.1-42.0)	42.6 (40.9-44.2)	42.3 (41.1-43.4)	41.2 (40.3-42.2)	40.2 (38.7-41.7)	<.001	2.91 (1.16, 4.65)
P for interaction				.18						
					**Restaurants**					
**Age group, y**										
20-49	27.7 (26.9-28.4)	27.6(26.8-28.4)	27.1 (26.4-27.8)	27.6 (26.5-28.6)	28.6 (27.7-29.4)	28.0 (27.2-28.8)	28.3 (27.5-29.2)	28.5 (27.4-29.6)	.03	0.83 (-0.47, 2.12)
≥50	30.1 (29.2-31)	29.7(28.9-30.4)	29.7 (29.1-30.4)	30.2 (29.4-31)	29.8 (28.8-30.9)	31.7 (30.7-32.8)	29.9 (28.5-31.3)	29.5 (28.6-30.5)	.77	-0.56 (-1.80, 0.70)
P for interaction				.14						
**Sex**										
Female	29.3 (28.3-30.4)	29.5 (28.6-30.4)	28.5 (27.9-29.0)	29.5 (28.7-30.2)	29.7 (28.8-30.7)	30.2 (29.6-30.9)	29.7 (28.6-30.8)	30.0 (29.1-31.0)	.07	0.69 (-0.71, 2.09)
Male	27.7 (26.7-28.7)	27.3 (26.6-28.0)	27.7 (26.9-28.5)	27.7 (26.7-28.7)	28.4 (27.5-29.4)	28.8 (27.9-29.7)	28.3 (27.2-29.3)	27.8 (27.1-28.6)	.10	0.09 (-1.10, 1.32)
P for interaction				.79						
**Race/ethnicity[b]**										
Non-Hispanic white	28.4 (27.5-29.2)	28.3 (27.7-29.0)	28.1 (27.6-28.6)	28.7 (27.8-29.6)	29.0 (28.0-29.9)	29.8 (28.9-30.7)	29.0 (27.7-30.3)	28.6 (27.7-29.6)	.08	0.26 (-1.0, 1.52)
Non-Hispanic black	27.8 (26.8-28.8)	27.0 (25.9-28.1)	26.9 (25.8-28.0)	27.3 (26.1-28.5)	27.9 (26.2-29.7)	27.6 (26.5-28.8)	27.9 (26.9-28.9)	27.9 (26.9-29.0)	.23	0.12 (-1.30, 1.57)
Hispanics	29.3 (27.8-30.7)	29.2 (27.8-30.5)	29.0 (27.9-30.1)	28.9 (28.2-29.6)	30.0 (29.3-30.6)	28.8 (27.8-29.9)	28.5 (27.6-29.3)	29.7 (28.5-31.0)	.89	0.47 (-1.40, 2.35)
P for interaction				.59						
**Education**										
<High school graduate	28.5 (27.5-29.5)	27.3 (26.2-28.3)	27.2 (26.2-28.3)	27.8 (26.6-29.0)	28.5 (27.5-29.4)	27.7 (26.9-28.5)	27.7 (26.8-28.6)	27.7 (26.1-29.3)	.86	-0.84 (-2.70, 1.04)
High school graduate or GED	26.6 (25.7-27.5)	26.9 (26.0-27.7)	26.5 (25.7-27.3)	27.0 (25.6-28.3)	26.3 (25.2-27.3)	26.9 (25.2-28.7)	26.5 (25.4-27.6)	26.7 (25.6-27.7)	.96	0.10 (-1.30, 1.46)
Some college or above	29.1 (28.3-29.9)	28.3 (27.4-29.2)	28.0 (27.2-28.9)	27.9 (27.0-28.9)	28.4 (27.2-29.6)	29.1 (28.2-30.0)	28.5 (27.5-29.6)	28.6 (27.8-29.4)	.80	-0.50 (-1.60, 0.61)
College graduate or above	29.9 (28.3-31.5)	30.3 (29.3-31.3)	30.2 (29.5-31.0)	31.0 (30.2-31.9)	31.7 (30.1-33.2)	32.4 (31.8-33.1)	31.2 (29.7-32.8)	31.6 (30.4-32.7)	.007	1.63 (-0.31, 3.57)
P for interaction				.94						

© 2021 Liu J et al. *JAMA Network Open.*

**Ratio of family income to poverty level[c]**										
<1.30 [d]	27.4 (26.3-28.6)	26.8 (25.5-28)	27.0 (25.9-28.1)	27.0 (25.9-28.1)	27.5 (26.5-28.5)	26.7 (25.2-28.2)	27.7 (26.3-29.2)	27.6 (26.3-29.0)	.52	0.20 (-1.50, 1.93)
1.30-3.49	28.7 (27.8-29.7)	27.0 (26.5-27.6)	27.2 (26.3-28.1)	28.0 (26.9-29.1)	28.0 (27.0-29.0)	28.6 (27.5-29.8)	28.1 (27.1-29.1)	28.8 (27.9-29.7)	.11	0.02 (-1.30, 1.36)
≥3.50 [e]	29.0 (28.2-29.7)	29.6 (28.9-30.4)	29.1 (28.4-29.9)	29.7 (29.0-30.4)	30.7 (29.6-31.8)	31.5 (30.8-32.2)	30.0 (28.5-31.4)	29.8 (28.5-31.0)	.03	0.80 (-0.68, 2.28)
P for interaction				.54						
**Worksites**										
**Age group, y**										
20-49	33.1 (31.7-34.5)	32.2 (31.1-33.2)	32.5 (31.5-33.4)	33.9 (32.9-34.9)	33.3 (31.5-35.0)	34.3 (33.0-35.6)	35.1 (33.6-36.5)	33.1 (31.5-34.6)	.07	-0.02 (-2.10, 2.10)
≥50	34.5 (32.8-36.1)	33.6 (32.3-35.0)	34.3 (33.1-35.4)	35.2 (33.9-36.4)	34.8 (32.7-36.9)	34.5 (31.3-37.7)	33.8 (31.5-36.0)	34.0 (31.3-36.7)	.98	-0.48 (-3.60, 2.67)
P for interaction				.28						
**Sex**										
Female	33.9 (32.6-35.2)	32.4 (30.6-34.2)	34.1 (33.3-34.9)	34.2 (33.1-35.3)	33.9 (32.3-35.5)	34.9 (33.0-36.8)	35.1 (33.0-37.1)	33.6 (31.8-35.3)	.26	-0.33 (-2.50, 1.86)
Male	33.1 (31.8-34.4)	32.9 (32.0-33.9)	32.2 (30.7-33.6)	34.4 (32.8-36.0)	33.6 (31.8-35.5)	33.9 (32.1-35.8)	34.3 (32.7-35.9)	33.2 (31.6-34.9)	.20	0.15 (-1.90, 2.24)
P for interaction				.99						
**Race/ethnicity[b]**										
Non-Hispanic white	33.8 (32.4-35.3)	32.7 (31.4-33.9)	33.3 (32.3-34.2)	34.6 (33.7-35.6)	34.2 (32.1-36.3)	34.7 (32.5-36.8)	34.6 (32.9-36.3)	33.1 (31.1-35)	.40	-0.76 (-3.20, 1.69)
Non-Hispanic black	32.5 (30.7-34.2)	34.6 (33.6-35.7)	32.8 (31.5-34.2)	33.7 (30.9-36.5)	31.9 (29.1-34.7)	34.7 (33.5-35.9)	35.3 (33.4-37.1)	35.2 (33.3-37.2)	.05	2.77 (0.15, 5.38)
Hispanics	31.9 (30.5-33.4)	31.6 (30.2-33.0)	32.5 (31.1-33.9)	33.7 (32.1-35.3)	32.4 (30.8-34.1)	33.3 (30.4-36.3)	33.0 (31.2-34.8)	33.1 (31.1-35.1)	.18	1.15 (-1.30, 3.64)
P for interaction				.69						
**Education**										
<High school graduate	32.7 (29.6-35.9)	33.5 (31.8-35.3)	32.3 (30.3-34.4)	32.5 (30.7-34.4)	32.3 (30.1-34.6)	34.5 (32.3-36.7)	34.0 (31.6-36.4)	35.6 (30.4-40.9)	.30	2.90 (-3.20, 9.03)
High school graduate or GED	33.0 (31.9-34.1)	30.3 (28.3-32.3)	31.1 (29.9-32.3)	34.6 (33.0-36.1)	32.2 (31.0-33.3)	33.0 (31.1-34.9)	32.8 (29.9-35.7)	32.9 (31.6-34.2)	.16	-0.08 (-1.80, 1.62)
Some college or above	33.2 (31.7-34.7)	33.4 (31.7-35.1)	34.2 (32.8-35.6)	33.3 (32.2-34.3)	32.1 (29.6-34.5)	32.7 (31.1-34.3)	34.2 (32.5-36)	32.7 (30.7-34.6)	.60	-0.55 (-3.0, 1.90)

© 2021 Liu J et al. *JAMA Network Open.*

College graduate or above	34.4 (31.4-37.4)	33.2 (31.3-35.2)	33.7 (31.9-35.5)	35.5 (34.3-36.7)	36.5 (33.7-39.2)	36.7 (33.9-39.5)	35.9 (34.4-37.3)	34.0 (31.5-36.5)	.17	-0.43 (-4.30, 3.48)
P for interaction				.21						
**Ratio of family income to poverty level[c]**										
<1.30 [d]	33.1 (30.6-35.7)	32.6 (31.3-33.9)	32.2 (30.9-33.5)	34.0 (32.5-35.4)	29.4 (27.0-31.8)	32.9 (31.0-34.8)	34.3 (29.7-38.8)	35.0 (32.1-37.9)	.52	1.86 (-2.0, 5.7)
1.30-3.49	33.4 (32.0-34.9)	32.5 (31.4-33.6)	32.8 (31.7-34)	33.4 (32.4-34.5)	33.7 (32.5-34.9)	33.4 (31.9-34.9)	34.9 (32.9-36.8)	32.6 (31.1-34.2)	.57	-0.78 (-2.90, 1.35)
≥3.50 [e]	33.5 (31.6-35.4)	32.8 (31.3-34.3)	33.4 (32.2-34.5)	35.1 (33.7-36.6)	35.8 (33.3-38.4)	35.3 (32.5-38.0)	35.0 (33.2-36.7)	33.9 (31.9-35.9)	.06	.39 (-2.36, 3.14)
P for interaction				.68						
				**Others**						
**Age group, y**										
20-49	35.2 (34.2-36.1)	34.9 (34.2-35.7)	33.4 (32.6-34.2)	35.0 (34.0-36.0)	34.4 (33.8-35.1)	34.2 (33.1-35.3)	34.0 (33.3-34.8)	34.0 (33.0-35.1)	.09	-1.10 (-2.60, 0.30)
≥50	37.3 (36.2-38.4)	36.1 (34.9-37.3)	37.1 (36-38.2)	36.1 (35.1-37.1)	36.9 (36.2-37.7)	35.4 (34.7-36.1)	36.0 (34.8-37.1)	35.4 (34.4-36.4)	.01	-1.90 (-3.40, -0.38)
P for interaction				.34						
**Sex**										
Female	36.2 (35.2-37.1)	36.1 (35.0-37.2)	36.0 (34.8-37.2)	36.1 (35.4-36.8)	36.7 (36.0-37.4)	35.5 (35.0-36.0)	35.7 (34.9-36.6)	35.5 (35.0-36.0)	.16	-0.66 (-1.80, 0.43)
Male	36.1 (35.1-37.1)	34.8 (34.1-35.4)	34.0 (33.1-34.9)	34.8 (33.8-35.9)	34.4 (33.7-35.2)	33.9 (32.9-35.0)	34.2 (33.4-35.1)	33.9 (32.3-35.6)	.03	-2.10 (-4.0, -0.21)
P for interaction				.26						
**Race/ethnicity[b]**										
Non-Hispanic white	36.3 (35.2-37.4)	35.6 (34.6-36.6)	35.0 (34.0-36.1)	35.6 (34.6-36.7)	35.7 (34.9-36.6)	34.1 (33.3-34.8)	34.7 (33.6-35.7)	34.2 (32.9-35.6)	.005	-2.10 (-3.80, -0.31)
Non-Hispanic black	35.0 (32.9-37.1)	34.5 (33.5-35.4)	34.2 (32.9-35.5)	34.4 (33.3-35.6)	35.8 (35.0-36.6)	35.7 (34.7-36.7)	35.1 (34.1-36.1)	34.5 (33.2-35.8)	.62	-0.48 (-3.0, 2.04)
Hispanics	34.8 (33.6-36)	34.9 (33.5-36.4)	35.1 (34.2-36.0)	34.7 (33.5-35.9)	34.5 (32.8-36.2)	36.2 (35.3-37.2)	35.0 (34.1-35.8)	35.8 (34.7-36.9)	.12	0.99 (-0.66, 2.64)
P for interaction				.006						
**Education**										
<High school graduate	36.1 (34.9-37.3)	35.3 (34.0-36.6)	34.5 (32.8-36.2)	35.2 (34.2-36.2)	35.2 (34.0-36.5)	34.5 (33.6-35.4)	34.2 (33.0-35.4)	35.3 (33.8-36.7)	.15	-0.85 (-2.80, 1.07)

© 2021 Liu J et al. *JAMA Network Open.*

High school graduate or GED	35.6 (34.0-37.2)	35.9 (33.7-38.1)	34.4 (33.3-35.5)	34.7 (33.3-36.1)	34.8 (33.2-36.4)	33.1 (31.6-34.5)	34.6 (33.3-35.8)	33.5 (32.3-34.6)	.01	-2.10 (-4.0, -0.12)
Some college or above	35.4 (34.3-36.4)	35.2 (34.1-36.4)	35.7 (34.4-37.1)	35.2 (33.9-36.5)	35.1 (34.5-35.8)	34.8 (33.9-35.7)	34.2 (32.8-35.5)	33.7 (33.0-34.3)	.002	-1.70 (-2.90, -0.49)
College graduate or above	37.5 (36.7-38.4)	35.4 (34.4-36.5)	35.4 (34.0-36.7)	36.5 (35.3-37.8)	36.5 (35.6-37.4)	35.9 (35.2-36.6)	36.2 (35.1-37.4)	36.7 (35.5-38.0)	.80	-0.77 (-2.30, 0.75)
P for interaction				.59						
**Ratio of family income to poverty level**[c]										
<1.30 [d]	35.0 (33.3-36.7)	34.7 (33.5-35.9)	34.1 (32.5-35.7)	35.6 (34.6-36.5)	34.9 (33.9-35.9)	35.0 (34.0-35.9)	34.1 (32.7-35.4)	34.2 (33.3-35.1)	.37	-0.80 (-2.70, 1.13)
1.30-3.49	35.6 (34.6-36.5)	35.4 (34.4-36.4)	35.9 (34.8-36.9)	35.6 (34.5-36.6)	35.4 (34.3-36.6)	33.9 (33.0-34.8)	34.6 (33.3-36)	34.7 (33.6-35.9)	.04	-0.83 (-2.30, 0.69)
≥3.50 [e]	36.9 (35.9-37.9)	35.7 (34.8-36.6)	35.0 (33.9-36.0)	35.5 (34.4-36.6)	36.2 (35.1-36.8)	35.2 (34.4-36)	35.8 (34.9-36.7)	34.9 (33.5-36.3)	.20	-1.95 (-3.64, -0.27)
P for interaction				.76						

Abbreviations: NHANES, National Health and Nutrition Examination Survey.

Food sources were grouped as grocery stores (consumed food items obtained from “grocery or supermarket”), restaurants (“restaurant fast food/pizza, restaurant with waiter/waitress, restaurant no additional information”), schools (“K-12 school cafeteria, childcare center”), worksites (“cafeteria not in a K-12 school, vending machine, common coffee pot or snack tray) and other sources refer to food items obtained from elsewhere (“sport, recreation, or entertainment facility, street vendor, vending truck, from someone else/gift, and others).

[a] All percentages were survey-weighted; %, proportion.

[b] Trends in diet for the other race or mixed race group are not presented in the race/ethnicity-stratified results due to their small sample sizes.

[c] Represents the ratio of family income to the federal poverty threshold, adjusting for household size. For reference, the federal threshold in 2016 for a family of 4 was $24,300/y. A family of 4 earning $33,534/y would have a ratio of 1.38.

[d] Indicates a lower level of income.

[e] Indicates a higher level of income.

© 2021 Liu J et al. *JAMA Network Open.*

eTable 21. Trends in Healthy Eating Index-2015 for Meals Consumed from Different Sources by Age Group, Sex, Race/Ethnicity, Education, and Income among American Adults aged 20 years +, NHANES 2003-2018

	HEI-2015, Weighted Mean (95% CI)[a]								P for trend	2017-2018 versus 2003-2004, difference (95% CI)
	2003-2004	2005-2006	2007-2008	2009-2010	2011-2012	2013-2014	2015-2016	2017-2018		
**Grocery stores**										
**Age group, y**										
20-49	49.9 (48.6-51.2)	50.4 (49.1-51.7)	50.7 (49.2-52.1)	52.4 (51.4-53.4)	52.9 (51.9-53.8)	52.5 (51.5-53.5)	52.3 (50.8-53.9)	51.0 (49.5-52.5)	.01	1.12 (-0.86, 3.10)
≥50	55.5 (54.7-56.3)	56.1 (55.0-57.2)	57.1 (55.0-59.2)	57.3 (56.3-58.3)	57.8 (56.6-58.9)	56.9 (55.8-58.0)	55.6 (54.3-56.9)	54.7 (53.1-56.3)	.22	-0.79 (-2.60, 1.01)
P for interaction				<.001						
**Sex**										
Female	53.0 (51.7-54.4)	54.3 (53.2-55.3)	54.4 (52.7-56.2)	56.0 (55.3-56.8)	56.5 (55.3-57.6)	56.0 (54.9-57.2)	55.1 (53.7-56.6)	53.7 (51.9-55.5)	.22	0.68 (-1.60, 2.91)
Male	51.3 (50.6-52.1)	51.2 (49.9-52.5)	52.2 (50.6-53.8)	52.9 (52-53.8)	53.7 (53.0-54.4)	52.9 (51.9-54)	52.6 (51.0-54.1)	51.8 (50.5-53)	.14	0.42 (-1.10, 1.92)
P for interaction				.95						
**Race/ethnicity[b]**										
Non-Hispanic white	52.1 (50.9-53.2)	53.3 (52.3-54.3)	53.6 (51.4-55.9)	55.1 (54.2-56.1)	55.6 (54.4-56.8)	54.8 (53.6-56)	54.3 (52.9-55.6)	52.3 (50.5-54.2)	.26	0.29 (-1.90, 2.47)
Non-Hispanic black	50.7 (49.6-51.7)	49.7 (47.7-51.7)	50.5 (49.9-51.2)	51 (49.2-52.7)	51.9 (49.5-54.2)	51.3 (50.4-52.3)	50.3 (48.7-52.0)	51.1 (49.2-53)	.37	0.47 (-1.70, 2.62)
Hispanics	54.2 (52.5-55.9)	52.7 (51.2-54.2)	53.3 (52.5-54.2)	53.6 (52.2-54.9)	54.2 (53.1-55.3)	54.0 (52.2-55.8)	52.8 (51.3-54.4)	53.7 (51.9-55.5)	.96	-0.48 (-2.90, 2.0)
P for interaction				.66						
**Education**										
<High school graduate	50.6 (49.2-52.1)	51.0 (49.7-52.4)	49.9 (48.3-51.5)	51.7 (50.5-52.9)	51.8 (50.7-52.8)	50.9 (49.6-52.1)	50.3 (48.7-51.9)	50.5 (48.4-52.6)	.99	-0.12 (-2.70, 2.44)
High school graduate or GED	49.8 (48.7-51.0)	49.8 (48.6-51.1)	51.7 (50.2-53.3)	50.8 (49.9-51.7)	52.9 (51.9-53.9)	51.0 (49.9-52.1)	50.7 (49.5-52.0)	49.6 (47.5-51.7)	.99	-0.25 (-2.70, 2.16)
Some college or above	52.0 (50.7-53.4)	52.3 (51.1-53.4)	53.0 (51.5-54.6)	53.7 (52.7-54.6)	54.0 (52.1-55.8)	53.2 (51.8-54.7)	53.1 (51.4-54.8)	51.0 (49.4-52.6)	.77	-1.0 (-3.20, 1.06)
College graduate or above	56.4 (55.2-57.6)	57.4 (55.8-59.1)	58.4 (56.5-60.2)	60.5 (59.5-61.5)	59.5 (57.6-61.4)	60.3 (59.1-61.5)	58.4 (56.8-59.9)	58.3 (56.5-60.2)	.09	1.93 (-0.30, 4.16)
P for interaction				.95						
**Ratio of family income to poverty level[c]**										

© 2021 Liu J et al. *JAMA Network Open.*

	HEI-2015, Weighted Mean (95% CI)[a]								P for trend	2017-2018 versus 2003-2004, difference (95% CI)
	2003-2004	2005-2006	2007-2008	2009-2010	2011-2012	2013-2014	2015-2016	2017-2018		
<1.30 [d]	49.8 (48.0-51.5)	50.4 (48.9-51.8)	50.0 (48.2-51.8)	50.7 (49.7-51.7)	51.4 (50.2-52.6)	50.6 (49.4-51.8)	50.5 (49.2-51.8)	50.0 (48.1-51.8)	.69	0.19 (-2.40, 2.74)
1.30-3.49	51.9 (50.2-53.6)	51.5 (50.5-52.4)	52.7 (51.1-54.3)	53.5 (52.5-54.6)	54.0 (52.8-55.1)	53.3 (52.3-54.3)	52.1 (50.9-53.3)	52.2 (50.4-53.9)	.52	0.24 (-2.20, 2.71)
≥3.50 [e]	53.8 (52.9-54.7)	54.9 (53.3-56.6)	55.2 (53.5-56.9)	57.1 (56.0-58.3)	58.4 (57.1-59.8)	57.9 (56.6-59.3)	56.9 (55.4-58.4)	54.6 (52.6-56.6)	.03	0.76 (-1.43, 2.96)
P for interaction				.44						
					**Restaurants**					
**Age group, y**										
20-49	40.0 (39.0-41.0)	39.3 (38.6-39.9)	38.4 (37.5-39.2)	39.4 (38.4-40.3)	40.9 (39.9-41.9)	39.6 (38.8-40.4)	39.8 (38.9-40.6)	39.4 (38.3-40.5)	.54	-0.56 (-2.0, 0.89)
≥50	41.0 (40.1-41.9)	40.7 (39.8-41.6)	40.8 (40.2-41.3)	40.8 (39.8-41.7)	41.5 (40.6-42.4)	42.3 (41.5-43.1)	40.5 (39.3-41.6)	38.8 (37.6-40.1)	.07	-2.10 (-3.70, -0.58)
P for interaction				.02						
**Sex**										
Female	40.7 (39.7-41.8)	40.1 (39.1-41.0)	39.4 (38.7-40.1)	40.3 (39.6-41.1)	41.4 (40.8-42.1)	41.3 (40.7-41.8)	40.2 (39.0-41.3)	40.0 (38.9-41.2)	.78	-0.69 (-2.20, 0.86)
Male	40.0 (39.0-41)	39.5 (38.9-40.2)	39.1 (38.3-39.9)	39.5 (38.5-40.4)	41.0 (40.0-41.9)	40.1 (39.3-40.9)	39.9 (39.0-40.9)	38.3 (37.1-39.4)	.34	-1.70 (-3.20, -0.16)
P for interaction				.28						
**Race/ethnicity[b]**										
Non-Hispanic white	40.1 (39.1-41.2)	39.8 (39.0-40.6)	39.2 (38.4-40.0)	40.1 (39.2-41.0)	41.1 (40.1-42.2)	40.9 (40.1-41.7)	40.2 (38.9-41.5)	38.7 (37.4-40.1)	.79	-1.40 (-3.10, 0.30)
Non-Hispanic black	40.0 (38.5-41.6)	39.3 (38.1-40.6)	39.4 (38.5-40.2)	39.2 (37.9-40.5)	40.9 (39.8-42.0)	40.0 (39.1-40.8)	40.2 (39.0-41.4)	39.1 (38.1-40.1)	.97	-0.97 (-2.80, 0.87)
Hispanics	41.1 (39.6-42.6)	40.2 (39.4-41.1)	39.4 (38.4-40.4)	39.7 (39.0-40.5)	41.9 (41.3-42.6)	39.6 (38.6-40.6)	39.0 (38.0-40.1)	40.2 (39.1-41.4)	.26	-0.89 (-2.80, 0.98)
P for interaction				.65						
**Education**										
<High school graduate	39.7 (38.5-40.8)	38.9 (37.8-40.0)	37.8 (36.9-38.7)	39.0 (37.9-40.1)	40.5 (39.3-41.8)	39.0 (38.0-39.9)	37.9 (36.7-39.1)	37.8 (36.2-39.4)	.19	-1.90 (-3.80, 0.12)
High school graduate or GED	38.6 (37.6-39.7)	38.6 (37.6-39.5)	38.2 (37.4-38.9)	39.1 (37.6-40.5)	39.2 (38.2-40.2)	38.4 (37.0-39.9)	38.7 (37.6-39.9)	36.3 (34.9-37.7)	.04	-2.40 (-4.10, -0.59)
Some college or above	40.7 (39.7-41.7)	39.4 (38.8-40.1)	39.8 (39.2-40.4)	39.3 (38.3-40.3)	40.9 (39.4-42.3)	40.8 (40.3-41.3)	39.4 (38.6-40.3)	39.5 (38.4-40.6)	.51	-1.20 (-2.70, 0.31)

© 2021 Liu J et al. *JAMA Network Open.*

	HEI-2015, Weighted Mean (95% CI)[a]								P for trend	2017-2018 versus 2003-2004, difference (95% CI)
	2003-2004	2005-2006	2007-2008	2009-2010	2011-2012	2013-2014	2015-2016	2017-2018		
College graduate or above	42.1 (40.6-43.7)	41.7 (40.8-42.6)	40.6 (39.6-41.6)	41.8 (40.5-43.0)	42.9 (41.5-44.4)	42.8 (41.8-43.8)	42.1 (40.5-43.7)	41.6 (40.1-43.2)	.53	-0.49 (-2.70, 1.71)
P for interaction				.30						
**Ratio of family income to poverty level[c]**										
<1.30 [d]	38.8 (37.5-40.2)	38.0 (36.8-39.1)	37.8 (36.5-39.1)	38.4 (37.5-39.4)	39.7 (38.8-40.6)	38.2 (37.1-39.2)	38.4 (37.0-39.8)	38.3 (36.7-39.9)	.98	-0.53 (-2.60, 1.57)
1.30-3.49	40.5 (39.6-41.4)	38.5 (37.8-39.2)	38.5 (37.4-39.6)	39.6 (38.8-40.3)	40.1 (39.0-41.2)	39.8 (38.8-40.9)	39.0 (38.2-39.7)	38.5 (37.4-39.5)	.16	-2.10 (-3.40, -0.66)
≥3.50 [e]	41.1 (40.1-42.0)	41.2 (40.2-42.3)	40.5 (39.9-41.1)	40.8 (39.6-41.9)	42.7 (41.5-43.9)	42.7 (42.0-43.4)	41.6 (40.1-43.1)	39.9 (38.3-41.6)	.85	-1.13 (-3.06, 0.80)
P for interaction				.48						
					**Worksites**					
**Age group, y**										
20-49	38.0 (37.0-38.9)	37.4 (36.4-38.5)	36.3 (34.8-37.9)	38.6 (36.8-40.5)	40.7 (38.7-42.8)	39.5 (38.1-40.8)	39.8 (37.9-41.8)	39.5 (37.6-41.3)	<.001	1.52 (-0.53, 3.58)
≥50	40.5 (37.9-43.0)	38.6 (35.8-41.4)	38.9 (37.0-40.7)	42.2 (40.1-44.4)	40.1 (37.3-43.0)	40.0 (36.8-43.2)	39.6 (36.9-42.4)	40.0 (36-44.1)	.76	-0.41 (-5.20, 4.39)
P for interaction				.20						
**Sex**										
Female	39.9 (38.3-41.5)	38.3 (36.1-40.5)	37.8 (35.9-39.6)	40.4 (38.2-42.7)	42.0 (39.7-44.2)	41.3 (38.8-43.7)	40.8 (38.1-43.5)	40.4 (38.6-42.2)	.04	0.46 (-2.0, 2.89)
Male	37.5 (36.0-39.0)	37.6 (36.0-39.1)	36.7 (35.0-38.4)	39.3 (36.8-41.9)	39.3 (36.5-42.1)	38.5 (36.8-40.2)	39.0 (37.0-40.9)	39.1 (35.9-42.2)	.09	1.58 (-1.9, 5.06)
P for interaction				.86						
**Race/ethnicity[b]**										
Non-Hispanic white	38.8 (37.1-40.5)	37.9 (36.3-39.6)	37.2 (35.4-39.0)	40.7 (39.2-42.3)	40.4 (37.4-43.4)	40.0 (37.9-42.1)	39.8 (37.7-41.8)	39.2 (36.1-42.3)	.14	0.39 (-3.10, 3.91)
Non-Hispanic black	37.6 (35.0-40.1)	39.5 (37.7-41.4)	37.7 (35.9-39.4)	40.2 (37.3-43.0)	40.1 (39.2-41.1)	40.6 (38.7-42.5)	40.3 (37.5-43.1)	41.4 (39.0-43.8)	.01	3.83 (0.31, 7.34)
Hispanics	36.6 (33.7-39.6)	35.4 (33.2-37.6)	36.6 (34.1-39.0)	37.3 (34.7-39.8)	39.7 (37.3-42.2)	39.4 (35.9-42.8)	37.8 (35.1-40.5)	38.7 (36.3-41.1)	.02	2.05 (-1.80, 5.85)
P for interaction				.59						

© 2021 Liu J et al. *JAMA Network Open.*

	HEI-2015, Weighted Mean (95% CI)[a]								P for trend	2017-2018 versus 2003-2004, difference (95% CI)
	2003-2004	2005-2006	2007-2008	2009-2010	2011-2012	2013-2014	2015-2016	2017-2018		
**Education**										
<High school graduate	37.5 (33.7-41.3)	37.3 (34.9-39.7)	37.9 (35.0-40.8)	37.5 (34.8-40.2)	35.1 (32.5-37.6)	39.7 (38.3-41.1)	37.0 (34.4-39.6)	43.7 (30.1-57.4)	.43	6.26 (-7.90, 20.4)
High school graduate or GED	37.1 (35.6-38.6)	35.1 (34.1-36.1)	35.5 (33.6-37.4)	39.3 (36.7-41.8)	38.0 (35.4-40.6)	36.9 (34.8-39)	37.8 (34.5-41.1)	38.0 (36.4-39.6)	.05	0.88 (-1.30, 3.05)
Some college or above	39.3 (37.8-40.8)	37.8 (35.8-39.7)	37.0 (34.9-39.1)	40.3 (38.6-42.0)	39.5 (37.1-41.8)	38.1 (36.5-39.8)	40.6 (37.8-43.4)	39.6 (37.6-41.6)	.20	0.28 (-2.20, 2.76)
College graduate or above	39.3 (35.7-42.9)	40.1 (37.9-42.4)	38.4 (35.6-41.2)	40.7 (38.4-43.0)	44.3 (39.9-48.7)	42.6 (39.4-45.9)	40.4 (38.3-42.6)	40.2 (37.5-42.9)	.16	0.87 (-3.60, 5.36)
P for interaction				.85						
**Ratio of family income to poverty level[c]**										
<1.30 [d]	37.6 (35.0-40.3)	36.6 (35.5-37.8)	33.7 (31.8-35.5)	38.4 (35.5-41.3)	38.9 (36.0-41.8)	36.9 (35.0-38.9)	40.5 (35.9-45.1)	43.6 (35.2-51.9)	.06	5.94 (-2.80, 14.7)
1.30-3.49	39.6 (38.1-41.2)	36.8 (35.2-38.4)	37.5 (36.0-39.1)	39.6 (37.7-41.6)	38.8 (37.1-40.5)	39.2 (37.6-40.7)	39.6 (37.5-41.7)	38.3 (36.9-39.7)	.64	-1.40 (-3.50, 0.77)
≥3.50 [e]	38.3 (35.9-40.6)	38.8 (36.7-40.8)	37.8 (35.2-40.3)	40.7 (39.0-42.4)	42.7 (39.0-46.5)	40.5 (37.6-43.4)	39.9 (37.9-42.0)	40.0 (37.8-42.2)	.03	1.78 (-1.43, 4.98)
P for interaction				.12						
				**Others**						
**Age group, y**										
20-49	40.9 (39.9-41.9)	40.4 (39.3-41.5)	39.5 (38.5-40.5)	40.6 (39.8-41.4)	36.3 (35.0-37.7)	35.7 (34.6-36.8)	36.0 (34.9-37.2)	35.5 (33.4-37.7)	<.001	-5.30 (-7.70, -3.0)
≥50	44.0 (42.6-45.4)	42.1 (40.8-43.4)	42.8 (41.4-44.1)	41.4 (40.3-42.6)	40.1 (38.9-41.4)	38.6 (36.8-40.3)	38.6 (37.2-40.1)	36.9 (35.2-38.6)	<.001	-7.10 (-9.40, -4.90)
P for interaction				.67						
**Sex**										
Female	42.3 (41.5-43.2)	41.4 (40.1-42.8)	41.9 (40.9-43.0)	41.9 (41.2-42.5)	39.2 (38.1-40.4)	37.6 (36.8-38.4)	39.0 (37.2-40.7)	37.0 (35.6-38.4)	<.001	-5.30 (-7.0, -3.7)
Male	42.2 (40.9-43.5)	40.9 (40.0-41.7)	39.9 (38.5-41.2)	40.0 (38.8-41.1)	36.9 (35.9-37.8)	36.6 (35.2-37.9)	35.7 (34.5-36.8)	35.4 (34.2-36.7)	<.001	-6.80 (-8.60, -5.0)
P for interaction				.07						
**Race/ethnicity[b]**										

© 2021 Liu J et al. *JAMA Network Open.*

	HEI-2015, Weighted Mean (95% CI)[a]								P for trend	2017-2018 versus 2003-2004, difference (95% CI)
	2003-2004	2005-2006	2007-2008	2009-2010	2011-2012	2013-2014	2015-2016	2017-2018		
Non-Hispanic white	42.6 (41.8-43.4)	41.6 (40.5-42.7)	40.9 (39.8-42.1)	41.2 (40.3-42.1)	37.9 (36.9-38.9)	36.4 (35.1-37.7)	37.5 (36.4-38.6)	35.9 (34.3-37.4)	<.001	-6.70 (-8.50, -5.0)
Non-Hispanic black	41.8 (40.1-43.5)	39.3 (38.2-40.3)	40.3 (38.6-42)	40.1 (38.9-41.4)	38.0 (36.6-39.4)	36.8 (35.2-38.5)	35.3 (32.3-38.3)	35.3 (33.0-37.7)	<.001	-6.50 (-9.40, -3.50)
Hispanics	40.6 (39.1-42.1)	39.4 (37.7-41.2)	40.3 (38.8-41.8)	39.9 (38.6-41.2)	38.0 (36.0-39.9)	39.6 (37.9-41.3)	38.3 (36.6-39.9)	37.6 (35.4-39.7)	.01	-3.0 (-5.70, -0.40)
P for interaction				.002						
**Education**										
<High school graduate	43.0 (40.9-45.1)	40.9 (39.5-42.4)	41.9 (40.6-43.1)	41.4 (39.8-43.0)	37.8 (35.3-40.2)	37.7 (35.5-40.0)	37.4 (35.4-39.4)	37.5 (34.8-40.1)	<.001	-5.50 (-8.90, -2.20)
High school graduate or GED	41.5 (40.1-42.8)	42.7 (40.5-44.8)	40.0 (38.4-41.6)	40.8 (39.1-42.4)	38.6 (36.2-41.1)	35.9 (33.9-37.8)	36.3 (34.6-38.1)	36.1 (33.6-38.5)	<.001	-5.40 (-8.20, -2.60)
Some college or above	42.3 (41.3-43.4)	40.4 (39.0-41.8)	42.0 (40.6-43.5)	40.5 (38.9-42.0)	37.2 (35.9-38.4)	37.1 (35.4-38.7)	37.9 (36.1-39.8)	33.8 (31.9-35.7)	<.001	-8.50 (-11.0 -6.40)
College graduate or above	42.5 (41.0-44.1)	40.9 (39.2-42.6)	40.2 (38.6-41.9)	41.4 (39.4-43.4)	38.8 (37.5-40.1)	37.5 (36.2-38.9)	37.2 (35.5-38.8)	38.4 (35.7-41)	<.001	-4.20 (-7.20, -1.10)
P for interaction				.70						
**Ratio of family income to poverty level**[c]										
<1.30 [d]	42.0 (40.9-43.1)	40.9 (39.5-42.3)	41.0 (39.7-42.2)	41.5 (40.2-42.8)	37.0 (35.2-38.8)	37.5 (36.1-38.9)	37.1 (34.9-39.2)	34.4 (30.4-38.4)	<.001	-7.60 (-12.0, -3.40)
1.30-3.49	42.6 (41.6-43.6)	40.8 (39.1-42.4)	42.8 (41.4-44.1)	41.1 (39.9-42.3)	39.3 (37.7-41)	37.2 (35.9-38.5)	36.4 (34.6-38.1)	35.9 (34.4-37.5)	<.001	-6.70 (-8.50, -4.80)
≥3.50 [e]	41.8 (40.8-42.9)	41.4 (40.3-42.5)	39.5 (38.1-41.0)	40.8 (39.7-41.8)	38.0 (36.5-39.4)	36.9 (35.5-38.3)	38.1 (36.5-39.8)	36.6 (34.4-38.8)	<.001	-5.20 (-7.62, -2.79)
P for interaction				.24						

Abbreviations: NHANES, National Health and Nutrition Examination Survey.
Food sources were grouped as grocery stores (consumed food items obtained from “grocery or supermarket”), restaurants (“restaurant fast food/pizza, restaurant with waiter/waitress, restaurant no additional information”), schools (“K-12 school cafeteria, childcare center”), worksites (“cafeteria not in a K-12 school, vending machine, common coffee pot or snack tray) and other sources refer to food items obtained from elsewhere (“sport, recreation, or entertainment facility, street vendor, vending truck, from someone else/gift, and others).
[a] All percentages were survey-weighted; %, proportion.
[b] Trends in diet for the other race or mixed race group are not presented in the race/ethnicity-stratified results due to their small sample sizes.
[c] Represents the ratio of family income to the federal poverty threshold, adjusting for household size. For reference, the federal threshold in 2016 for a family of 4 was $24,300/y. A family of 4 earning $33,534/y would have a ratio of 1.38. [d] Indicates a lower level of income. [e] Indicates a higher level of income.

© 2021 Liu J et al. *JAMA Network Open.*

eFigure 1. Trends in Estimated Percentage of Energy Intake from Grocery Stores, Restaurants, Schools or Worksites, and Other Sources Among US Children Aged 5-19 Years (A: top) and Adults Age 20+ Years (B: bottom), NHANES 2003-2004 to 2017-2018.

Data were weighted to be nationally representative.

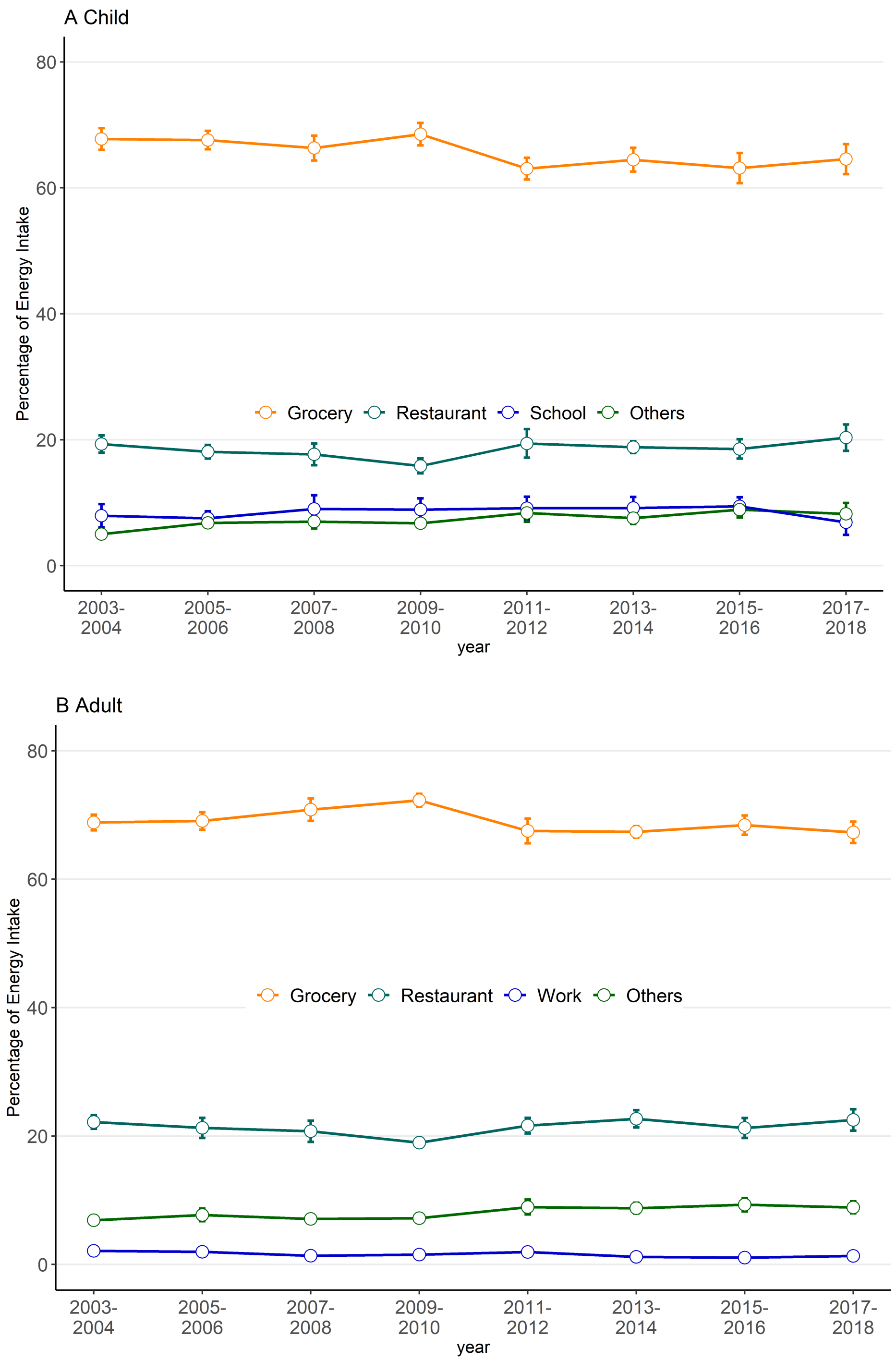

© 2021 Liu J et al. *JAMA Network Open.*

eFigure 2. Trends in Estimated Proportions of Americans Consuming Foods from Grocery Stores (A), Restaurants (B), Schools or Worksites (C), and Other Sources (D) with Poor, Intermediate or Ideal Diet Quality Among Children Aged 5 to 19 Years (left) and Adults Aged 20 Years or Older (right), NHANES 2003-2004 to 2017-2018.

Data were weighted to be nationally representative.

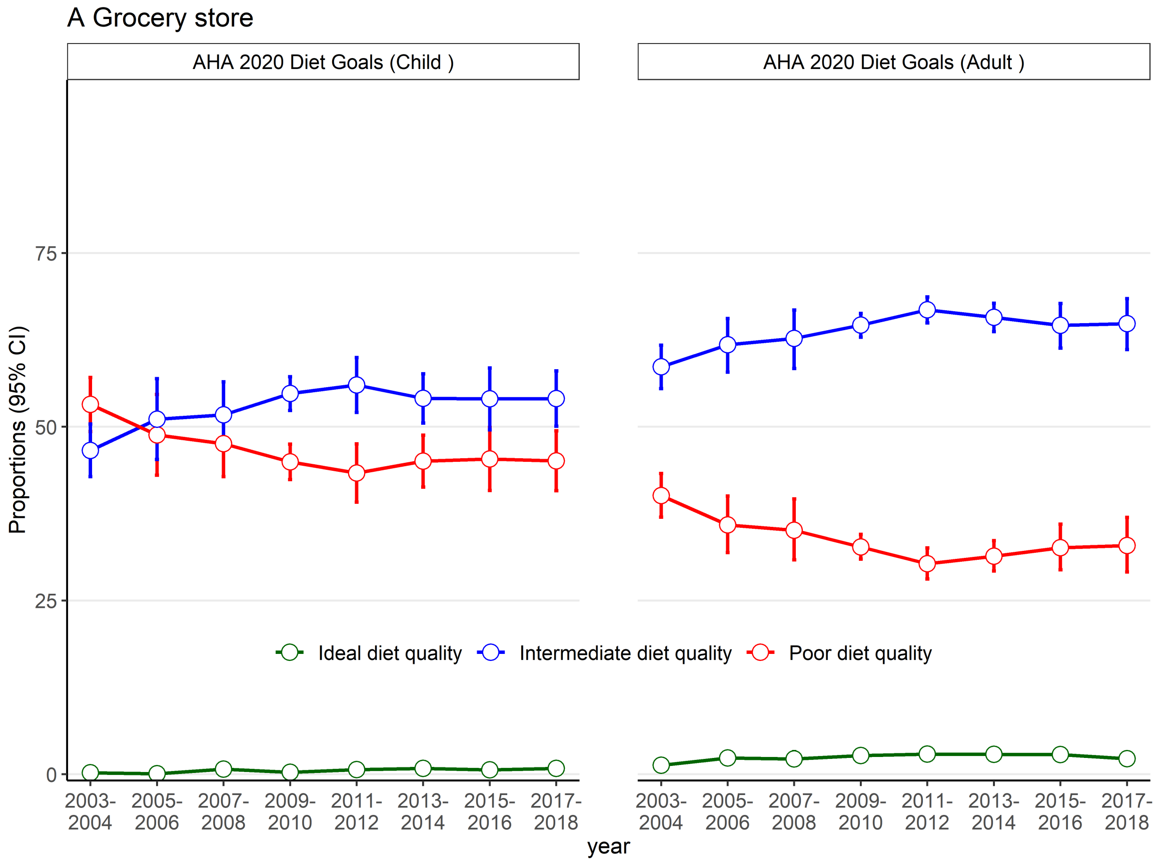

© 2021 Liu J et al. *JAMA Network Open.*

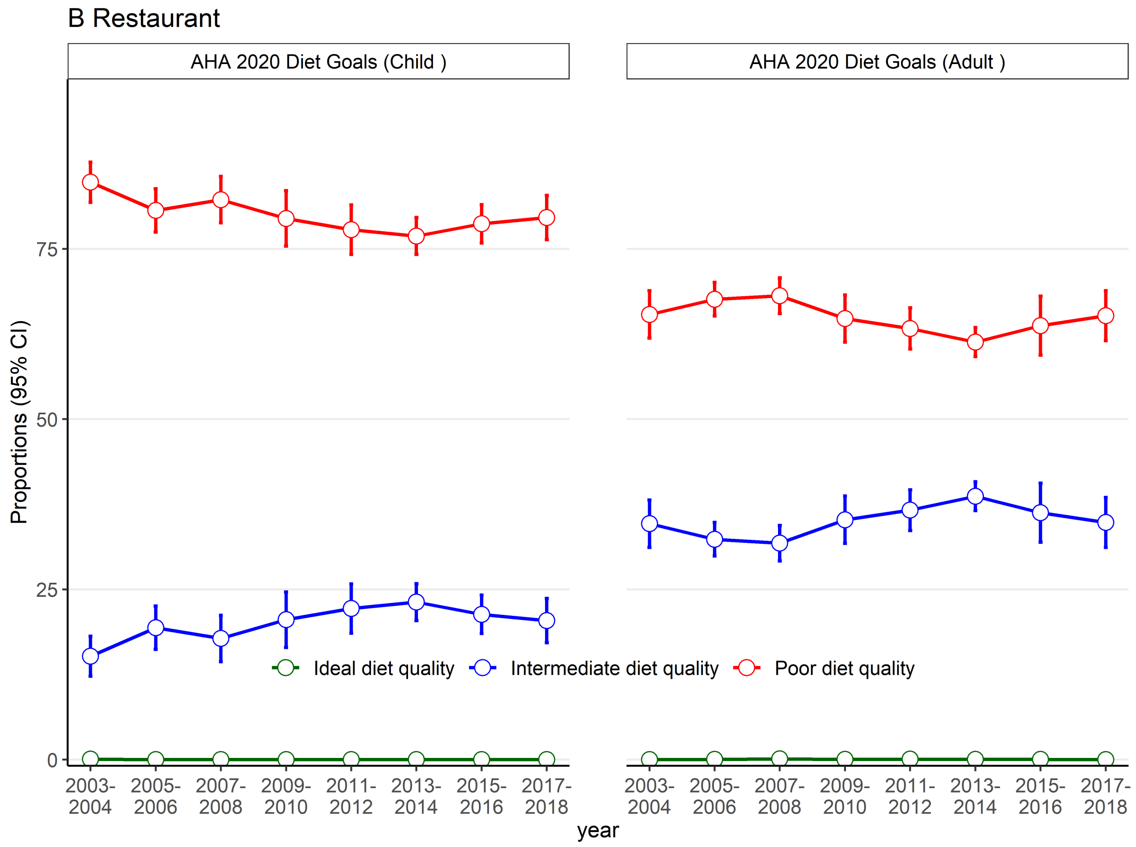

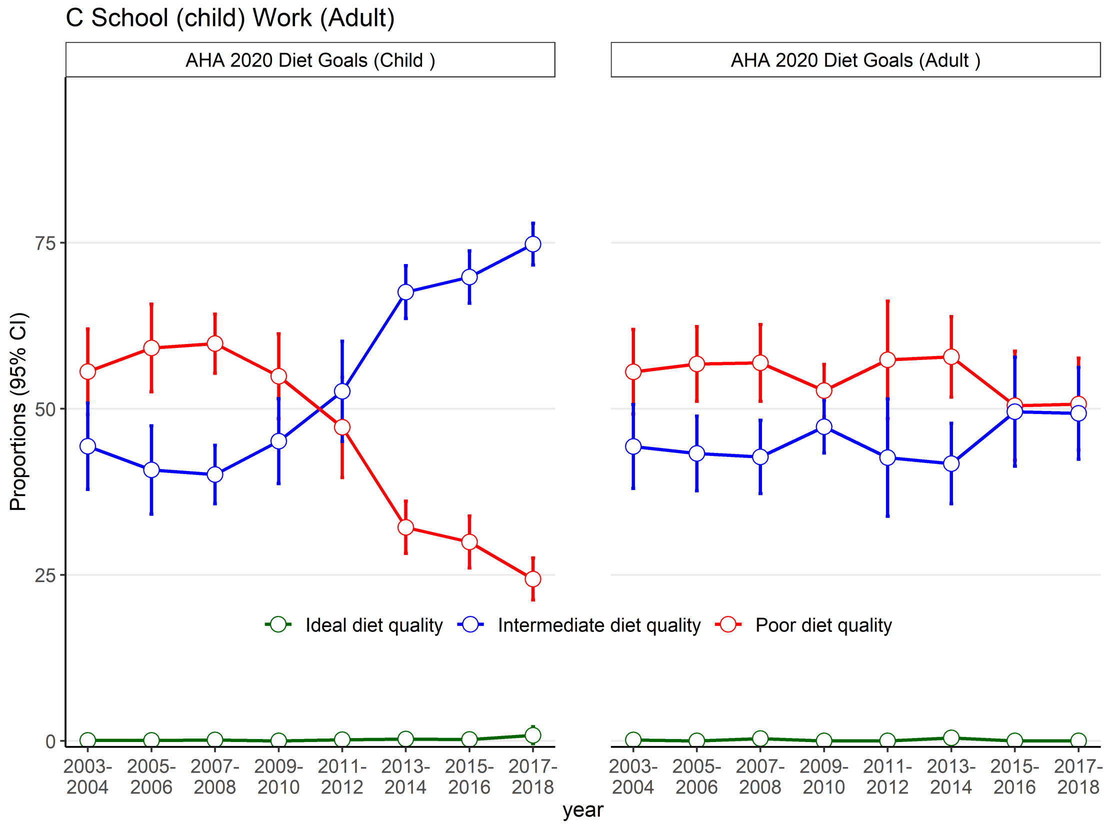

© 2021 Liu J et al. *JAMA Network Open.*

D Others

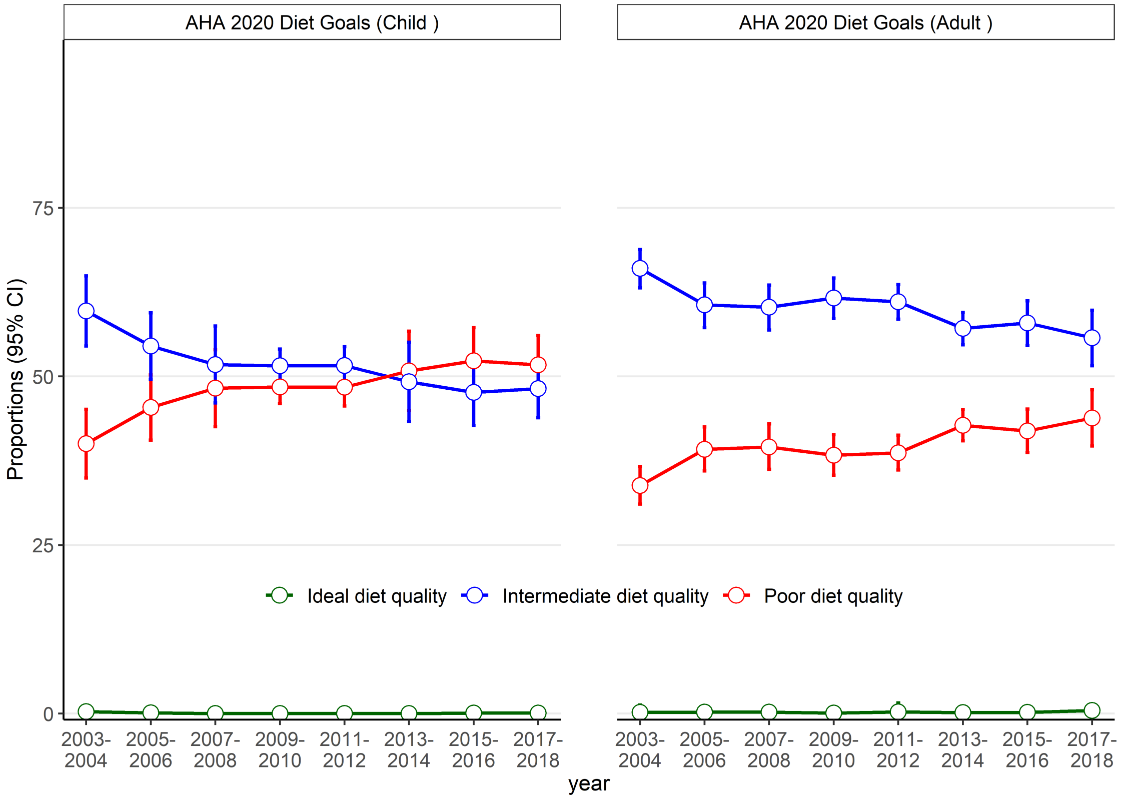

© 2021 Liu J et al. *JAMA Network Open.*

eFigure 3. Trends in Estimated Proportions of Americans Consuming Foods from Schools with Poor, Intermediate or Ideal Diet Quality Among Children Aged 5 to 19 Years by Age (top) and Sex (bottom), NHANES 2003-2004 to 2017-2018.

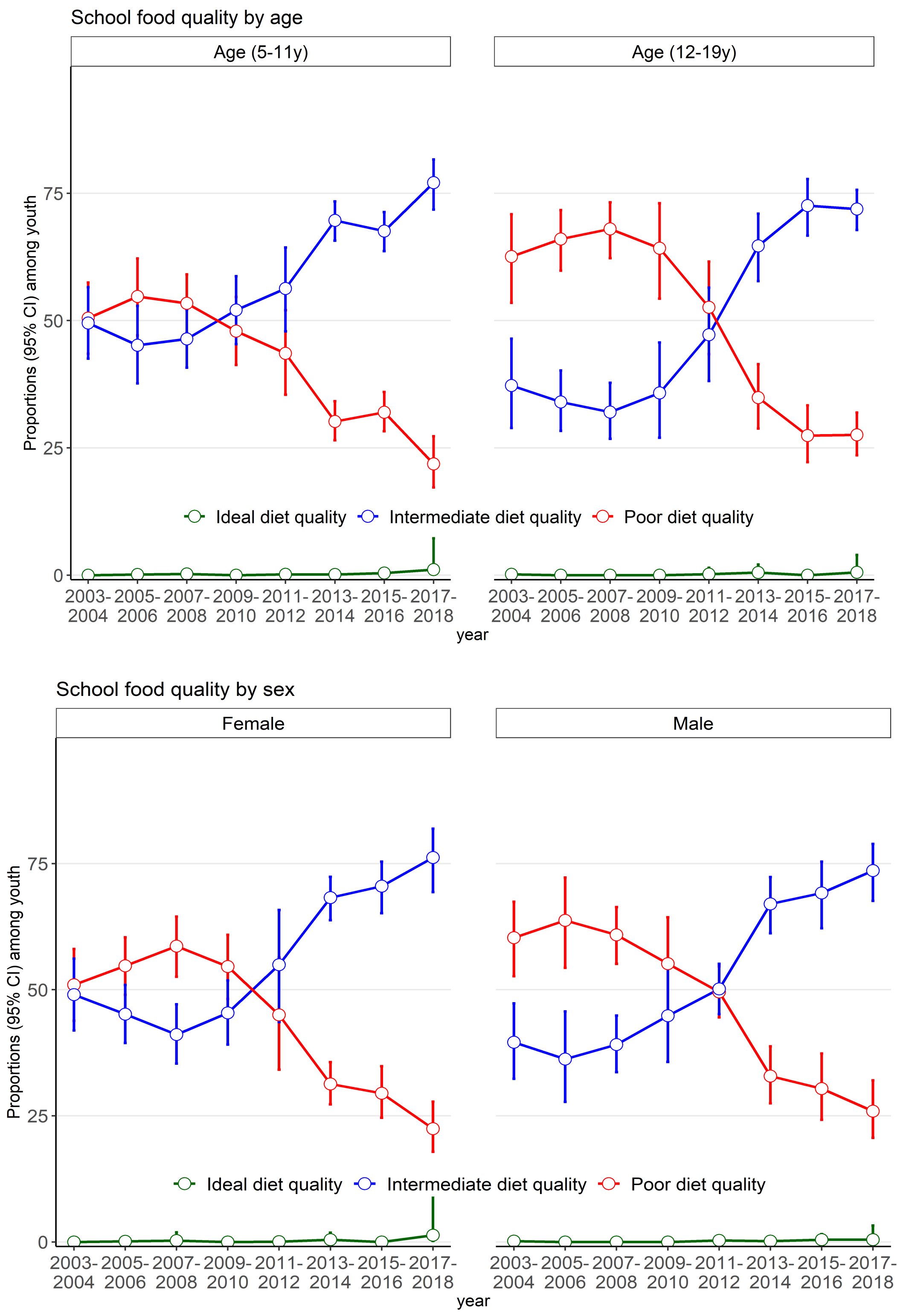

© 2021 Liu J et al. *JAMA Network Open.*

eFigure 4. Trends in Estimated Proportions of Americans Consuming Foods from Grocery with Poor, Intermediate or Ideal Diet Quality by Age for Children (top) and Adults (bottom), NHANES 2003-2004 to 2017-2018.

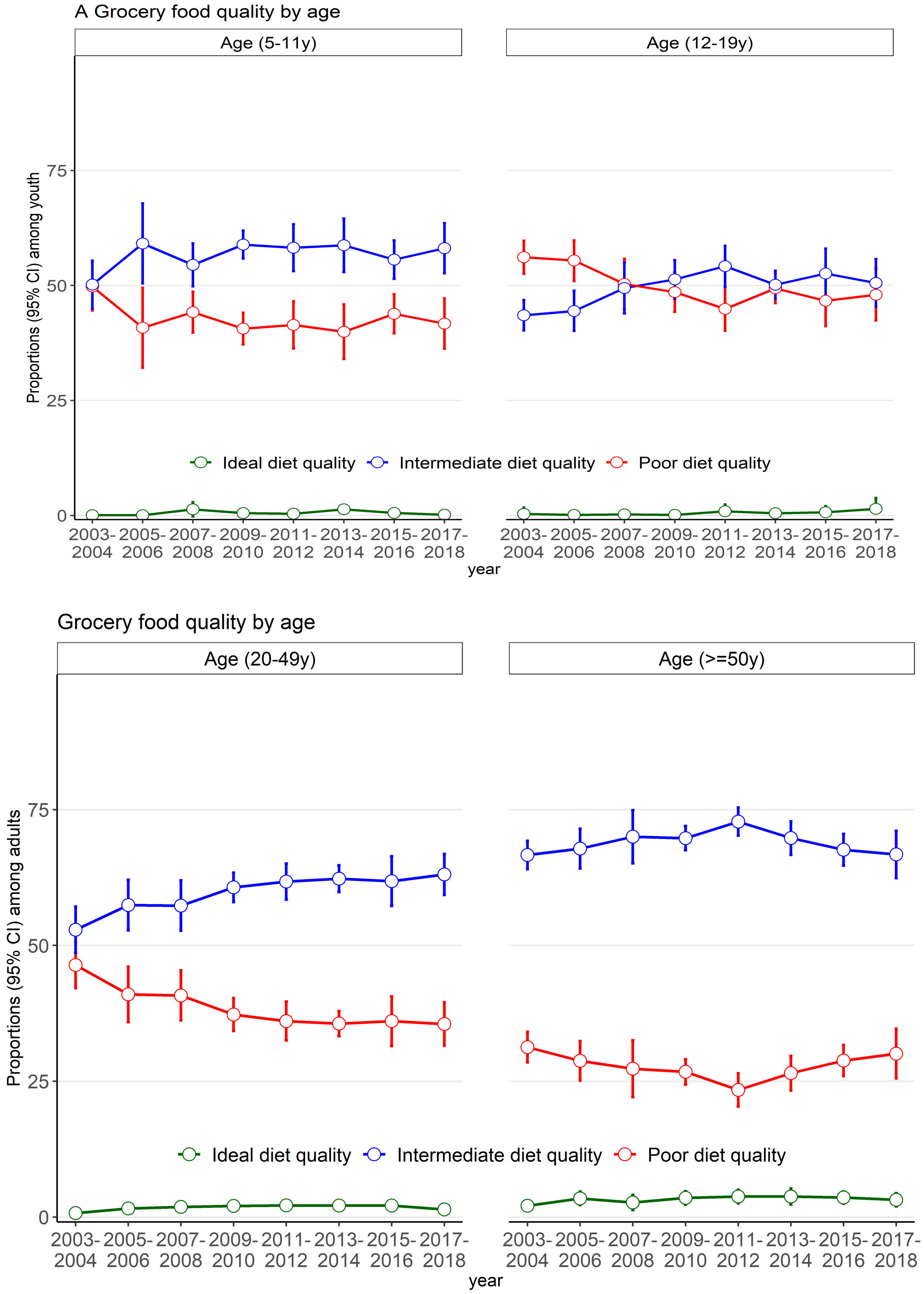

© 2021 Liu J et al. *JAMA Network Open.*

References

1. American Association for Public Opinion Research. Standard definitions: final disposition of case codes and outcome rates for surveys. Available at, https://www.aapor.org/AAPOR_Main/media/publications/Standard-Definitions20169theditionfinal.pdf. Accessed on December 1, 2020.
2. Centers for Disease Prevention and Control. NHANES Survey Methods and Analytic Guidelines. Avaliable at: https://wwwn.cdc.gov/nchs/nhanes/analyticguidelines.aspx. Accessed on 4/10/2020. .
3. American Association for Public Opinion Research. Standard definitions: final disposition of case codes and outcome rates for surveys. Available at, https://www.aapor.org/AAPOR_Main/media/publications/Standard-Definitions20169theditionfinal.pdf. Accessed on December 1, 2020.
4. Mozaffarian D, Benjamin EJ, Go AS, et al. Heart disease and stroke statistics--2015 update: a report from the American Heart Association. *Circulation.* 2015;131(4):e29-322.
5. Rehm CD, Penalvo JL, Afshin A, Mozaffarian D. Dietary Intake Among US Adults, 1999-2012. *JAMA.* 2016;315(23):2542-2553.
6. U.S. Department of Agriculture Food and Nutrition Service. Healthy Eating Index (HEI). Available at, https://www.fns.usda.gov/resource/healthy-eating-index-hei. Accessed on May 19, 2019.

© 2021 Liu J et al. *JAMA Network Open.*
